# Confronting historical legacies of biological anthropology in South Africa—Restitution, redress and community-centered science: The Sutherland Nine

**DOI:** 10.1371/journal.pone.0284785

**Published:** 2023-05-24

**Authors:** Victoria E. Gibbon, Loretta Feris, Joscha Gretzinger, Kathryn Smith, Simon Hall, Nigel Penn, Tinashe E. M. Mutsvangwa, Michaela Heale, Devin A. Finaughty, Yvonne W. Karanja, Jan Esterhuyse, Daniël Kotze, Nina Barnes, Geney Gunston, Je’nine May, Johannes Krause, Caroline M. Wilkinson, Stephan Schiffels, Doreen Februarie, Sianne Alves, Judith C. Sealy

**Affiliations:** 1 Division of Clinical Anatomy and Biological Anthropology, Department of Human Biology, University of Cape Town, Cape Town, South Africa; 2 Department of Public Law, University of Cape Town, Cape Town, South Africa; 3 Max Planck Institute for the Science of Human History, Jena, Germany; 4 Max Planck Institute for Evolutionary Anthropology, Leipzig, Germany; 5 Face Lab, Liverpool John Moores University, Liverpool, United Kingdom; 6 VIZ.Lab, Department of Visual Arts, Stellenbosch University, Stellenbosch South Africa; 7 Department of Archaeology, University of Cape Town, Cape Town, South Africa; 8 Department of History, University of Cape Town, Cape Town, South Africa; 9 Division of Biomedical Engineering, Department of Human Biology, University of Cape Town, Cape Town, South Africa; 10 School of Chemistry and Forensic Science, Division of Natural Sciences, University of Kent, Canterbury, United Kingdom; 11 University of Cape Town, Cape Town, South Africa; 12 Office for Inclusivity & Change, Office of the Deputy Vice Chancellor for Transformation: University of Cape Town, Cape Town, South Africa; 13 Social Work Private Practice, Cape Town, South Africa; The Cyprus Institute, CYPRUS

## Abstract

We describe a process of restitution of nine unethically acquired human skeletons to their families, together with attempts at redress. Between 1925–1927 C.E., the skeletonised remains of nine San or Khoekhoe people, eight of them known-in-life, were removed from their graves on the farm Kruisrivier, near Sutherland in the Northern Cape Province of South Africa. They were donated to the Anatomy Department at the University of Cape Town. This was done without the knowledge or permission of their families. The donor was a medical student who removed the remains from the labourers’ cemetery on his family farm. Nearly 100 years later, the remains are being returned to their community, accompanied by a range of community-driven interdisciplinary historical, archaeological and analytical (osteobiographic, craniofacial, ancient DNA, stable isotope) studies to document, as far as possible, their lives and deaths. The restitution process began by contacting families living in the same area with the same surnames as the deceased. The restitution and redress process prioritises the descendant families’ memories, wishes and desire to understand the situation, and learn more about their ancestors. The descendant families have described the process as helping them to reconnect with their ancestors. A richer appreciation of their ancestors’ lives, gained in part from scientific analyses, culminating with reburial, is hoped to aid the descendant families and wider community in [re-]connecting with their heritage and culture, and contribute to restorative justice, reconciliation and healing while confronting a traumatic historical moment. While these nine individuals were exhumed as specimens, they will be reburied as people.

## Introduction

During the colonial era and into the first half of the twentieth century, anatomy departments and museums collected human skeletal remains for museological display and scientific study. One major goal was to document human anatomical variation, thus, skeletal remains of Indigenous populations from around the world were pursued. Some skeletons were acquired unethically or illegally and used in studies of race typological science that haunt the discipline of biological anthropology today. Classification of humans by early western scientists was used to legitimise the race construct and provide a basis for the belief that some races were superior to others. Between 1850 and 1930 C.E., human remains of San and Khoekhoe people were sought by and traded between museums and universities globally [1–4]. San and Khoekhoe people were exhibited as a form of entertainment and for financial gain, although their exhibitors portrayed this activity as contributing to scientific knowledge [5]. In South Africa, as elsewhere, universities and museums have participated in studies and house collections of skeletonized human remains that were used for racial scientific analysis. There is an active dialogue today among stakeholders globally regarding ways to grapple with this troubled historical legacy and promote ethical stewardship of human remains in the 21^st^ century [1–14].

**Fig 1:**
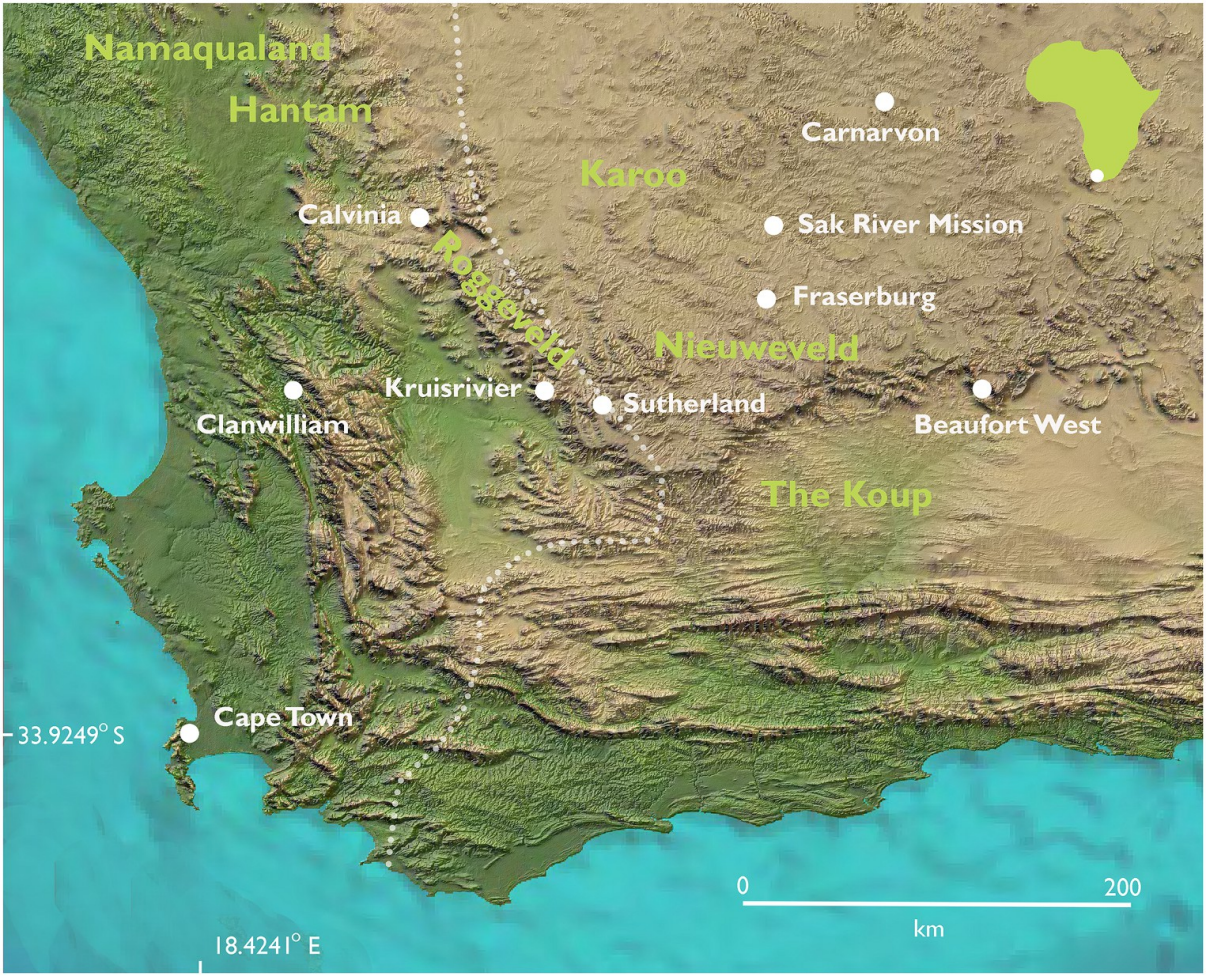
Map showing places mentioned in the text. Dotted line indicates approximate eastern boundary of the winter rainfall zone (>66% of mean annual precipitation falling between Apr–Sept), following Chase & Meadows [18].

The Human Skeletal Repository in the Department of Human Biology at the University of Cape Town (UCT) was established in 1913, when medical education and the teaching of human anatomy were formally established [14–16]. In 2017, influenced by a human remains management and repatriation (the return of human remains to their country of origin) symposium organised by the International Council of Museums and held at Iziko Museums in Cape Town, an audit of the UCT Human Skeletal Repository was conducted to identify unethically acquired skeletons. This audit identified nine individuals brought to UCT by a single donor. Most had recorded names and were known-in-life. Their skeletons had been exhumed in the 1920s from a graveyard on the farm Kruisrivier, near the town of Sutherland in the Northern Cape Province, South Africa (Fig 1). After this audit, a moratorium (a temporary prohibition of an activity) was immediately placed on access to these skeletons and their associated records. A process of restitution was initiated, led by UCT with a public participation advisor. Restitution is the restoration of something lost or stolen to its proper owner, here the return of human remains to their community of origin. This is a return of human remains or associated objects and documentation that occurs formally according to a claim for return and restoration, either based on ownership or unethical or illegal acquisition. It is a restoration of an original loss and could involve repatriation or return to the origin country.–Republic of South Africa Department of Sports, Arts and Culture 2021 [17].

In this paper we describe the process of confronting and attempting to redress this legacy, at the core being the issue of historical identity. Critically, the process began by contacting the descendants of the named individuals, who still live in Sutherland. The restitution process prioritises the descendant families’ memories, wishes and desire to understand the situation and their ancestors. Their memories link the past with the present. Below, we briefly outline the historical context that led to these graves being robbed and the skeletons brought to the University. Nearly a hundred years later, their skeletons are being returned to their community, where the reburial and its ritual salve will provide historical closure. This process is accompanied by a range of community-driven studies to document, as far as possible, their lives and deaths. We hope that this body of knowledge will help to provide understanding, closure, restorative justice and family reconnection to history and culture, all of which can bring about healing, redress and a return to dignity [19]. While these nine individuals were exhumed as specimens, they will be reburied as people.

### Background

The skeletonized remains of nine people were donated to UCT by Carel Gert Coetzee, whose family owned the farm Kruisrivier, near Sutherland (Fig 1). Biographical information on the exhumed individuals was provided at the time of donation, based mostly on knowledge passed down from the donor’s father and grandfather who had known these individuals in life. The Sutherland Nine were removed from their graves and brought to UCT over a three-year period from 1925 to 1927. Eight of these people lived and worked on Kruisrivier farm and after their deaths, seven were buried there while one was buried in nearby mountains. The ninth individual was an unnamed adult said to have been buried 40 years earlier near Sutherland although the precise burial location was unrecorded. This study has shown by means of radiocarbon dating that he died approximately 700 years ago (Table 1). There is no evidence that this individual was directly ancestral to others in the group, or to contemporary inhabitants of Sutherland, but his remains came from the same donor, presumably for the same purpose as the other eight individuals described here. He is therefore included as part of the group.

**Table 1:** Summary of biological and osteobiographic data.

Name	Sex	Age (yrs)	Stature (m)	MtDNA Haplo.	Y-Haplo.	MtDNA Contam.	X- Contam.	Pathology	Trauma	Notes
Igue We (UCT 31)	Male	30–50	-	L0d1b2b	A0-T-L1085	0.00–0.02	*	Moderate tooth wear, without dental disease.	Perimortem cranial trauma.	Only 4 bones present. No mandible. Dated to 709 ± 23 BP (MAMS-42111). For details see S4.1
G!ae (UCT 52)	Male	4–6	-	L0d2a1a	*	0.09–0.13	*	Evidence of childhood stress (5 HL)	None	None
Saa (UCT 51)	Female	6–8	-	L0d2c2	-	0.00–0.02	-	Evidence of childhood stress (3 LEH and 3 HL).	None	None
Cornelius Abraham (UCT 44)	Male	30–45	1.62	L0d1a1a1	E1b1a1a1d1	0.00–0.01	0.095 ± 0.015	1 dental abscess; pronounced overbite; extra tooth; malocclusion, TMJD, childhood stress (4 LEH), osteoarthritis; evidence of childhood stress.	None	Copper staining.
Jannetje (UCT54)	Female	45–60	1.45	L0d2a1a	-	0.00–0.01	-	Squatting facets; osteoporosis; osteoarthritis; platybasia; basilar invagination; biparietal thinning; favoured left side while chewing; 6 dental abscesses; alveolar bone resorption; dental calculus; TMJD.	None	Copper bangles and copper staining.
Klaas Stuurman (UCT 50)	Male	40–60	1.44	L0d2a1a	A1b1b2a	0.00–0.02	0.008 ± 0.003	Squatting facets; heavy tooth wear; early osteoarthritis.	Cranial trauma: antemortem, healed blunt force; perimortem sharp-blunt force.	Copper staining. No mandible.
Saartje (UCT 43)	Female	30–45	1.38	L0d2a1a	-	0.00–0.02	-	Heavy tooth wear; squatting facets; 4 dental abscesses. 2 HL.	None	Copper staining. Cranial damage after death.
Totje (UCT 45)	Male	25–35	1.51	L0d2a1	*	*	*	Activity markers on ribs and vertebrae.	Antemortem metacarpal fracture.	No cranium or mandible.
Voetje (UCT 29)	Male	50+	1.54	L0d1b2b2c	A	0.00–0.02	*	Benign metopic ridge; squatting facets; osteoarthritis; severe joint wear knee; pronounced muscle markings; edentulous; TMJD; widespread active dental infection.	Antemortem, healed fractures to nose, zygomas and ribs.	No mandible.

Yrs = years; m = meters; mtDNA = mitochondrial DNA; Haplo. = haplogroup; Contam. = contamination; LEH = linear enamel hypoplasia; TMJD = temporomandibular joint disease; HL = Harris lines

Most of the adults were identified by first names (Cornelius, Klaas, Saartje, Jannetje, Voetje, Totje); for two, surnames were also specified: Cornelius Abraham and Klaas Stuurman. There were three unnamed individuals, who were renamed as part of the process by the National San Council in collaboration with the descendant families. The younger boy child has been named G!ae, which translates to “springbok”—an animal symbolising the pride the San have in their culture and future prosperity. The springbok is also an animal characteristic of the Northern Cape Province. The older girl child has been named Saa, which translates to “eland”, a sacred and spiritual animal in the San culture. The adult mentioned above has been named Igue We, meaning “blessing”, to symbolize acceptance and blessing by the San ancestors for his reburial.

For some individuals, records associated with the accession of the skeletons into the UCT Human Skeletal Repository give approximate dates of death (*e*.*g*. “40 years ago”), racial classifications, and information on personal history and/or biology. In addition, there was information on the styles of some graves. Klaas and Saartje were said to be husband and wife, and the two children reportedly buried between them to be their children or possibly grandchildren. It was stated that Klaas died “48 years ago”, aged 40–50 years and Saartje was 60–70 years-at-death. Cause-of-death was stated for two individuals: Totje was said to have died of tetanus and Klaas to have been murdered. Cornelius reportedly had a brother still living on the farm at the time of donation. The donor knew Voetje and after his death, had buried him in the mountains on the farm. Two individuals (Klaas and Saartje), as well as the parents of Jannetje, had reportedly been free-living San who were captured and taken to the farm by the donor’s great-grandfather. This occurred almost certainly in the notorious commando raids in which many San people were killed or forced into labour [20, 21]. The notes specifically state that Klaas was captured between Carnarvon and Sutherland (see Fig 1). Three graves are described: Cornelius’s was five feet deep with stones on each side, and [stone] slabs over it; G!ae’s was six feet deep but otherwise similar. Saartje’s grave was four feet deep, with the body laid in a side niche off the main grave shaft. Large stones were placed in the grave, blocking the niche entrance.

## Results

This study revealed that the donor Coetzee was a registered medical student at UCT between 1925 and 1931 (S1 Fig). Prof MR Drennan, Head of the Department of Anatomy at that time and the person who accessioned the Sutherland Nine, would have been one of his lecturers. Drennan may have directly solicited San and/or Khoekhoe remains, in keeping with the rationale of skeleton collecting and the racist paradigm of the typological approach in biological anthropology at the time [4, 6, 22]. Specific ‘racial information’ was recorded as part of the accessioning of the skeletons, so this was clearly considered important.

Restitution began with a public participation process that was conducted following the Aitken [23] community engagement model coupled with the Vermillion Accord [24] as described in the SI. It was initiated by contacting families in Sutherland with the surnames Abraham and Stuurman, and their relatives. The consultation process was informed by the Promotion of Administration Justice (Act 3 of 2000) and the South African National Heritage Resources Act (1999), which protects historical burial grounds and graves. Whilst the legal requirement (S1 Table in S1 File) was only to place an advertisement in the local newspaper, UCT elected to engage directly with the community, starting with informal consultation and moving on to a process of identifying relevant stakeholders. In October 2018, a delegation from UCT met the direct descendants, Sutherland community leaders, members of the farming forum and others from the broader community in a public meeting held in Sutherland. In addition, notices were posted in local newspapers and a press conference was held in Cape Town to announce the situation, reaching all major national media.

At the initial meeting between UCT and the Sutherland families, the circumstances of the skeletons of these nine individuals at UCT was revealed. The University made an unreserved apology to the community. The families were shocked and dismayed; the pain of the situation was palpable, and there were many questions about why and how this could have happened. The families were informed of the moratorium and it was explained that all decisions from that point onward were in their hands. It was immediately clear that reburial was the priority, however, the families wanted to learn as much as possible about the situation and their relatives. They requested that the Sutherland Nine be comprehensively studied. This was the beginning of a journey guided by the Sutherland families. A multidisciplinary collaborative team was assembled to investigate historical context, archaeology, osteobiographies (reconstruction of an individual and their life history through analyses of their skeletal remains [25]) digital facial reconstruction and depiction, genetic and stable isotope analyses. Informed consent for these investigations was requested from the families and granted (outlined in S1 File, S2). To ensure that the families felt comfortable with the ethics and informed consent process, this was discussed over several meetings. To prevent undue influence by scientists, a member of the scientific team was present at some but not all meetings. The process of academic publication and benefits of scholarly outputs were discussed with the families, along with possible risks associated with being identified as descendants. Family members wrote in their own words what research they wanted done and why, along with their restrictions on data use. Informed consent was obtained for all aspects of this study, as well as permission from the University of Cape Town’s Human Research Ethics Committee for analysis of tissue samples (HREC# 715/2017). A legally binding materials transfer agreement was established between UCT and the Max Planck Institute for the Science of Human History in Jena, Germany on the use and storage of aDNA and molecular data [26]. For this publication, there was discussion about the timing of submission and whether the families would be co-authors or acknowledged. They requested acknowledgement rather than co-authorship.

### Historical and archaeological context

The rural town of Sutherland, established in 1855, is in the Western Roggeveld mountains of the Karoo (32.39S 20.66E0) South Africa (Fig 1). These three place names, one English, one Dutch and one San and/or Khoekhoe, reflect a complex frontier history of Indigenous dispossession from the 18th century onwards (see S1 File, S3). The people exhumed from the Kruisrivier cemetery in the 1920s were victims of this dispossession.

For San and Khoekoe people to live in a harsh, dry region like the Roggeveld required intimate knowledge of local ecological and climatic patterns. As herders they managed their flocks through seasonal transhumance. Colonial encroachment into these regions continued from the 18^th^ throughout the 19^th^ century, despite significant resistance [20, 21, 27, 28]. Competition for land and resources led to farmers raising commandos (informal, mounted, quasi-military units)to raid for livestock, and to slaughter San and Khoekhoe peoples [20, 21, 27, 28]. In these raids, San men were almost always killed, while women and children might be taken captive and forced into farm labour. Bank [28], summarising accounts given by /Xam prisoners to Bleek and Lloyd, described the fear and anger of dispossession on the northern colonial frontier in the second half of the 19^th^ century. The Roggeveld is part of one of the most blood-stained colonial frontiers in South African history [20, 21, 27, 28]. Those captured were forced into a life of subjugated labour on farms with little protection from the power and abuse of farmers [20, 21, 29]. The colonial history of dispossession, destruction, and murder in this region (and elsewhere) led to the loss of many aspects of San and Khoekhoe lifeways, but not to their complete destruction [30].

The archival records state that seven of the nine individuals described in this paper were removed from graves on Kruisrivier farm. As part of the research undertaken for this study, an archaeological survey of the farm was conducted, and the labourers’ cemetery was located (S2 Fig) and mapped (S3 Fig) 3D model link here. A minimum of four, and probably five disturbed graves were identified by their packed stone coverings having been dismantled and the stones scattered. Four of the disturbed graves had head- and footstones, which correspond to Christian burial practice in the region. One burial lacked clear head- and footstones but had been covered with a sub-circular cairn of rocks. This grave style can be assigned to a San and/or Khoekhoe identity [31–34]. It was not possible to link individuals with specific graves, although an area of general disturbance between two graves might be where the children were buried, between Klaas and Saartje (S3 Fig). Milky quartz pebbles, probably imported from the Fish River gravels a kilometre away, suggest some continuity of San and Khoekhoe cultural practice: quartz, quartz crystals, and white seashells are recorded from precolonial graves and were used in San and Khoekhoe rituals [35: pp. 53–65]. A key observation is that the disturbed graves are surrounded by many that were undisturbed. Considering the specific biographical details supplied at the time of donation, particular individuals were targeted, probably as “specimens” of “racial types” based on information known by the donor and his father.

### Osteobiographies

For each of the nine individuals an osteobiography was developed, assessing age-at-death, sex, stature, markers of habitual activities and evidence of disease and injury that inform us about how they lived and died. Methods used are described in SI S4 and the results obtained summarised in Table 1. Injury was categorised in terms of timing relative to death. All results should be understood in the temporal and spatial context in which these people lived, and how these circumstances would have affected health.

Five of the seven adults were male and two were female, consistent with the DNA results (see below). Ages-at-death range from 25 to more than 60 years. All had relatively short stature (1.38–1.54 m, apart from Cornelius at 1.62 m). All five adult men showed signs of osteoarthritis and activity markers (enthesopathy) resulting from heavy physical labour and active lives.

Nearly all the adults showed signs of heavy tooth wear and dental infections, and abscesses were noted in 83% (5/6) of adult Sutherland individuals with crania or mandibles preserved. Poor dental health was likely exacerbated by life on the farms with a limited range of foods available, largely carbohydrates. Jannetje, Saartje and Voetje all had extensive dental abscessing that, towards the ends of their lives, would have made eating difficult or impossible and may have led to systemic infections that contributed to or caused their deaths. Common dental problems such as cavities and abscesses sometimes resulted in complications such as cavernous sinus thrombosis and Ludwig’s angina [36], that contributed to high rates of mortality in the past.

Two individuals were children: G!ae, a boy 4–6 years-of-age, and Saa, a girl 6–8 years-of-age. Sex was determined from DNA, as explained below. G!ae, Saa, Saartje and Cornelius showed signs of childhood stress indicated by linear enamel hypoplasias and Harris lines, *i*.*e*. episodic interruptions in the formation of tooth enamel and radiopaque transverse lines detectable in the metaphyses of long bones as an indicator of growth arrest. These are due to physiological stress and are typically caused by infection, disease or malnutrition. G!ae showed five Harris lines indicating stress periods between 1–4 years. Saa showed at least four episodes of stress from 2–4 years-of-age and three Harris lines corresponding to stress at ages one year and 4–7 years. Saartje had Harris lines in her tibiae, showing stress at age one year and 13–14 years. Cornelius had enamel hypoplasias on his first molars, indicating stress at approximately 1–2 years-of-age. Individuals with childhood stress tend to have lower life expectancy [37] Stress during the first years of life may be linked with weaning—an often-traumatic period that can result in caloric deficiency, in turn increasing susceptibility to infection or illness [37–39].

There are signs of injury on four out of the five adult men: fractured noses, fingers, ribs, cheeks and cranial vaults. Most injuries had healed antemortem, but Klaas and Igue We both show evidence of severe cranial trauma that caused their deaths (S4 Fig). Voetje had old injuries to his left foot and knee and probably walked with a limp–perhaps the origin of his name, which translates as ‘small foot’. He also had a congenital condition resulting in premature fusion of his cranial plates (craniosynostosis and benign metopic ridge) [40, 41]. Jannetje’s cranium showed evidence of pathology (S6 Fig): she had biparietal thinning, associated with postmenopausal osteoporosis [42]. Additionally, platybasia and basilar invagination were found, the latter of which is known to be debilitating with neurological and/or musculoskeletal complications, suggesting that she may have required assistance [43, 44].

Squatting facets were found on the distal tibiae and tali of four adults (Jannetje, Klaas, Saartje, Voetje), evidence of a habitual resting posture with deeply flexed knees and ankles, a position culturally important among southern African hunter-gatherers and herders [45, 46].

Cornelius, Jannetje and Saartje had green (copper) staining on one or more locations on their skeletons (S1 File, S4), in patterns consistent with copper ornaments worn on the body and included in the burial. These are preserved only with Jannetje, in the form of two copper bangles that produced marked copper staining on her right arm and diffuse staining on the left-hand side of her face, pointing to a side burial position with her head pillowed on her arms. Saartje probably wore a copper ornament attached to her hair in the midline above her forehead, and two copper bangles on her lower right leg. A substantial area of copper staining on the right-hand side of Cornelius’s face, extending from the cheek to lower jaw, indicates the presence of a sizable copper object. Tentatively, the staining might have been produced by a decorative ear-plate such as that shown in the illustration by Burchell [47, reproduced in S5 Fig], who reported that these varied from two to five inches in length. San people along the Riet River also wore copper ear-plates [48].

### Craniofacial reconstruction and depiction process

Facial reconstruction and depiction were completed for the eight individuals whose crania were preserved. These were based on computerised tomography scans in a complete digital workflow (see S1 File, S5). Visualising the face is an important forensic tool and has been used in archaeological cases, enabling empathy, and the potential for an emotional connection with the person represented [49–51].

Depiction involves the conversion of reconstructed facial shape to a plausible and relatable face through the addition of surface details such as skin tone, texture, hair and wrinkles that cannot confidently be predicted from the skull alone, but may be inferred from available contextual information [52, 53: pp.57]. Clothing and hairstyles were selected from a visual database that included nineteenth century [54] and modern photographic portraits of San and Khoekhoe people from the western and northern Cape regions sourced online [for example Hammond in 50], and non-photographic visual sources [47, 55, 56].

**Fig 2:**
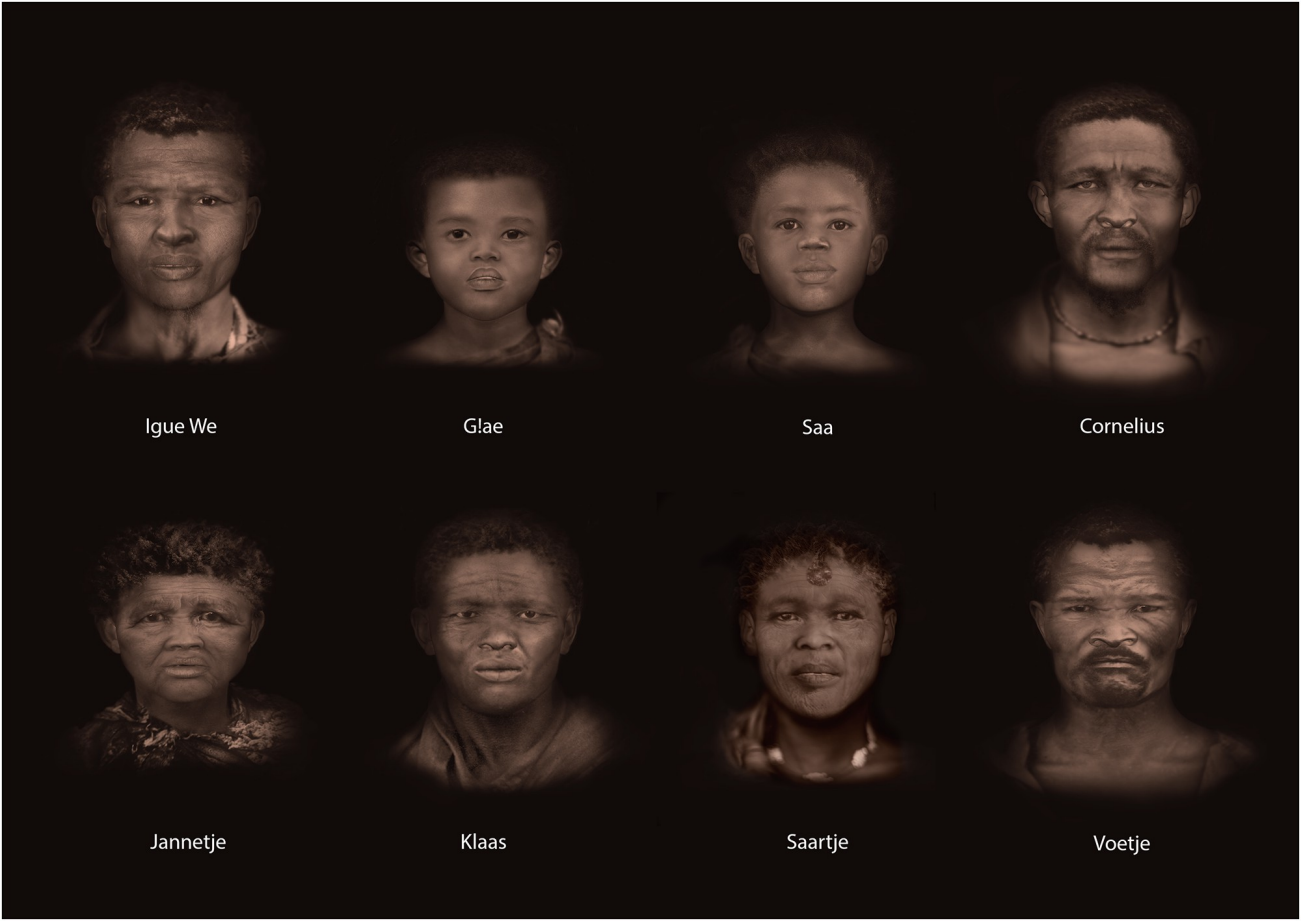
Facial reconstructions presented as 2.5D stills from frontal screenshots of 3D models. Photographic textures applied using digital montage techniques.

Decisions were informed by the osteobiographical, biological and archaeological data of each individual. Ambiguity around certain details was visually stressed through techniques such as subtle blurring, which may also imply motion: a facial depiction can only reasonably estimate appearance at the time of death. A subtle sepia tone was applied to avoid assuming colour where it cannot be known (see Fig 2), whilst adding visual warmth and locating these individuals in the visual style of nineteenth and early twentieth century photography, when all except Igue We lived.

Upon presentation, family members expressed amazement at the realism of the faces, commenting that they did not expect them to look like ‘someone they could meet in the street’, and excitedly pointing out which bore a resemblance to living relatives. Facial gestalts from strength to sadness were remarked upon–‘hy lyk nogal kwaai’ (he looks quite fierce); ‘daar is iets in haar gesig wat baie swaar is’ (there is something in her face that is very ‘heavy’, implying suffering or hardship)–despite the faces being presented without obvious facial expressions. Ultimately, they agreed that ‘the images bring everything together’ and ‘the faces provide the way into the bigger story,’ echoing the philosopher E Levinas’s [57, 58] insistence that as humans, we cannot help but seek interpersonal connection through the face. The process of facial depiction thus contributed positively to the restoration of individual personhood for unknown or past people.

### Genetic analyses

Ancient human DNA was recovered from the teeth of all nine individuals using established protocols (see S1 File, S6). The sequence data showed evidence of authentic ancient human endogenous DNA ranging between 0.02% and 14.41% although DNA-library contamination, caused by people handling the skeletons from the collection to the lab, was estimated to be present in several samples (S2 Table in S1 File). Molecular sex was determined using the genome-wide sequencing data; six individuals were males and three were females (Table 1 and S2 Table in S1 File). Kin relationships among the nine were assessed by comparing pairwise mismatch rates of genome-wide analysed polymorphic sites (single nucleotide polymorphisms) between all pairs of individuals [59]. Inferred kin relationships were found between three pairs of individuals: a 2^nd^ degree relationship (*i*.*e*. half siblings or double cousins) was identified between Saartje and Klaas and a 3^rd^ degree relationship (*e*.*g*. first cousins) between Jannetje and both Klaas and Saartje. It was confirmed that Klaas and Saartjie were not the parents of the two children.

**Fig 3:**
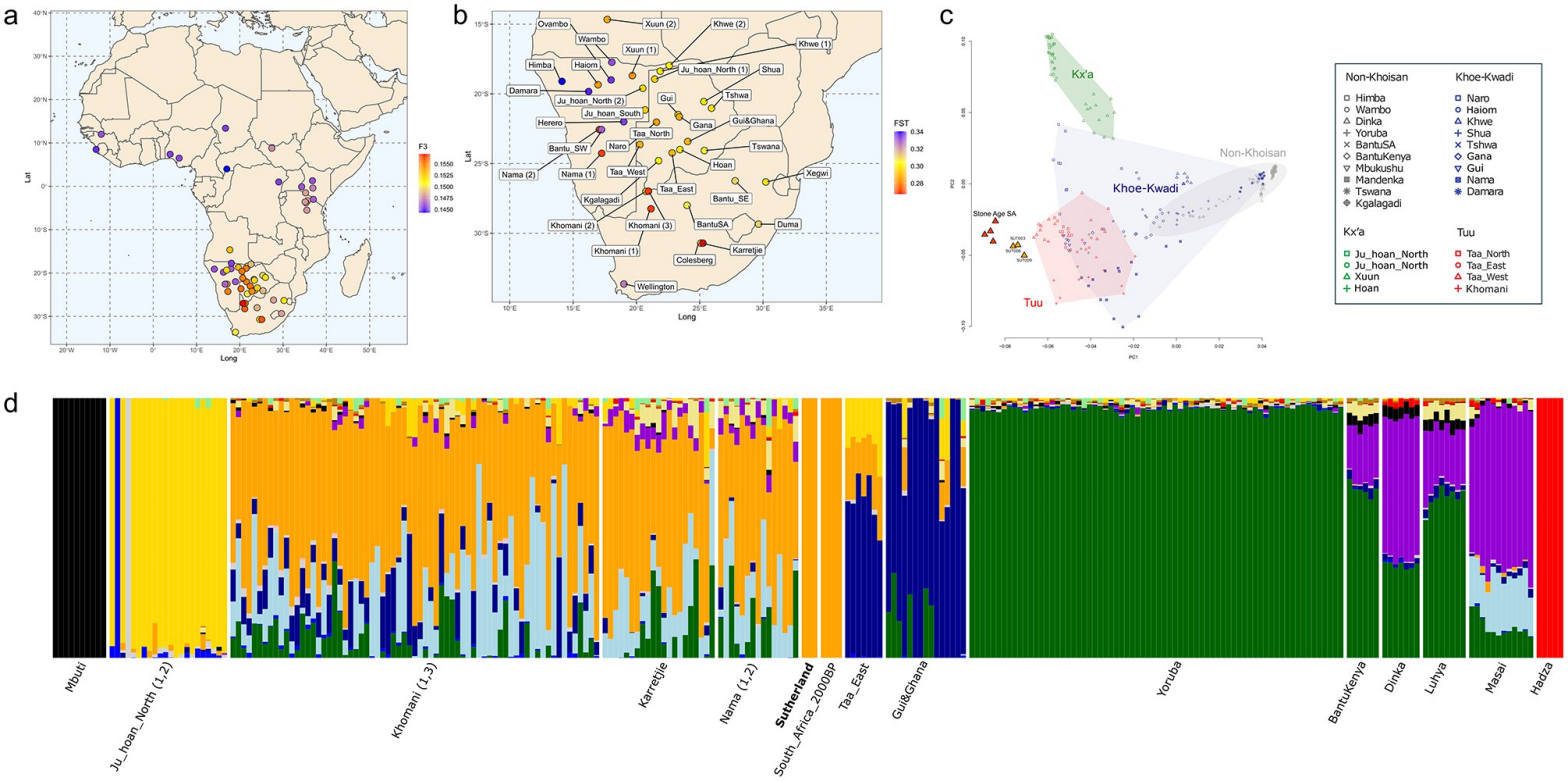
Summary of genetic results. A) Outgroup f_3_ statistics calculated between the three Sutherland individuals grouped together and present-day African populations. B) F_ST_ distances calculated between the three Sutherland individuals grouped together and present-day southern African populations. C) PCA of present-day individuals from 27 African populations. Symbols and colours denote the individual’s population and linguistic affiliation. Previously published Stone Age samples from South Africa as well as the Sutherland individuals are projected onto first and second axes of genetic variation and plotted as red and orange triangles, respectively. D) Unsupervised ADMIXTURE analysis of an extended dataset including non-South African individuals at *K* = 15.

The genomes of Saartje, Klaas, and Jannetje were well preserved and met the quality requirements (sufficient autosomal coverage and low contamination) for comparisons with previously published present-day and ancient human genome-wide data (S1 File, S6). A principal components analysis [60] was performed, as well as model-based clustering [61], ancestry modelling [62] and allele frequency correlations with other published ancient and modern populations (so-called *f*-statistics [63] (Fig 3, S26–S31 Figs). Across all analyses, these three Sutherland individuals show the greatest genetic similarity with Kx’a and Tuu-speaking groups from the northwestern and southeastern Kalahari, such as the Ju/’hoansi, Taa, ǂKhomani, Karretjie, /Gui and Naro, which are the populations with the greatest degree of Indigenous South African ancestry and the smallest non-San and/or Khoe admixture [64]. In the three genomes that allowed these analyses, we did not detect any admixture from groups in present-day Europe and Asia. To maximise the resolution of population genetic affinities, the three Sutherland genomes were grouped together and the F_ST_ was calculated between them and the modern reference panel. The Sutherland individuals exhibit the lowest F_ST_, and therefore have the greatest genetic affinity to southeastern Kalahari populations such as the ǂKhomani, Karretjie and Nama peoples.

Regarding the non-San or Khoekhoe admixture within the Sutherland population, formal ancestry modelling using qpAdm was performed following the approach of Skoglund and colleagues [66]. As with other San and/or Khoekhoe groups, the Sutherland population can be modelled using two ancestry components: one related to Later Stone Age hunter-gatherers (South_Africa_2000BP.SG) as a proxy for Indigenous South African ancestry [65, 66], and one related to a Pastoral Neolithic East African individual (named Tanzania_Luxmanda_3100BP) as a proxy for incoming eastern African pastoralist ancestry [67]. The Sutherland population showed 11.2% East African-related ancestry, which is comparable to other less admixed populations like the ǂKhomani (Khomani_San.DG: 8.7%), Ju/’hoansi (Ju_hoan_North: 11.9%), /Gui (Gui: 16.5%) or Taa-speaking groups (Taa_West: 12.4%, Taa_East: 15.5%).

### Stable isotope analyses

The stable isotope composition of body tissues provides information about the environments in which people lived, and the foods consumed at the time of tissue formation. Analysis of δ^13^C and δ^15^N (see SI S7 for definition) in serial samples of dentine from first molars of Klaas and Igue We, and first incisors from Cornelius, Saartje and Jannetje enabled us to track their diets from soon after birth until the age of approximately 9 years [68, 69]. Additional molars extend this timeline into the teenage years (for Klaas, based on his second molar) or early twenties (for Saartje, based on her third molar). Bone remodels throughout life and records diet over a longer period. More rapidly remodelling bones such as ribs probably reflect mainly the diet consumed during adulthood.

**Fig 4:**
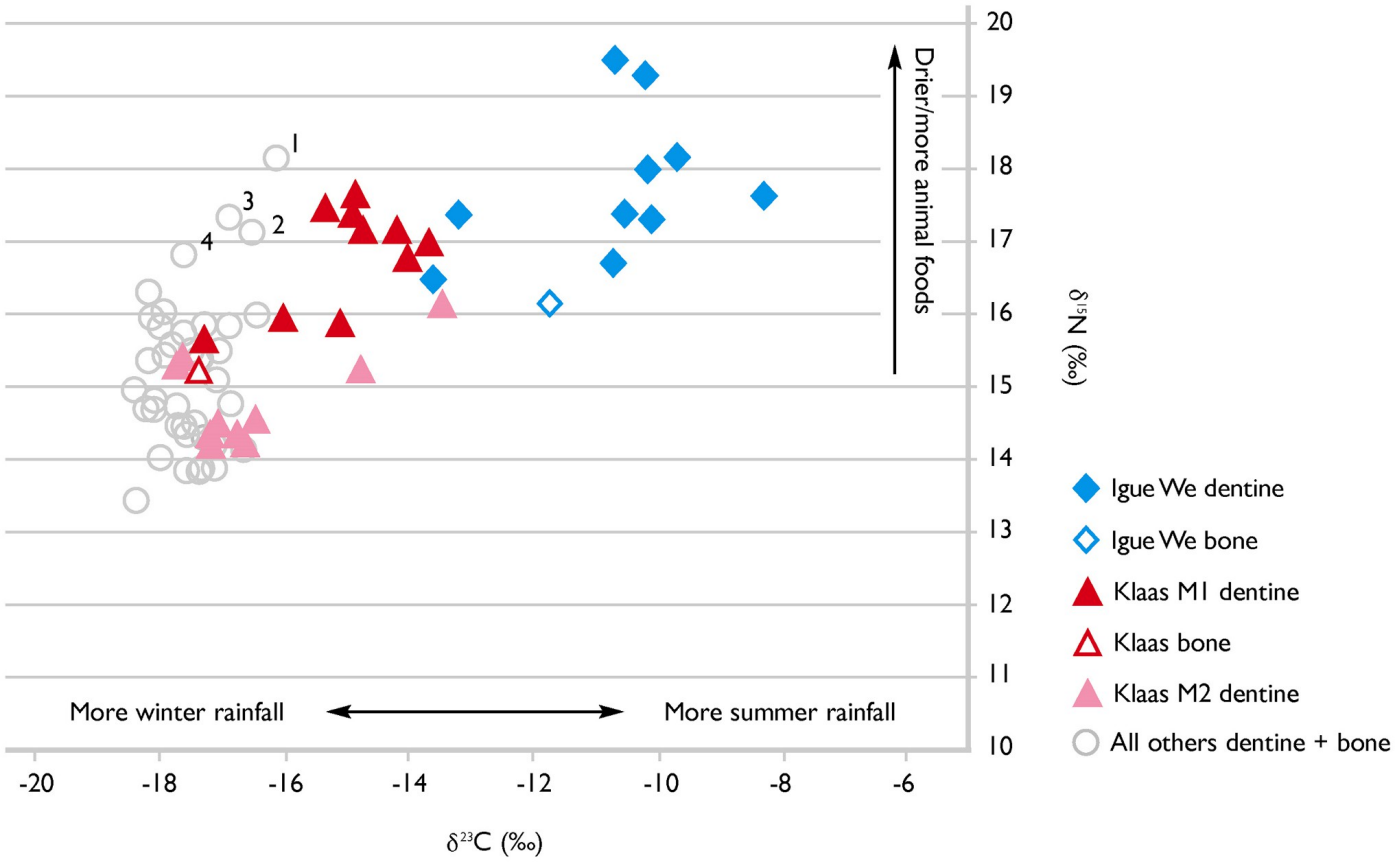
δ^15^N plotted against δ^13^C for bone and dentine samples analysed in this study. Points labelled 1 and 2 represent dentine from Cornelius’s first incisor crown (1 is the occlusal half of the crown, 2 is the half closest to the cemento-enamel junction) and point 3 is the root tip of his mandibular second molar. Point 4 indicates dentine from the occlusal half of Saartje’s first incisor crown.

The results are provided in S2 Dataset and plotted in Fig 4 and S34 Fig. Igue We lies at the high δ^15^N / positive δ^13^C end of the distribution seen here. This may result from slight differences in climate and environment approximately 700 years ago compared with the 19th/early 20th century, and/or it may indicate residence in a drier area with more summer rainfall. Substantial variation within the first molar of Igue We (ranges of 5.3‰ in δ^13^C and 2.9‰ in δ^15^N) indicates movement across the landscape during the period of tooth formation. Values for bone and dentine of the other individuals except Klaas cluster towards the lower left of the plot (δ^13^C < -16.0‰, δ^15^N < 16.5‰, n = 39), apart from four points labelled 1–4 in Fig 4, to be discussed further below. Values for dentine from Klaas’s first and second molars were varied. Those for tissue formed up to the age of approximately one year fall within the cluster just mentioned. Dentine formed between ages 1–2 years has intermediate values, and that formed from 3–10 years has δ^13^C > -16.0‰ and δ^15^N > 16.5‰, outside the range of the other Sutherland individuals. From the age of about 10 years onwards, values shift towards more negative δ^13^C and lower δ^15^N, and from the ages of 12–15 once again fall within the cluster of the other seven historic individuals, as do those for his bone. Leaving infancy aside, it is inferred that from the ages of approximately 3–10 years Klaas consumed a different diet and/or lived in a different environment. This is consistent with the archival information recorded at the time of donation, which stated that he had been part of an independent, free-living group, but was captured in the area between Sutherland and Carnarvon (see Fig 1) and taken to work on the Kruisrivier farm. From the isotope values reported here, we now know that he was about 10 years old when this happened. The later-forming dentine of his second molar and his bone reflect his post-capture diet and environment, shared with others on the farm but different from those of his childhood.

In Fig 4, four data points from other historic Kruisrivier individuals also have δ^15^N > 16.5‰. Three are from Cornelius: two are dentine from his first incisor crown (forms ca. 0–4 years-of-age) and the third is the root tip of his mandibular second molar (forms ca 15 years-of-age). The fourth point is dentine from the occlusal half of Saartje’s first incisor crown (forms ca. 0–2 years). Higher δ^15^N values in dentine laid down during the first few years of life may reflect the consumption of breast milk [70] although we do not see this pattern in Igue We, Klaas or Jannetje (for whom only half the tooth crown was available for analysis due to heavy tooth-wear). They may also reflect residential/dietary shifts. We note that Saartje was reportedly also a member of a free-living family and was captured and brought to the farm, as were Jannetje’s parents. The more negative δ^13^C and in most cases lower δ^15^N values in Saartje and Jannetje’s first incisors are inconsistent with their having lived during early childhood in an area with more summer rainfall, as Klaas did. Values for Saartje’s first and second decades of life, reflected in her first incisor and the root of her third molar, are very similar.

For the remaining individuals, the clustering in δ^13^C and δ^15^N values, and the similarity between values for bone and dentine, are consistent with having lived close to Sutherland most or all their lives. The isotopic results are discussed in more detail in the S1 File, S7.

## Discussion and conclusions

Although the human skeleton may be regarded as inanimate, dried remains of a once-living being, during life the skeleton forms the foundation of our social and personal interactions with our surroundings. Thus, it preserves a unique record of life experiences. The interdisciplinary research completed on this small group of people tells us about their lives and provides a picture of life for labourers on the northern frontier of the Cape Colony (now part of South Africa) in the nineteenth century C.E. Their DNA confirms they were all descended from San and Khoekhoe genetic lineages. Life was physically hard and violent. Common conditions like toothache and infections may have caused death. Children were abducted from their families and forced to grow up without their biological parents. In other parts of the world the destruction of a people’s way of life, as described for the Roggeveld, has been classified as ‘cultural genocide’ [71], and histories like these have far reaching impacts on descendant communities for generations. Despite the hardship and challenges for people living on this farm and others like it, these people resisted complete assimilation into the farm-owners’ way of life. They retained some of their own cultural practices, as shown by their habitual use of a resting posture with deeply flexed knees and ankles [45, 46], the styles of their graves and aspects of burial practice (niche graves, inclusion of quartz pebbles), and the wearing of traditional body ornamentation. This window into the past reveals hardworking people with perseverance and resolve.

The presence of children in this group is not unexpected: in the era before the widespread use of antibiotics child morbidity and mortality were devastatingly common. Childhood (post-first year through to puberty) is more than a biological stage of life; it is a period in which a child’s experience is shaped by social and cultural events and exposure [37]. Globally and through time children in pre-modern civilisations had high mortality rates. Approximately 27% of infants did not survive their first year, and nearly 50% didn’t survive to puberty [72]. Children who have underlying congenital problems or experience substantial biological stress in early childhood are particularly susceptible to disease and infection, and violence and accidents may result in early mortality [72]. Most diseases do not persist long enough to leave their marks on the skeleton.

The archival records dating from the 1920s state that both Klaas and Saartje were captured by the donor’s great-grandfather. They suggest the two were husband and wife, and were the parents of Saa and possibly G!ae. DNA evidence shows that Klaas and Saartje were close relatives (half siblings or double cousins), so they were probably not husband and wife. Neither was a biological parent of either of the children, although they may have been their primary caregivers on the farm. This sort of adoption must have taken place on farms with children captured and forced into labour. Stable isotope values for Klaas show that he spent his early childhood in an area with more summer rainfall and moved to Sutherland at around 10 years of age. This is consistent with the archival record of his capture between Sutherland and Carnarvon. Despite the close genetic relationship between Klaas and Saartje they did not live in the same area before moving to Kruisrivier. Both Klaas and Igue We show substantial isotopic variation during early life, consistent with the mobile lifestyles documented for foraging communities in the dry interior of southern Africa, where groups ranged over large territories of up to 2500 km^2^ annually [summarised in [73] Table 4.1]. Individuals or families might also spend time with relatives or friends in other groups, thus increasing their geographical range.

Other points on which the analytical results contradict the archival records are that Igue We was stated to have been buried 40 years previously, but the radiocarbon date shows this to be a substantial underestimate. Osteobiographical work shows that Saartje died at a considerably younger age, probably 35–40 years, rather than 60–70 years as reported in the accession register. These records largely recount information supplied by the donor’s father and grandfather, and therefore lapses of memory and inaccuracies are not surprising. In addition, they represent the farm owner’s perspective of these people as farm labourers. It is noteworthy that the names recorded are of European origin, nearly all are Dutch, and some were common in the Coetzee family, such as Klaas and Cornelius. These names would have been used on the farm, but at least some individuals (especially those who began life as part of free-living groups) would have had names in their own language, which were probably known and used by those closest to them, but have gone unrecorded.

The remains of the Sutherland Nine reminded UCT of its complicity in South Africa’s reprehensible colonial past and the violence committed against the country’s Indigenous communities. The discovery also provided an opportunity for atonement, redress and ultimately for learning and healing. Examination of the remains of these individuals stands as a strong moment of agency by the descendant families who wanted to know more about their ancestors. It also revealed a very rich picture of these people’s lives. The combination of interdisciplinary information has allowed for a more holistic view and a bringing to life of these people once again in the 21^st^ century. This is exemplified in the facial reconstructions and depictions, whereby the recreated face was a powerful medium to enable the confrontation of forgotten histories, fostering mutual recognition and forging connections to the past for the future, providing a tangible perspective on the past that is more immediately relatable than the scientific story alone. For the families and community, seeing the faces of their ancestors was a poignant and emotionally powerful moment.

**Fig 5:**
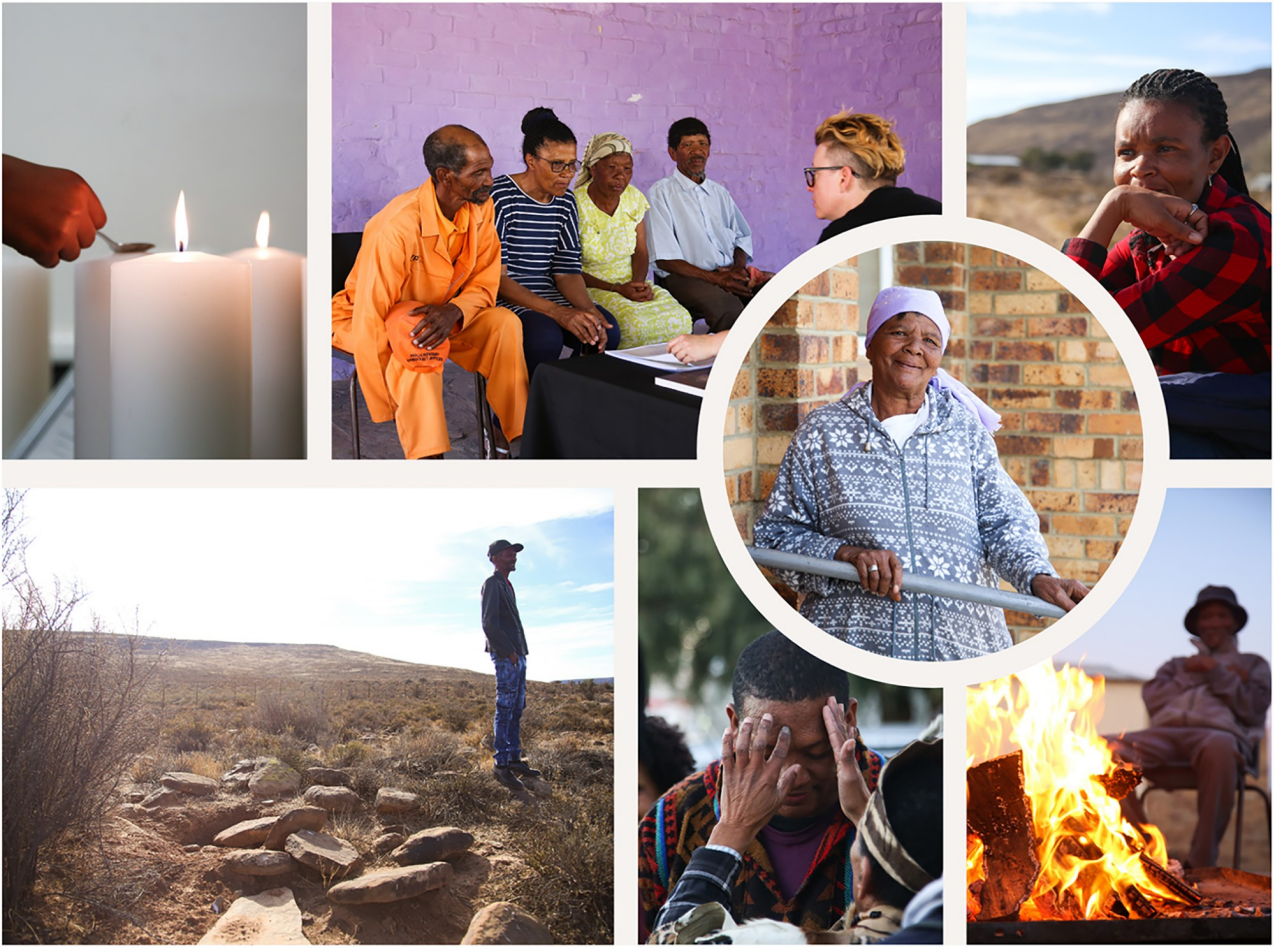
Top left: Candles from the blessing ceremony held at UCT on February 8, 2019; top middle: Sutherland families looking at the facial reconstructions; top right: Sutherland families visit to Kruisrivier farm and the disturbed graves; bottom left: one of the disturbed graves on Kruisrivier farm; bottom middle: traditional knowledge sharing; bottom right: traditional land cleansing; circle: A De Wee (nee Abrahams).

As expected, there were many challenges in the restitution process (see S1 File, S1). At the start, lack of a South African government policy on restitution and repatriation of human remains was a challenge. While there have been other restitution processes in South Africa, none have involved a direct descendant family or been initiated by a university. In the absence of a clear government policy, UCT had to develop its own process. This was admittedly imperfect and has been critiqued for not consulting broadly enough. Given the large number of stakeholders, it was important to ensure that the voices of the descendant families remained primary. Language differences complicated communication, and different stakeholders sometimes held conflicting views. There are debates amongst family members as to the geographic origin (and thus appropriate location for reburial) of Klaas Stuurman, which led to delays in the reburial process (still ongoing at the time of writing). Many aspects of academic practice were initially unfamiliar to non-academic stakeholders. The public participation consultant played a critical role in supporting the families in expressing their wishes and preferences. The COVID-19 pandemic affected the descendant families and the Sutherland community at large and made broad in-person consultation and meetings difficult or impossible. Whilst the process was admittedly imperfect, the Sutherland example sets a precedent in South Africa for a process of restitution in combination with community driven multidisciplinary science, thus building a fuller picture of these nine people. Research was only one aspect of this restitution process. The families were able to begin to reconnect with the remains of their ancestors, spending time with them at the university, and visiting the graveyard to see the disturbed graves (Fig 5). To include the wider Sutherland community in the restitution process, there has been traditional and scientific knowledge sharing and outreach activities in schools in the town of Sutherland. The community was able to strengthen their connection with ancestral culture and practices, rolling back a small part of historical damage.

Confronting historical colonial legacies like these places institutions in a position to attempt to redress past wrongs and restore a measure of justice. Biological anthropology and its long history in the formation of racial science and typology along with restitution is generating an important dialogue in South Africa. It has resulted in a new management strategy for the UCT Human Skeletal Repository [14]. Notably, since the completion of this process a national policy has been established in South Africa for restitution and repatriation, influenced by the Sutherland process. Its implementation is expected soon and is anticipated to transform the management of human skeletal repositories in the country. It is hoped that this policy will remove barriers and provide effective guidelines for action with regards to restitution and repatriation.

In Latin “mortui vivos docent”–the dead teach the living. The Sutherland Nine have fostered discovery, connection and reconnection between the past and the present for all involved. This restitution process affirms the UCT commitment to decolonisation and transformation in the form of redress, restoring dignity in the pursuit of closure and justice.

## Supporting information

S1 Fig Academic record of Carel Gert Coetzee, the donor, at the University of Cape Town from 1925–1931. It gives his place of residence as Kruisrivier Farm, Sutherland.
(TIF)

S2 Fig Aerial photo showing features on the Kruisriver farm complex including the location of the farm labourers’ cemetery. a and d both indicate threshing floors; b is the Kruisrivier school; c is an 18^th^ century archaeological deposit; e was a farm labourer dwelling; f is the Coetzee family cemetery; and g is the farm labourers’ cemetery.
(TIF)

S3 Fig Plan of the Kruisrivier farm labourers’ cemetery. Green dots indicate the western ends of stone cairn burials (n = 11), white dots the headstone (western) ends of headstone and footstone burials (n = 25). Green ring indicates disturbed cairn burial, white rings disturbed headstone and footstone burials.
(TIF)

S4 Fig Digital 3D models illustrating cranial trauma. Top is Klaas’s cranium, illustrating the perimortem trauma. Left anterolateral view, shows entrance wound on the anterior surface of the left maxilla inferior to the orbit and medial to the left infraorbital foramen. A radiating fracture extends through the left lacrimal, ethmoid and sphenoid bones. Top right shows the inferior view, with a radiating base-of-skull fracture through the occipital bone on the left side of the foramen magnum. The bottom images are of Igue We’s cranium illustrating the perimortem trauma. Bottom left, left superolateral view, radiating fractures from the points of impact on the right are observed dissipating through to the sagittal and coronal sutures; a portion of this energy dissipates into the left parietal bone with production of a primary radiating fracture extending posteriorly, which terminates inferior to the left eurion. Bottom right, two points of impact are observed with radiating fractures.
(TIF)

S5 Fig Tswana man wearing an ear-plate, from Burchell (1824). (TIF)

S6 Fig Digital 3D and 2D models illustrating the pathology of Jannetje’s cranium. An anterior left superolateral view (top left) and the 2D reconstructed CT images (bottom left, middle and right) show three major impressions in the cranium (white arrows) as a result of calvarial thinning. The Lodox images (top middle and right) illustrate the presence of osteoporosis as evidenced by a uniform decrease in radiodensity, suggesting extensive rarefaction of spongy bone. The white arrows in these two images further illustrate examples of thinning and rarefaction of cortical bone, observed as thin, faded or non-existent lines of radiodensity. Mid-sagittal reconstructed CT images (bottom middle and right) illustrate metrics evaluated for the diagnosis of platybasia and basilar invagination.
(TIF)

S7 Fig Process of mandible estimation for Igue We (left, showing in-filled cranial cavity), Voetje (middle) and Klaas (right). (TIF)

S8 Fig 3D cranial model of Igue We from CT data (left) and reconstructed parts (right) including realigned parietal bones and teeth lost post-mortem. (TIF)

S9 Fig Process of facial reconstruction (left, middle) and final depiction (right) for Igue We. (TIF)

S10 Fig Process of facial reconstruction (left, middle) and final depiction (right) for G!ae. (TIF)

S11 Fig Process of facial reconstruction (left, middle) and final depiction (right) for Saa. (TIF)

S12 Fig Process of facial reconstruction (left, middle) and final depiction (right) for Cornelius. (TIF)

S13 Fig Process of facial reconstruction (left, middle) and final depiction (right) for Jannetje. (TIF)

S14 Fig Process of facial reconstruction (left, middle) and final depiction (right) for Klaas. (TIF)

S15 Fig Process of facial reconstruction (left, middle) and final depiction (right) for Saartje. (TIF)

S16 Fig Process of facial reconstruction (left, middle) and final depiction (right) for Voetje. (TIF)

S17 Fig The facial depictions were digitally printed on wood panels (left) and presented to each family in an archival box (right). (TIF)

S18 Fig A bilingual (Afrikaans and English), illustrated facial reconstruction album visually illustrated the process alongside detailed biographical narratives of each individual. The panels and albums were presented at a knowledge sharing session for the families in Sutherland, October 2019.
(TIF)

S19 Fig 3D mesh surface model formation from CT images for Klaas’s cranium. (TIF)

S20 Fig Sex determination. X-chromosomal coverage vs. Y-chromosomal coverage, normalised by autosomal coverage. Error bars represent the uncertainty in the calculation of relative coverages.
(TIF)

S21 Fig Pairwise mismatch rates between all sequenced libraries. Individual pairs of interest are highlighted accordingly.
(TIF)

S22 Fig PCA and sampling locations of 40 African populations (human origins panel, 600,000 SNPs), the Sutherland and published ancient African samples are projected onto the PCs. (TIF)

S23 Fig PCA and sampling locations of seven present-day San and Khoekhoe populations (Schlebusch panel, 540,000 SNPs), the Sutherland samples are projected onto the PCs. (TIF)

S24 Fig Maximum likelihood based on the allele frequencies of 1.2 million SNPs from populations of the <1240k> panel show no evidence of asymmetrical allele sharing with non-Africans indicative of recent gene flow. (TIF)

S25 Fig ADMIXTURE results for K = (2…10,15,20) based on 21 present-day and ancient populations from the Schlebusch <H0> panel. (TIF)

S26 Fig a) Outgroup f3 scores of the Sutherland samples calculated on 600,000 SNPs for 42 African populations from the human origins panel. B) Outgroup f3 scores of the Sutherland samples calculated on 540,000 SNPs for 10 Southern African populations from the Schlebusch panel.
(TIF)

S27 Fig a) Heatmap of outgroup f_3_ values calculated pairwise on 1,200,000 SNPs between all analysed published ancient samples and the 3 Sutherland individuals. b) Corresponding Multidimensional-scaling-plot (MDS) of the outgroup f_3_ values calculated pairwise between all ancient samples and the Sutherland individuals.
(TIF)

S28 Fig a) F_ST_ scores calculated for all 3 Sutherland individuals grouped together on 600,000 SNPs for 42 African populations from the human origins panel. b) F_ST_ scores calculated for all 3 Sutherland individuals grouped together on 540,000 SNPs for 10 southern African populations from the Schlebusch panel.
(TIF)

S29 Fig a) f_4_ statistic of the form f_4_ (Outgroup, Test; South_Africa_2000BP, Sutherland), calculated on 600,000 SNPs of the human origins panel. The Test population iterates through 45 ancient and present-day Sub-Saharan African populations. b) f_4_ statistic of the form f_4_ (Outgroup, Sutherland; South_Africa_2000BP, Test), calculated on 600,000 SNPs of the human origins panel. The test population iterates through 45 ancient and present-day sub-Saharan African populations.
(TIF)

S30 Fig a) f_4_ statistic of the form f_4_ (Outgroup, Sutherland; Khomani_San.DG, Test), calculated on 600,000 SNPs of the Human Origins panel. The test population iterates through 44 ancient and present-day sub-Saharan African populations. b) f_4_ statistic of the form f_4_ (Outgroup, Test; Khomani_San.DG, Sutherland), calculated on 600,000 SNPs of the human origins panel. The test population iterates through 43 present-day sub-Saharan African populations.
(TIF)

S31 Fig f_4_ statistic of the form f_4_ (Outgroup, Test; South_Africa_2000BP_East, South_Africa_2000BP_West), calculated on 600,000 SNPs of the human origins panel. The test population iterates through 46 ancient and present-day sub-Saharan African populations.
(TIF)

S32 Fig qpADM ancestry modelling on ~600,000 SNPs of selected modern and ancient southern African populations from the human origins panel. (TIF)

S33 Fig a) Admixture graph of six selected present-day and ancient sub-Saharan African populations from the 1240k panel allowing for one admixture event. b) Admixture graph of seven selected present-day and ancient sub-Saharan African populations from the human origins panel, allowing for two admixture events.
(TIF)

S34 Fig δ^15^N and δ^13^C values of serial samples of dentine (reflecting diet in early life) compared with bone from the same individuals. (JPG)

S1 File (DOCX)

S1 Raw images (PDF)

S1 Dataset Genetic analyses sample statistics overview used reference data, mitochondrial DNA and Y chromosome haplogroup determination, and qpAdm modelling. (XLSX)

S2 Dataset Stable isotope values and collagen quality indicators. (XLSX)
