# Supplementary figures and images for: Confronting historical legacies of biological anthropology in South Africa—Restitution, redress and community-centered science: The Sutherland Nine
Source: PLoS One. 2023 May 24;18(5):e0284785. doi: 10.1371/journal.pone.0284785 (PMC10208512; doi:10.1371/journal.pone.0284785)

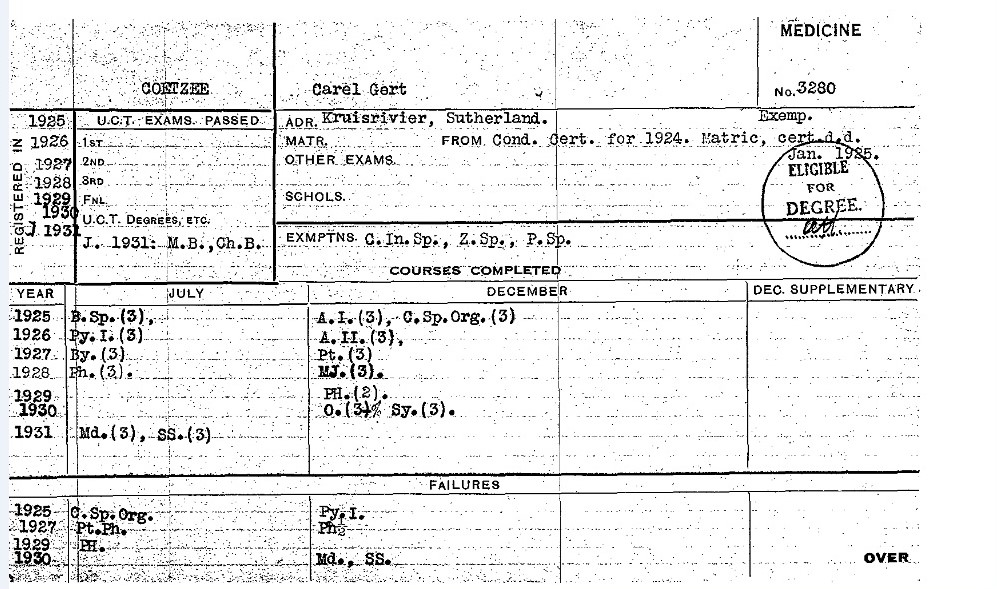

Supplement: S1 Fig — It gives his place of residence as Kruisrivier Farm, Sutherland. (TIF) [file pone.0284785.s001.tif]

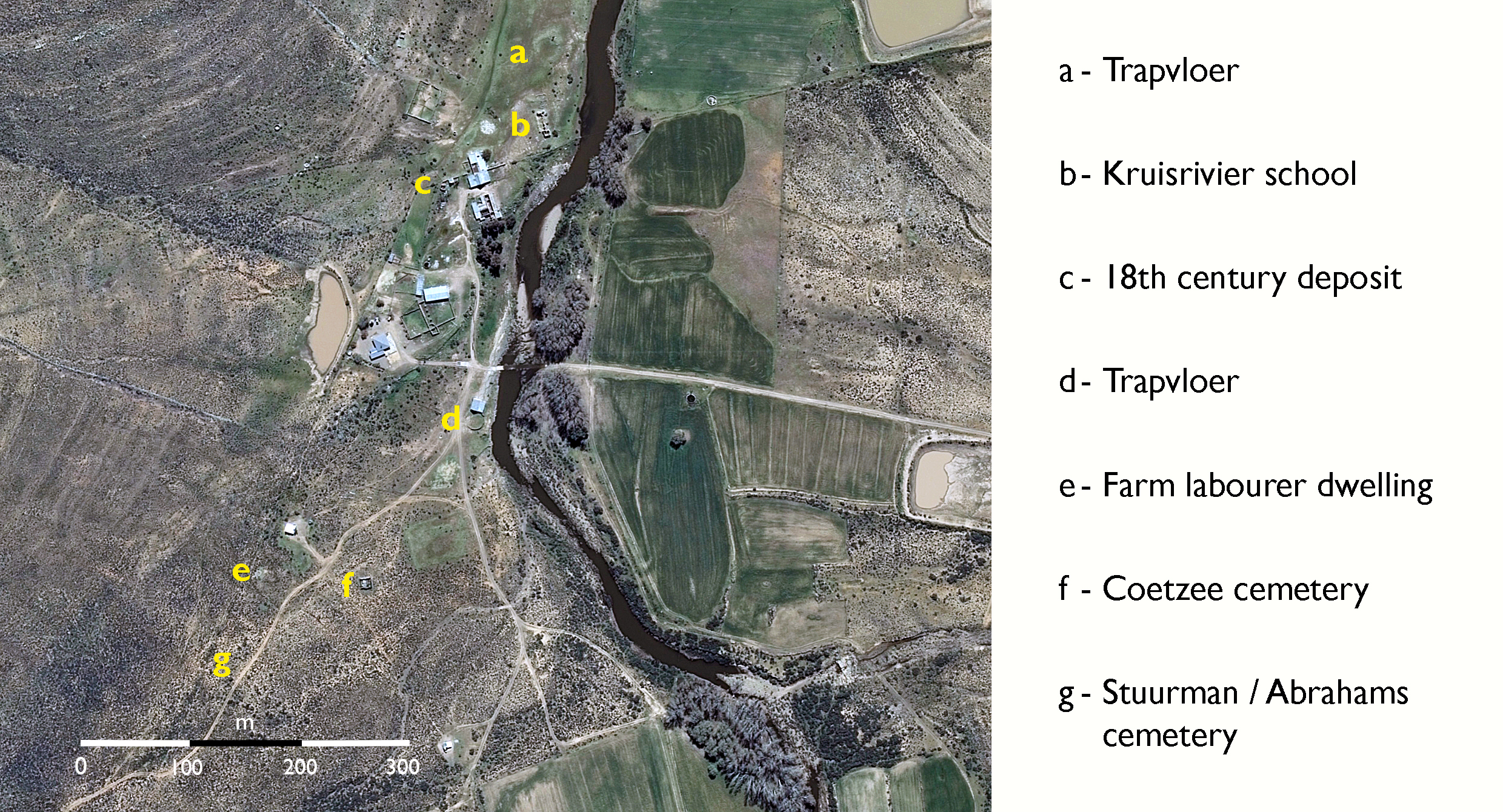

Supplement: S2 Fig — a and d both indicate threshing floors; b is the Kruisrivier school; c is an 18th century archaeological deposit; e was a farm labourer dwelling; f is the Coetzee family cemetery; and g is the farm labourers’ cemetery. (TIF) [file pone.0284785.s002.tif]

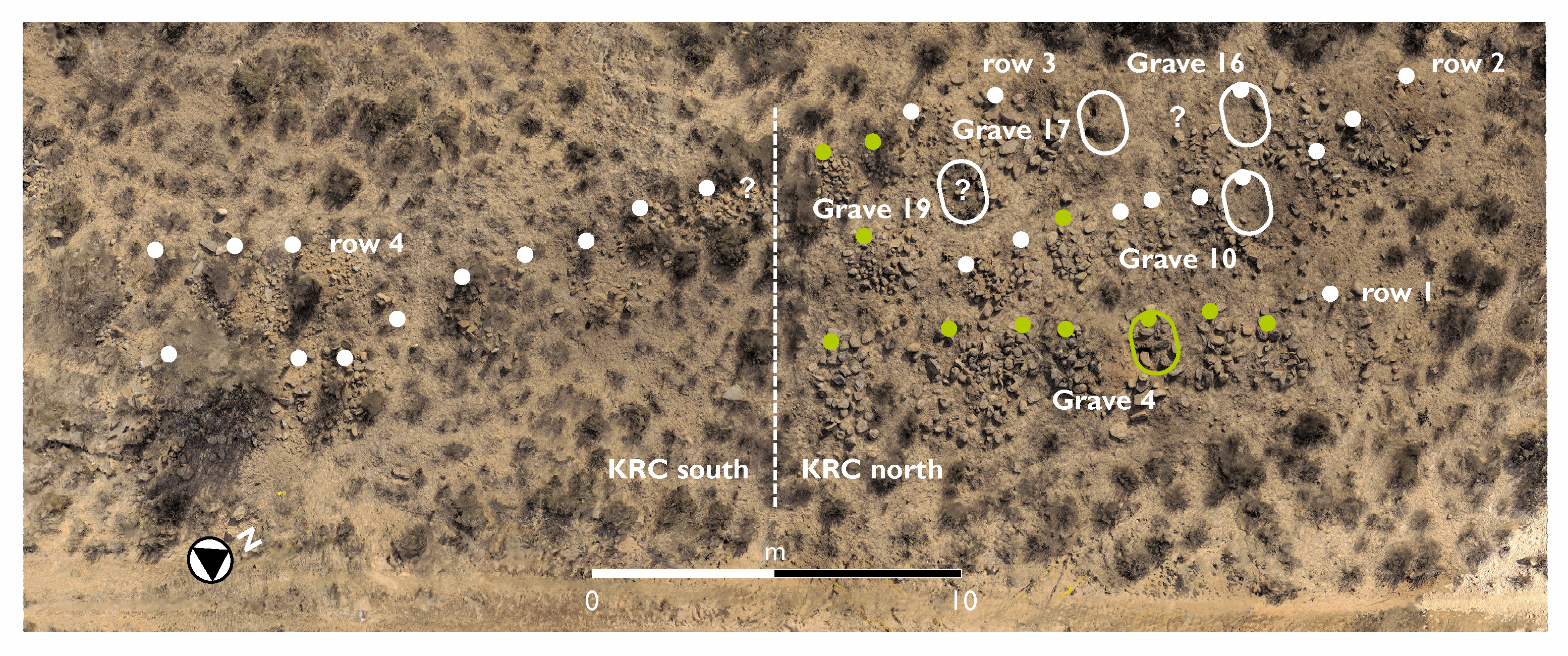

Supplement: S3 Fig — Green dots indicate the western ends of stone cairn burials (n = 11), white dots the headstone (western) ends of headstone and footstone burials (n = 25). Green ring indicates disturbed cairn burial, white rings disturbed headstone and footstone burials. (TIF) [file pone.0284785.s003.tif]

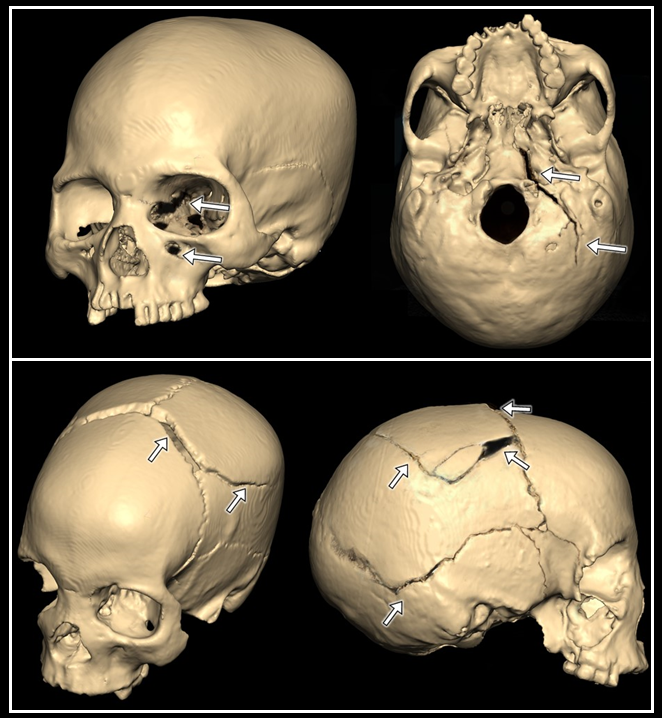

Supplement: S4 Fig — Top is Klaas’s cranium, illustrating the perimortem trauma. Left anterolateral view, shows entrance wound on the anterior surface of the left maxilla inferior to the orbit and medial to the left infraorbital foramen. A radiating fracture extends through the left lacrimal, ethmoid and sphenoid bones. Top right shows the inferior view, with a radiating base-of-skull fracture through the occipital bone on the left side of the foramen magnum. The bottom images are of Igue We’s cranium illustrating the perimortem trauma. Bottom left, left superolateral view, radiating fractures from the points of impact on the right are observed dissipating through to the sagittal and coronal sutures; a portion of this energy dissipates into the left parietal bone with production of a primary radiating fracture extending posteriorly, which terminates inferior to the left eurion. Bottom right, two points of impact are observed with radiating fractures. (TIF) [file pone.0284785.s004.tif]

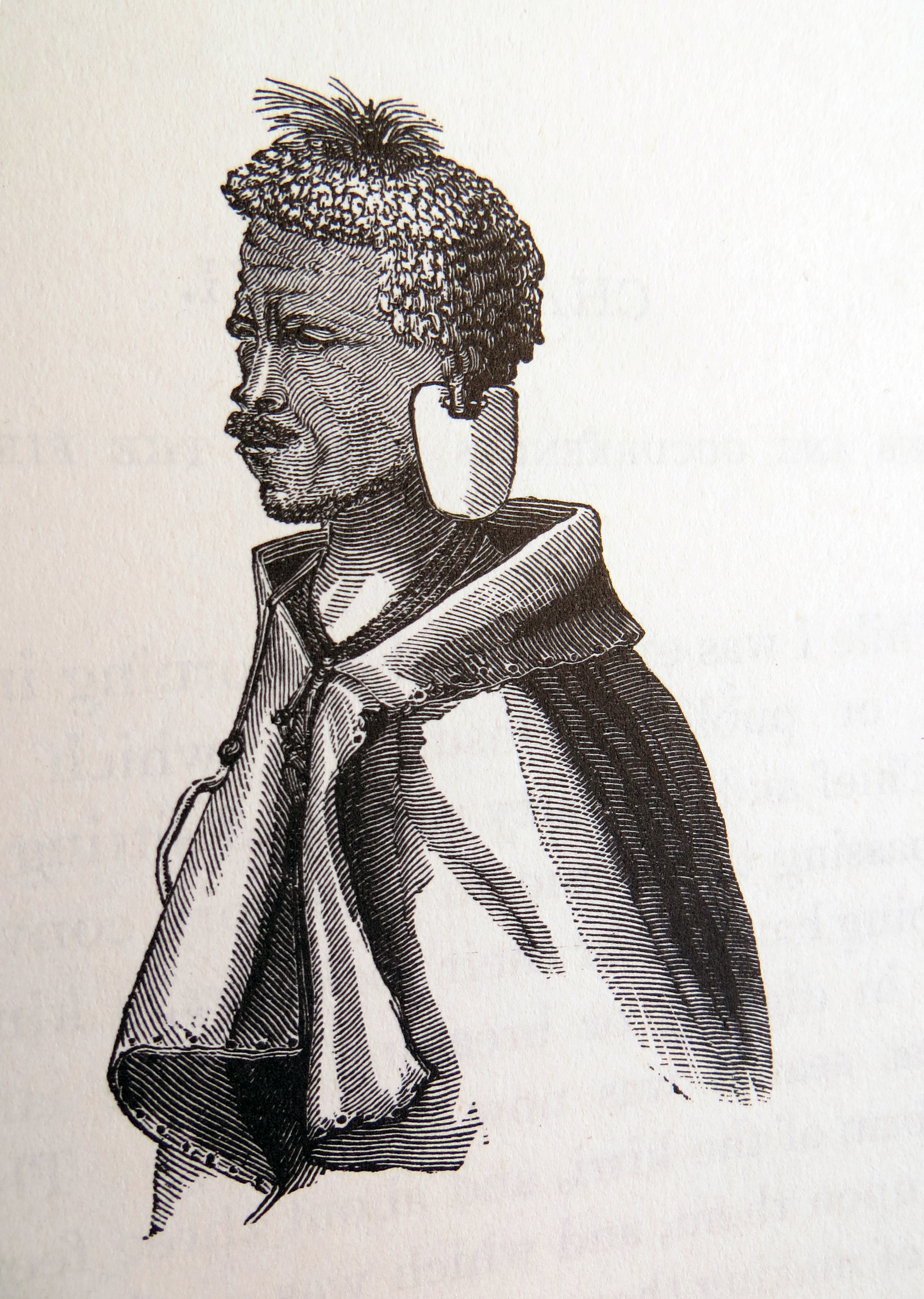

Supplement: S5 Fig — (TIF) [file pone.0284785.s005.tif]

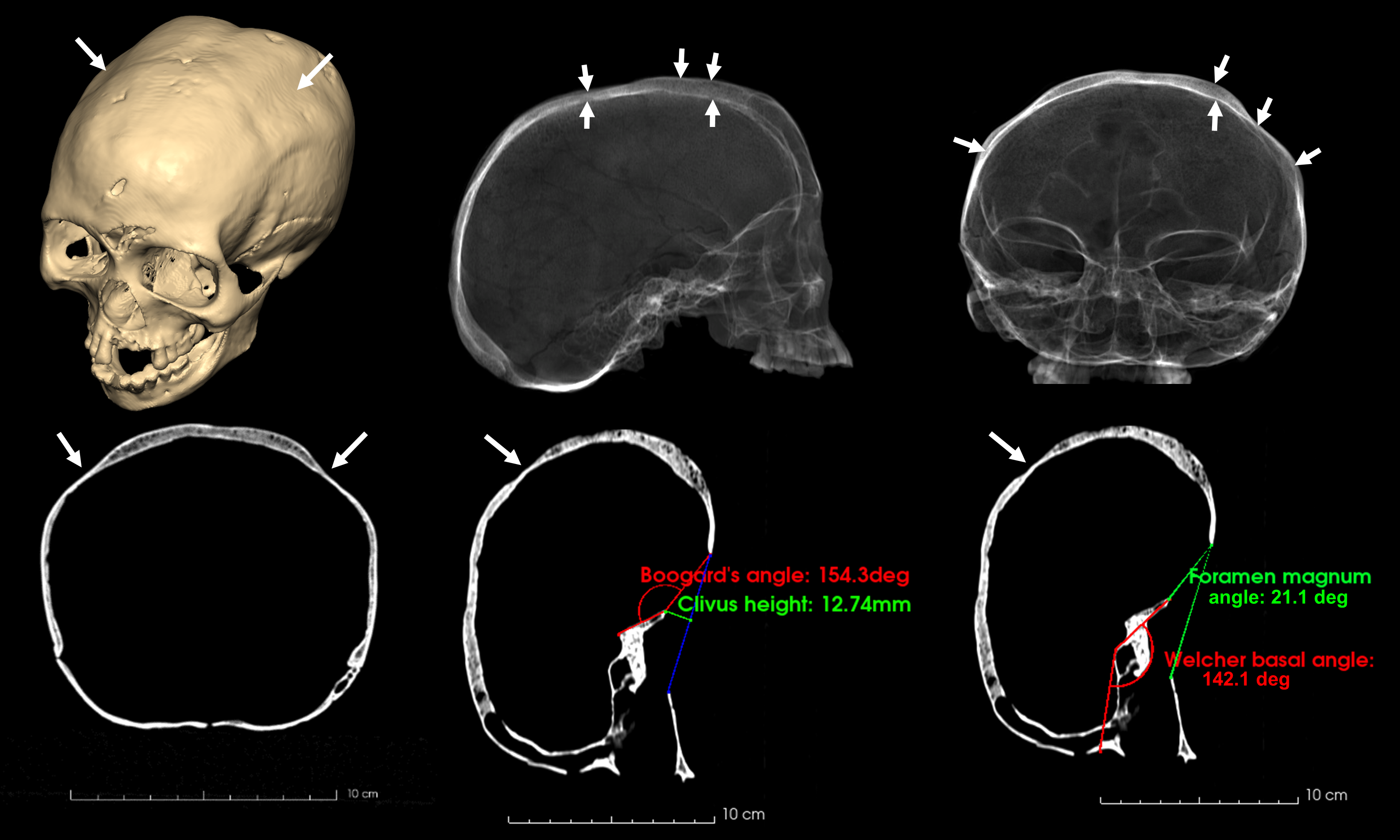

Supplement: S6 Fig — An anterior left superolateral view (top left) and the 2D reconstructed CT images (bottom left, middle and right) show three major impressions in the cranium (white arrows) as a result of calvarial thinning. The Lodox images (top middle and right) illustrate the presence of osteoporosis as evidenced by a uniform decrease in radiodensity, suggesting extensive rarefaction of spongy bone. The white arrows in these two images further illustrate examples of thinning and rarefaction of cortical bone, observed as thin, faded or non-existent lines of radiodensity. Mid-sagittal reconstructed CT images (bottom middle and right) illustrate metrics evaluated for the diagnosis of platybasia and basilar invagination. (TIF) [file pone.0284785.s006.tif]

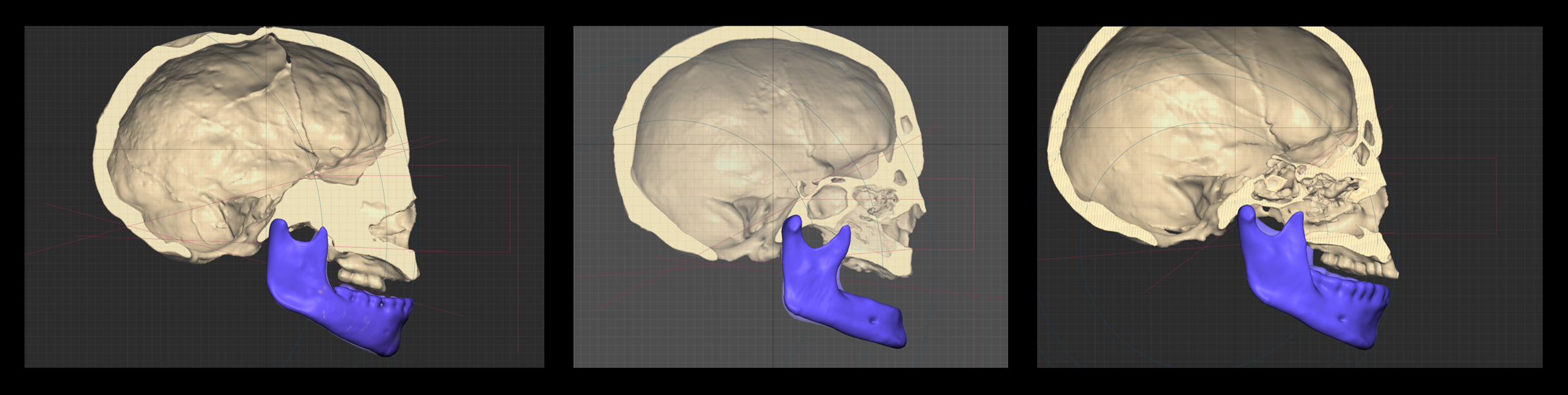

Supplement: S7 Fig — (TIF) [file pone.0284785.s007.tif]

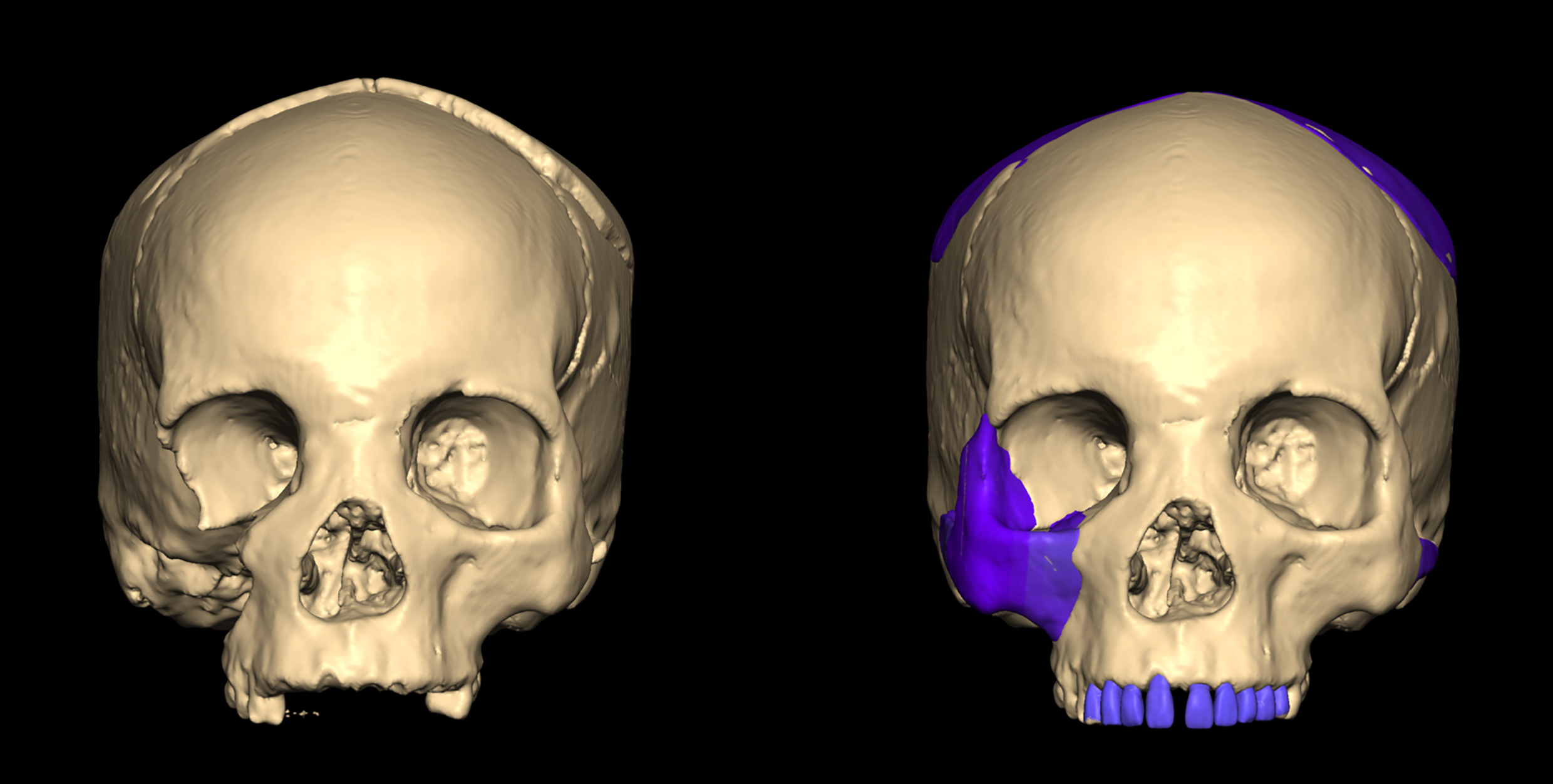

Supplement: S8 Fig — (TIF) [file pone.0284785.s008.tif]

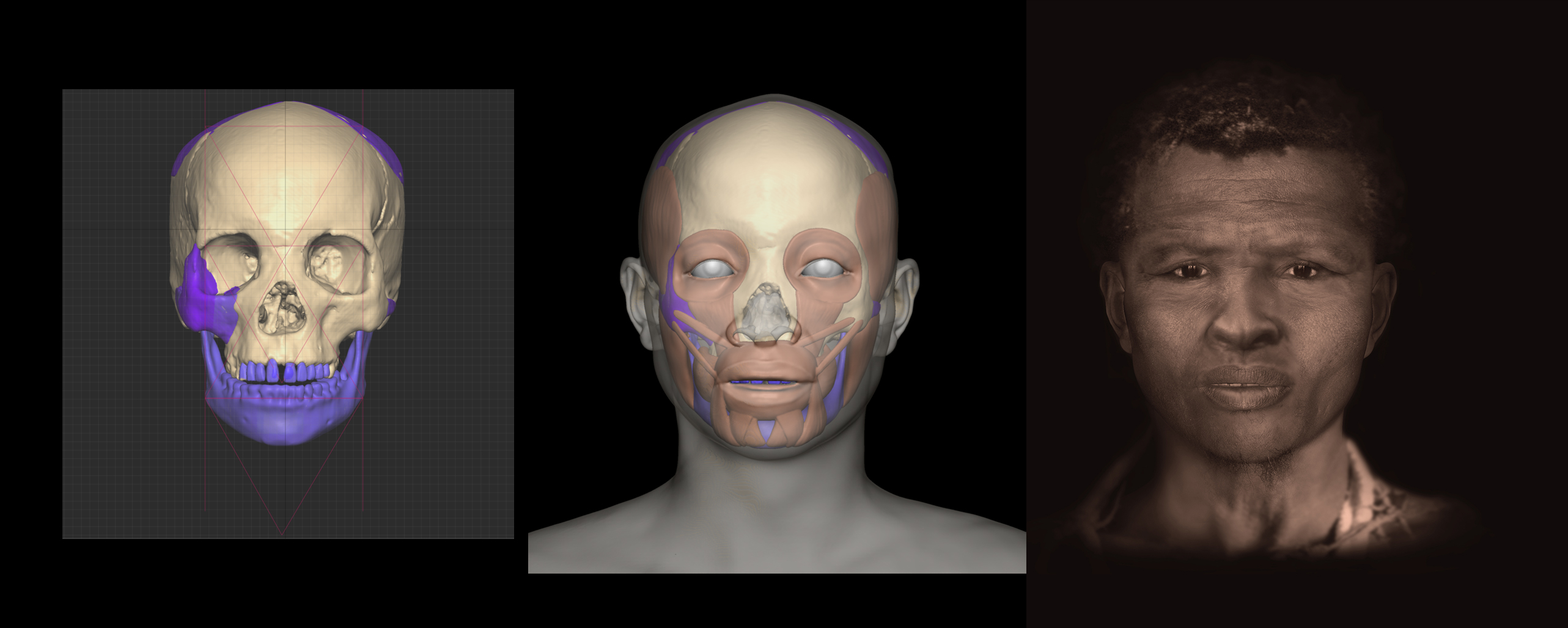

Supplement: S9 Fig — (TIF) [file pone.0284785.s009.tif]

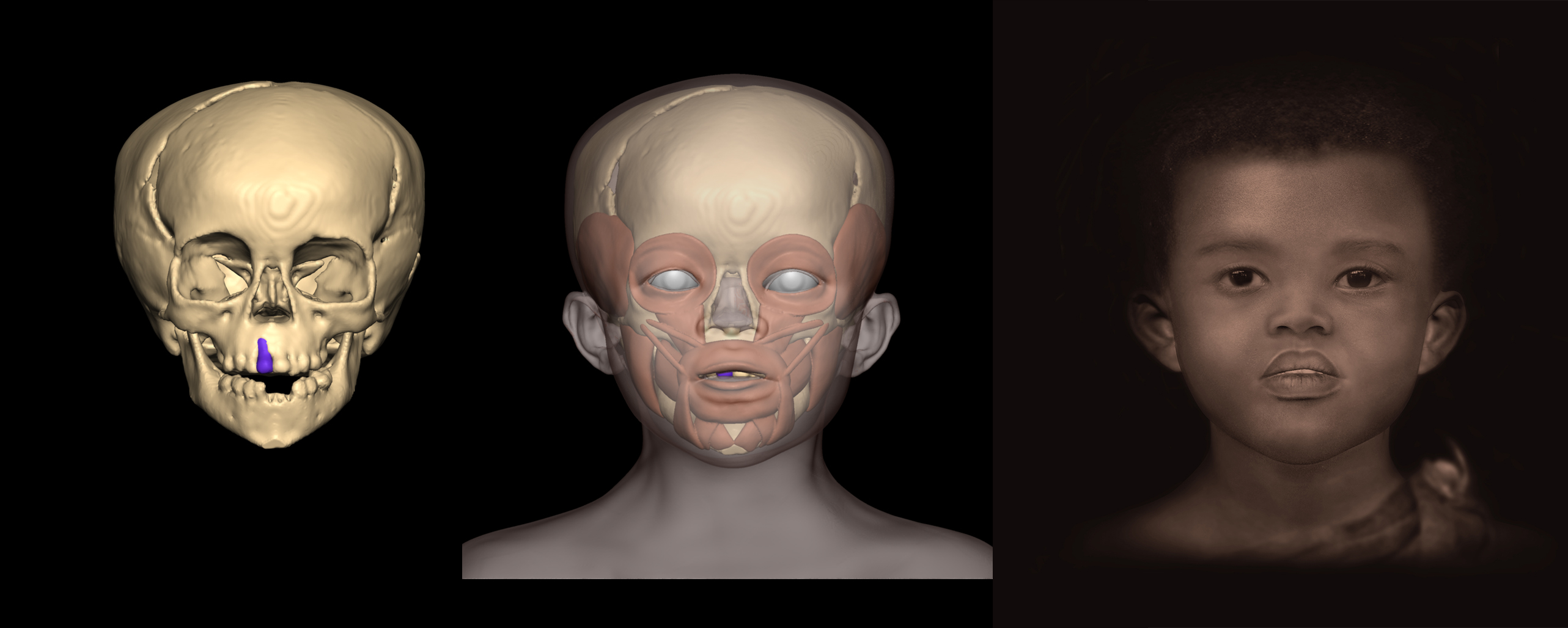

Supplement: S10 Fig — (TIF) [file pone.0284785.s010.tif]

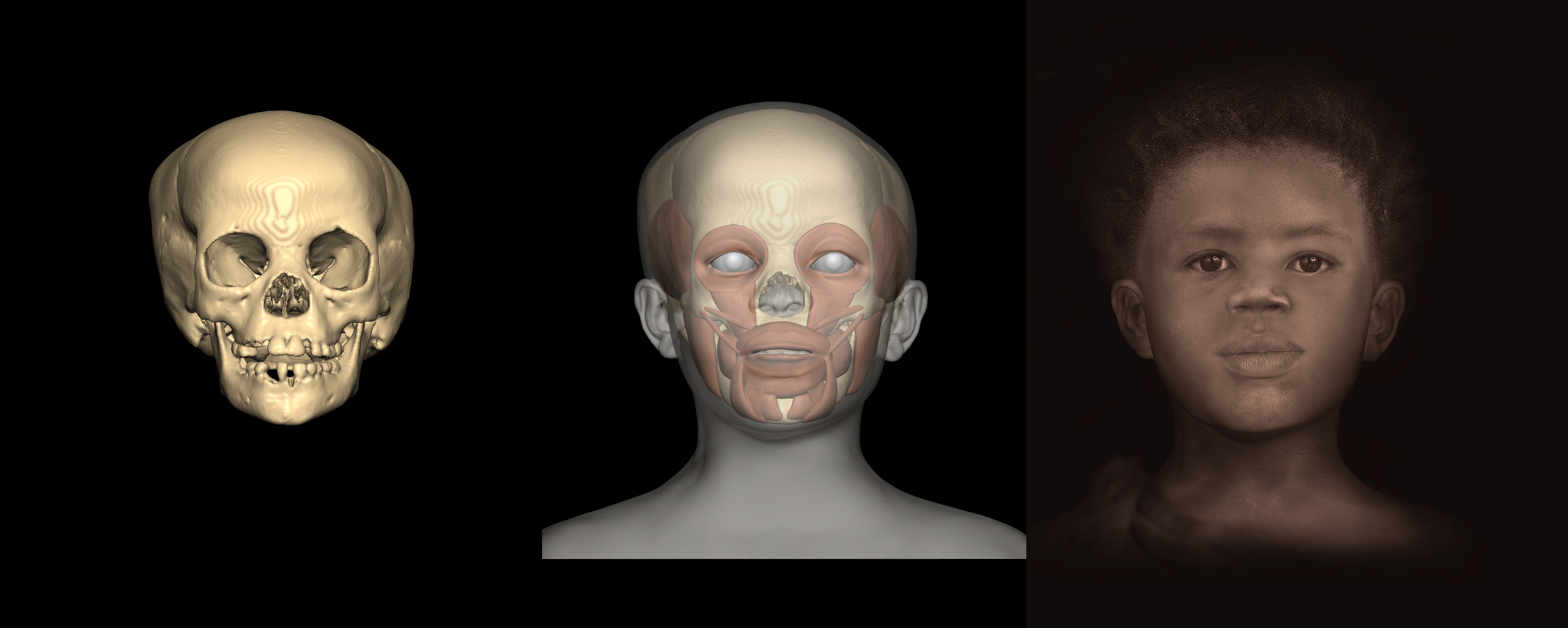

Supplement: S11 Fig — (TIF) [file pone.0284785.s011.tif]

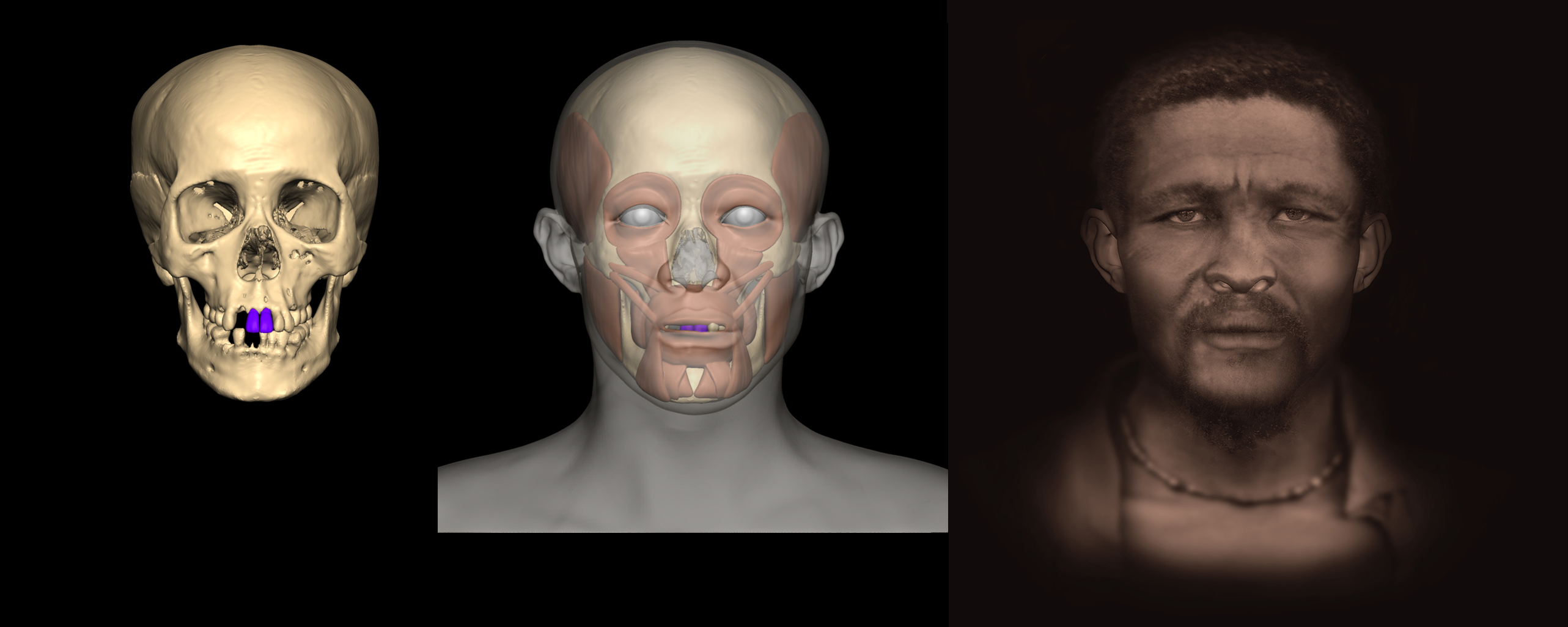

Supplement: S12 Fig — (TIF) [file pone.0284785.s012.tif]

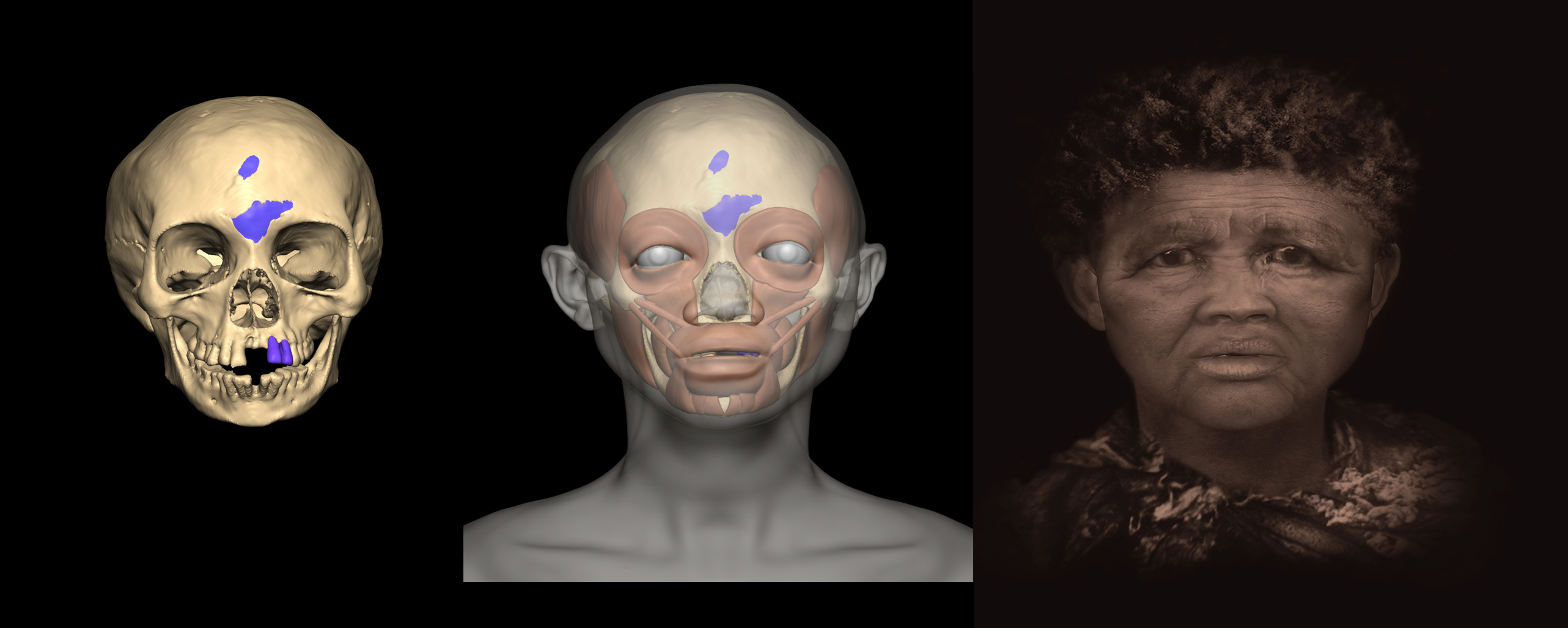

Supplement: S13 Fig — (TIF) [file pone.0284785.s013.tif]

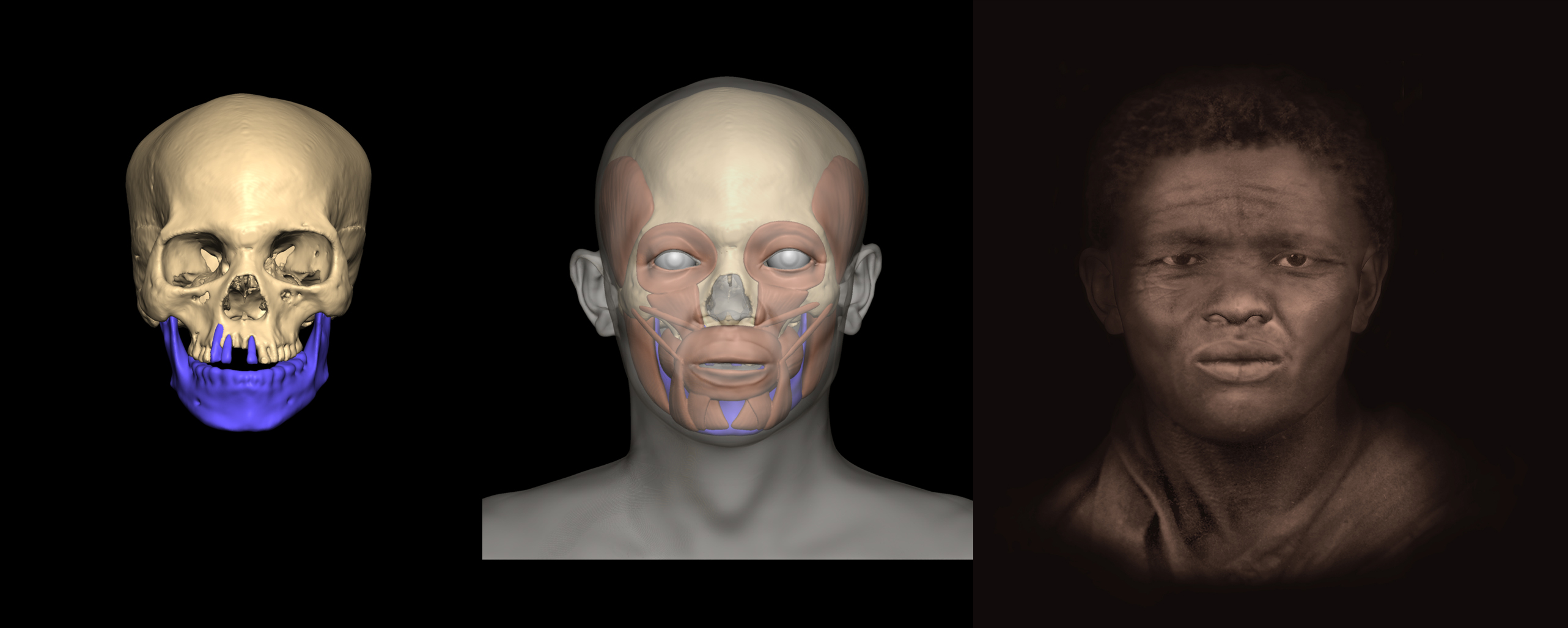

Supplement: S14 Fig — (TIF) [file pone.0284785.s014.tif]

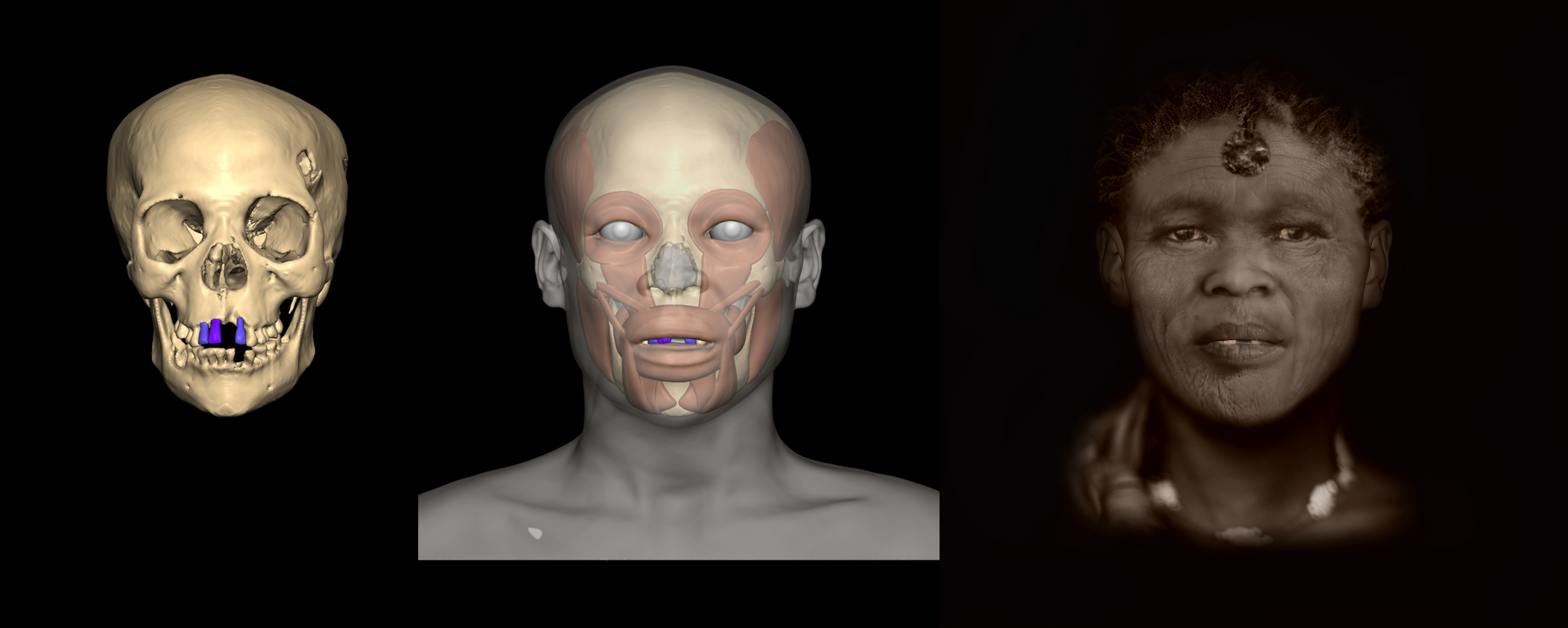

Supplement: S15 Fig — (TIF) [file pone.0284785.s015.tif]

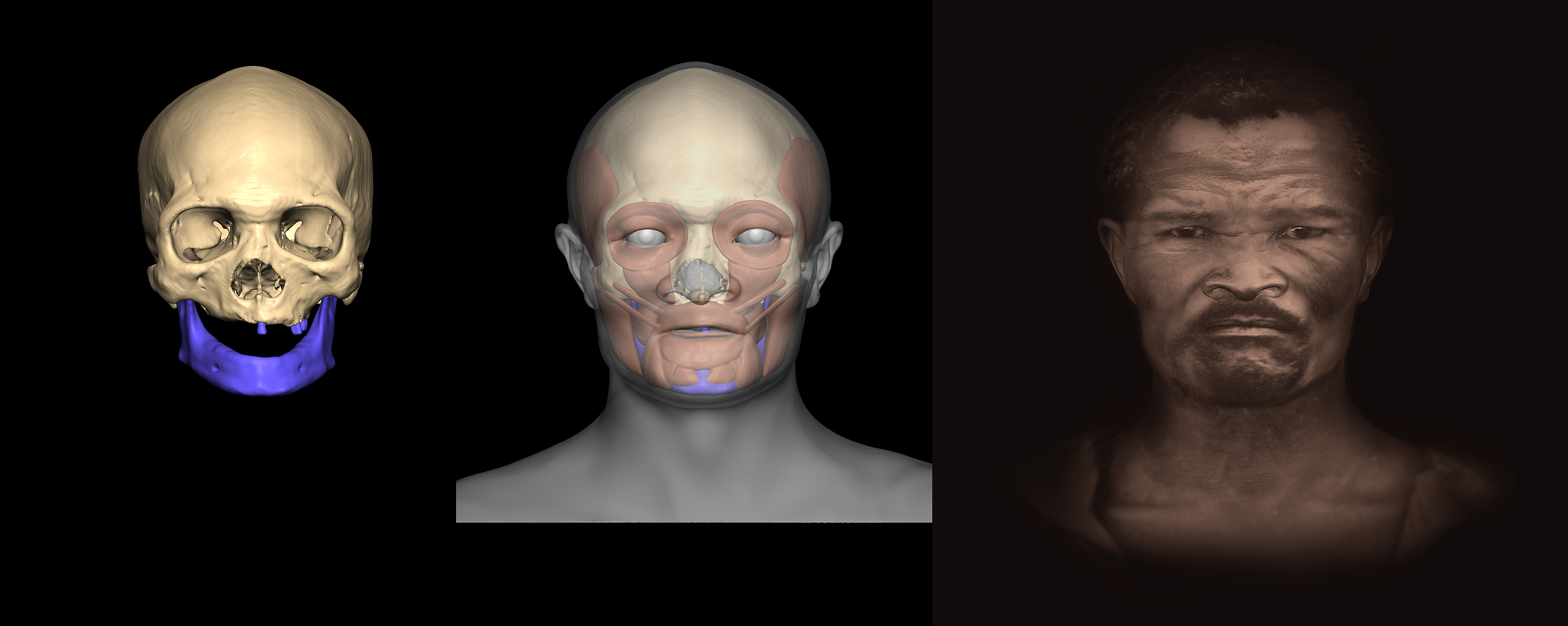

Supplement: S16 Fig — (TIF) [file pone.0284785.s016.tif]

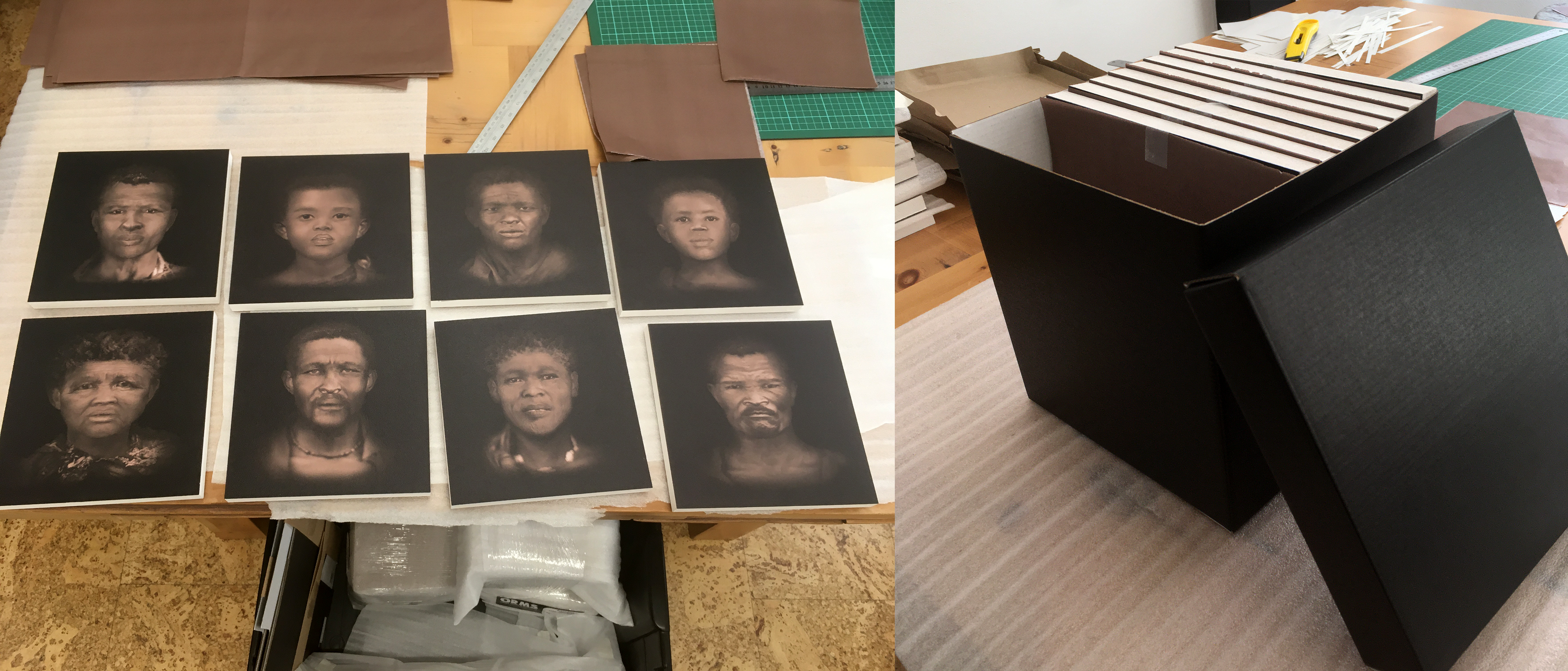

Supplement: S17 Fig — (TIF) [file pone.0284785.s017.tif]

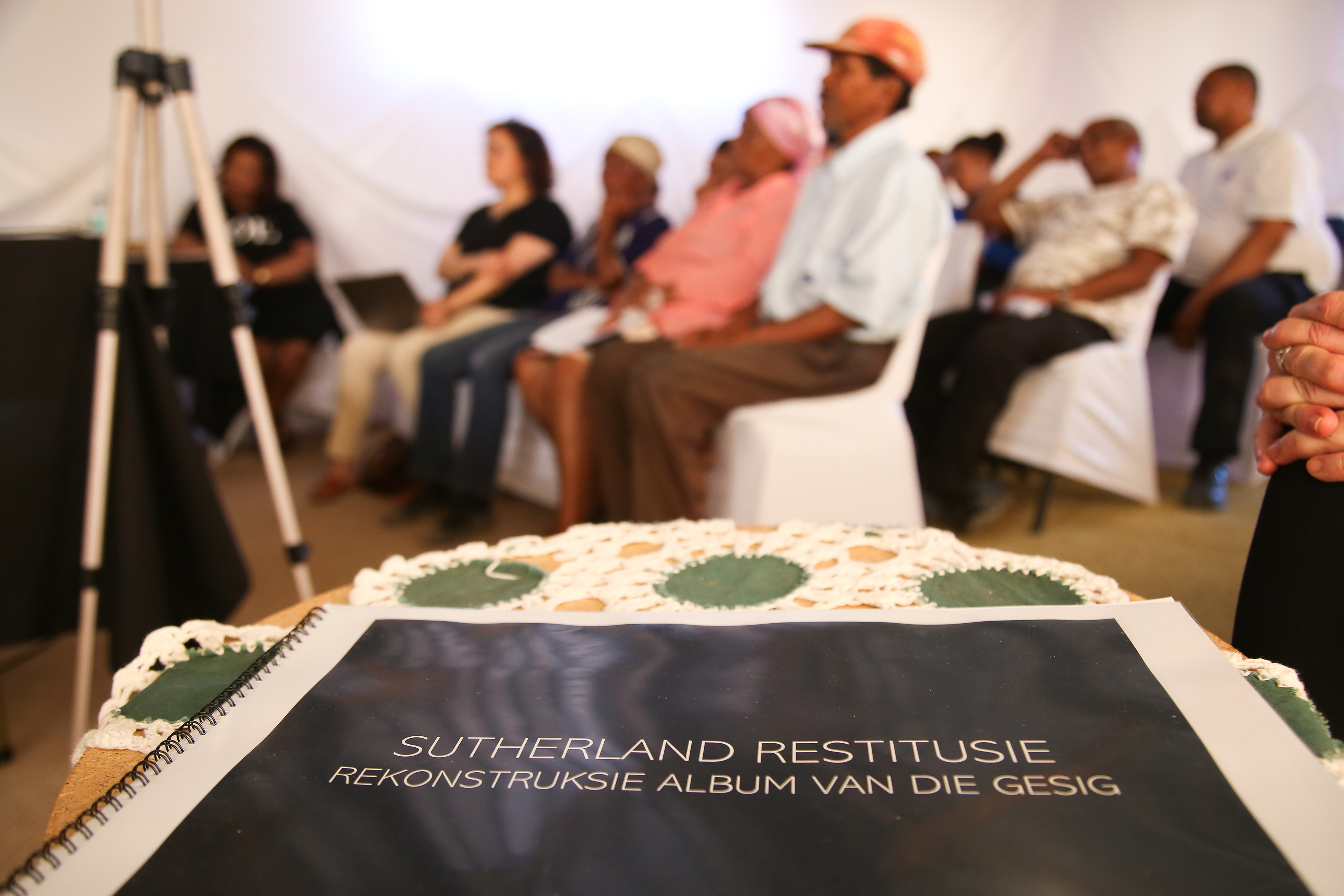

Supplement: S18 Fig — The panels and albums were presented at a knowledge sharing session for the families in Sutherland, October 2019. (TIF) [file pone.0284785.s018.tif]

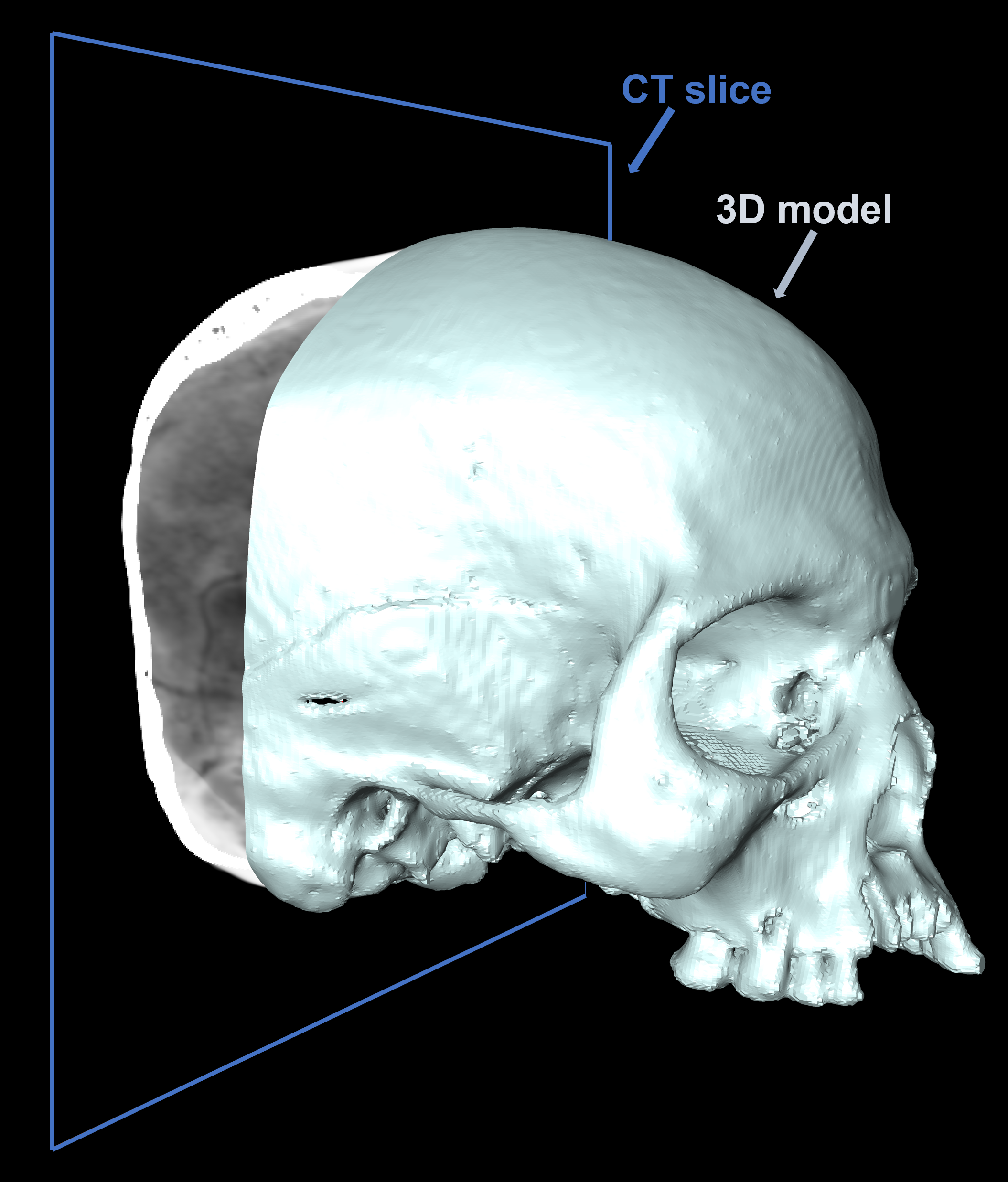

Supplement: S19 Fig — (TIF) [file pone.0284785.s019.tif]

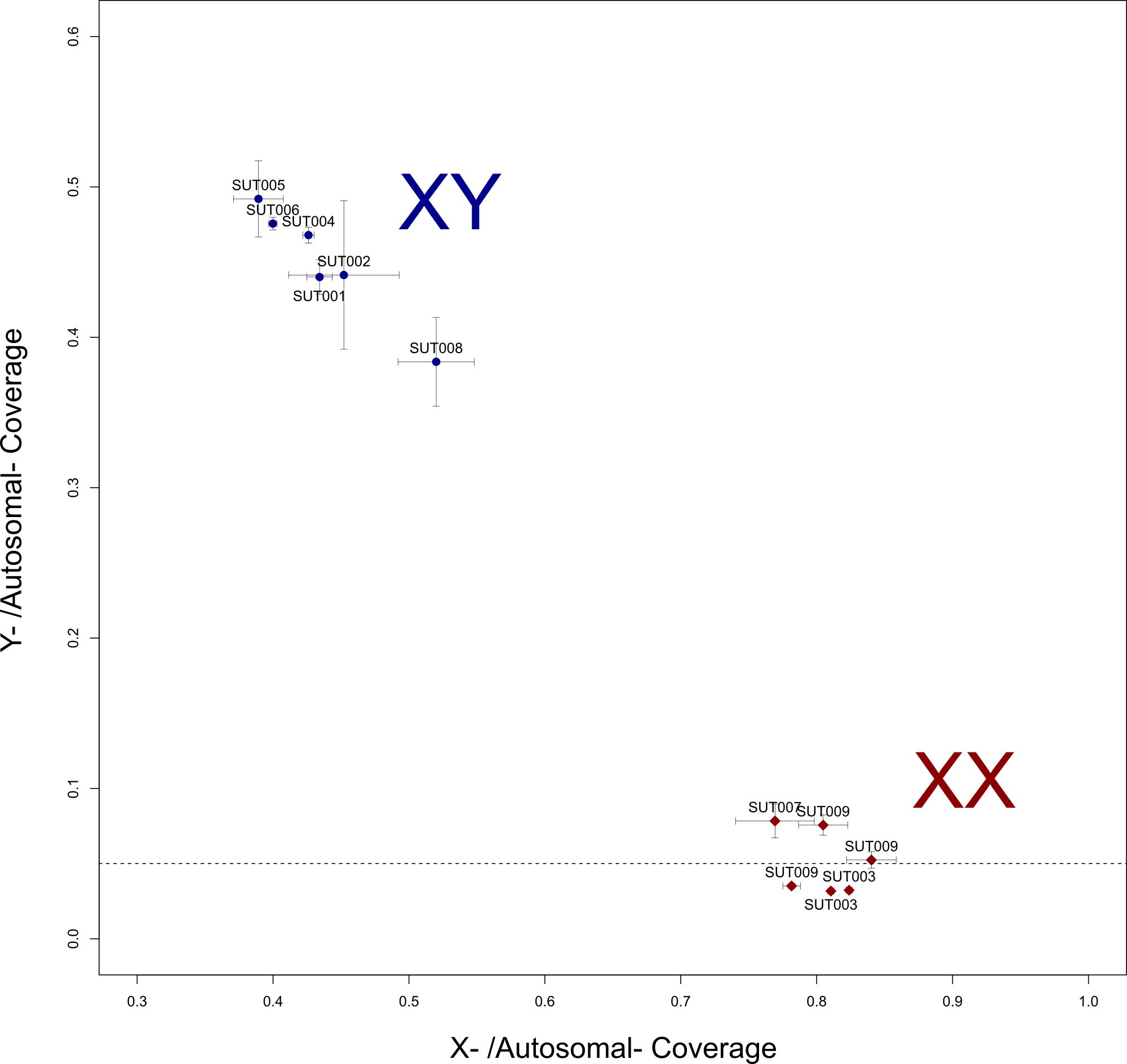

Supplement: S20 Fig — Error bars represent the uncertainty in the calculation of relative coverages. (TIF) [file pone.0284785.s020.tif]

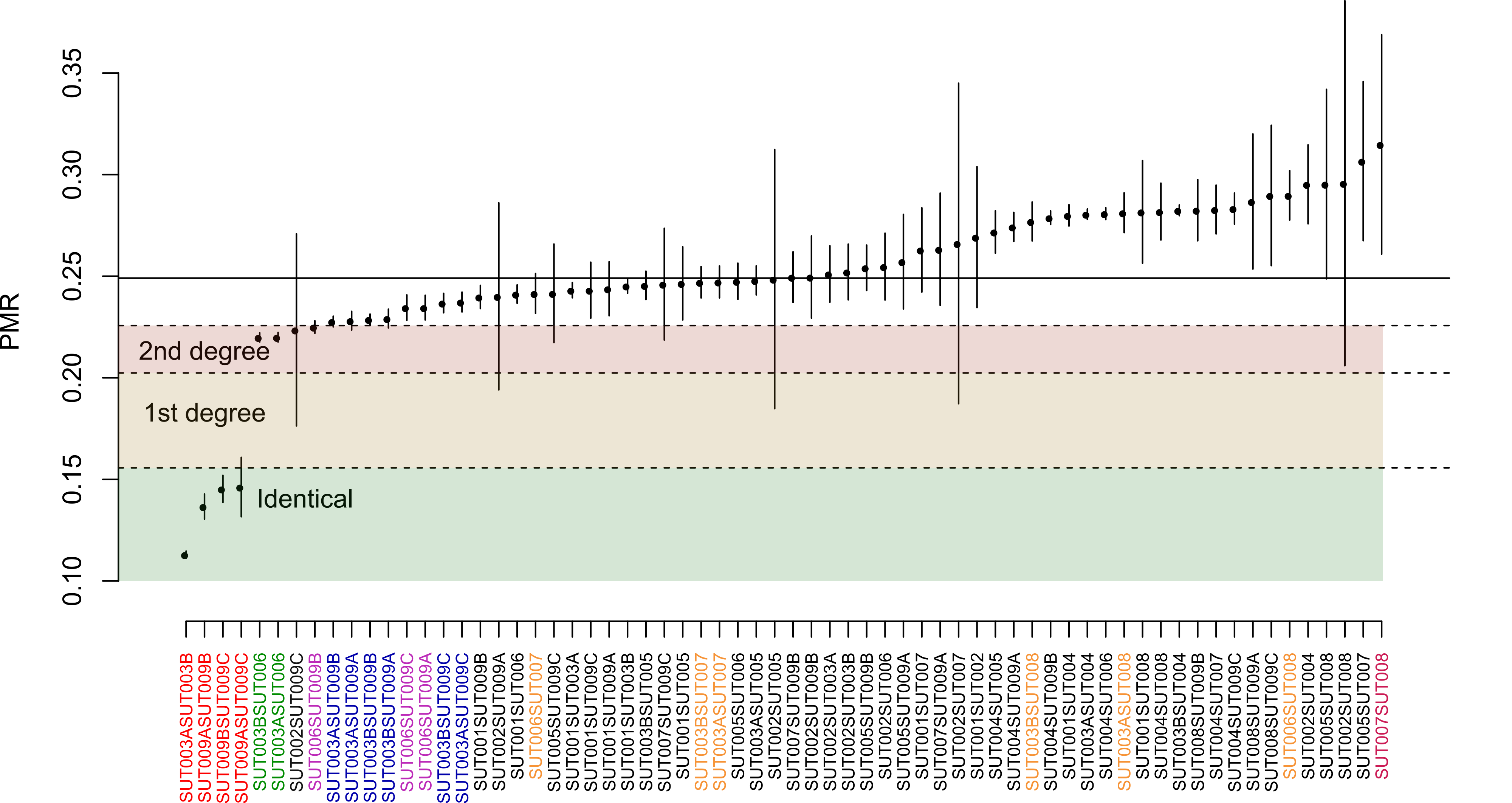

Supplement: S21 Fig — Individual pairs of interest are highlighted accordingly. (TIF) [file pone.0284785.s021.tif]

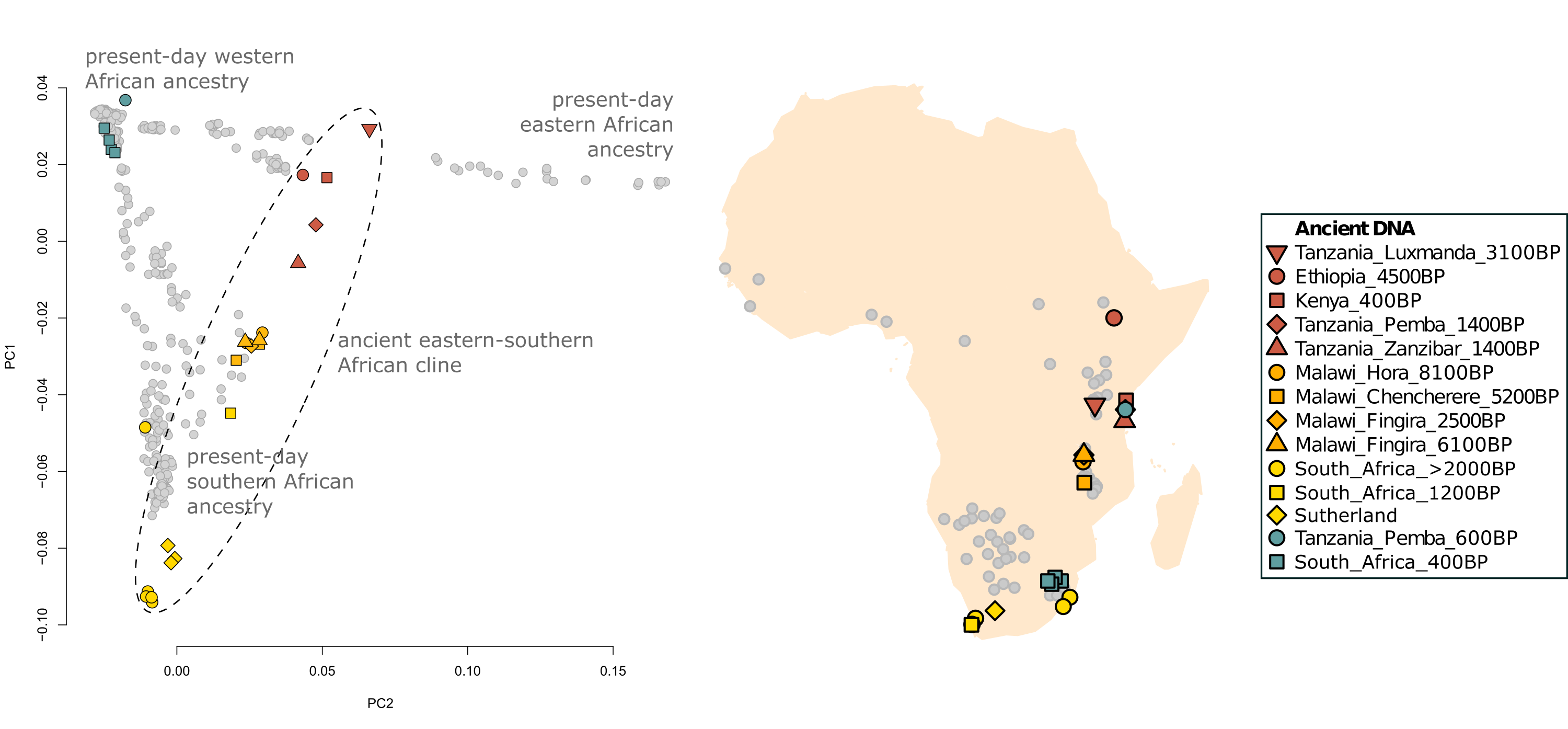

Supplement: S22 Fig — (TIF) [file pone.0284785.s022.tif]

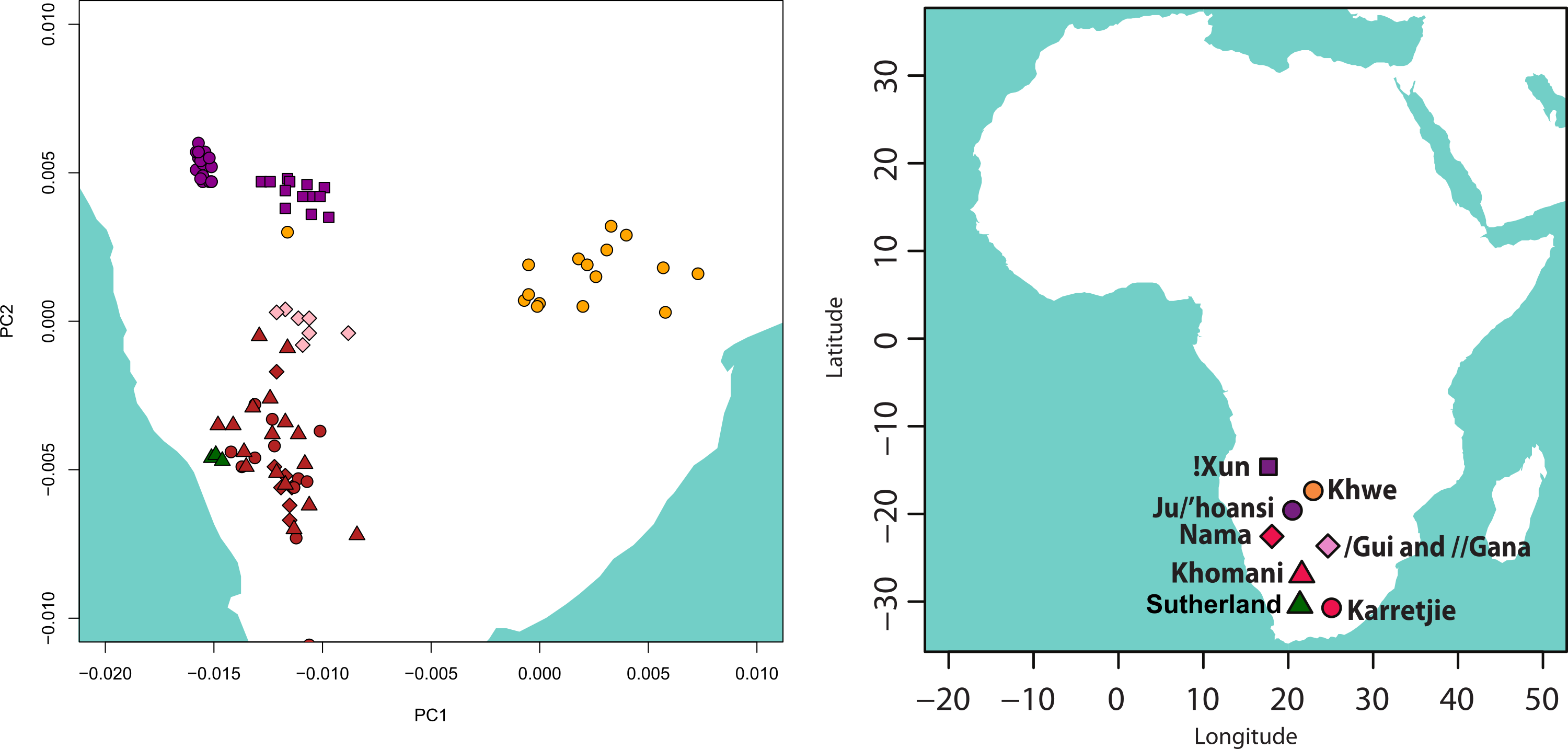

Supplement: S23 Fig — (TIF) [file pone.0284785.s023.tif]

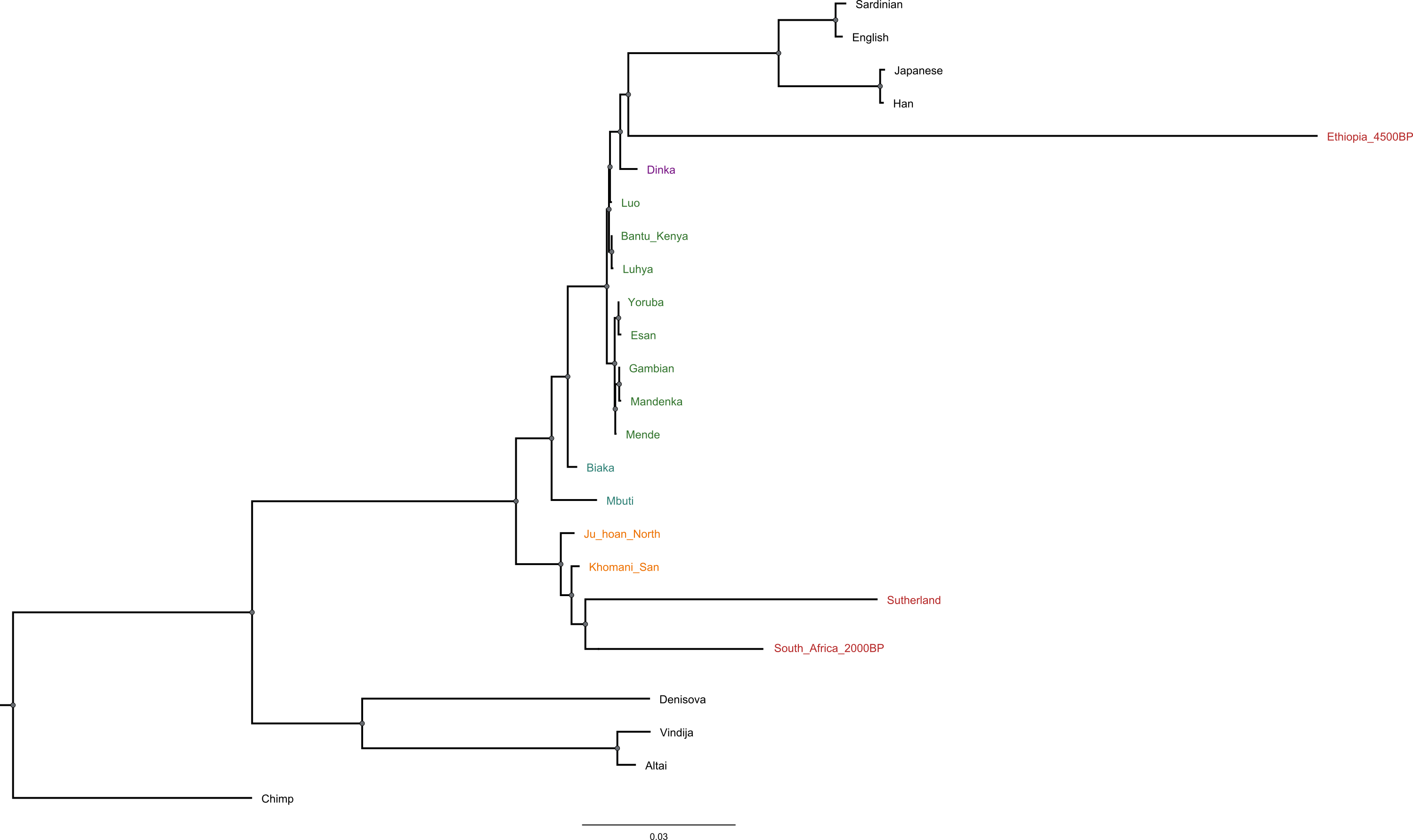

Supplement: S24 Fig — (TIF) [file pone.0284785.s024.tif]

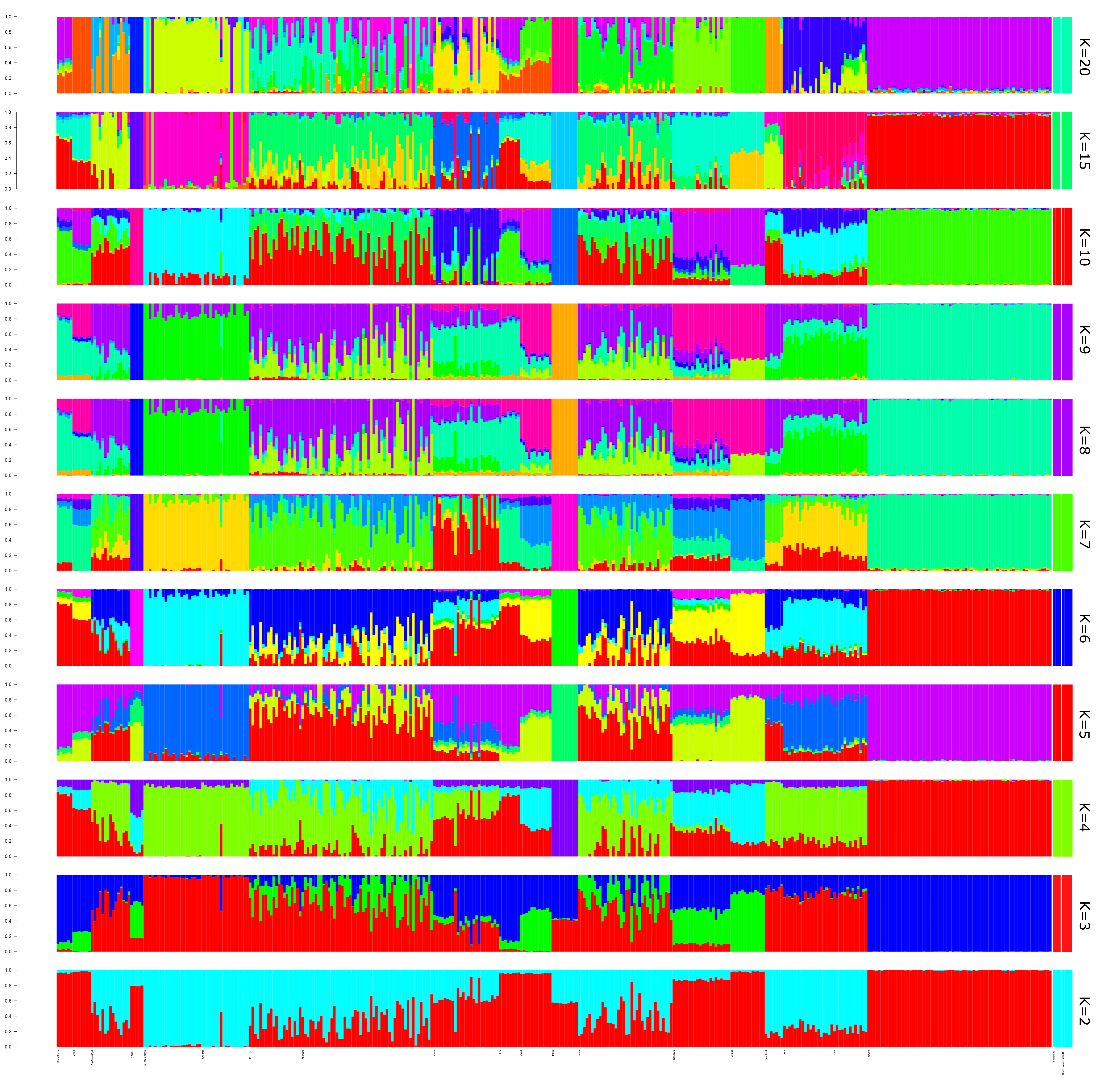

Supplement: S25 Fig — (TIF) [file pone.0284785.s025.tif]

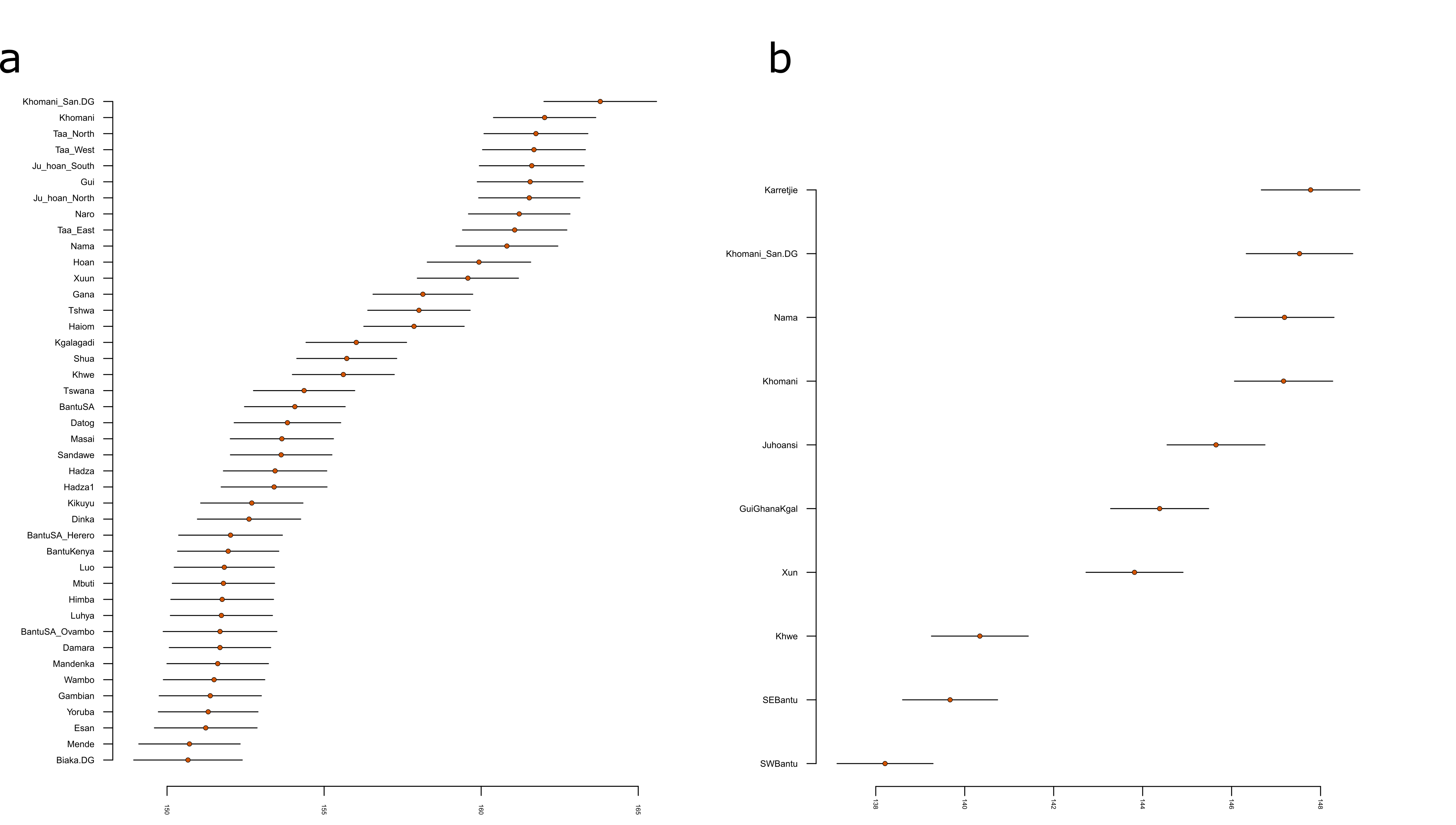

Supplement: S26 Fig — a) Outgroup f3 scores of the Sutherland samples calculated on 600,000 SNPs for 42 African populations from the human origins panel. B) Outgroup f3 scores of the Sutherland samples calculated on 540,000 SNPs for 10 Southern African populations from the Schlebusch panel. (TIF) [file pone.0284785.s026.tif]

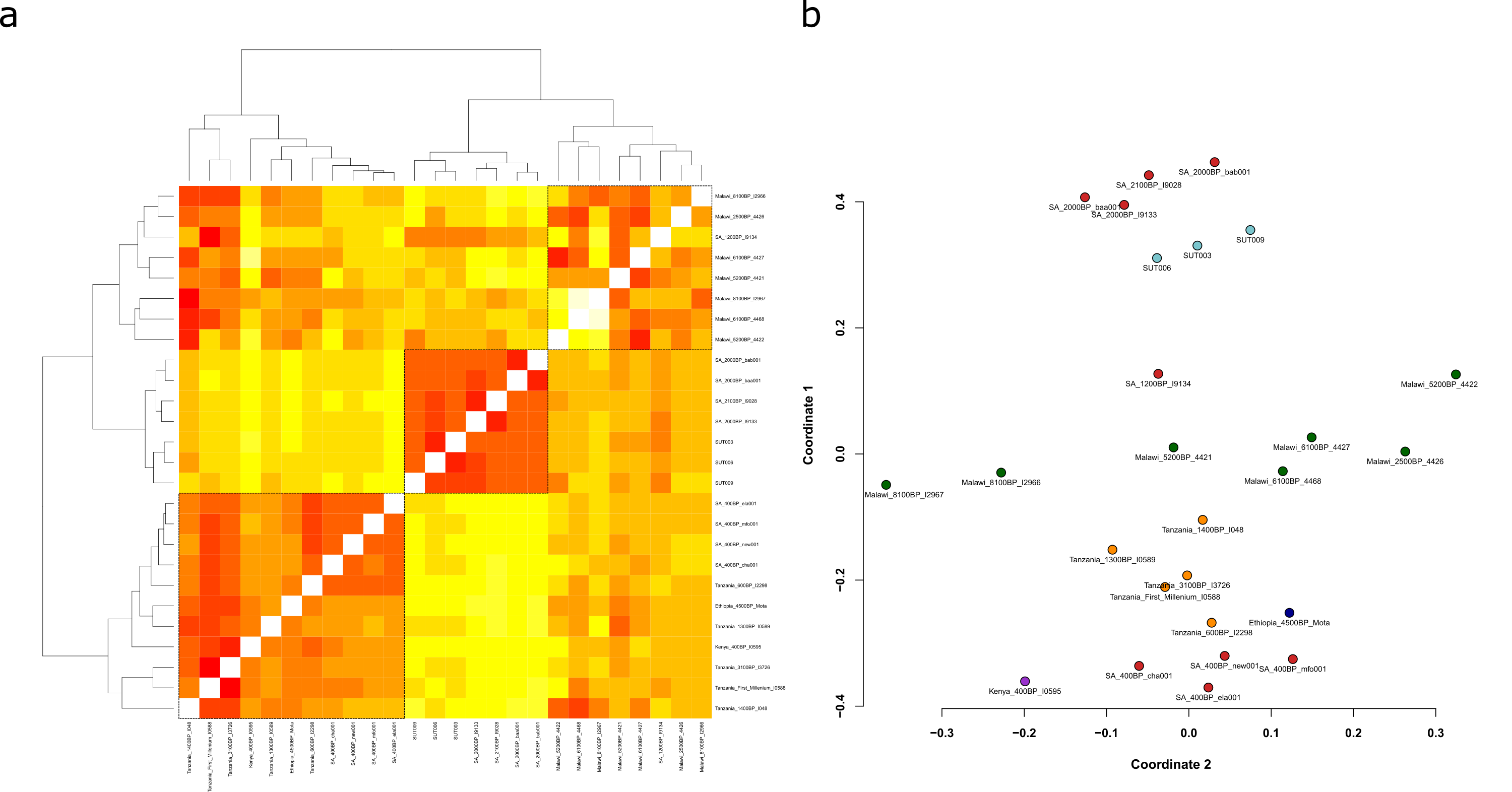

Supplement: S27 Fig — a) Heatmap of outgroup f3 values calculated pairwise on 1,200,000 SNPs between all analysed published ancient samples and the 3 Sutherland individuals. b) Corresponding Multidimensional-scaling-plot (MDS) of the outgroup f3 values calculated pairwise between all ancient samples and the Sutherland individuals. (TIF) [file pone.0284785.s027.tif]

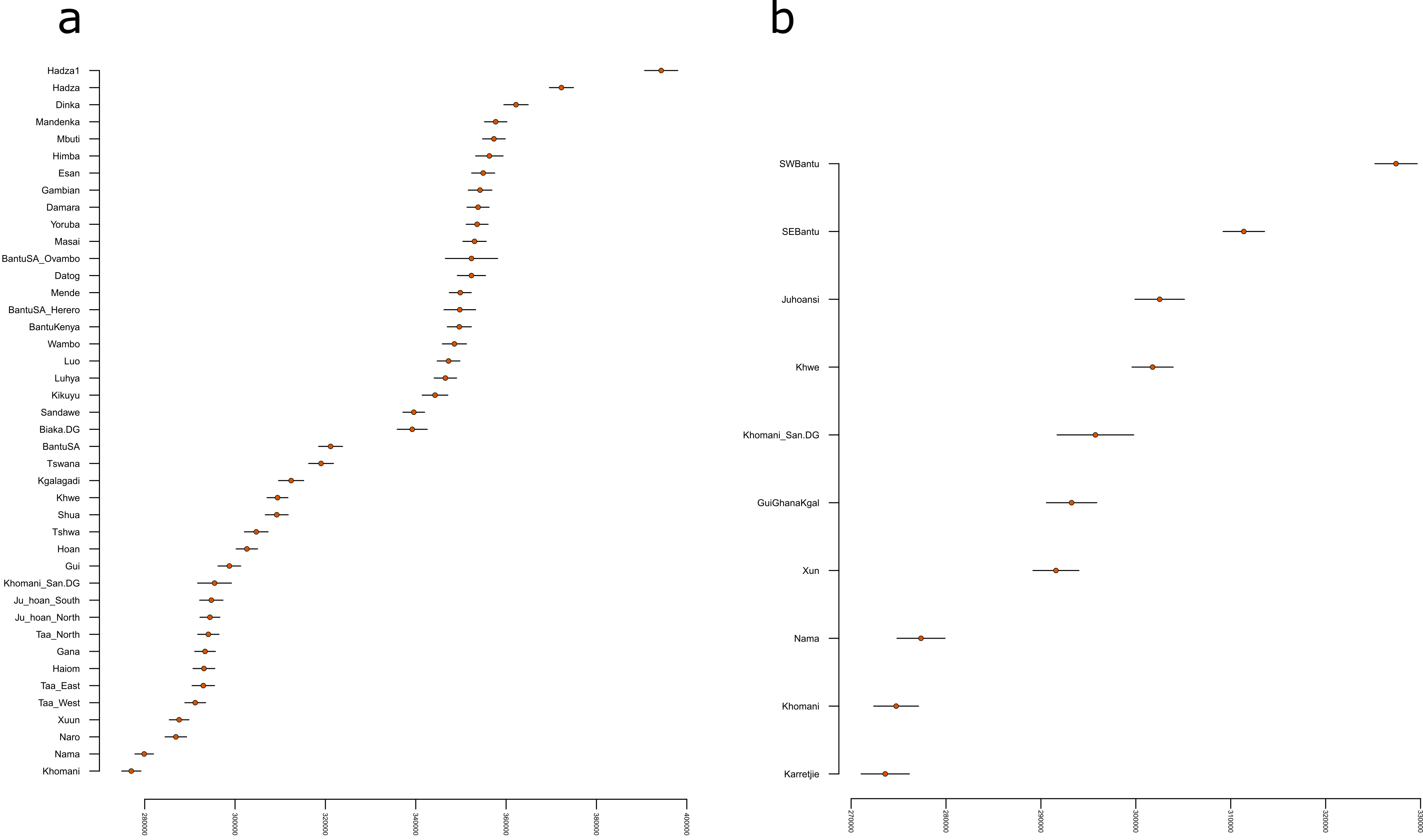

Supplement: S28 Fig — a) FST scores calculated for all 3 Sutherland individuals grouped together on 600,000 SNPs for 42 African populations from the human origins panel. b) FST scores calculated for all 3 Sutherland individuals grouped together on 540,000 SNPs for 10 southern African populations from the Schlebusch panel. (TIF) [file pone.0284785.s028.tif]

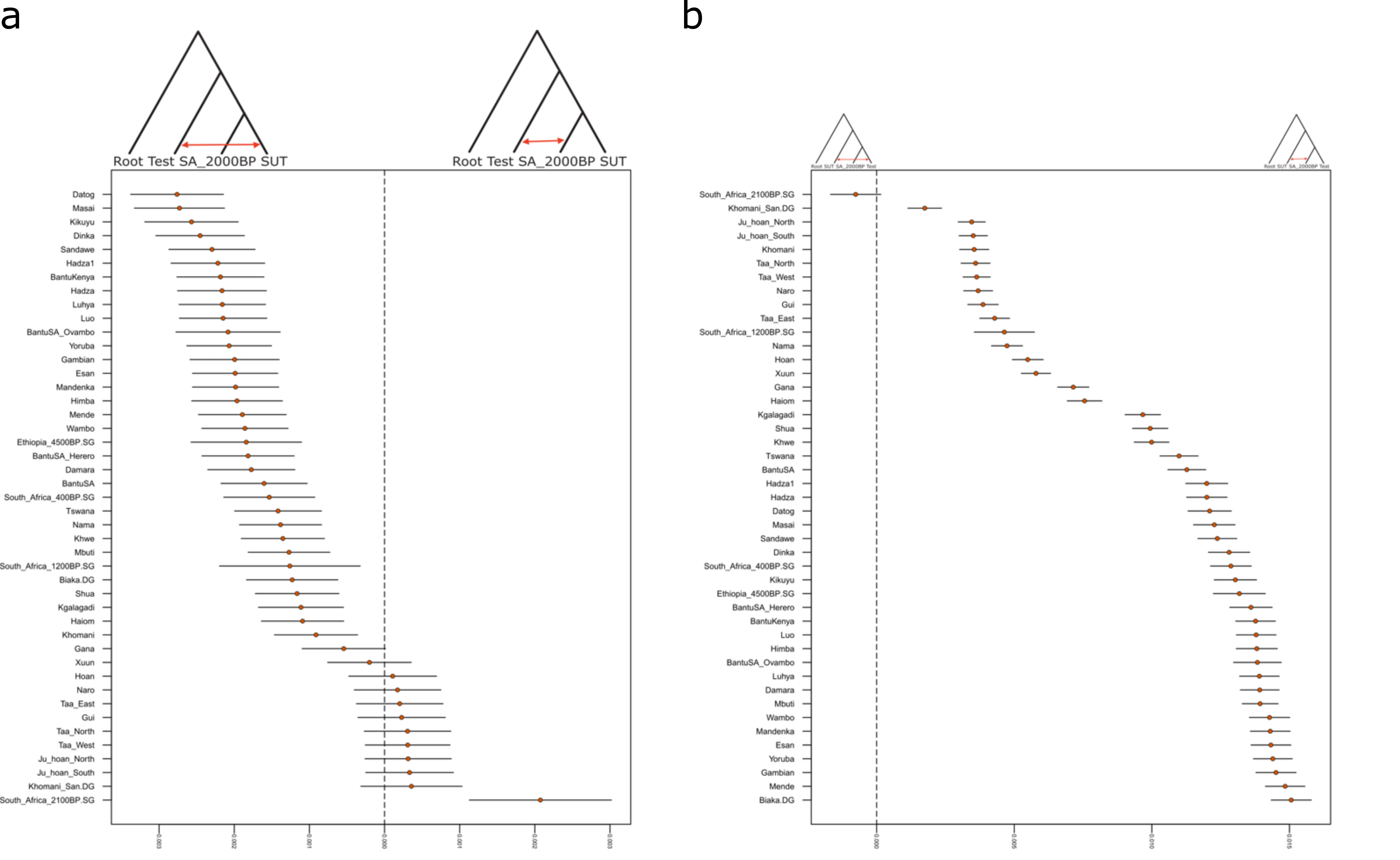

Supplement: S29 Fig — a) f4 statistic of the form f4 (Outgroup, Test; South_Africa_2000BP, Sutherland), calculated on 600,000 SNPs of the human origins panel. The Test population iterates through 45 ancient and present-day Sub-Saharan African populations. b) f4 statistic of the form f4 (Outgroup, Sutherland; South_Africa_2000BP, Test), calculated on 600,000 SNPs of the human origins panel. The test population iterates through 45 ancient and present-day sub-Saharan African populations. (TIF) [file pone.0284785.s029.tif]

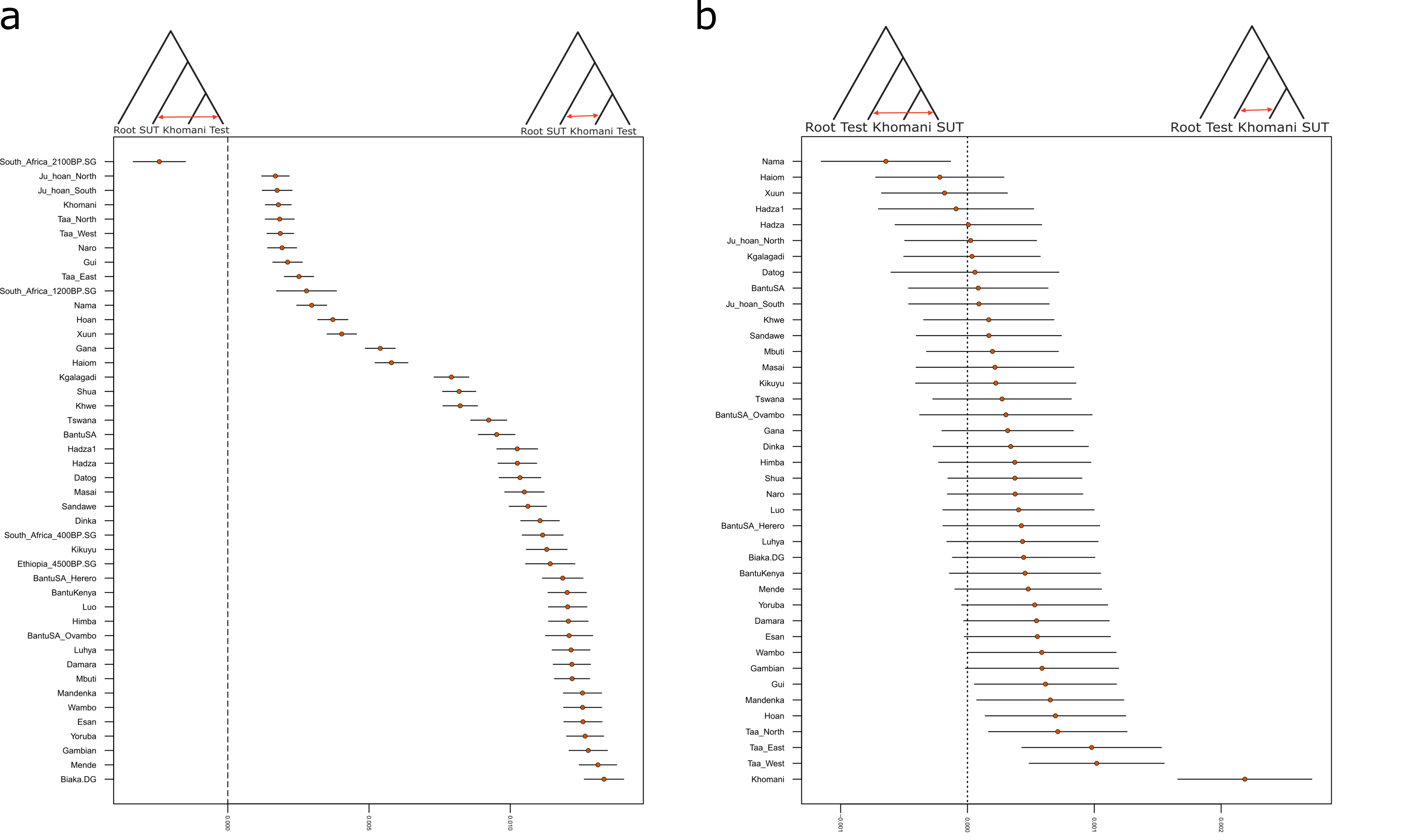

Supplement: S30 Fig — a) f4 statistic of the form f4 (Outgroup, Sutherland; Khomani_San.DG, Test), calculated on 600,000 SNPs of the Human Origins panel. The test population iterates through 44 ancient and present-day sub-Saharan African populations. b) f4 statistic of the form f4 (Outgroup, Test; Khomani_San.DG, Sutherland), calculated on 600,000 SNPs of the human origins panel. The test population iterates through 43 present-day sub-Saharan African populations. (TIF) [file pone.0284785.s030.tif]

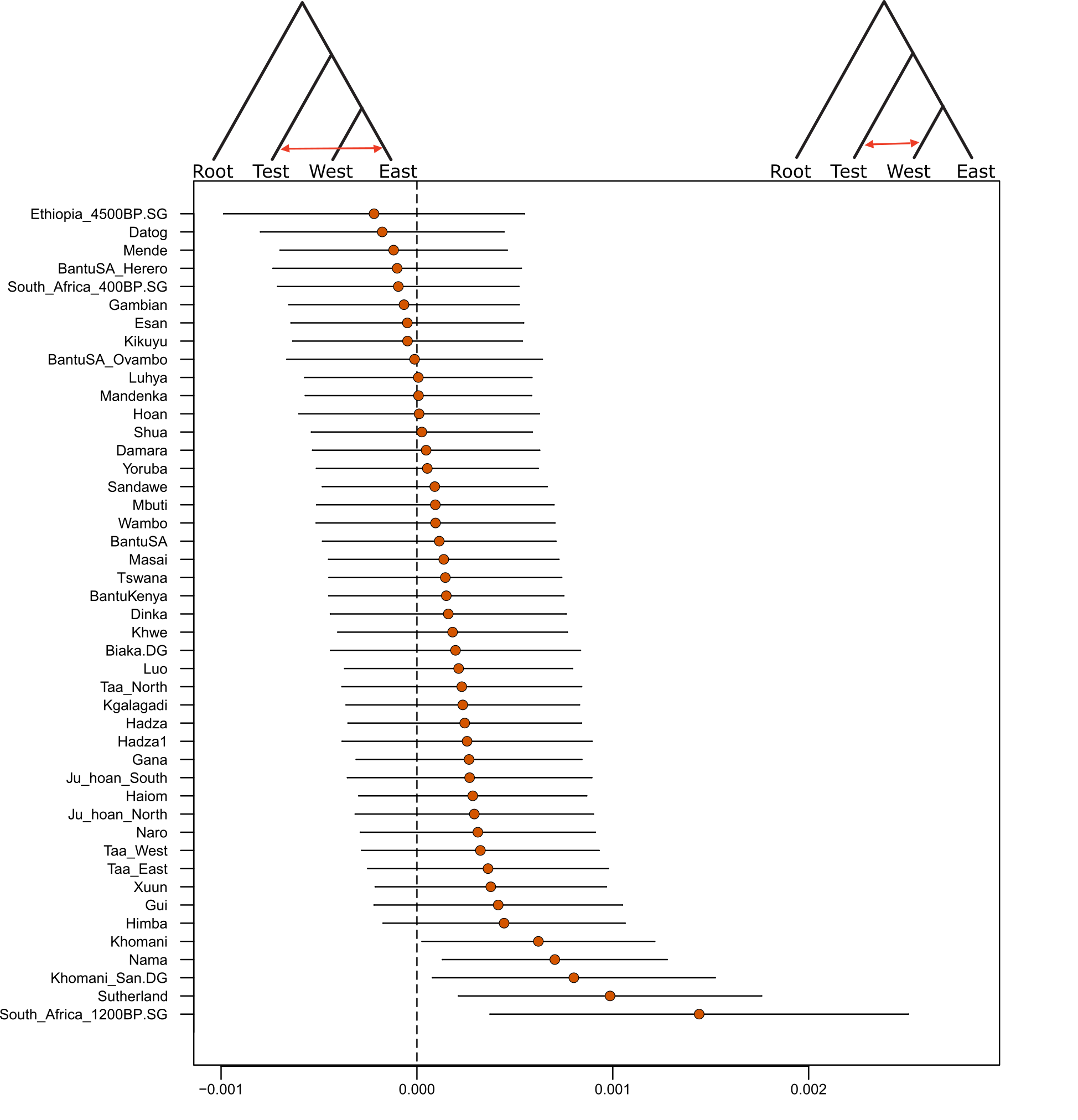

Supplement: S31 Fig — The test population iterates through 46 ancient and present-day sub-Saharan African populations. (TIF) [file pone.0284785.s031.tif]

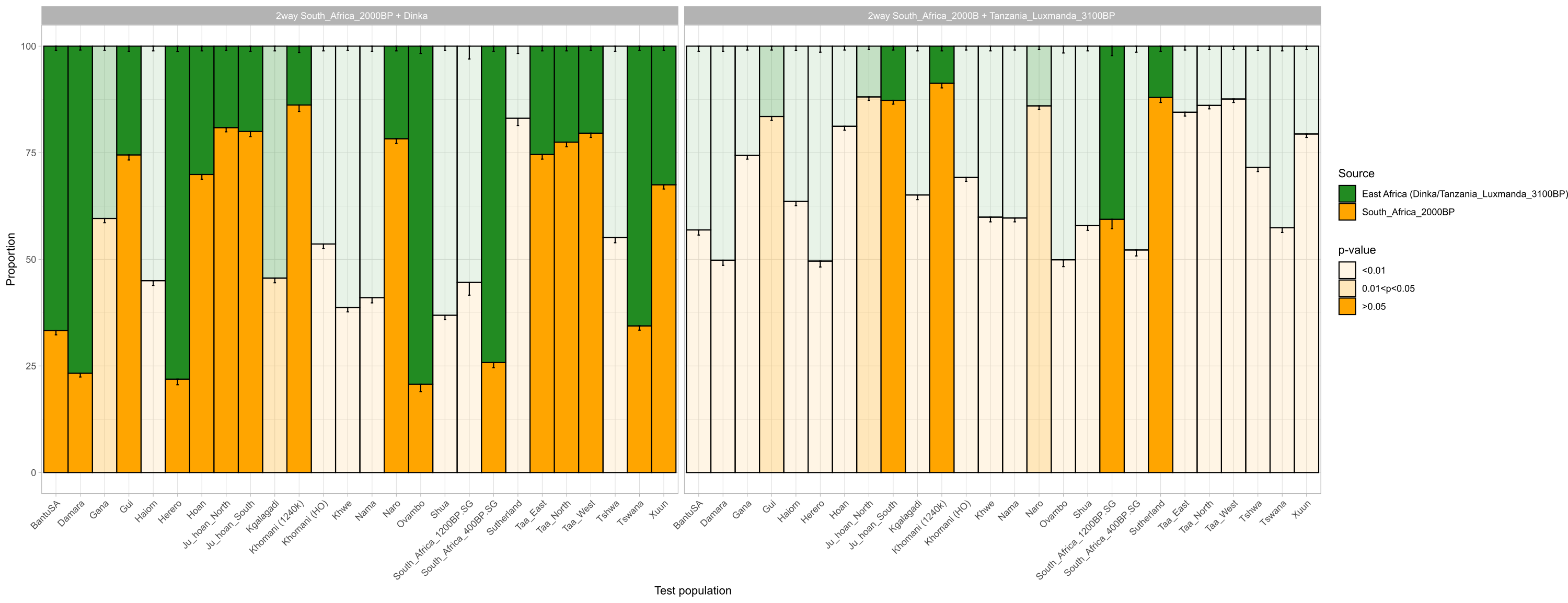

Supplement: S32 Fig — (TIF) [file pone.0284785.s032.tif]

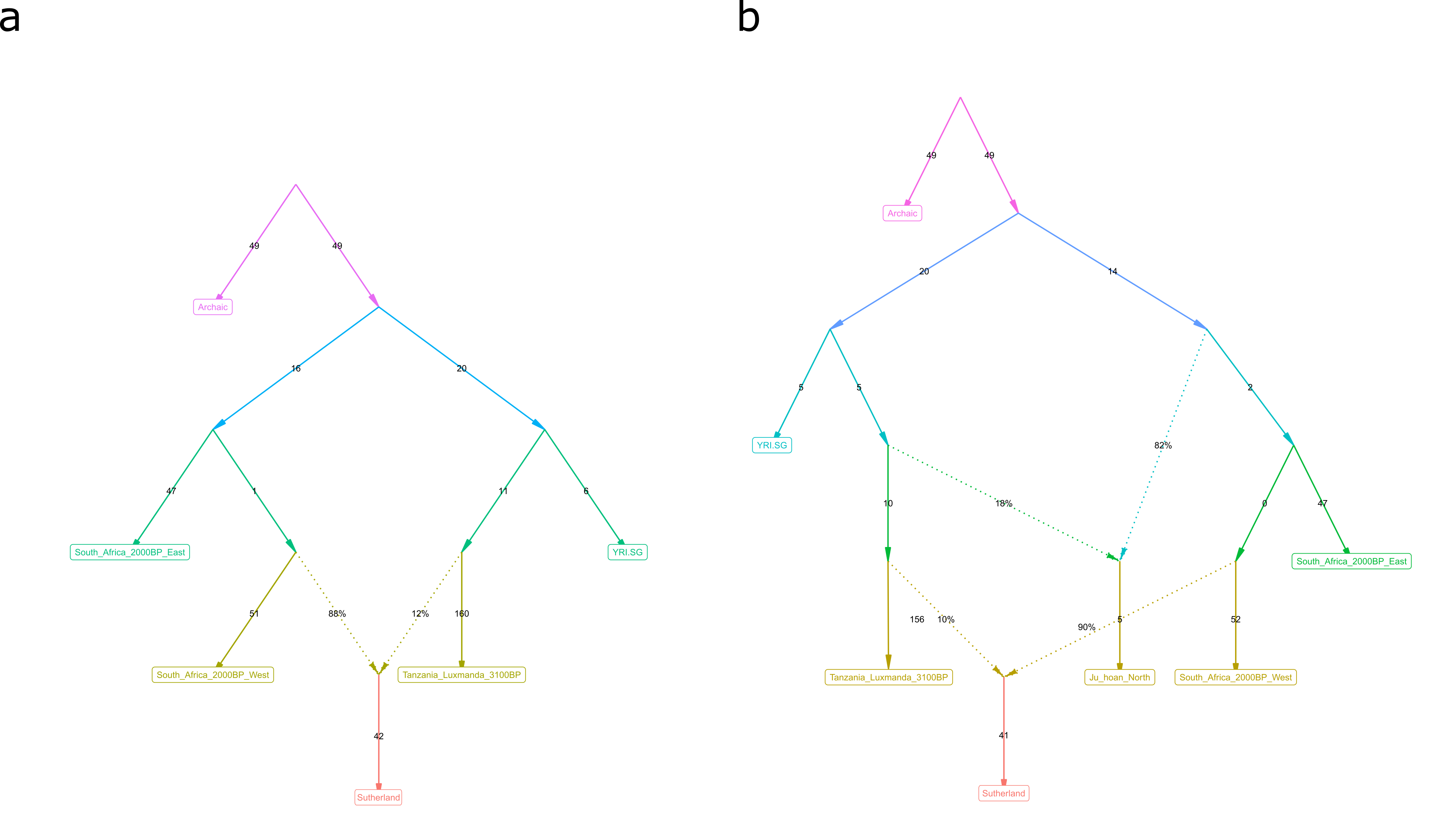

Supplement: S33 Fig — a) Admixture graph of six selected present-day and ancient sub-Saharan African populations from the 1240k panel allowing for one admixture event. b) Admixture graph of seven selected present-day and ancient sub-Saharan African populations from the human origins panel, allowing for two admixture events. (TIF) [file pone.0284785.s033.tif]

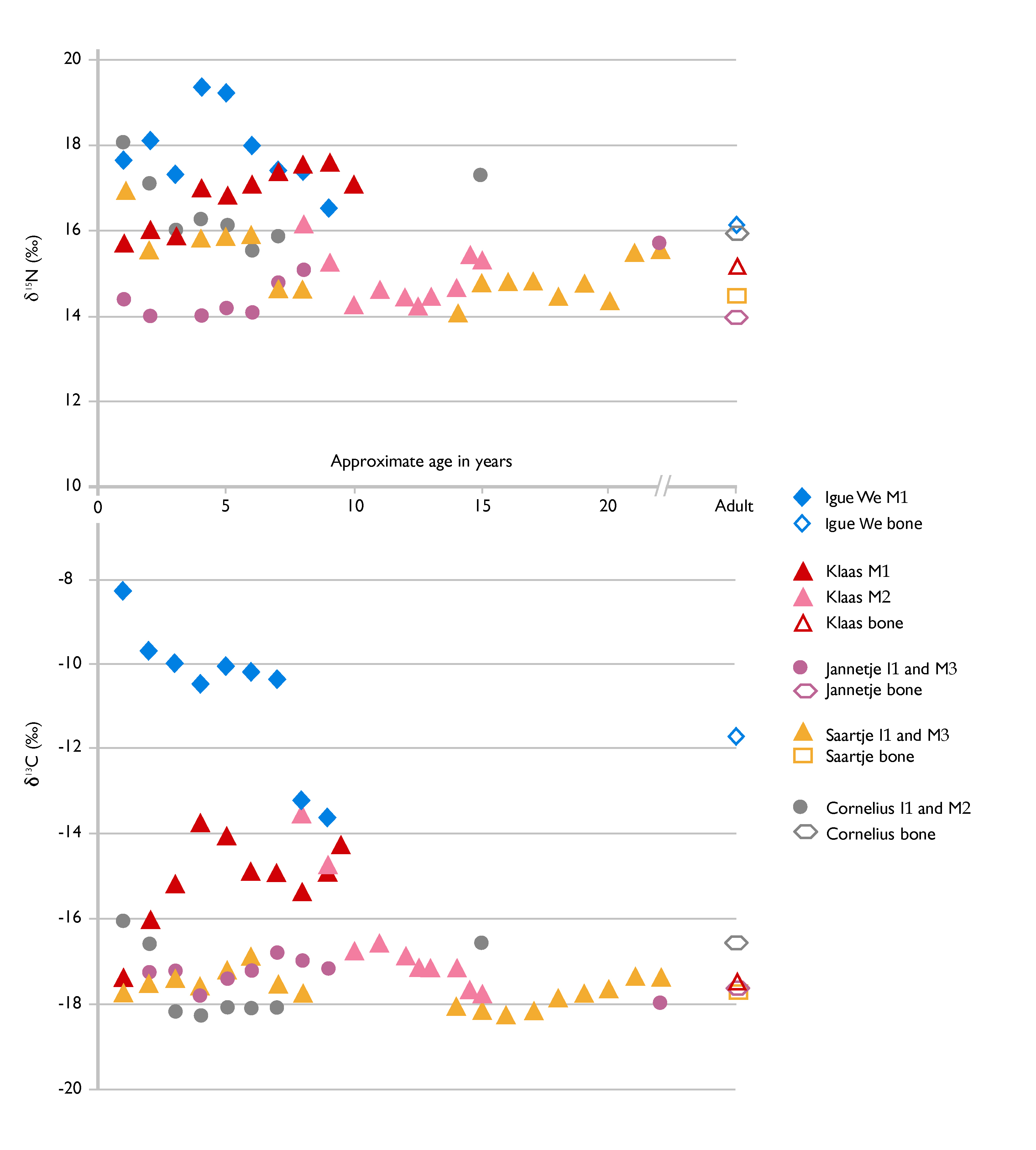

Supplement: S34 Fig — (JPG) [file pone.0284785.s034.jpg]
