# Supplementary material for: Confronting historical legacies of biological anthropology in South Africa—Restitution, redress and community-centered science: The Sutherland Nine
Source: PLoS One. 2023 May 24;18(5):e0284785. doi: 10.1371/journal.pone.0284785 (PMC10208512; doi:10.1371/journal.pone.0284785)
Supplement: S1 File — (DOCX) [file pone.0284785.s035.docx]

**Supplementary Information for**

Confronting historical legacies of biological anthropology in South Africa - Restitution, redress and community-centered science: the Sutherland Nine.

**This PDF file includes:**

Supplementary information text

Figures S1 to S34

Tables S1 to S1

Legends for Datasets S1 to S2

SI References

# S1. Public participation process

The University of Cape Town (UCT) and the nine individuals whose remains were unethically obtained from Sutherland and accessioned into the Division of Clinical Anatomy and Biological Anthropology in the Department of Human Biology bear testimony to the research once conducted on San and Khoekhoe people in South Africa. The university recognized that a process of atonement, restitution [1] and if desired by the descendants, reburial was necessary not only to bring justice and dignity for these individuals, but also to give effect to a broader process of transformation and decolonization, which seeks to undo the injustices of the past.

## S1.1 National framework and theory informing the restitution process

The university was guided in this process by the South African Constitution (1996) and specifically the mandates of Chapter 2, the Bill of Rights as well as the Promotion of Administrative Justice Act, 2000 (Act 3 of 2000), the National Heritage Resources Act, 1999 (Act 25 of 1999) (hereafter NHRA) see S1 Table. Section 10 of the Bill of Rights states that “everyone has inherent dignity and the right to have their dignity respected and protected. The treatment of the Sutherland Nine was not only unjust, but also impaired their dignity and that of their descendants. The decision to engage in restitution for these nine individuals was under the principles of redress and an attempt to restore dignity.

The question at the time was what process guides the restitution and restoration of dignity in respect of unethically obtained human remains? The purpose of the NHRA as set out in its preamble is “….to promote good management of the national estate, and to enable and encourage communities to nurture and conserve their legacy so that it may be bequeathed to future generations…” and also clearly states that heritage contributes to redressing past inequities. Section 3(2)(g)(vi) makes it clear that human remains that fall outside of the National Health Act, 2003 (Act 61 of 2003) form part of the National Estate. As such, the NHRA applied to this process. The challenge, however, was that at the time, no clear process for reburial of remains that fall within the mandate of the NHRA had been established. Subsequently, such a process has been finalized by way of a policy currently in draft format, *Draft National Policy on the Repatriation and Restitution of Human Remains and Heritage Objects.* The UCT process in the absence of this policy was guided by the permit regulations of the South African Heritage Resources Agency (SAHRA) Permit Regulations [GoN 668, G. 27759 (c.i.o 8 July 2005)]). SAHRA is a statutory organisation established in terms of the NHRA as the national body responsible for the protection of South Africa’s cultural heritage. The regulations do not provide for reburial of human remains, only for the disturbance of burial grounds that fall under the NHRA. In the absence of a clearly defined legal process the University followed the public participation process established in terms of the regulations.

UCT decided to recruit an experienced public participation consultant with knowledge of San and Khoekhoe cultural practices This was important given the nature of the process, which required a level of sensitivity that foregrounded the needs of the families, whilst balancing the intentions of the university. The public participation consultant (Ms Doreen Februarie) was guided by existing national regulations (above) and ensured that, over and above these regulations, the families’ requests were adhered to. To achieve a more expansive engagement leading to a more meaningful community engagement process, it was necessary to move beyond the existing national regulations. This process is more costly, requires agility in terms of time frames and relies heavily on expert conflict management and negotiation skills [2-3].

The public participation process began in September 2018 and a final report with a broad reburial plan was submitted to SAHRA in May 2019 with an application for a reburial permit. New claimant groups from a different province came forward in April 2019, at the end of the initial public participation process and submission of the application for the reburial permit. SAHRA provided a written response to the permit application in August 2019, stating that no permit would be required as the choice of the final resting place was in a municipal cemetery. However, SAHRA officially informed UCT of an objection to the process raised by this new claimant group and requested that UCT delay the reburial until the objection was resolved. This was the beginning of a further series of engagements in collaboration with the Department of Sports, Arts and Culture, Republic of South Africa to try to address these concerns and bring closure to the process.

The location for reburial is contested despite archaeological and scientific evidence, as well as oral history clarifying where the remains were unearthed. On February 25, 2020, in a mediated meeting with leaders from the various family groups, a single family member was identified to represent all descendant families in engagement with the university and an agreement reached that reburial would take place in Sutherland. With the arrival of Covid-19 the process came to a halt, and reengagement in the process occurred in January 2021. However, the final resting place continued to be a matter of contestation. Despite multiple attempts to resolve the dispute and move forward in the process, the university was informed in October 2021 that the contestation had been escalated to the President of the Republic of South Africa. The university was asked in January 2022 to host a fifth and hopefully final mediated and externally facilitated discussion to allow the family leaders an opportunity to reach a peaceful compromise on the final phase of the process for the reburial. This was done, with UCT, the National Department of Sports Arts and Culture and the Northern Cape Government collaborating in hosting the meeting. Despite the national requirements for a public participation process having been met and concluded, reburial for the Sutherland Nine was postponed due to ongoing disagreemente among claimant groups. At the time of completing this paper (May 2023) after a final phase of external moderation and assessment of the impasse a decision was provided by the National Minister for the Department of Sports Arts and Culture to rebury all Nine in Sutherland, however, no date has been set.

## S1.2 Theoretical frameworks that informed the public participation process

Public participation processes are informed by sociological models of community engagement. The purpose is to achieve consultation that results in decisions that are informed and led by the community. Pivotal aspects that informed the Sutherland reburial process were redress, authentic engagement, ethical practices, facilitating the ownership and ceding of the project to the families [4-6]. The Aitken [6] community engagement model, consisting of awareness raising, consultation and empowerment, was a particularly useful framework which, together with the Vermillion Accord agreement [7], shaped the process of engagement.

### S1.2.1 Phase 1: Awareness Raising

Phase 1 focused on stakeholder mapping and consultation with identified leadership in the community, government, and families. It began with broad consultation involving individual leaders of the community, community structures (NGOs, local government, community forums, religious leaders, cultural and Indigenous leadership) and the Sutherland community at large. The purpose of this broad consultation was to create awareness, gather information, establish trust and willingness to participate [6: pp.12]. This broader consultation process identified important stakeholders in the community and in local government who might have been missed in a narrower approach [6: pp.14]. For example, a female elder in the community was able to share oral history about the ancestors of the families who lived on the farm.

In this phase the public participation consultant was able to cultivate the “social condition” [4: pp.450] that enabled the families’ leadership in the Sutherland process. Quite quickly the consultant recognised the families’ agency and interest in reconnecting with the unethically procured human remains. In the initial meeting the consultant felt that the families were able to deepen their own understanding of memories and connections to the ancestors as they recalled family stories and historical memories. The families consisted of different generations, with individuals spanning an age range from 16 to 78 years old. Each family member was present to consider and contribute to the process. The agency of the families was formed and all stakeholders, including the university, remained on the periphery of decision-making. The families therefore directed key actions on the Sutherland process from Phase 1, which resulted in the implementation of Phase 2 of the public participation process.

### S1.2.2 Phase 2: Consultation

Phase 2 emphasized the families’ voice and agency to follow their direction. Drawing on the principles of the Vermillion agreement [7], the work of knowledge production and sharing began with the families leading on decisions regarding the processes that were tabled for their consideration. Broad consultation processes continued in Phase 2 as media outlets including national and local newspapers were used to raise public awareness and maximize stakeholder inclusion. These engagements further enhanced trust among stakeholder groups and leaders of the Sutherland community. The families and community requested research on the origin of the human remains, biological information, cause-of-death and facial reconstruction of the human remains. In addition, the families wanted to learn more about the cultural habits and lifestyles of their ancestors and lastly, they wanted the knowledge generated to be shared with the Sutherland community, resulting in a better understanding of the history of the Indigenous people of Sutherland. The families’ leadership in the articulation of the process is understood by researchers to be a significant outcome of a public participation consultation process [4]. It was apparent that the role of the families evolved in each phase with their voice and agency foregrounding the consultation and knowledge production processes. Phase 3 concluded the consultation process, which focused on the families’ intentions for the reburial. By this point the families were clearly the owners of the process, providing direction on the process and the reburial.

### S1.2.3. Engagements with Indigenous leadership

These phases had specific foci and were interdependent and fluid engagements with Indigenous leadership and structures formed a significant aspect of the public participation process. As part of the process of renaming a central building on its campus, the Jameson Memorial Hall to Sarah Baartman Hall, UCT had already extensively engaged with local Indigenous leaders and structures and through its Centre for African Studies (CAS) established ongoing dialogues with a formation of these leadership groups and structures, the A/Xarra Restorative Justice Forum (Forum). The Forum was instrumental in guiding the cultural and restorative process in respect of the human remains.

Engagements included consultation to guide the preparations for the reburial process and to sensitively consider a culturally appropriate approach for the descendent families. Preparation for the reburial commenced with an ancestral blessing ceremony at UCT, facilitated by a Forum of Indigenous leaders with the families’ elders. Indigenous leaders were then introduced to the Sutherland community through facilitated cultural exchange programs as some members of the families wished to learn more about their ancestors. Khoekhoe cultural rituals involving herbs, food, sacred scriptures (commonly known as rock paintings), and more were shared, and opportunities were created to address questions the community had in preparation for the reburial. Following the scientific analysis and confirmation of San lineage, spiritual proceedings were led by San leadership in the Northern Cape Province at the grave sites from which the remains were removed, including sacred offerings to honor their ancestors and restore peace for their souls in preparation for the reburial.

# S2. Actioning an ethical approach through the lens of redress

At the start of the process, there were no established procedures at UCT for applying for ethical approval for these types of activities. To avoid reproducing historical research legacies where information was sourced from “subjects” who received little or no benefit and/or engagement for their contribution to research processes, a different approach was required. Authentic redress required that the Sutherland families’ agency and voice did not sit alongside the research process, but that they had a direct and equal role in the research as well as access to the benefits of that research.

Through consultation and drawing from documents such as the San Code of Research Ethics, a pathway was developed for UCT to broaden the informed consent and ethics process to ensure the families were foregrounded. To avoid members of the families feeling pressured by UCT into giving permission for study, the consultation process was led by the public participation consultant mentioned earlier. Informed consent was not a once-off procedure, but rather a process that involved three initial meetings with the descendant community. Information was shared to ensure there was awareness of previous historical research that studied San and Khoekhoe people, and how (where relevant) communities/individuals had previously been exploited in the name of science. For the families to fully understand the parameters of consent, it was necessary to explore how the research would benefit the researcher(s) and the university as an institution; and how the research could result in both benefit and possible harm. The series of three meetings allowed time for reflection and possible re-consideration between visits. Members of the scientific team were present on two visits, to explain what kinds of information could be obtained, and answer questions. On one visit, no scientific team members were present, in case their presence might be perceived as coercive. The objective of these engagements with the families was to ensure that, beyond the traditional informed consent and ethics process, there was an intentional focus on redress. The families were recognized as custodians and co-producers of the knowledge. Only when the families felt well informed did they sign the informed consent, along with writing in their own words what they wanted done during the research process, including any conditions. In this way the formal submission by the descendants ensured that the university could honor the wishes of the Sutherland descendants as equal contributors and the custodians of the knowledge in the research process. The explicit declarations that occurred during the informed consent process were an important element of redress, which ensured that the Sutherland families could make key changes to research processes based on the anticipated benefits that would be experienced by the university, researchers and third parties. Informed consent was obtained for all aspects of this study, as well as permission from the University of Cape Town Faculty of Health Sciences Human Research Ethics Committee for analysis of tissue samples (HREC# 715/2017).

A materials transfer agreement was established and implemented for the *s*amples for DNA analysis, which were sent to the Max Planck Institute for the Science of Human History in Jena, Germany. Permission from the Human Ethics Research Committee at the University of Cape Town and informed consent from the families were obtained before commencing with the DNA project. Any tissue that remained after the DNA analyses has been returned to UCT and reunited with the rest of the remains. To ensure protection of the Sutherland families, the procedures for obtaining ethics approval were in accordance with the description in Gibbon (8), which go above and beyond the current legislative requirements.

The informed consent in this restitution process was not an event but is and continues to be a process on its own, guided by both the stakeholder community and the university. For this publication there was discussion with the families regarding the timing of manuscript submission, and aspects such as them being authors or acknowledged. To make these decisions, several discussions were held explaining the academic and publishing process, as well as the requirements of authorship versus acknowledgment. The risks and responsibilities for descendant communities being named in scholarly research were discussed. The outcome of these discussions was that the families requested acknowledgment rather than co-authorship.

## S2.1 Ethics in the process of filming and photography

The media team that documented this process consisted of a photographer, videographer, and media liaison person. They were present throughout the public participation process to capture significant moments, including the families’ visit to the grave sites at Kruisrivier, visits to potential reburial sites, the workshops with Indigenous leaders educating local primary and high school learners about Indigenous traditions, pivotal moments in the ceremonial blessings and the presentation to the families of the reconstructions of the faces of the deceased by the forensic artist.

The UCT does not have a Code of Conduct that guides processes of documentation through film and media. However, the media team needed to ensure that the process was transparent and inclusive especially for the families. The media team was guided by the San Code of Research Ethics, which emphasizes the need for an ethic of fairness, care, respect and honesty.

At the beginning of the process, the media team discussed and highlighted the need for consent from the community and the families as to which parts of the proceedings should be filmed or recorded, and the importance of doing this in as non-intrusive a manner as possible. For example, to the families and community in general, seeing cameras may seem exciting, however the University was aware of historical exploitative filming practices, and therefore the media team applied sensitivity and caution when documenting the process. In addition, while moments such as the unveiling of the facial reconstructions brought joy and amazement, they also triggered a sense of loss and pain. It was important to document these moments with care and respect. The photographer chose, for example, to keep her camera equipment to a minimum and most of the time used the same lens, to spend more time looking rather than changing lenses or setting up lighting.

While thorough photographic documentation was important to create an archive of the process, film and photography involves real people and sensitivity to who is photographed, and how, is of key importance. At least one member of the media team came from an Indigenous community and was able to create a connection with her own lived experience and heritage, thus guiding the process to ensure the necessary care and integrity in the ethics of documentation. She notes: “It was under a starry sky in front of an open fire when elders told their stories, laughed, ate together, and played a few rounds of dominoes. It was these human encounters behind the scenes, moments that allowed me the freedom to photograph them with a connection from both sides and mutual respect for the process.”

## S2.2 Reflection on redress

## S2.2.1 Reflections on redress through ethics

Through the broad informed consent approach and deepening the ethics of care, UCT was able to achieve the following: Active agency of participants as contributors to the research process*-* *e.g.* research participants could contribute to the writing process, if desired, and/or be acknowledged in all publications; Expanded informed consent - Declaring how the university and/or the researcher(s) benefit from the knowledge acquired from the community/research participants is an important element of redress. Initially, the participants may not have been fully aware of how researchers and institutions benefit from knowledge production, (*e.g.* *ad hominem* promotion; publications; government subsidies; professional and reputational gain). There may be financial gain for researcher/s and the University through book royalties or other forms of direct financial gain due to the research production. Declaring these forms of direct and indirect gain provides the community/research participants with knowledge about the academic process. The families had an opportunity to share their own views on these gains and if possible, think about how they too wish to gain from this process. Some examples that emerged included the families being recipients of any royalties from book sales, perhaps as part of a scholarship fund for the community. The families also requested that their community be acknowledged in all related publications in perpetuity. Finally, they requested the continued commitment of the University towards educational outreach in the Sutherland community beyond the reburial.

University Redress - Declaring the university’s purpose in addressing its complicity in unethical processes was an important part of the informed consent process. The university’s position was that if it did not initiate the work of redress, it would remain complicit in continuing the injustices of the past. The institution understood that it had an ethical duty to attempt to mitigate its past actions and engaging with the family on its wishes in the restitution process became an important part of the process.

Family agency - the families’ response to the broadened informed consent process was that they had not known or understood all this information prior to this series of engagements. The extended informed consent process empowered them to request the university to comply with their wishes. For example, the families requested acknowledgement in publications, as opposed to the request from the university to co-author research outputs. They also sent to the Human Ethics Research Committee at the University of Cape Town a document they prepared in their own words, which articulated how they would like to be acknowledged/represented in publications and research. Actioning an ethical approach through the lens of redress was a pivotal part of this process, as it shifted the power and agency back to the Sutherland families as custodians and beneficiaries of their knowledge and positioned members of the university as co-contributors to the knowledge generation process.

## S2.2.2 Reflections on redress as part of the public participation and consultation process

A process that initially appeared straightforward and was anticipated to be resolved within a year became laborious, onerous, cumbersome and at times exhausting. The existing draft legislation and policies did not provide guidance on how to resolve the dispute about the location of reburial, which led to a longer, more drawn-out process. This has not only impacted all those involved emotionally, but the political and financial consequences and publicity surrounding the process have had a negative impact on restitution processes in South Africa in general.

The fact that, more than four years after the initial approach to the Sutherland community, the reburial has still not taken place has had a deep impact on the families and descendant communities. It has been emotional; they have grown weary and anxious about the reburial. The university has been at pains to mitigate the impact on the families whilst balancing the needs of declared interest groups and the requests by government. University stakeholders have been personally and professionally affected by the impact of disparaging media articles, have been on the receiving end of negative commentary in academic seminars and aggressive negative conversations and discussions as part of the process. The repeated extensions of the public participation process, and requirement for further consultations, have been draining. If the dispute cannot be resolved through continued mediation, government will need to chart a way forward in the process. Until finalization of the restitution process, we continue our commitment to seek justice and closure for the Sutherland Nine and their descendants.

Upon reflection, lessons have been learned through this process that have influenced the development of national, provincial, and institutional level policies. This process and others in South Africa led Black, Gibbon and Omar [9] to prepare a set of guidelines for restitution processes to assist others in the future.

# S3. Historical and archaeological background

## S3.1 C.G. Coetzee the donor & medical student

Some way into the Sutherland restitution process, investigations by Esterhuyse into the Kruisrivier farm and the Coetzee family revealed that the C.G. Coetzee who donated the Sutherland skeletons to UCT had been a medical doctor. This led to a search in the records of degrees conferred by the Faculty of Health Sciences at UCT. A student record was found for a Carl Gert Coetzee of Kruisrivier farm, Sutherland, showing that he was a registered medical student at UCT between 1925 and 1931 C.E., which include the years of donation (Fig. S1).

The years of donation coincide with years in the medical degree program in which students take courses in anatomy and physiology. The donor would have known the then Department of Anatomy and its Head, Prof M.R. Drennan (Head of Department from 1919-1955 C.E.) [10]. Drennan established the human skeletal repository at UCT and many documents in his handwriting exist in the associated records. The archival records for the Sutherland Nine individuals (summarized in the main text) are in his handwriting, indicating that he received and may have even solicited these remains; obtaining skeletons of San and/or Khoekhoe individuals would have aligned with the goals of building collections of skeletal remains at that time. Coetzee was a mature student in his thirties and had previously been a schoolteacher, which may have led him to have a different relationship with his lecturers compared with most of his classmates, and perhaps more inclined to assist Drennan. Drennan’s acceptance of the Sutherland Nine and recording of information about their lives shows he was aware of their origin and that he did not find this problematic. Both Drennan and Coetzee were known to be Christians; a religion that does not condone the desecration of graves. Neither the donor nor Drennan apparently saw the need to accord these nine individuals the respect they would have accorded members of their own communities. The donation of these nine people was accompanied by the bones of baboons and a lion.

## S3.2 Roggeveld history

The wide interior region of the Karoo, a San and/or Khoekhoe word which translates as ‘land of thirst’, receives annual precipitation of only 50-250 mm, resulting in a diverse semi-desert vegetation dominated by low scrub and succulents. The Roggeveld (‘rog’ is the Dutch word for rye) escarpment receives somewhat more rain than surrounding areas due to its higher elevation [11]. This underpins the desirability of this part of the landscape for 18th century colonial stock farmers (trekboers). The line of the escarpment from Namaqualand in the north-west, through the Hantam and Roggeveld Mountains to the Nieuweveld Mountains in the south-east is also a climatic frontier between the winter rainfall region to the south and west and the summer rainfall region to the north and east (main text Fig 1).

The linguistic layering evidenced in the contemporary use of English, Dutch and San and/or Khoekhoe place names is the result of cultural replacement, erasure, and entanglement, a process through which the Roggeveld became one of the most blood-stained colonial frontiers in South African history. The Roggeveld was fiercely contested, as San and Khoekhoe people fought to prevent the invasion of their territory by semi-nomadic colonial farmers, with thousands of San and Khoekhoe people killed for their resistance [12-15]. The colonists appropriated livestock, took land and, as the Kruisrivier archival records show, captured people to work as farm laborers [14]. San and Khoekhoe people waged an effective and continuous war of resistance along the line of the Roggeveld through the second half of the 18th century and were sometimes joined by armed fugitives from the colony, of mixed Khoekhoe and slave descent, or people of European descent. Throughout the 18th and 19th centuries, repeated epidemics of diseases such as smallpox had a major impact on the colony. San and Khoekhoe populations in adjacent areas were especially vulnerable [16], suffering massive social and cultural disruption, similar to the description in Diamond [17].

This dry landscape (‘Karoo’ means ‘land of thirst’) required foragers and Khoekhoe herding communities to be highly mobile, with stock-herders in particular pursuing transhumant patterns of landscape utilisation to seek out the best water and grazing. These settlement patterns were based on a deep knowledge of ecological and climatic structure premised on a long precolonial experience of this landscape over thousands of years before European pastoralists arrived in 1740 C.E. [13, 14]. Colonial pastoralists acknowledged this experience and adopted the same transhumant strategies; they continued to follow ancient patterns of transhumance well into the twentieth century. During the Dutch colonial period, incoming farmers of European descent laid claim to the places they regularly used as loan farms or *legplaatsen*, for which no rent was paid although rights of ownership were exerted. The Nieuweveld region (main text Fig 1) was one of the last areas to be occupied by colonial farmers due to its particularly harsh environment, and therefore became an area of retreat for San and Khoekhoe resistors. Throughout the second half of the eighteenth century the combination of a harsh natural environment together with strong San and Khoekhoe resistance gave the region its character of being considered outside colonial control.

Competition over resources meant progressive displacement and dispossession for San and Khoekhoe people, and from the 1750s C.E. onwards, there were continuous reports of San theft of the colonists’ livestock and killing of workers [13, 18]. These attacks heralded the beginning of a protracted guerrilla war waged by the San, and sometimes the Khoekhoe, against colonists, both people and their livestock. In response, the farmers sent out commandos to try to reclaim stolen cattle and sheep and kill the men, while women and children were often taken captive and forced into labor. There is documentary evidence that hundreds of San were killed between 1770 and 1774 C.E.; one commando alone, in 1771 C.E., killed 92 San [13,14]. Additionally, at this time, a Khoekhoe rebellion known as the Roggeveld Rebellion demonstrated that even Khoekhoe on colonial farms did not feel safe from the vengeance of commandos [13, 18].

The response of the authorities at the Cape (the Dutch East India Company) to this crisis was to launch the General Commando of 1774 C.E. to crush San and Khoekhoe resistance throughout the length and breadth of the northern frontier zone. A force of 250 colonists killed over 400 men, women and children (with the loss of only one commando member, who was killed by a poisoned arrow), and took over 200 women and children captive. Mass graves on the farms “Oorlogskloof'' and “Gunsfontein”, near Kruisrivier, are an indication that mass killings occurred. However, the General Commando did not end San and Khoekhoe resistance. By the 1790s C.E. there were reports of mixed San and Khoekhoe groups of more than 1000 members in the Nieuweveld and Koup (main text Fig 1), which were strong enough to beat off the commandos sent after them.

In addition to brute force, an attempt to ‘civilize’ San people arose out of a revival of Christian evangelism in the 1790s C.E., following the British occupation of the Cape in 1795 C.E. Missions such as the London Mission Society’s Sak River Mission (main text Fig 1) were a failure because the San were not prepared to ‘settle down’ and receive the Christian message. The British government encouraged the colonists to make peace with San people by giving them gifts. There were sporadic periods of calm during which farmers advanced into San territory, after which hostilities were renewed. However, a British peace initiative, in which San people were seduced by grants of livestock, fatally weakened their resistance. By dropping their guard, they allowed the farmers into their midst.

In the late 18th and early 19th centuries, independent San, fugitives and refugees continued to hold out undetected in the remote Nieuweveld region (main text Fig 1) but they were physically marginalized, and many were starving. Those who stole sheep to survive were either shot or arrested [18]. In this way the new town of Beaufort West became a place of imprisonment for San convicts who were later dispatched to Cape Town as labourers on public works. As the frontier moved further to the north and east the Roggeveld farmers were relieved of the constant need for commando duty and concentrated on the demands of farming. Although the Roggeveld was pacified by the 1820s C.E., San resistance continued in regions to the north well into the nineteenth century [14,19,20], and colonists reported that hundreds of San people were being killed by commandos whilst the condition of San and Khoekhoe farm labourers remained dire. Despite this tragic history, there has been a degree of San and Khoekhoe cultural continuity into the present time [21-23].

In these circumstances the history of the Roggeveld in the early nineteenth century was largely the history of a remote farming community dominated by agricultural concerns. For our purposes, given the testimony of the human remains from Kruisrivier farm, the most important issue to explore must surely be the nature of the relationships that existed between farm owners and their laborer’s.

## S3.3 Kruisrivier farm

The first record of the farm Kruisrivier (32°19'34.0"S, 20°22'40.0"E) in the Roggeveld was its grant as a loan farm in 1749 C.E., in accordance with the system of land tenure under the Dutch administration. By 1770 C.E., there were some 221 loan farms in the Roggeveld and 75 in the neighboring district of the Nieuweveld [13]. A significant change in the land tenure system was made after the British took control of the Cape in 1795 C.E.. Farmers became formal landowners, holding title deeds and able to bequeath properties to their heirs.

It is probable that a Coetzee ancestor was the first owner of Kruisrivier. Under the British system, Kruisrivier was surveyed “...for G. Coetse [and] C. Zoon by (Sgd.) C.L. Stretch” in 1832 C.E. and a title deed was granted in 1838 C.E. Information from the archival records suggests that a Coetzee must have been in possession of the farm in the first half of the 19th century, because the great-grandfather of C.G. Coetzee (the donor) captured Klaas Stuurman as a child, somewhere between Sutherland and Carnarvon (main text Fig 1). This is not surprising, given that the area to the north and east of the Roggeveld remained remote but open for the capture of San and Khoekhoe people well into the 19th century [14,19].

This process of capture and indenture as farm labour involved San and Khoekhoe people being renamed by landowners. Even today in South Africa, many domestic workers have nicknames or choose names that are Europeanised, and thus easy for employers to use, in addition to their given names in their own languages. The practice of giving Dutch names to /Xam and San informants in the Bleek and Lloyd records is discussed in depth in Bank [18]. This is likely to have been the case with Klaas Stuurman, Cornelius Abraham and the other named Kruisrivier farm workers. It is probable that they also had San or Khoekhoe birth names. The choice of European names may have been arbitrary, but the archival records show that nearly all the Kruisrivier names are Dutch, linked to Christianity, and some are names prominent within the Coetzee family, such as Klaas and Cornelius. Klaas was a very common name at this time [18]. The surname Stuurman is a well-known South African name derived from the Dutch word “helmsman” and in the colonial Cape was applied to men who steered boats or drove ox-wagons [24]. In the 18th and 19th centuries the name was found in Middle Orange River, Namaqualand, Eastern Cape and the Griqua territories of Transorania [25-29]. Similarly, the surname Abraham is today shared by many South Africans across the country.

Kruisrivier was a regionally significant centre. In 1841 C.E., residents petitioned the authorities for a church to be built on the farm; this appeal was unsuccessful, and the regional church was built on its present site in Sutherland in 1857 C.E. A farm school was established and serviced by a half a dozen teachers over more than two decades (S2 Fig).

Later in the 19th century, Kruisrivier was subdivided into northern and southern portions of approximately equal size (S2 Fig). The southern section was owned by Carel Gert Coetzee (senior), whose son, also Carel Gert Coetzee, was the medical student who exhumed the burials in the early 1920s, and later qualified and practised as a doctor in Paarl, near Cape Town. Carel senior was buried in the Coetzee family cemetery in the southern portion of the farm, along with his wife. This cemetery was relocated in the late 1950s C.E. from its original location near the river. The spatially separate cemetery for the Kruisrivier farm laborer’s is also located on the southern portion. The northern section was owned by another Carel Gert Coetzee, the cousin of the owner of the southern section.

## S3.4 Kruisrvier cemetery

The prime objective for mapping the Kruisrivier farm cemetery was to identify the locations of the graves from which the burials were removed. This was an important objective if the farm cemetery was to be considered as a possible place for reburial. A 3D model of the cemetery was created by the Zamani Project [link here](https://sketchfab.com/3d-models/kruisrivier-farm-cemetery-sutherland-2555d2ea178848958dc2154d3f3a9f66).

The laborer’s cemetery was located 300 meters south-west of the main Kruisrivier farm complex on a flat alluvial terrace (S2 Fig). The location was deliberately distanced from the main farm buildings, or any domestic dwelling at the time when it was first used in the 19^th^ century. There are small ash heaps within 100 meters of the cemetery that are associated with the ruins of small single room structures that were probably farm worker dwellings. The broken ceramics in these middens, however, appear to be of 20^th^ century date and postdate the probable 1880s C.E. establishment of the cemetery and possibly the 1925-1927 C.E. exhumations. Similar isolation is also true of the small Coetzee family cemetery.

All the graves were marked by low platforms or cairns, built from locally collected stone. A minimum of 36 burials were mapped [30, S3 Fig]. There are two distinct grave styles. Rectangular graves are marked by upright headstones, and some also have smaller footstones (H/F graves). These graves are generally orientated east to west with the larger headstone located at the western end of the grave and are Christian in style. The second style consists of circular or sub-circular stone platforms or cairns without headstones or footstones (C graves). We ascribe a San and/or Khoekhoe identity to these graves, and this is supported ethnographically [31, 32: pp.134, 33: pp. 61, 34: pp. 306-7].

Disturbed graves were identified by platforms that had been unpacked and the stones scattered. There are five positively identified disturbed graves and one probable (the area between graves 16 and 17). This may be where the two children were buried [30]. This preliminary assessment, made in the field, was subsequently confirmed, and refined by manipulation of the 3-D model created by the Zamani Project, which allowed close up oblique views of the topography. The disturbed graves cluster at the northern end of the cemetery where H/F and C graves are intermingled. In contrast, the southern part of the cemetery has only H/F graves (S3 Fig). A minimum of five identified disturbed graves is as expected, given that Igue We came from elsewhere and Voetje was stated to have been buried in the mountain. With the detail currently at hand it is impossible to link individual sets of human remains to specific graves. However, the archival records note that Klaas and Saartje were buried next to each other, and the children between them. The area between graves 16 and 17 is the most disturbed and it is possible that two other graves were located here.

The presence of both H/F and C graves in the northern section and only H/F graves in the southern half (S3 Fig) may indicate that the northern section is older. We know that the exhumed burials date to the 1880s and if the cemetery grew linearly from north to south then the cemetery may have been established only in the late 1870s or early 1880s.

The disturbed graves in the northern part of the cemetery are not contiguous. For example, burial 4 is the only disturbed burial in a line of five other cairn burials (S3 Fig). The inference is that this individual was specifically targeted. The same may be true for burials 10 and 16. Despite being badly disturbed, these graves can be identified as H/F burials because the headstones are still in place, but H/F burials immediately to the north remain fully intact (S3 Fig). When the burials were exhumed in 1926 C.E., the cemetery had expanded, and according to Klaas Coetzee (nephew of Dr C.G. Coetzee), was still in use in 1959 C.E. Given the number of burials in the cemetery, the question as to what informed the selection of specific graves is important. As noted above, we know from the archival records that most of the individuals were named. This information must have been supplied by Carel Gert Coetzee senior, because the donor was born only in 1891 C.E. The choice to exhume specific burials, therefore, was probably made with the help of Carel Gert Coetzee senior, who knew the individuals by name as well as some details of their life histories.

This certainly applied to Klaas Stuurman, who, as noted above, was “caught” earlier in the 19th century by the donor’s great grandfather (C.J. Coetzee) “…between Carnarvon and Sutherland”. We might assume that this and other references to people of San and/or Khoekhoe descent drew attention only to the Indigenous style cairn burials. However, the record of disturbed graves shows that H/F graves were also exhumed, and this suggests that the known biographies of those buried informed the selection process rather than a focus only on graves that stylistically were not Christian.

The archival records also provide descriptions of the structure of some grave shafts. One was a “niche” burial in which the body was laid in a chamber dug into the side wall from the base of the burial shaft. In two other burials, the bodies had been encased in stone slabs within the grave shaft. These grave forms have been described in the ethnography and archaeology of San and Khoekhoe burial practice [20,31: pp.165, 35: pp. 17, 36: pp. 32], and niche burials were also observed in the graveyard of the 19^th^ century mission station of De Tuin near Kenhardt, approximately 400 km north of Kruisrivier [37]. If the structure of three of the grave shafts indicate San and/or Khoekhoe burial practice and, out of the five disturbed burials, three and probably four were H/F burials, this could suggest a cultural mix, in which a publicly visible H/F grave style may have capped a burial expressive of San and/or Khoekhoe belief. This possibility is confirmed by Klaas Coetzee (the nephew of the donor) who, in an interview carried out during this research, described a Christian burial in the Kruisrivier farm cemetery where, in the absence of a coffin, the woman was buried in a traditional *grondgraf* (literally, a ‘soil grave’). This was a niche burial, in which the body was laid in a side chamber, wrapped in either animal skins or a sheet. This burial form was a sign of respect.

Jannetje was buried wearing copper bangles and Cornelius and Saartje had copper stains on their skeletons in positions that suggest they were also buried wearing copper jewelry. These objects, which expressed something of their San and/or Khoekhoe identity in life, were probably worn all the time, and were on their bodies when they died. The exhumations in 1925-27 would have paid little attention to artefacts with the burials, quotidian or otherwise. However, within the soil and rocks of the disturbed cairn burial 4 (S3 Fig), two pieces of milky quartz were noted. Milky quartz is not present in the local geology, so these were collected, possibly from the Fish River gravels one kilometer to the east (S3 Fig), and deliberately placed in the grave. From archaeological and ethnographic sources, we know that quartz, quartz crystals, white seashells and ‘shiny things’ were part of San and Khoekhoe ritual paraphernalia [see 38: pp. 53-65].

In our initial visit to the Kruisrivier cemetery, we also located an archaeological site on the northern portion of the farm (S2 Fig). This site comprises a shallow layer of ash and work there in 2019 recovered pieces of Khoekhoe coarse earthenware ceramic, 18th century Batavian and other Asian porcelain, European stoneware, glass trade beads, beads made from ostrich eggshell, lead shot, freshwater mussel shell and sheep bone food waste [39]. This mix of European and Indigenous material dates to the later 18th century and may mark the central domestic area of Kruisrivier when it was a loan farm. The mix also hints at the complex 18th century interactions between European pastoralists and San and/or Khoekhoe, which resulted in cultural erosion and change. Despite the brutality of dispossession and genocide suffered by the San and/or Khoekhoe people in the area, some of the evidence from the later 19th century Kruisrivier burials indicates that aspects of their cultural practices were still intact.

## S3.5 History and archaeology methodology

Primary sources including archives (university and governmental) and interviews with living descendants were used to compile a historical understanding of this donation. Esterhuyse interviewed Klaas Coetzee, the nephew of the donor, for a family and farm history. Esterhuyse himself grew up on the farm next door to Kruisrivier and conducted several interviews to assist with piecing together events of the past.

The archaeological field survey of the Kruisrivier farm was conducted on 8^th^ December 2018 with the permission of the landowner. The mapping of the cemetery was done by The Zamani Project (Geomatics, UCT) on 24, 25 and 26 June 2019. A 3-D model was generated using laser scanning with a photographic overlay.

# S4. Osteobiography

The Sutherland families requested osteobiographic study of the remains to gain more knowledge about their ancestors and better understand their lived experiences. Results for each person are summarized in Table 1.

## S4.1 Igue We

This individual was not linked directly to Kruisrivier farm, but came from Sutherland, from the same donor at the same time as the other individuals Radiocarbon dating of collagen from the right maxillary second molar yielded a result of 709 ± 23 BP (MAMS-42111). This gives a calibrated age range of 1277-1395 C.E. (99.7% probability, using OxCal version 4.4 and SHCal 20) [40-41], placing him securely in the pre-colonial period. This individual was fragmentary: only the cranium, right humerus, right femur and left tibia were present. This combined with the extreme gracility in the population made it difficult to assess sex based on morphology, as demonstrated in contemporary forensic investigations [42]. However, from the genetic analyses described below, he was male. The cranial sutures and eruption of third molars indicate a young to middle aged adult (30-50 years-of-age). Stature estimation was impossible because of breakage at the distal end of femur. Tooth wear was moderate, with presence of dentine clusters in a horizontal and plane direction. No dental disease was present. The anterior teeth were lost postmortem so that only the maxillary molars were present.

There was perimortem sharp-blunt force trauma with two points of impact on the right side of cranium (S4 Fig). The primary point of impact occurred on the right parietal bone 8 mm posterior to the coronal suture. A primary radiating fracture extends posteriorly from the impact site, turns medially midway across the right parietal bone, crosses the sagittal suture, and terminates just posterior to the left eurion. The second impact site terminates into the primary fracture, with the energy propagating along the coronal suture, running right to left. Halfway along the left coronal suture, a portion of this energy dissipates into the left parietal with production of a primary radiating fracture extending posteriorly. This fracture terminates inferior to the left eurion. Just anterior to the termination point, a smaller secondary fracture extends medially, producing a tertiary fracture just before its termination that extends posteriorly, parallel to the primary fracture. Additional energy continued to propagate along the left coronal suture, dissipating into the left temporal bone with formation of a bisecting secondary fracture (Fig. S4). The injury indicates a heavy object was used with enough force to loosen the temporal and occipital sutures. It is inconsistent with a fall. The impact probably killed him instantly.

## S4.2 G!ae

This was a boy (determined from genetic analyses), aged between 4 and 6 years-at-death based on tooth eruption and epiphyseal fusion. He showed evidence of physiological stress in the form of bilateral Harris lines on his tibiae; two on the distal end consistent with ages 1-3, and three on the proximal end corresponding to ages 1-4. These indicate nutritional stress (commonly from protein and vitamin deficiency) [43] throughout G!ae’s short life. The ages were consistent with the post-weaning period and this evidence indicates a risk factor that likely contributed to his early mortality [44-46]. There were no signs of trauma or active disease on his skeleton.

## S4.2 Saa

This was a girl (determined from genetic analyses), aged between 6 and 8 years-at-death based on tooth eruption and epiphyseal fusion. Linear enamel hypoplasias on the permanent incisors of the maxilla and mandible indicated three separate episodes of physiological stress (severe illness or malnourishment) between the ages of 2 and 4 years. She also showed evidence of physiological stress with bilateral Harris lines on her tibiae and femora; two on the distal tibia consistent with ages 1 and 4-5 respectively, and one on the distal femur corresponding to ages 4-7. These indicate nutritional stress, (commonly from protein and vitamin deficiency) [43]. Together, the linear enamel hypoplasias and Harris lines indicate evidence of nutritional stress caused by inadequate food intake or severe illness - risk factors that likely contributed to her early mortality [44-46]. There were no signs of trauma or active disease on her skeleton.

## S4.3 Cornelius

Cornelius was an adult man aged between 30 and 45 years-at-death. He stood approximately 1.62 m tall. Dental wear was slight, confined largely to enamel with the first mandibular molar showing early signs of dentine exposure; the direction of wear was horizontal and plane. Some teeth were lost post-mortem but those present were healthy and devoid of carious lesions. A single dental abscess was starting to form behind his left third mandibular molar. A supernumerary left maxillary canine was located posteriorly into the palate. He had malocclusion of the left first mandibular premolar and a pronounced overbite, which would have been characteristic in life. It appears he may have suffered from temporomandibular joint (TMJ) disease: the articulation was generally poor, especially on the right side. There was evidence of linear enamel hypoplasias on the left first mandibular and maxillary molars, showing periods of stress during his childhood likely around the ages of 1-2. His bones were robust, and there were early signs of osteoarthritis developing in his spine and elbows, consistent with a physically active lifestyle. There was diffuse green (copper) staining on the right zygoma, zygomatic arch, and right side of the mandible below the canine and premolars. No grave goods were retained with the skeleton, but it is likely that he was buried with a copper object next to the right-hand side of his face, which caused this staining. This may have been a decorative ear-plate worn by men, such as that illustrated by Burchell [47, reproduced in S5 Fig]. These varied from two to five inches in length and were known to have been worn by San people along the Riet River [48].

## S4.4 Jannetje

Jannetje was an adult woman aged between 45 and 60 years-at-death. She was an extremely gracile, small-bodied woman who stood approximately 1.45 m tall in life. The presence of squatting facets on her distal tibiae and on her tali indicate that she habitually used a resting posture with deeply flexed knees and ankles. Her skeleton bore the signs of age: her bones were light, consistent with osteoporosis, with signs of osteoarthritis in her hips and elbows. On Lodox radiography, the postcranial skeletal elements confirmed the presence of osteoporosis, observed as an increase in radiolucency, cortical thinning and associated expansion of the medullary cavities. Jannetje’s cranium showed a uniform/diffuse decrease in radiodensity, suggesting extensive rarefaction of spongy bone, a diagnostic sign of generalised osteoporosis [49] (see S6 Fig). Additionally, there were signs of severe cortical bone thinning and rarefaction of the inner and outer tables, presenting as thin, faded or non-existent or similar lines of radiodensity (S6 Fig). On CT, calvarial biparietal thinning was observed, resulting in groove/sulcus-like depressions in the ectocranial surface [50,51] (Fig. S6). Platybasia, a condition that results in abnormal flattening of the cranial base, was diagnosed based on the Welcher basal angle, which was greater than 140˚ [52] (S6 Fig). Basilar invagination refers to an anomaly at the craniovertebral junction that results in projection of the odontoid process into the foramen magnum and the vertebral column presses into the skull base [53,54]. The Boogard’s angle (154.3˚), foramen magnum angle (21.1˚) and clivus height (12.74mm) were measured and the values are consistent with this diagnosis [52, 55-58] (S6 Fig). The exact aetiology is unknown. Microcephaly was assessed metrically and ruled out. Only the basion-bregma height measurement was smaller compared with the average dataset [59], most likely the result of basilar invagination. Clinical studies reveal that people with basilar invagination show a variety of symptoms such as neurological and/or musculoskeletal complications [60,61]. Patients commonly require assistance with activities such as walking and/or carrying out routine household tasks [60]. Symptoms can become progressively worse over time [62]. Jannetje may therefore have been reliant on others in her community, especially as she aged.

Her teeth were heavily worn with full dentine exposure; the incisors showed reduced crown height. Wear was mainly in an oblique and concave direction. Her tooth wear pattern indicated that she favored her left side when chewing. Her maxillary third molars were missing; it appears likely they were congenitally absent. She had six active dental abscesses at the time-of-death, with severe alveolar bone resorption consistent with systemic dental infection. There was build-up of dental calculus on her molar teeth. Her left TMJ was badly eroded with signs of osteoarthritis (erosion and posteriorly shallow joint surface), which are indications of TMJ disease. Eating would have been difficult and painful, resulting in her growing progressively weaker and more prone to illness. Two copper bangles were preserved with Jannetje’s skeleton; she was probably buried wearing these on her right arm, which shows copper staining on the distal right humerus and proximal ulna and radius. There was also fainter copper staining on the lateral part of the left zygoma and temporal bone, which may suggest a burial position flexed on her left side, with her arms tucked tightly under her head, so that it was pillowed on her elbows. This position would place the right elbow on the left side of the facial bones. One bangle is fragmentary, one is complete. The latter is oval, shaped like the letter C, 50 x 45 mm internal diameter.

## S4.5 Klaas

Klaas was an adult man aged between 40 and 60 years-at-death. He stood approximately 1.44 m tall in life. The presence of squatting facets on his distal tibiae and tali suggest he habitually used a resting posture with deeply flexed knees and ankles; these facets were, however, less pronounced than in Jannetje. There is a small copper stain on the superior surface of the right pubic ramus; this is unlikely to derive from an ornament but may have been caused by a shroud pin or similar. His maxillary teeth were well-worn (the mandible was missing), all showing dentine exposure, in some cases with no occlusal enamel preserved; teeth were worn in either horizontal and plane or oblique and concave directions. His third maxillary molars had been lost antemortem. There were no signs of carious lesions, however, the teeth were over erupted and show early signs of alveolar inflammation likely due to the high wear levels. His spine showed early signs of osteoarthritis [63]).

Two healed antemortem traumatic lesions were observed on the right parietal bone; one a circular shape measuring 8.5 mm in height by 8.4 mm in length; and the other elongated in shape, 5.7 mm in height by 20.3 mm in length. Perimortem trauma was also shown by a circular, penetrating impact on the anterior surface of the left maxilla inferior to the orbit and medial to the left infraorbital foramen. The energy from this impact produced two primary radiating fractures: one extending laterally from the impact site across the anterior surface of the maxilla, terminating as the maxilla rounds posteriorly. A second, larger primary radiating fracture runs through the left lacrimal, ethmoid and sphenoid bones and produces a radiating basilar cranial (base-of-skull) fracture through the occipital bone on the left side of the foramen magnum (S4 Fig). This kind of fracture suggests his head was against something hard – like a wall, or the ground – when the high impact anterior penetration occurred, diffusing the force of the injury to the basilar cranium. This injury supports the statement in the archival records that the cause-of-death was “murder”.

## S4.6 Saartje

Saartje was a middle-aged adult woman, probably between 30 and 45 years-at-death and standing approximately 1.38 m tall. The presence of squatting facets on her distal tibiae and tali suggest she habitually rested in a resting posture of deeply flexed knees and ankles; these were less pronounced than in Jannetje. Copper staining was observed on the metopic ridge of the frontal bone approximately 3 cm anterior to glabella. This is consistent with a copper ornament worn in her hair; wearing of ornaments in this position is widely documented amongst San people [64,65]. Copper staining was also present on both the medial and lateral surfaces of the lower right ribs, at two locations on the anterior right tibia about one-third and two-thirds of the way down the bone, on the anterior right proximal femur, as well as the medial aspect of the left talus. On the talus, there are green encrustations (not just staining) but there is no discolouration of the articular surface of the medial malleolus of the tibia. The talus must have been in contact with a copper object only after burial and decomposition of the body. The pattern of copper staining on the post-cranial skeleton is consistent with the presence of two copper bangles on her right lower leg, perhaps ankle bangles that rode up her calf during or after burial. These would account for the two distinct copper stains on the right tibia, and if she was buried in a tightly flexed position, they could also have produced the copper staining on the right femur and lower right ribs. This last is post-depositional since it occurs on both the medial and lateral surfaces of the ribs. There were no signs of osteoarthritis, though she had fusion of the 5^th^ lumbar vertebra to the sacrum. There is evidence of physiological stress with two bilateral Harris lines on her tibiae. Estimates of the age at onset indicate that one occurred when she was younger than one and the other at around 13 or 14 years of age. These suggest nutritional stress especially from protein and vitamin deficiency [43]. She had maxillary and mandibular tori. Most teeth were worn to the level of dentine clusters or full dentine exposure, primarily horizontal wear in both planes and in some areas concave. The only exception was the maxillary right third molar that showed no wear. There was evidence of active dental abscesses around four mandibular teeth, with alveolar bone resorption and dental calculus build-up on the teeth. Such infections would have been very painful and made eating difficult. The archival records mention two pick holes in the left side of the cranium. The light color of the bone surrounding these holes indicates they were recent postmortem damage, likely caused in the 1920s at the time of disinterment.

## S4.7 Totje

Totje was a young adult man between 25 and 30 years-at-death. He stood approximately 1.51 m tall in life. The cranium and mandible were absent although they are mentioned in the archival catalogue. There was antemortem trauma in the form of a healed fracture of his right second metacarpal. Ossification on the costal cartilage between the ribs and thoracic vertebrae suggest physical labor during life. There was no other evidence of trauma or disease on his skeleton. The archival records stated that he died of tetanus, a rapid infection that would not leave any evidence on the skeleton.

## S4.8 Voetje

Voetje was an older man, over 44 years-at-death, who stood approximately 1.54 m tall in life. The frontal bone showed evidence of the premature fusion of the metopic suture, known as benign metopic ridge [66], creating an unusual shape to the frontal bone in adulthood. The presence of slight squatting facets on his distal tibiae and on his tali suggest he regularly employed a resting posture of deeply flexed knees and ankles. There was antemortem trauma - healed fractures - of his nose, both zygomas and the left zygomatic arch with more severity on the left side, along with healed fractures of his lower ribs on both sides. There was evidence of osteoarthritis on his spine (lower thoracic and lumbar areas) and in both shoulders (humeri and scapulae). There was evidence of apophyseal degenerative joint disease between the 4th and 5th lumbar vertebrae, the inferior articular facets of L4 and superior of L5 with eburnation [67: pp. 96]. In addition, the left patella, proximal tibia and left cuboid had eburnation caused by severe joint wear, which would have been painful, and he may have walked with a limp on the left side. There were prominent muscle attachment sites across his shoulders, arms, legs and knees, showing he had habitually engaged in activities such as carrying heavy objects or heavy manual labor. There was no mandible preserved, and he was edentulous in the maxilla with evidence of an active, chronic peri-mortem dental infection. Eating would have been difficult, if not impossible. There was possible left side TMJ disease present.

## S4.9. Osteobiographic methodology

Osteobiography is the reconstruction of an individual and their life history through analyses of their skeletal remains and should be contextualised within a person's biological, physical, socio-cultural and temporal environment [68-70]. Data were obtained using standard non-destructive and non-invasive methods. Visual analyses were conducted using a magnifying lamp (3 diopter with 1.75x magnification), whilst measurements were carried out using Vernier callipers and osteometric boards.

To estimate sex, the pelves and crania were assessed [71-72]. To age immature individuals, epiphyseal fusion and dental eruption were assessed [73-76]. To age adults, in addition to tooth eruption and epiphyseal fusion, sternal rib ends, cranial suture closure, cranial transition analyses, auricular and pubic symphyseal surfaces of the pelvis were assessed [77-81]. It is known that standard methods of assessing age-at-death, many of which rely on assessment of wear and tear on the joints, tend to under-estimate true ages in small-bodied populations such as San and/or Khoekhoe people. Available methodologies are based on recent populations whose cultural practices and activity levels may differ from historical populations, leading to errors in age estimates [46,82,83]. Stature was estimated using femoral length [84,85]. The remains of each person were visually assessed for abnormalities (disease, trauma or stress) and any that were identified were described and studied [67,86,87]. Analyses of Harris lines were carried out on the anteroposterior radiographs of humerii, radii, ulnae, femora, tibii and fibulae, acquired on a Lodox Statscan digital X-ray machine (Lodox Systems, Johannesburg, South Africa). The radiographic parameters applied were 50 kVP, 80 mA, 44 millisecond exposure time and 1.3 m distance. Harris lines were diagnosed using the criteria outlined by Bloem et al. [43], and only bilateral incidence was recorded. Age-at-onset was determined using the methods of 88 and 89. The quantity and direction of tooth wear was scored [90-91].

# S5. Facial reconstruction and depiction

The technical process of facial reconstruction refers to the remodeling of facial muscles and soft tissue, and estimation of facial features, to recreate an accurate craniofacial shape based on cranial anatomy. The principle is that the cranium and the face are directly related to each other in shape and proportions. Given that every cranium is unique, it follows that close analysis of it along with an understanding of facial anatomy will allow a recognizable face to be recreated from a cranium. The process follows anatomical principles and anthropometric standards that apply across all populations.

The difficult part of this process is creating a plausible and relatable face from an accurate reconstruction of facial shape, where the unavailable facial detail such as skin colour, eye colour, hair colour/style and fine texture (such as moles, scars, wrinkles) from skeletal assessment becomes salient. Without these details, a facial reconstruction is less relatable/recognizable, so they must be carefully interpreted, relying on individual biological profile, craniofacial analysis, informed by other relevant analyses (*e.g.* DNA), and other contextual information. This interpretation is limited to what can be scientifically justified, so the preference is for facial depictions in grayscale, with external details such as clothing and hair suggested rather than explicitly stated, using visual techniques (selective focus, blurring) that introduce ambiguity, especially in forensic cases [92]. The known population of an individual becomes relevant only when this surface detail is applied.

Sometimes, as in this project, forensic objectives and archaeological interests find common ground, where facial depiction may contribute to the visual communication of science as well as reconstituting individual personhood for unknown or past people. The depictions present the faces of highly individual people who once lived, breathed, loved, laughed, had relationships and families, suffered great hardship and hopefully also moments of celebration, felt the hot sun on their skins in summer and the bitter bite of winter, and dreamed together under the Sutherland night sky.

## S5.1. Craniofacial analysis and reconstruction

The most accurate facial reconstructions are produced from complete crania, but it is also possible to produce reconstructions from those that are damaged or have the mandible missing. Without the mandible present, we cannot know any specific information about the jawline, chin shape and lower teeth or lip shape. Incomplete crania requiring parts to be estimated impacts the confidence of the resulting depiction.

Close craniofacial analysis prioritizes the reconstruction of individualized anatomical features using validated standards [93,94]. Craniofacial landmarking and average soft tissue data tables guide the process, while their limits are recognized and critically appraised. Paying close attention to the characteristic details of a particular cranium to depict a highly individual face can be both methodology and ethos.

For Schramm [95], the use of averaged soft tissue thickness data as a core feature of facial reconstruction methodology is a persistent artefact of biological anthropology legacies as ‘race science’. She argues that the practice is inseparable from a ‘classificatory gaze’ because soft-tissue data collection is frequently linked to a specific population or group. Thus, it can imply only facial ‘types’ rather than individual faces. However, this demonstrates a partial understanding of how different facial reconstruction methodologies regard and apply these data and assumes a lack of critical perspective on the reprehensible legacies of biological anthropology from within the field.

In the South African context, a history of organizing people into assumed or self-determined population groups is well-documented and recognized -- including within biological anthropology -- as continuing to haunt the present [see 96-101]. Furthermore, contemporary expressions of resisting and reviewing such legacies may be seen in more recent approaches to considering concepts of ancestry and sex in biological anthropology [102-103].

Published soft tissue thickness data derive from a broad range of sampling and collecting methods that should be critically considered before their applicability to a craniofacial reconstruction case can be assessed, including claims to homogeneity based on ethnic (self-)identification or citizenship of a particular country. Facial reconstruction accuracy and validation studies have demonstrated best results from data gathered via *in vivo* computerized tomography (CT) imaging, in combination with virtual (computer-enabled) methods [104-108]. Further, it has been shown that facial reconstruction will produce the same face shape regardless of the population-based soft-tissue thickness data used; this has been comparatively tested using tissue depth data from different populations on the same cranium [109: pp. 151-6]. Some researchers have advocated for averaging all published soft tissue thickness datasets and organizing them only according to adults and juveniles, and then by sex, for different reasons [110-112].

No reliable soft-tissue data are available for individuals of San or Khoekhoe ancestry, but various *in vivo* datasets for South African populations were available for both adults and juveniles, and were used to create new aggregated data based on common craniofacial landmarks with reference to the osteological profiles. Mean values for adult males of ‘mixed ancestry from the Western Cape’ [113] were compared and averaged with those for ‘adult Zulu males’ [114], and mean values for adult females were compared and averaged for common landmarks using Phillips & Smuts [113] and Cavanagh & Steyn [115] for ‘black South African females’.

Unlike in adult faces, few reliable facial reconstruction and feature estimation methods have been developed specifically for children. Growth happens in a downwards-and-forwards pattern in children, with boys exhibiting more obvious changes to their facial structure after puberty. The development of the permanent teeth plays a significant role in the general lengthening of the face. Thus, although a child’s features appear fairly generic, they do hint at the basic elements of adult appearance. For the two juvenile faces, mean values for South Africans of Bantu-speaking [116] ( “bureaucratically identified as ‘black’, and descendant from the migration of Bantu-speaking agropastoralist sub-Saharan migrants around 2000BP” [pp. 285]) and Mixed Ancestry [117] “bureaucratically identified as ‘coloured’, which is a biologically heterogenous group with variable and complex admixture. This group includes people descendant from Indigenous African Khoesan, European, Bantu-speaking African, Asian (Malaysia, Indonesia and India) and Madagascan Cape slaves/migrants. Therefore, genetic admixture from Europe, Africa and Asia makes ancestry estimation a challenge. The term ‘mixed’ is used knowing many are in fact genetically homogenous to one origin yet were classified under a single racial label during apartheid, which is still used today.” [ pp. 285]) ages 6-10 were compared with averaged values for additional landmarks derived from ‘Canadian aboriginal subadult females’ 3-8 yrs [118] and ‘black North American children’ 3-8 years [119 modified by 93].

Detailed photographic documentation aided the interpretation of detailed surface information in the 3D virtual cranial models. Missing or damaged bony parts, including missing mandibles for Igue We, Voetje and Klaas, were first rebuilt using available mandible estimation methods (see Fig. S7), which provide only general proportions as they have not yet been as robustly tested as those for other facial features [120-226].

Missing maxillary teeth were remodeled for illustrative purposes, but not used for feature estimation calculations. All reconstructed skeletal parts are shown as purple in the cranial models depicted in the figures in this section (see Fig. S8). Distinctive dentition that would have been a characteristic or potentially identifying feature in life (*e.g.* diastema, mixed adult/juvenile dentition, central incisors lost antemortem) was shown through slightly parted lips while maintaining a neutral facial pose.

## S5.2 Facial depiction

Crania provide only facial shape and proportions, and some feature details. Precise information about body mass, hair length or style, distinguishing marks, skin texture or any color information is not available; these details also change throughout a person’s life. In facial reconstruction, this information is interpreted using the best available associated information. With reference to the osteobiographical findings, craniofacial analysis and visual research, supported by the other findings presented here, some features of interest in the synthesis between reconstruction and depiction are summarized.

### S5.2.1 Igue We

Signs of rudimentary attempts at preserving the integrity of this cranium (stabilizing the fragmentation that possibly resulted from its excavation context) were evident on visual inspection, along with misaligned/distorted parietal area. A sagittal slice through the CT-generated 3D model revealed that the cranial cavity was at some stage filled with a substance with a similar density to bone (probably Plaster of Paris) as it rendered solid in the CT segmentation process, making it impossible to accurately identify the necessary internal structures for mandible estimation (see S7 Fig, far left), thereby making this depiction the most qualified of the set. Virtual cranial reconstruction included remodeling of the left zygomatic arch, and the right zygomatic bone and part of the right maxilla were reconstructed by mirroring the left side and adjusting relative to the surrounding bone (see S8 Fig). In the final depiction (see S9 Fig) he is shown with a fairly pronounced diastema, his dark hair worn slightly long as if it might be trained into short dreadlocks, with a subtle suggestion of facial hair, and wearing beads and possibly a kaross.

### S5.2.2 G!ae

The eruption of adult central incisors in G!ae is an indication of age, so his right central incisor (lost post-mortem) was remodeled for illustrative purposes. His orbits had very defined lower margins which would have created characteristic creases below his eyes. At the younger end of the estimated age spectrum, this child would have just passed through toddlerhood. Some characteristic elements of early childhood appearance are still evident in his large head and dainty lower face, full cheeks and pointed chin. His ears still appear to sit low on his head. He is depicted with close-cropped hair and the suggestion of a kaross (see S10 Fig).

### S5.2.3 Saa

Saa has a wide, full mouth and almond-shaped eyes with a slightly upturned nose. Her face is beginning to show the characteristic bone structure of the San, with well-defined cheekbones and angled jaw. She is depicted with unbraided hair worn slightly long, with the suggestion of a kaross over one shoulder (see S11 Fig).

### S5.2.4 Cornelius

His characteristic dentition and dental occlusion would have produced the appearance of an overbite, with a slightly protruding lower left lip, and thicker tissue at the chin to compensate (close the lips). His strong face is depicted as reflecting the signs of middle-age; his dark hair is worn slightly long with the suggestion of a short beard/moustache. His clothing includes the suggestion of a white work shirt open at the neck, revealing a small string of beads (see S12 Fig).

### S5.2.5 Jannetje

Calvarial biparietal thinning, resulting in groove/sulcus-like depressions in the ectocranial surface, and platybasia were visible in her cranium (see S6.6), but these were somewhat moderated through the facial reconstruction, resulting in an only slightly unusual facial appearance. It appears that the proportions of Jannetje’s upper face developed normally, but her midface and lower face did not develop at the same rate, resulting in prominent, downturned eyes (lateral rotation of orbits) with characteristic creases below her eyes (related to very sharp lower orbital margins), a short chin and delicate jawline. Her nasal septum deviates right with a slight crest on the nasal bridge, but her nose would have appeared very flat in profile. The undulating form of her cranium would have been visible had she worn her hair very short. Towards the end of her life, both her central incisors were missing, with the right lost more recently than the left. She is depicted with wrinkled skin suggestive of her age and environmental conditions, with greying hair worn slightly long and a sheepskin kaross around her shoulders (see S13 Fig).

### S5.2.6 Klaas

His reconstruction is somewhat qualified due to mandibular estimation. Klaas had unusually short and wide mastoid processes, which were somewhat asymmetrical resulting in his right ear being slightly more prominent than his left. His strong face is framed by short hair and he is depicted wearing a shirt/jacket in the style of a nineteenth century labourer (see S14 Fig).

### S5.2.7 Saartje

She is depicted with her lips slightly parted to show a missing left central frontal incisor which would have been a characteristic and identifiable feature in life. Her nose would have appeared slightly asymmetrical, deviating slightly to the left, with the left nostril and lateral nasal cartilage slightly larger than the right. She is shown with wrinkled skin suggestive of age and environmental conditions, along with greying hair, worn short but with enough length to have some volume. She wears a kaross knotted on the shoulder, with various beads around her neck. Her depiction was updated in 2021 to include a pendant on her forehead, which would have been woven into her hair, evidenced by copper staining on her frontal bone, and with reference to visual sources showing traditional San and/or Khoekhoe dress (see S15 Fig).

### S5.2.8 Voetje

No maxillary teeth were present but three had been lost post-mortem; these were remodeled for illustrative purposes. Given his general dental condition, an edentulous mandible was considered the most plausible of the three estimated versions produced. As a result, his lips would have appeared thinner and sunken with possible asymmetry in the cheeks due to significant bone resorption in the right maxilla, emphasizing his strongly defined cheekbones. If the result of blunt force trauma, the healed depression fracture to his right zygomatic and full fracture of his left zygomatic would not necessarily show scarring on the skin surface, so this is not depicted. With additional evidence of a previously broken nose, age and environmental exposure, Voetje would have presented a very weathered face to the world. He is depicted as wearing his greying hair short-cropped, showing some signs of thinning, with a short beard and moustache. Like Klaas and Cornelius, he wears a work shirt, open at the neck (see S16 Fig).

## S5.3. Presentation to families

The process of reconstructing crania (where relevant) and face were carefully documented visually along with accompanying written narratives synthesizing the various scientific and biographical data for each individual. This was done in the interests of complete transparency; in a historical restitution initiative such as this, ‘reconstruction’ carries both literal as well as metaphorical import in respect of bodily integrity and reconciliation. Furthermore, the process needed to be explicitly understood by a broad and diverse audience of varying literacies and diverse spoken languages.

For presentation to the families, the eight facial depictions were digitally printed onto compressed and treated wood panels (20 x 20 cm each) prepared for wall-mounting, with a summary biography of each individual on a label affixed to the back of each panel, and set into an archival presentation box (see S17 Fig). A bilingual (Afrikaans and English) and fully illustrated facial reconstruction ‘album’ was produced alongside the panels to explain the facial reconstruction process and its objectives in forensic investigation and historical research (see S18 Fig). Two copies of the portrait collection and album were presented to the families, with an additional four copies lodged with UCT (2), Face Lab (1) and practitioner Smith (1).

The anticipation of seeing these faces was palpable among those members of the descendent families assembled for the knowledge sharing session in October 2019 in Sutherland. Responses were generally very positive, characterized by a spectrum of complex emotions including nervous excitement, caution (perhaps not fully trusting or understanding the process of facial reconstruction) and joy. One expressed surprise at their photorealism, expecting ‘something simpler, like a carving or a sketch… something more ancient-looking’. Some frankly shared that while they appreciated the images, they found them somewhat unnerving. Others were preoccupied with pointing out physical resemblances between the faces and other family members, evidencing immediate connection with (or projections of) imagined personhood and relatability.

The use of facial reconstruction/depiction is noteworthy in the context of this initiative and the broader practice of heritage and scientific knowledge-sharing internationally. We only aware of one other heritage project with a facial reconstruction of a known individual, George Lister, completed with participation of his contemporary descendants. The depiction of Lister referred to as a ‘striking visual reminder’, was produced by Face Lab and is included in the [Fewston Assemblage exhibition at the Washburn Heritage Centre](http://www.washburnvalley.org/exhibitions) (York, United Kingdom).No other examples using facial depiction of recent ancestors exist in South African heritage practice that we are aware of, except for the Cobern busts as discussed by Morris [127] and Schramm [128] (Referring to three facial reconstructions produced by odontologist V Phillips and visual artist S Rosendorff using manual methods and presented as sculptural busts. They are currently situated in a vitrine, in the Department of Human Biology at UCT, and no longer in the ‘tearoom’ as Schramm describes.), and certainly not as part of a participatory process with restitution and knowledge-sharing as primary objectives. Critiques have been directed at facial reconstructions in traditional museum contexts for their “socio-ethical implications” [129] or “invalidated” methods that produce “inaccurate” results [130]. For heritage institutions or individual practitioners who do not consider the ethical implications of working with human remains (or representations thereof) in this way, including the concerns or wishes of descendant communities (known or unknown), or the claims and limitations of such depictions, such critiques are justified. Presenting the Sutherland faces as 2.5D digital composites that resemble (but are not) photographs, versus as realistic sculptural busts, was to allow the images to suggest a moment in time; snapshots or formal portraits do not define an individual’s entire story [see 131-133] - and they may be easily adjusted should new information emerge (as in the case of Saartje). The circumstances by which the Sutherland facial depictions came to be -- requested by descendent families; critically considering the methods and reference materials used to produce them, and their final presentation -- thus make a significant contribution in the context of critical heritage practices more broadly, and further stress the interdisciplinary nature of this process (science, art and humanities) as a primary means of visual communication of the scientific findings.

## S5.4. Facial reconstruction and depiction methodology

### S5.4.1 Computed tomography imaging

The Medical Image-based Inferencing and Distributed Diagnosis research group based in the Division of Biomedical Engineering at UCT conducted the CT imaging to generate better virtual cranial (and mandibular, where available) models. The CT imaging was performed on a Philips Brilliance 64-channel CT machine (Phillips Healthcare; Best, Netherlands) located at the UCT Private Academic Hospital. The imaging was conducted with the assistance of an expert clinical radiographer. A single cranium, with its corresponding jaw, where applicable, were imaged and placed on a polystyrene block of 55 mm thickness to raise it above the imaging bed and improve segmentation. The CT parameters used were (slice thickness and increment: 3 mm x 3 mm with reconstruction of 1.5 mm x 0.5 mm; Tube voltage: 120 kVp; Current: 35 mAs; and Matrix 512 x 512). Throughout the process of imaging, the remains were handled with the utmost respect and carefully concealed to prevent accidental viewing. The raw CT reconstructions were saved in digital imaging and communications in medicine (DICOM) format and written to separate digital video disc storage.

### S5.4.2 Image processing

The image processing steps were trivial since the crania were dry and imaged without other objects in the field of view. Each set of DICOM files belonging to an individual were loaded into Amira v6.2.0 software (Fei Imaging, [www.fei.com](http://www.fei.com)) and a standard filter (noise reduction non-local means filter) was applied to reduce noise in the images. Next, a semi-automatic segmentation of the image volumes was performed to obtain labels and subsequently an isosurface defined label scalar fields were computed. Visual verification of every CT slice was performed and where errors were detected, manual correction was applied to ensure high quality segmentation. For the teeth, a similar process of manual segmentation was used.

To ensure only the exterior surface of the segmented crania would be generated, the “Fill hole” module in Amira was used to fill holes and remove spots on the segmented crania and on the mandibles. Holes that were not filled using this module were manually selected and added to the bone tissue. The results of the segmentation process were evaluated to ensure they were representative of the objects in the CT images. The “goodness measure” assessed the segmentation on intra-region uniformity, inter-region contrast and region shape [134]. A segmentation was deemed to be good if, for a CT slice having objects (cranium bone) and a background (exterior region), the cranium was uniform rather than having holes (object and background). From this, high-resolution 3D surfaces were extracted and smoothed to produce high resolution 3D surface mesh models. Crania 3D surface mesh models were composed of an average of approximately 0.65 million vertices or nodes and 1.3 million triangles or facets (see S19 Fig). Mandible 3D surface mesh models were composed of an average of approximately 50 000 vertices and 100 000 triangles. These models were saved in the stereolithography file format.

### S5.4.3 Facial reconstruction

An entirely digital and non-destructive workflow was followed, importing stereolithography files into Geomagic Freeform Plus (v.2019.1.69) with Phantom Touch X haptic device. The system provides the facial reconstruction practitioner with touch-based feedback, aiding assessment of surface textures.

All virtual crania and mandibles were rearticulated to replicate occlusion with a relaxed jaw (upper and lower teeth slightly parted). Complete adult crania were then positioned in the Frankfurt horizontal plane prior to commencing with feature assessment and reconstruction. Juvenile skulls were positioned on a horizontal plane running from anterior nasal spine through the middle of the external auditory meatus [93: pp. 250, fig. 8.9], resulting in a slight upward tilt. This replicates the angle children’s faces are usually seen by adults, which may encourage recognition in forensic contexts [93: pp. 249].

Following the Manchester method updated for virtual sculpture [105], virtual clay facial muscles from a pre-existing custom-built database were then placed and deformed according to individual cranial features. Facial features and fat were then modelled using evaluated methods and a skin layer was added that reflects the estimated age and likely body mass of each individual to complete the craniofacial shape. Skin opacity was adjusted throughout the process to evaluate the accuracy and consistency of feature prediction methods. Once the craniofacial shape was complete, screenshots of the 3D shape model were taken in various views, and the frontal view was imported into Photoshop, where appropriate textures are added from photographic databases.

### S5.4.4 Texturing

For each completed facial reconstruction, visual textures were applied to a screenshot of each reconstruction taken in the frontal view. Working in Adobe Photoshop CC using layers and layer masks, these included suggested clothing and hairstyles selected from a visual database constructed from various historical and contemporary sources depicting San and Khoekhoe people, as described in the main paper.

# S6. Genetic analyses

## S6.1. Sex determination

For the Sutherland Nine and the corresponding DNA libraries the genetic sex was successfully determined (main text Table 1 and S1A Dataset, S20 Fig). The genetic sexes were congruent between different DNA libraries from one individual (SUT003.A, SUT003.B & SUT009.A1, SUT009.A2, SUT009.B) for Voetje (UCT 29/SUT001), Saartje (UCT 43/SUT003), Cornelius (UCT 44/SUT004), Totje (UCT 45/SUT005), Klaas (UCT 50/SUT006), and Jannetje (UCT 54/SUT009).

## S6.2. Y chromosome haplogroup results

Klaas (UCT 50/SUT006) belongs to Y-chromosomal haplogroup A1b1b2a, as supported by 26 derived allele states. The sub-haplogroup A1b1b2a is, together with A1b1a, common among southern African San and/or Khoekhoe populations while being rare in Bantu-speaking populations [135]. Two approximately 2000-year-old individuals from South Africa, from St. Helena Bay and Faraoskop site (near Clanwilliam), were previously also assigned to A1b1b2a [136]. Another two individuals from Ballito Bay also approximately 2000 years old, belong to the ancestral haplogroup A1b1b2 [137]; their further downstream subtype is unclear.

Voetje and Igue We (UCT 29/SUT001 and UCT 31/SUT002) potentially also carry downstream A haplotypes. Although these individuals show higher X chromosome contamination estimates, the majority-rules genotyping should compensate for this circumstance.

Cornelius (UCT 44/SUT004) was assigned to haplogroup E1b1a1a1d1, as supported by 102 derived allele states. This haplogroup is widely distributed across western and southern Africa but is found predominantly in Gabon, Angola, and Zambia. In general, Haplogroup E is the most common Y-chromosome haplogroup in sub-Saharan Africa and is present at a frequency of 63.2 % in the Karretjie people and 60% in South Africans of Mixed Ancestry [135]. However, the only other ancient E Y-chromosomal data available to date from sub-Saharan Africa are from the 4,500-year-old Mota hunter-gatherer from Ethiopia, who belonged to haplogroup E1b1 [138] and a 400-year-old forager from Panga ya Saidi, Kenya, who belonged to haplogroup E1b1b1b2 [136]. Since Cornelius exhibits strong signs of contamination in autosomal principal component analysis (PCA) (X chromosome contamination estimate: 9 %), this downstream assignment is stated with reservations (S1C Dataset).

## S6.3. Mitochondrial DNA (mtDNA) haplogroup results.

Regarding the mtDNA haplogroups, The Sutherland Nine all belong to L0d lineages (L0d2a1, L0d2a1a, L0d2c2, L0d1a1a1, L0d1b2b and L0d1b2b2c) (Dataset S1B). Haplogroup L0d (as well as L0k which is absent in the Sutherland sample) is present almost exclusively in San and/or Khoekhoe southern African populations, and those in close contact with them such as neighbouring Bantu-speaking populations [139-140].

The L0d2c haplogroup, found as downstream haplotype L0d2c2 in Saa (UCT 51/SUT007) individual, is most common in present-day ǂKhomani San and Nama people from Namibia and South Africa, but it is also found at lower frequencies in other San and/or Khoekhoe populations and South Africans of Mixed Ancestry and has recently been identified in some Bantu-speaking populations [141]. Furthermore, one of the South_Africa_2000BP hunter-gatherers from St. Helena Bay on the southwest coast of South Africa displays the sub-haplogroup L0d2c1c and another from Ballito Bay on the southeast coast carries the sub-haplogroup L0d2c1[136,137].

Haplogroup L0d2a1 is present in Saartje (UCT 43/SUT003), Totje (UCT 45/SUT005), Klaas (UCT 50/SUT006), G!ae (UCT 52/SUT008), and Jannetje (UCT 54/SUT009) (as both L0d2a1 and sub-haplotype L0d2a1a). L0d2a and its downstream sub-haplogroups (with a predominance of subclade L0d2a1a) are more frequently observed in present-day populations than L0d2c [139]. L0d2a is also carried by the South_Africa_2000BP.SG Ballito Bay B individual [137]. The highest frequency is found in the Karretjie people, ǂKhomani San, and Nama people [142]. L0d2a is also found in Bantu-speaking populations as well as in South Africans of Mixed Ancestry [143]. Consistently, L0d2a1a was also observed in one 300-500-year-old female South African individual from Champagne Castle (Drakensberg), who exhibits an otherwise typical Bantu-speaker genomic signature [137].

L0d1 is the most common sub-haplogroup in southern Africa: it is present in all San and/or Khoekhoe populations, as well as in most southern Bantu-speaking populations, and to some extent in individuals from Bantu-speaking populations of Zambia and Angola [139,141,143]. Sub-haplogroups L0d1b, found in Voetje (UCT 29/SUT001) (L0d1b2b2c) and Igue We (UCT 31/SUT002) (L0d1b2b), and L0d1a, found in Cornelius (UCT 44/SUT004) (L0d1a1a1), are represented within San and/or Khoekhoe, and also recently admixed populations, including the ǂKhomani San, and South Africans of Mixed Ancestry. While Lod1b sub-haplogroup L0d1b1 is almost absent in linguistically classified San (6 %), L0d1b2 predominates within culturally classified San (65.7%) [141]. Besides Voetje (UCT 29/SUT001) and Igue We (UCT 31/SUT002), two other ancient African samples were assigned to L0d1b2 sub-haplogroups, a 2000-year-old hunter gatherer from Faraoskop, South Africa (L0d1b2b1b), and a ~6100-year-old hunter-gatherer from Fingira, Malawi (L0d1b2b) [136]. L0d1a haplogroups are attested so far in only one ancient sample, a ~1200-year-old pastoralist from Kasteelberg, South Africa (South_Africa_1200BP.SG) (L0d1a1a) [136].

## S6.4. Kinship analysis

Kin relationships were investigated using the pairwise nucleotide mismatch rate between all pairs of individuals (S21 Fig). Two important base lines were observed. First, for some individuals multiple libraries were generated, thus enabling us to report the average mismatch rate within individuals (caused by heterozygosity, *i.e.* differences in nucleotides within individuals) at between 0.1 and 0.15 (green area in S21 Fig). Second, for the bulk of individuals a distant degree of kin-relationship can be assumed, with the median of that group expected to be around 0.25. These values are consistent with one another, as within-person mismatch rates are expected to be 50% of the unrelated baseline, which is the case here. First-degree relatives are expected to fall between the two baselines (yellow shading in S21 Fig). The fact that all pairs are quite clearly outside that area suggests that there are no first-degree relatives in the sample, *i.e.* no parent-child relationships or siblings. The only pair for which a 2^nd^ degree relationship is robustly supported is Saartje (UCT 43/SUT003) and Klaas (UCT 50/SUT006). Further, evidence was observed for a 3^rd^ degree relationship between Saartje (UCT 43/SUT003) and Jannetje (UCT 54/SUT009) and between Klaas (UCT 50/SUT006) and Jannetje (UCT 54/SUT009).

## S6.5. Population genetic analysis

### S6.5.1 Principal component analysis

We used PCA to relate the three Sutherland individuals with well-preserved DNA (Jannetje, Klaas, and Saartje) to 42 present-day sub-Saharan African populations (S22 Fig). Like the four previously published 2,000-year-old South Africans (discussed earlier), the Sutherland individuals share most ancestry with southern African San and/or Khoekhoe populations in the PCA [136]. However, although the Sutherland individuals cluster closely with those 2,000-year-old South Africans, they are slightly shifted in the direction of Tanzania_Luxamand_3100BP on the ancient eastern-southern African cline. Therefore, the intermediate position of the Sutherland individuals is also indicative of their genomic composition, suggesting a high level of Indigenous South African ancestry but also an additional, small amount of non-San and/or Khoekhoe ancestry [136].

To investigate the fine-scale affinities of the Sutherland sample, a second PCA of 28 populations was calculated. This also identifies sub-structure within individual San and/or Khoekhoe populations as well as highlighting relationships between genetic and linguistic groups. According to Pickrell and colleagues [144], present-day South Africans can be divided into three broad genetic clusters: a predominantly non-San and/or Khoekhoe cluster and two San and/or Khoekhoe clusters. The San and/or Khoekhoe clusters do not conclusively correspond to linguistic affiliation; whereas one is comprised of Ju/’hoansi (Ju_hoan_North & Ju_hoan_South) and !Xuun, who speak closely related languages/dialects, the other includes populations speaking languages belonging to all three major language families. The San and/or Khoekhoe clusters instead reflect to some extent geography, corresponding roughly to the northwestern Kalahari (*e.g.* Haiom, Khwe, Shua, Tshwa) and southeastern Kalahari (*e.g.* Taa groups, ǂKhomani San [human origins (HO) panel], ǂHoan) with isolation-by-distance explaining most genetic affinities between the different autochthonous groups [145].

In congruence with previous PCA and ADMIXTURE analyses by Schlebusch and colleagues [137], we show in a third PCA of 7 southern African San and/or Khoekhoe populations that the Sutherland individuals cluster closest to present-day southern San and/or Khoekhoe, especially ǂKhomani San <HO>, Karretjie and Nama peoples, which is congruent with their geographical origin (S23 Fig). This was further supported by unsupervised likelihood model-based clustering using allele frequencies (S24 and S25 Figs).

### S6.5.2 f-statistics

*f_3._* Outgroup *f*_3_-statistics were calculated for Saartje, Klaas, and Jannetje (SUT003/UCT 43, SUT006/UCT 50, and SUT009/UCT 54) to measure the shared genetic drift between their genomes and the genomes of the present-day HO reference populations. All three exhibit highest shared genetic drift and strongest genetic affinity to ǂKhomani San <1240k>, followed by other San and/or Khoekhoe populations like ǂKhomani San <HO>, Ju/’hoansi (Ju_hoan_North & Ju_hoan_South), Taa_East, Taa_West, Taa_North, /Gui and Naro (Fig. S26a). Although the differences between the population affinities are not significant (applying a significance threshold of α = 0.05), the ordering can be considered to some extent informative. The affinity to members of both the northwestern Kalahari and southeastern Kalahari clusters can be explained, since the above-mentioned populations harbour the highest amounts of Indigenous South African ancestry and the smallest non-San and/or Khoekhoe admixture components. Populations that are heavily admixed with west African or East African/Eurasian sources score systematically lower in the *f*_3_-statistics (*e.g.* Tshwa, Khwe, Shua and //Gana). Similar results are obtained when comparing the Sutherland individuals to populations from the Schlebusch panel (146), with the Karretjie population exhibiting even more shared drift with the Sutherland genomes than the ǂKhomani San <1240k> (S26b Fig).

When comparing the Sutherland individuals with ancient samples from Africa, highest affinities are observed with non-admixed Indigenous South Africans like South_Africa_2000BP.SG. Again, the affinity decreases with decreasing Indigenous South African ancestry and increasing non-San and/or Khoekhoe ancestry. Ancient Malawi hunter gatherers exhibit intermediate outgroup *f*_3_ values according to their ~60 to 65 % Indigenous South African ancestry^19^. Lowest outgroup *f*_3_ values are estimated for Tanzania_Pemba_600BP and South_Africa_400BP, which trace all or most of their ancestry to West Africa because of the Bantu expansion (S27 Fig) [137].

*F_ST._* The three genomes of Saartje, Klaas, and Jannetje were grouped together and F_ST_ was calculated between them and the present-day populations of the HO reference panel using smartpca [147] v.16000 with the options inbreed: YES and fsthiprecision: YES. The Sutherland individuals exhibit lowest F_ST,_ therefore, the strongest genetic affinity to ǂKhomani San <HO> and Nama people, being significantly less differentiated from the following highest scoring populations Naro, !Xuun, Taa_West and Taa_East (S28a Fig). Congruently, F_ST_ statistics were also calculated using the Schlebusch-1240k dataset. Populations that show the lowest F_ST_ to the Sutherland individuals are the Karretjie, the Khomani San and the Nama (S28b Fig).

*f_4._* Most non-San and/or Khoekhoe populations share more genetic drift with the Sutherland individuals than with South_Africa_2000BP as shown by the *f*_4_ statistic (Outgroup, Test; Sutherland, South_Africa_2000BP). This is expected since the Sutherland individuals already harbour non-San and/or Khoekhoe ancestry that was introgressed into the South African Indigenous population during the spread of pastoralism after 2000 years BP. Therefore, the Sutherland individuals exhibit an excess in non-San and/or Khoekhoe alleles not found in South_Africa_2000BP. Interestingly, for many populations that scored high in the outgroup *f*_3_ statistic (like ǂKhomani San <1240k>, Ju/’hoansi (Ju_hoan_North <HO> & Ju_Hoan_South), Taa_East, Taa_West, Taa_North, /Gui etc.), this *f*_4_ statistic is insignificant. This is probably because these groups show a genomic composition similar to the Sutherland individuals (with only a minor contribution of non-San and/or Khoekhoe ancestry) (S29a Fig).

To test the monophylum comprising Sutherland and South_Africa_2000BP.SG, the Sutherland individuals were compared with modern populations and with South_Africa_2000BP.SG (Outgroup, Sutherland; Test, South_Africa_2000BP). No ancient or present-day population shares more genetic drift with the Sutherland individuals than South_Africa_2000BP. Only South_Africa_2100BP.SG seems to be equally related to both as expected from the sample age (S29b Fig). Subsequently, the relationship between the Sutherland-South_Africa_2000BP.SG clade and the ǂKhomani San (ǂKhomani_San.DG from 1240k) was tested.

The *f*_4_ (Outgroup, Sutherland; Test, Khomani_San.DG) demonstrates that the Sutherland individuals share more alleles with the ǂKhomani San <1240k> than with any other present-day population (S30a Fig). Reversely, the same applies to the ǂKhomani San <1240k> population (Outgroup, Khomani_San.DG; Test, Sutherland). However, the statistic is not significant for both Stone Age South Africans and ǂKhomani San <HO>, suggesting equal phylogenetic relationship. This implies that ǂKhomani San <HO> and ǂKhomani San <1240k> indeed share more evolutionary history between them than with any other contemporary human population, *i.e.* they represent the same population. However, there seems to be strong heterogeneity and regional differences in genomic composition within the ǂKhomani San population not explainable by assuming a simple isolation-by-distance model.

The statistic *f*_4_ (Outgroup; Test, Khomani_San.DG, Sutherland) was calculated to explore possible differentiating gene flow into these populations. Although, most African populations are equally related to the Sutherland and ǂKhomani San <1240k> population, several populations break the assumed phylogeny. While the Mandenka, ǂHoan, Taa (Taa_North, Taa_East, Taa_West) and ǂKhomani San <HO> share significantly more alleles with the ǂKhomani San <1240k>, the Nama share more alleles with the Sutherland individuals than with the ǂKhomani San <1240k> (S30b Fig).

To test if the Sutherland individuals show asymmetrical relationship to ancient western and eastern San and/or Khoekhoe of South Africa, the published Later Stone Age (LSA) genomes from the western (St. Helena and Faraoskop) and eastern (Ballito Bay and Doonside in KwaZulu-Natal) parts of the country were grouped into two corresponding meta-populations: western LSA South Africans and eastern LSA South Africans. Subsequently, a *f*_4_ statistic of the form (Outgroup; Test; ancient West, ancient East) was calculated (S31 Fig). As expected, most present-day African populations are equally related to both western and eastern LSA South Africans. Interestingly, however, some southern San exhibit significantly stronger affinities to western LSA South Africans than to eastern LSA South Africans. They comprise the ǂKhomani San <HO> and ǂKhomani San <1240k>, the Nama as well as the Sutherland individuals, and a ~1200-year-old South African pastoralist. Although these results must be interpreted with caution, they add to the observation that the San and/or Khoekhoe of the southern Kalahari (despite not belonging to the same language families) share a deeply related ancestry component.

### S6.5.3 qpAdm modelling & admixture graph fitting.

Regarding the non-San and/or Khoekhoe admixture component within the Sutherland sample, formal ancestry modelling using *qpADM* was performed following the approach of Skoglund and colleagues [136]. As some other San and/or Khoekhoe groups, the Sutherland individuals can be modelled as simple two-way admixture model including South_Africa_2000BP.SG as proxy for Indigenous South African ancestry and Tanzania_Luxmanda_3100BP as proxy for incoming eastern African/Eurasian pastoralist ancestry^19^. The amount of non-Indigenous South African ancestry within the Sutherland individuals (11.2 %) is comparable to other less admixed populations like Khomani San <1240k> (8.7 %), Ju_hoan_North <HO> (11.9 %), Taa_West (12.4 %), Taa_East (15.5 %), or /Gui (16.5 %). Very similar results can be produced using the present-day Dinka as second source instead of Tanzania_Luxmanda_3100BP: Sutherland (16.9%), Khomani San <1240k> (13.8 %), Ju_hoan_North <HO> (19.1%) (S32 Fig, S1F Dataset).

Based on these results, the relationships between populations were modelled in a simple admixture graph framework with the ADMIXTOOLS 2 R package (<https://github.com/uqrmaie1/admixtools>) using a subset of the 1240k dataset that includes the three Sutherland individuals, four LSA South Africans older than 2000 BP, Tanzania_Luxmanda_3100BP.SG, present-day Yoruba, and a Neanderthal and Denisovan genome together as outgroup (labelled here as “Archaic”). The find_graphs function was used with the parameters numgraphs = 100, numgen = 100, max_admix = 5, numsel = 5, stop_gen2 = 20 and subsequently the best fitting model with a likelihood score of 312.45 was extracted. Corresponding with the previously computed *f*_4_ and *qpAdm* results, the Sutherland individuals are placed as sister clade to western LSA South Africans. Further, we estimate 12 % Tanzania_Luxmanda_3100BP.SG-related ancestry within the three Sutherland individuals (S33 Fig). Additionally, a second admixture graph was computed by adding Ju_hoan_North <HO> (a Kx’a-speaking San population without major non-San and/or Khoekhoe admixture which is equally related to ancient South Africans from the East and the West), allowing for two admixture events. In congruency with the previously calculated *f*_4_ statistics, the Ju_hoan_North <HO> individuals trace their Indigenous South African ancestry to a population that diverged before the split of western and eastern LSA South Africans (S33b Fig). This contrasts with the branching of the Sutherland individuals that are asymmetrically related to those ancient populations, sharing more genetic drift with ancient western South African individuals.

## S6.6. Genetic analyses methodology

### S8.4.1 Informed consent and ethics

Stringent informed consent procedures were adhered to, as outlined by Gibbon [8], which go above and beyond the current legislative requirements of the South African Heritage Resource Agency (SAHRA) and meet the requirements of the Department of Health for extracting DNA from living persons in accordance with international guidelines [148]. A materials transfer agreement was implemented between the Max Planck Institute for the Science of Human History in Jena, Germany and UCT. Permission from the Human Ethics Research Committee at the University of Cape Town and informed consent were obtained before commencing with the DNA project (as described earlier in Section S2). Any tissue remaining after the DNA analyses was returned to UCT and reunited with the rest of the remains.

Due to the unique situation with named descendants, these genetic data are not open access. In accordance with the informed consent process, access to the new genetic data described here may be possible with permission from the Abraham and Stuurman families in Sutherland. Users who would like to apply for access should contact the Curator of the UCT Human Skeletal Repository (http://www.anatomybioanth.uct.ac.za/uct-human-skeletal-collection) with a document detailing:

i) A project description and motivation.

ii) Main applicant's name, affiliation and short CV detailing the applicant’s scientific qualifications for handling human genetic data.

iii) A list of project members who will be given direct access to the data and their names, affiliations and roles in the project.

iv) A data protection plan, including details of how access will be limited to the individuals listed in (iii).

### S8.4.2 Sampling, DNA extraction, sequencing, and contamination assessment.

Genetic sampling for the Sutherland Nine took place in a clean-room facility dedicated to ancient DNA work at the Max Planck Institute for Science of Human History in Jena. The pre-sampling workflow included documenting and photographing the tooth samples provided. Samples were then irradiated with ultraviolet light for 30 min on all sides. Prepared teeth were cut along the cementum/enamel junction, and powder was collected by drilling into the pulp chamber [149].

Ancient DNA was extracted from dentine powder following a modified protocol described by Dabney et al. 2013 [150,151]. Twelve double-stranded libraries were built with unique index pairs following modified protocols by Meyer & Kircher [152] and Kircher et al. 2012 [153-155]. A partial uracil-DNA-glycosylase protocol was applied to remove most of the aDNA damage while preserving the characteristic damage pattern in the terminal nucleotides as described in Rohland et al. [156].

All indexed libraries were first screened via shotgun sequencing of 7 million reads on an Illumina HiSeq 4000 sequencer using a single [1 × 75–base pair (bp) reads] kit. Subsequently, all libraries were hybridized in-solution to different oligonucleotide probe sets synthesized by Agilent Technologies to enrich for sequences that overlap approximately 1,24 million informative nuclear single nucleotide polymorphisms. For each of the captured libraries 58 million reads were sequenced (1 × 75–base pair (bp) reads) on an Illumina HiSeq 4000 platform at the Max Planck Institute for the Science of Human History in Jena. Out of the nine originally processed Sutherland samples, three [Saartje (UCT 43/SUT003), Klaas (UCT 50/SUT006), and Jannetje (UCT 54/SUT009)] proved to be of a quality adequate for use in downstream autosomal analyses.

After demultiplexing, raw sequence data were processed using EAGER [157]. This included clipping sequencing adaptors from reads with AdapterRemoval v.2.3.0 (<https://github.com/MikkelSchubert/adapterremoval>) and mapping of reads with BWA (Burrows-Wheeler Aligner) [158] v.0.7.12 (<https://sourceforge.net/projects/bio-bwa/files>) against the Human Reference Genome hg19, with seed length (-l) disabled, max number of differences (-n) of 0.01 and a quality filter (-q) of 30. Removing duplicate reads with the same orientation and start and end positions was performed using DeDup v.0.12.1. Terminal base deamination damage calculation was done using mapDamage [159] v.2.0.6, specifying a length (-l) of 100 bp. We used BamUtil v.1.0.13 (<https://github.com/statgen/bamUtil.git>) to clip two bases at the start and end of all reads for each sample to remove residual deaminations.

To determine the genetic sex of each ancient individual we calculated the coverage on the autosomes as well as on each sex chromosome. A custom script (<https://github.com/TCLamnidis/Sex.DetERRmine>) [160] was used for the calculation of each relative coverage as well as their associated error bars. According to Lamnidis and colleagues, females are expected to have an x-rate of 1 and a y-rate of 0, while males are expected to have both x- and y-rate of 0.5 [161] (S20 Fig).

Contamination estimation was accomplished using the Analysis of Next Generation Sequencing Data package [161] v.0.910 was used to test for heterozygosity of polymorphic sites on the X chromosome in male individuals, applying a contamination threshold of 5%. For male and female samples, contamination levels were estimated using Schmutzi [162]by comparing the consensus mitogenome of the ancient sample to a panel of 197 worldwide mitogenomes as a potential contamination source applying a contamination threshold of 5% as well.

The program pileupCaller from sequenceTools v.1.4.0.2 (<https://github.com/stschiff/sequenceTools.git>) was used to genotype the three individuals mentioned earlier (Saartje, Klaas, and Jannetje). A pileup file was generated using samtools v1.3.1 mpileup with parameters -q 30 -Q 30 -B containing only sites overlapping with our capture panel. From this file, for each individual and each SNP on the 1240k panel, one read covering the SNP was drawn at random, and a pseudohaploid call was made.

To process mitochondrial DNA data, reads from 1240k data were extracted using samtools [163] v.1.3.1 and mapped to the revised Cambridge reference sequence. Subsequently, consensus sequences were generated using Geneious [164] R8.1.974 and loaded into HaploGrep 2 [165] to determine mitochondrial haplotypes. For the male individuals, Y chromosome haplogroups were manually assigned using pileups of Y-SNPs included in the 1240k panel that overlap with SNPs included on the International Society of Genetic Genealogy SNP index v.14.07.

Kinship analysis was achieved with the degree of genetic relatedness between the Sutherland individuals estimated by applying relationship estimation from ancient DNA [166] (<https://bitbucket.org/tguenther/read>).

### S8.4.3 Population genetic analysis

*Merging new individuals with published data.* Four datasets were created for genome-wide analysis. First, the new Sutherland data of Saartje (UCT 43/SUT003), Klaas (UCT 50/SUT006), and Jannetje (UCT 54/SUT009) were merged with published ancient data (136-138,167-171) to the HO panel (~600 K SNPs) [167,172,173]. This dataset was used for PCA, Model-Based Clustering and *f*-Statistics (see below). The Sutherland data was also merged with published ancient data to the 1240k SNP panel including 300 present-day individuals from 142 populations sequenced to high coverage (~1200 K SNPs) [174]. This second dataset, restricted to the autosomes, was used for all population genetic analyses that comprised only ancient genomes. The third dataset comprises the Sutherland data, the published ancient data, the 300 present-day individuals from 1240k SNP panel, and 228 present-day individuals from 15 populations published by Schlebusch et al. [146,175] (~540 K SNPs). The fourth dataset included the Sutherland data, the published ancient data, the HO panel present-day individuals as well as the present-day individuals from Schlebusch et al. [172,173] (~210 K SNPs) (S1D Dataset & S5).

*Principal components analysis.* We performed PCA using the smartpca program ^[^147] v.16000 from EIGENSOFT (<https://github.com/DReichLab/EIG>) on the HO panel, 1240k, and the Schlebusch-1240k datasets on which ancient individuals were then projected using the options lsqproject: YES and shrinkmode: YES. The naming of the populations follows the established standard of the Allen ancient DNA resource (<https://reichdata.hms.harvard.edu/pub/datasets/amh_repo/curated_releases/index.html>). More information about populations and their naming can be found in Dataset S1E.

For the African HO PCA, the following populations were used to construct principal components: Mende, Mandenka, Yoruba, Esan, Gambian, Masai, Luo, Dinka, Datog, Kikuyu, Luhya, Hadza, Hadza1, Sandawe, Mbuti, Biaka, BantuSA, BantuSA_Ovambo, BantuSA_Herero, BantuKenya, BantuTswana.DG, Himba, Wambo, Tswana, Kgalagadi, Damara, Nama, Gui, Gana, Shua, Haiom, Naro, Khwe, Ju_hoan_North, Ju_hoan_South, Taa_North, Taa_East, Taa_West, Khomani <HO>, Khomani_San.DG <1240k>, Xuun, Hoan.

For the South African HO PCA, the following populations were used to construct principal components: Himba, Wambo, Dinka, Yoruba, BantuSA, BantuSA_Ovambo, BantuSA_Herero, BantuKenya, Mbukushu, Mandenka, Tswana, Kgalagadi, Naro, Haiom, Khwe, Shua, Tshwa, Gana, Gui, Nama, Damara, Ju_hoan_North, Ju_hoan_South, Xuun, Hoan, Taa_West, Taa_North, Taa_East, Khomani <HO>, Khomani_San.DG <1240k>.

For the Schlebusch-1240k PCA, the following populations were used to construct principal components: Ju_hoan [Juhoansi], Xuun [Xun], Khwe, Gui, Gana, Kgalagadi [GuiGhanaKgal], Nama, Karretjie, Khomani.

*Maximum likelihood tree model.* The three Sutherland genomes were used together with four ca. 2000 year old South Africans as well as present-day genomes from African populations in the Simons Genome Diversity project^35^ to reconstruct a maximum likelihood tree using Treemix(176) v1.12 (<https://bitbucket.org/nygcresearch/treemix/wiki/Home>) as described in Skoglund et al. [136]. To assess the uncertainty of the fitted model, 100 bootstrap replicates were performed. 100% bootstrap support was found for the Sutherland samples being most closely related to the present-day ǂKhomani San (Khomani_San.DG from 1240k) and ancient South Africans (South_Africa_2000BP.SG) (S24 Fig).

*Model-based clustering.* ADMIXTURE (177) (<https://www.genetics.ucla.edu/software/admixture/download.html>) was run with version 1.3.0, following exclusion of variants with minor allele frequency of 0.01 and after linkage disequilibrium runing using plink (version 1.90b3.29) with a window size of 200, a step size of 25 and an R^2^ threshold of 0.5 [according to 178]. Five replicates were run for each K value, with K values ranging between 2 and 20 (S25 Fig).

*f-statistics.* Outgroup *f*_3_-statistics were calculated using qp3Pop v.435 and *f*_4_-statistics using qpDstat v.755 with the f_4_ mode from ADMIXTOOLS [179] (<https://github.com/DReichLab/AdmixTools>) on the 1240k, HO, Schlebusch-1240k, and Schlebusch-HO SNP panels. Standard errors were computed using the default block jackknife approach. Two standard errors are reported and plotted for the F_ST_, *f*_3_- and *f*_4_-statistics in the supplementary figures. Chimp.REF was used as outgroup in all computed statistics.

*qpAdm modelling and admixture graph fitting.* qpAdm v.810 in ADMIXTOOLS [179] and the HO dataset SNP panel were used to estimate ancestry proportions with respect to a basic set of 13 outgroups: Jordan_PPNB, Anatolian_Neolithic, Iran_Ganj_Dareh_Neolithic, Denisova_published.DG, Ust_Ishim_HG_published.DG, Georgian.DG, Iranian.DG, Greek.DG, Punjabi.DG, Orcadian.DG, Ami.DG, and Mixe.DG [136].

Admixture graphs were calculated with the ADMIXTOOLS 2 R package (<https://github.com/uqrmaie1/admixtools>) using a subset of the 1240k dataset that includes the three Sutherland individuals, four LSA South Africans older than 2000 BP, Tanzania_Luxmanda_3100BP.SG, present-day Yoruba, and a Neanderthal and Denisovan genome together as outgroup (labelled here as “Archaic”). The find_graphs function was used with the parameters numgraphs = 100, numgen = 100, max_admix = 5, numsel = 5, stop_gen2 = 20 and subsequently the best fitting model with the lowest likelihood score was extracted.

# S7. Stable isotope analyses

Sutherland lies close to the boundary between winter and summer rainfall zones of South Africa (see main text Fig 1). Rain falls mainly in winter. The area is dry, supporting sparse Karoo shrubland vegetation, with grass appearing after rain. In South Africa, dicotyledonous plants are nearly all C_3_ while grasses may be C_3_ (mainly in winter rainfall regions) or C_4_ (in summer rainfall regions) [180, see also 181]. Because the area is warm and dry, δ^13^C values (see methods section below for definition) of C_3_ plants are expected to be more positive than the global mean [182,183]. The current best estimate for the global mean is -28.77 ± 2.68‰, n = 3478 [184], although values were approximately 2‰ more positive in the 19^th^ century due to recent intensive burning of fossil fuels [185]. δ^13^C of consumer tissues reflect primarily the proportions of C_3_ and C_4_ plants at the base of the foodweb. ^15^N/^14^N is higher in arid regions, and (to a lesser extent) in individuals heavily dependent on animal foods (meat and/or milk). Dietary insufficiency (starvation) also leads to elevated ^15^N/^14^N, as the body breaks down and re-uses its own tissue [186,187]. Marine foods, which also have high ^13^C/^12^C and ^15^N/^14^N, are not relevant here.

We report analyses of δ^13^C and δ^15^N in bone collagen, which integrates the isotopic signal of diet over many years of life, and dentine collagen, which reflects diet at the time of tooth formation. Analysis of serial samples of dentine from the occlusal surface to the root tip tracks diet over the period of tooth formation [186]. Since collagen is a protein tissue, the record it preserves is biased towards the protein component of the diet (188,189). We also report analyses of δ^13^C and δ^18^O in tooth enamel; δ^13^C_enamel_ provides a better index of proteins, carbohydrates and fats in the diet. We consider differences in δ^13^C and δ^15^N of consumer tissues ≥ 2‰ as likely to indicate shifts in diet or environment (*i.e.* place of residence); smaller changes may be due to metabolic variation [190,191]. Dietary or residential transitions between foods or localities with similar δ^13^C and δ^15^N values cannot be detected using the methods employed here. A total of 79 samples of bone and dentine collagen and seven samples of tooth enamel were analyzed for this study. δ^13^C values of bone collagen from the eight historic Sutherland skeletons (mean: -17.7 ± 0.6‰, n=8, Igue We excluded) reflect a predominantly C_3_ diet. δ^15^N values (mean: 14.6 ± 0.8‰, n=8) indicate an arid environment and consumption of animal-derived foods, as documented in ethnographic and historical sources. These δ^13^C values are very similar to those reported for late Holocene skeletons from the Clanwilliam District of the Western Cape, approximately 170 km to the west (mean: -18.0 ± 1.0‰, n=10, all values for bone) [192]. Clanwilliam lies within the winter rainfall zone, where the great majority of grasses are C_3_ [180]. If values for dentine samples from Sutherland are grouped with bone collagen, the picture shifts only slightly (mean δ^13^C: -17.1 ± 1.2‰, n = 69, mean δ^15^N: 15.4 ± 1.1‰, n = 69, Igue We excluded).

Igue We (UCT 31) is a clear outlier in both δ^13^C and δ^15^N. Most values for his first molar plot at the positive δ^13^C/ high δ^15^N end of the range reported here (main text Fig. 5). It is possible that the climate and environment around Sutherland in the 14^th^ century CE was different from that during the second half of the 19^th^ and early 20^th^ centuries, when the other individuals lived there. These values are, however, substantially different, so it is more likely that Igue We spent his early childhood in a drier area with a greater proportion of summer rainfall, perhaps the region to the north, towards the Orange River. Values for the latest-forming M1 dentine, at age approximately 8-9 years, show lower δ^15^N values and markedly more negative δ^13^C values (-13.7 and -13.3‰ for the root tip and adjacent sample, compared with -10.0 ± 0.7‰ for the other 7 samples) (S2 Dataset, S34 Fig). The degree of variation (range of 5.3‰ in δ^13^C and 2.9‰ in δ^15^N within the first molar, the latter increasing to 3.3‰ if the second molar is included) constitute strong evidence for dietary change and/or residential mobility during the period of tooth formation.

This is true also for Klaas, with a range of 4.2‰ in δ^13^C and 3.4‰ in δ^15^N in his first and second molars. There is a marked increase in δ^13^C (from -17.4 to -13.8‰) over the period of formation of his first molar crown and the upper part of the root. Values then remain similar (between -13.8 and -15.4‰) until approximately 3 mm below the DEJ of his second molar, with δ^13^C of -16.8‰, after which values remain in the range -16.6 to -17.8‰ as far as the root tip of the M2 (S34 Fig). δ^15^N increases during the period of growth of the first molar (except for the root tip), decreases in the second molar and then shows a slight increase towards the root tip of the second molar. The pattern of variation in δ^13^C differs from that in δ^15^N. Comparison of the tip of the tooth root with bone informs of possible changes between the time of root completion and adult diet, as reflected in bone. Klaas shows a difference >3‰ between the root tip of his first molar (formed at approximately 9 years of age) (δ^13^C of -14.3‰) and his bone (-17.4‰). Values for the root tip of his second molar (formed at approximately 15 years of age) are very similar to bone (δ^13^C of -17.8 and -17.4‰, δ^15^N of 15.3 and 15.2‰ respectively). As can be seen in S2 Dataset and S34 Fig, δ^13^C and δ^15^N values of the first slice of the M2 root immediately adjacent to the DEJ fall within the range of the M1 root. In the second slice, the δ^13^C value is within the M1 range but δ^15^N is lower, and from the third slice to the root tip, both δ^13^C and δ^15^N values lie outside the values for the first molar root. δ^13^C shows more negative values, while δ^15^N is somewhat variable, but uniformly lower than the first molar root. Based on the chronological ages assigned by Beaumont and Montgomery (193), the dietary transition reflected in the second/third slice of the M2 root occurred at approximately 10 years-of-age.

In general, there is more within-individual fluctuation in δ^15^N than δ^13^C, and the patterning is less clear (S34 Fig). The two isotopes do not necessarily co-vary. This is not surprising, since δ^15^N values in plants and herbivores can be variable (194, and references therein), and there are multiple possible drivers of variation in δ^15^N in higher-level consumers. In this case, the most likely are aridity, the proportion of protein in the diet, consumption of animal-based foods, and periodic dietary insufficiency. Therefore, we are conservative in our interpretation of these results. Examination of δ^13^C and δ^15^N combined shows the patterns more clearly (main text Fig 5).

As described above, Igue We and Klaas both show a great deal of variation, indicating mobility during early childhood across a landscape with gradients in δ^13^C and δ^15^N. This is consistent with the mobile lifeways described in ethnographic studies of southern African foragers in arid inland regions, in which foraging groups range over large territories of up to 2500 km^2^ annually (summarized in 195 S2 Dataset 4.1). In addition, individuals or families may temporarily join other groups to spend time with friends or relatives, thus increasing their geographical range. By the time that Klaas was born, forager settlement patterns would have been very much disrupted by colonial encroachment, which may also have increased mobility.

The variation seen in Cornelius (range of 2.2‰ in δ^13^C, 2.6‰ in δ^15^N) and Saartje (2.9‰ in δ^15^N) may indicate some dietary or environmental variation, but on a much smaller scale than seen in Igue We and Klaas. Without more detailed characterization of the environment, it is difficult to know how much significance to attach to these differences. For Jannetje, within-individual variation in both δ^13^C and δ^15^N is less than 2‰.

The analytical results are consistent with the archival information that Klaas came from the area between Sutherland and Carnarvon, which is farther into the summer rainfall region of South Africa (main text Fig 1) where there are more C_4_ grasses (180). He was captured and taken to Sutherland to work on the Kruisrivier farm. We can now tell that this happened when he was about 10 years old.

The archival records state that Saartje, too, was captured from a free-living San group and taken to the farm. Genetic analyses reported above show that she and Klaas were second-degree relatives (*e.g.* half siblings or double cousins). If the archival records are correct, we can infer from the isotopic analyses that during her early life (pre-capture), she did not live in the same [higher summer rainfall] area as Klaas, so she came from a different group or band. She shows much less isotopic variation across her first incisor, third molar and rib bone (2.9‰ in δ^15^N, 1.3‰ in δ^13^C). Mean δ^13^C of -17.7 ± 0.3‰ for 18 samples of dentine and bone indicates that throughout her life, she lived in area/s of mainly winter rainfall. It is possible that the archival records are incorrect, and she lived at or near Kruisrivier throughout her life.

δ^13^C values for tooth enamel (a single analysis per tooth) vary from -5.1‰ for Igue We to -13.9‰ for Saartje’s first molar. Excluding Igue We, the range is -10.2 (for Klaas’s third molar, formed after his move to the farm) to -13.9‰, confirming the C_3_ nature of the diet. Values for Saartje’s first molar (-13.9‰) and her first incisor (-13.7‰) are within analytical error and support the inference from dentine collagen that she spent her early childhood in a C_3_ environment. δ^18^O_PDB_ values for the historic individuals range from 0.9 to -2.2‰, while that for Igue We is 2.9‰.

## S7.1 Stable isotope methodology

Bone and dentine collagen were prepared by lightly sanding the surfaces of small fragments of bone or the dentine cores of teeth (with the enamel removed) to remove surface contaminants. They were then left in a solution of 2% HCl, changed every second day, until they were decalcified, which typically took a few days to a week. The progress of decalcification was monitored by probing with a needle and continued until no hard core remained. At this stage, dentine was cut with a scalpel into slices approximately 1 mm thick, perpendicular to the long axis of the tooth. The bone collagen or dentine slices were then soaked overnight in 0.1M NaOH to remove humic contaminants. Next, they were left to soak in distilled water, changed every one or two days, until the pH remained neutral. Finally, they were freeze-dried. Approximately 0.45 mg of collagen was weighed into a tin cup and folded tightly to exclude air, then loaded into an automated Flash 2000 organic elemental analyzer set to 1020**°**C and combusted to produce CO_2_ and N_2_ gas. For all samples except UCT 24265h, i and j and 24291a-j the gases were introduced to a Delta V Plus isotope ratio mass spectrometer via a Conflo IV gas control unit, using helium as a carrier gas. Samples UCT 24265h, i and j and 24291a-j were analyzed later on a Delta XP mass spectrometer, in the same way. Bone samples were analyzed in duplicate, and the results averaged. Since only small amounts of dentine collagen were available, only a single analysis of each dentine sample was carried out. Internal laboratory standards were analyzed with each run. Total numbers of standards for all runs were: Merck gelatine = 12, new Merck gelatine = 11, ANU sucrose = 28, chocolate = 23 and valine = 34. Carbon isotope values are reported relative to Vienna PeeDee Belemnite, and nitrogen isotope values are reported relative to atmospheric nitrogen. Isotope measurements are expressed in delta notation in parts per mille (‰) calculated as δR=(R_sample_/R_standard_-1)*1000 where R = the ratio of heavy/light isotopes of the element R. The standard deviation of all measurements of each laboratory standard was <0.2‰ for both δ^13^C and δ^15^N.

For analyses of tooth enamel, 4-8 mg of enamel powder was removed from the tooth using a hand-held Dremel drill fitted with a diamond-tipped dental drill bit. Each sample spanned the height of the tooth crown, to obtain enamel representative of the entire period of crown formation. The enamel powders were placed in 1.5 ml snap-top micro-centrifuge tubes and treated with 1 ml of 1.75 v/v % sodium hypochlorite for 45 minutes [196] to remove organics. After rinsing three times with de-ionized water, each sample was treated with 0.1M hydrochloric acid for 15 minutes to remove diagenetic carbonates. Lastly, they were again rinsed three times with de-ionized water before being freeze dried.

Approximately 2 mg of each pre-treated powder was weighed into a clean, round-bottomed borosilicate glass vial with an exetainer cap, placed in a Finnigan Gas Bench II at 72°C, and flushed with helium gas. Five to seven drops of 100% phosphoric acid were manually injected into each vial and left to react for a minimum of two hours. Evolved CO_2_ was then swept in a stream of helium carrier gas through a gas chromatographic column and into a Delta Plus XP (Thermo-Finnigan) light isotope mass spectrometer. δ^13^C and δ^18^O values are reported relative to Vienna PDB via calibration curves based on in-house standard Cavendish Marble (n=8), Carrara-Z new (n=7) and NBS-18 (n=8). The standard deviation of all measurements of standard materials was less than 0.1‰ for *δ*^13^C and 0.2‰ for *δ*^18^O.

**S1 Fig. Academic record of Carel Gert Coetzee, the donor, at the University of Cape Town from 1925-1931. It gives his place of residence as Kruisrivier Farm, Sutherland.**

**
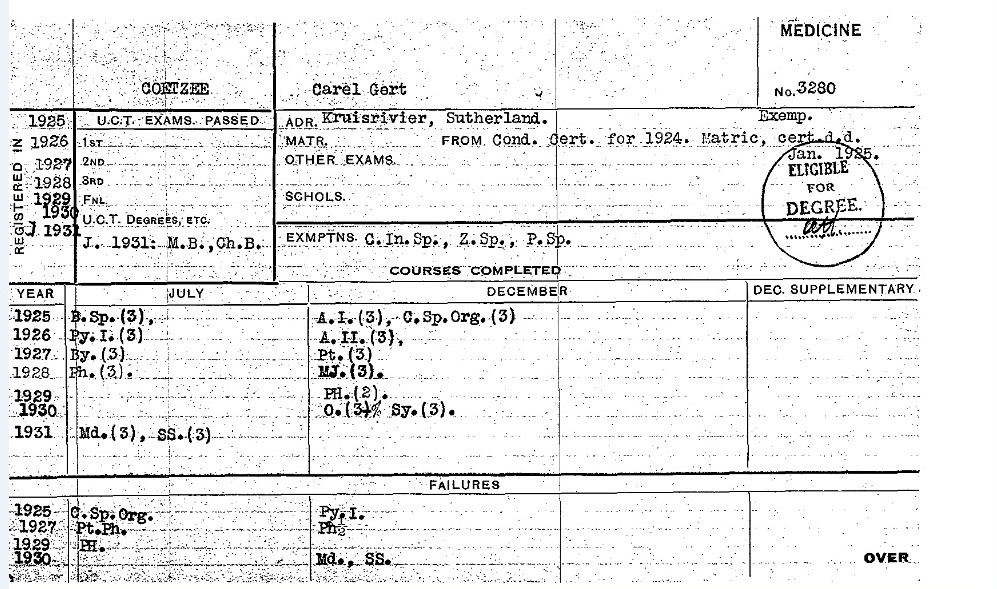
**

**S2 Fig. Aerial photo showing features on the Kruisriver farm complex including the location of the farm laborer’s cemetery. a and d both indicate threshing floors; b is the Kruisrivier school; c is an 18^th^ century archaeological deposit; e was a farm laborer dwelling; f is the Coetzee family cemetery; and g is the farm laborer’s cemetery.**


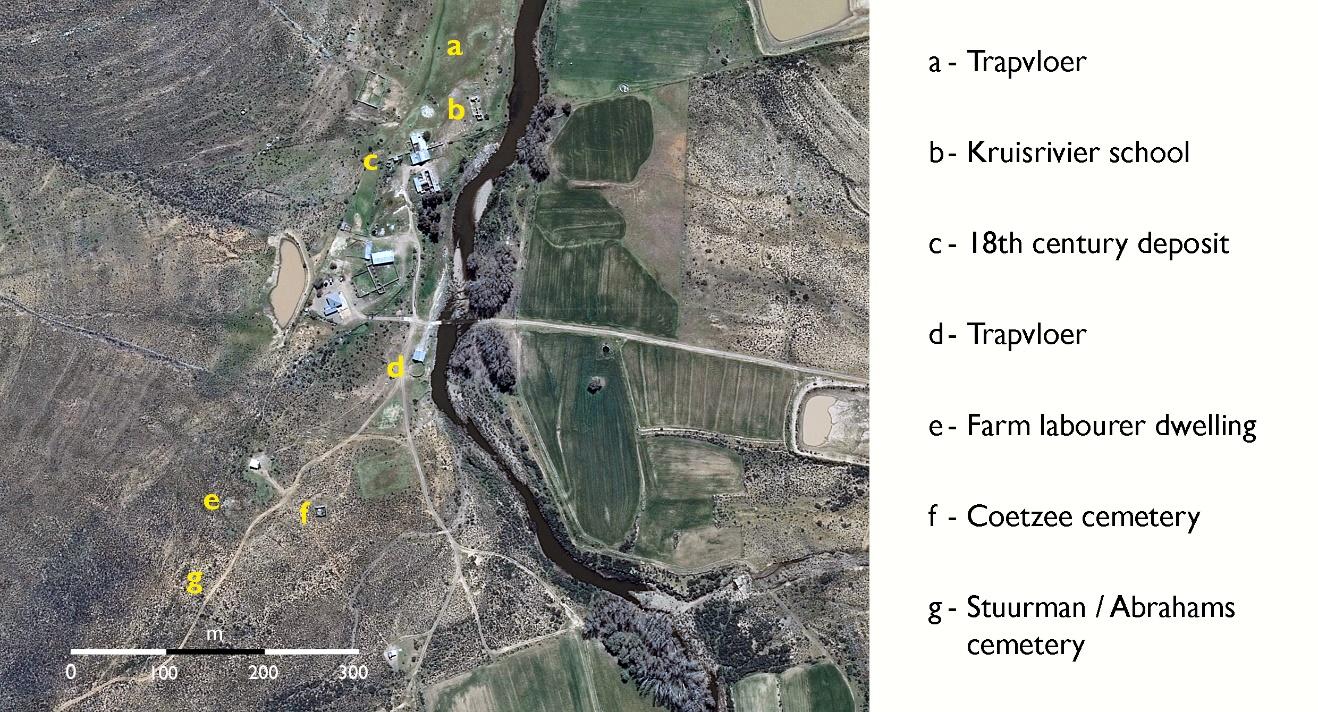


**S3 Fig. Plan of the Kruisrivier farm laborer’s cemetery. Green dots indicate the western ends of stone cairn burials (n=11), white dots the headstone (western) ends of headstone and footstone burials (n=25). Green ring indicates disturbed cairn burial, white rings disturbed headstone and footstone burials.**


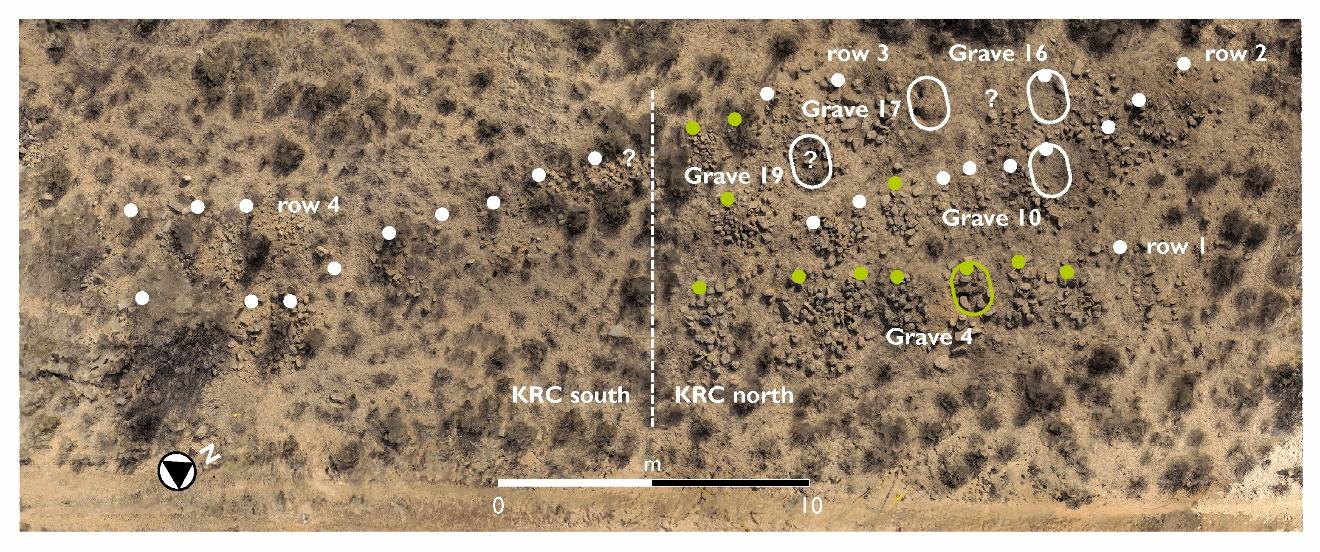


**S4 Fig. Digital 3D models illustrating cranial trauma. Top is Klaas’s cranium, illustrating the perimortem trauma. Left anterolateral view, shows entrance wound on the anterior surface of the left maxilla inferior to the orbit and medial to the left infraorbital foramen. A radiating fracture extends through the left lacrimal, ethmoid and sphenoid bones. Top right shows the inferior view, with a radiating base-of-skull fracture through the occipital bone on the left side of the foramen magnum. The bottom images are of Igue We’s cranium illustrating the perimortem trauma. Bottom left, left superolateral view, radiating fractures from the points of impact on the right are observed dissipating through to the sagittal and coronal sutures; a portion of this energy dissipates into the left parietal bone with production of a primary radiating fracture extending posteriorly, which terminates inferior to the left eurion. Bottom right, two points of impact are observed with radiating fractures.**

***
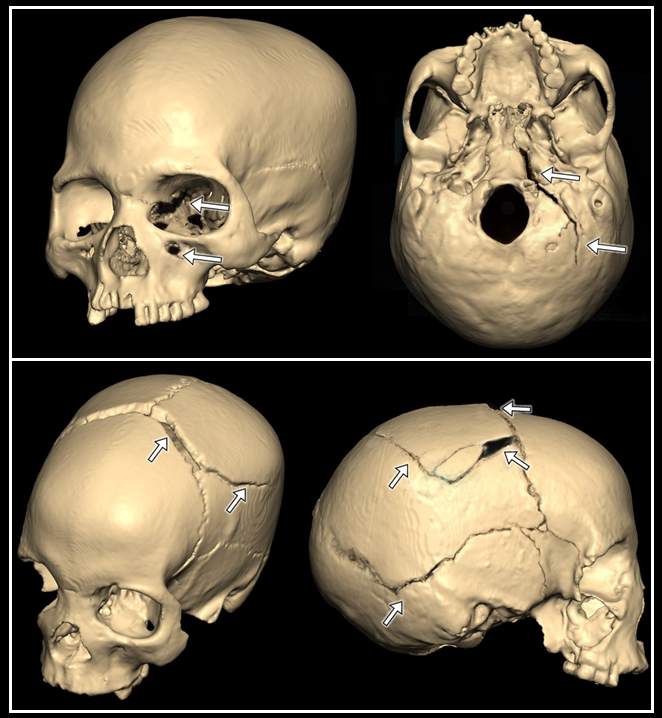
***

**S5 Fig. Tswana man wearing an ear-plate, from Burchell (1824).**


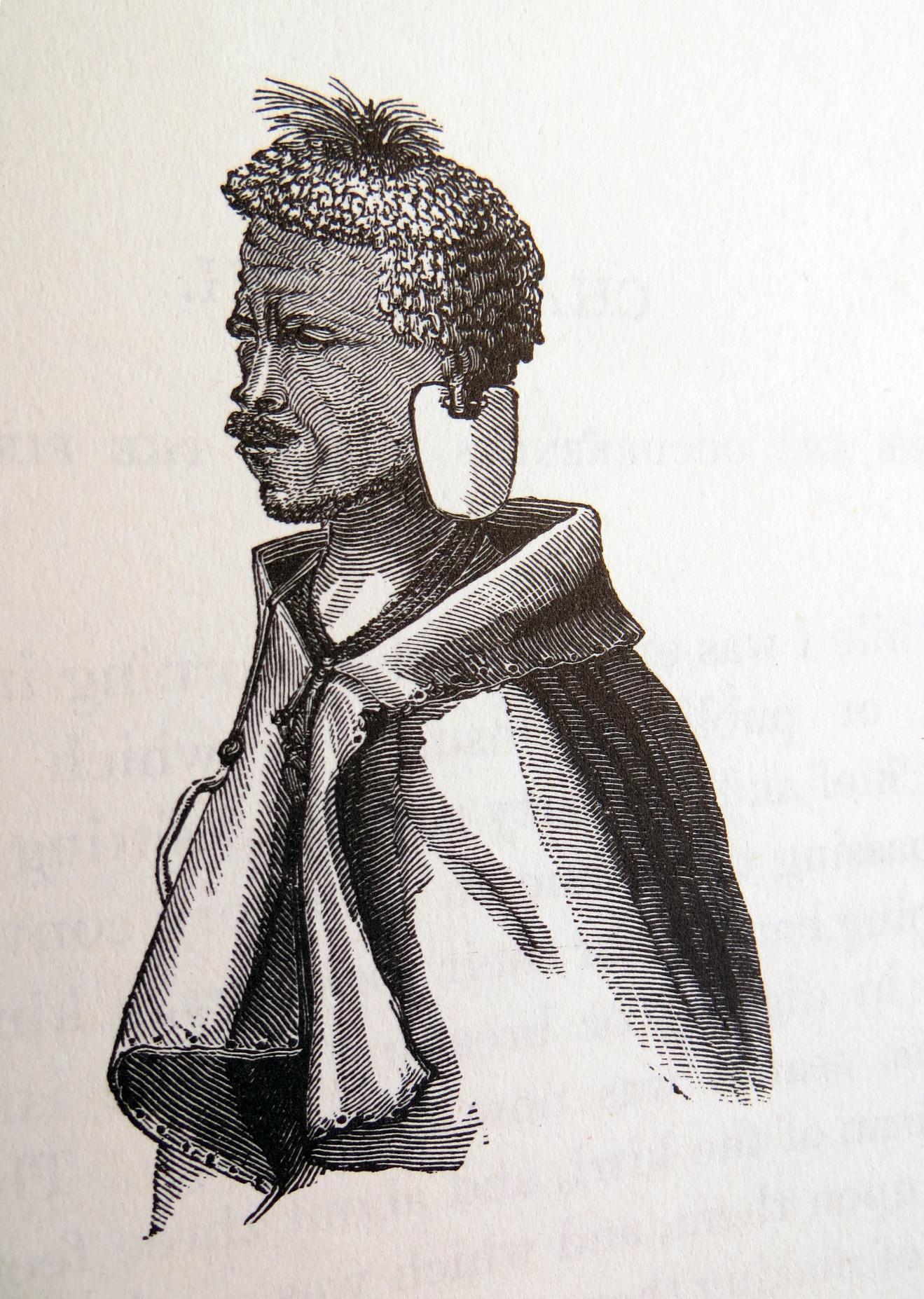


**S6 Fig. Digital 3D and 2D models illustrating the pathology of Jannetje’s cranium. An anterior left superolateral view (top left) and the 2D reconstructed CT images (bottom left, middle and right) show three major impressions in the cranium (white arrows) as a result of calvarial thinning. The Lodox images (top middle and right) illustrate the presence of osteoporosis as evidenced by a uniform decrease in radiodensity, suggesting extensive rarefaction of spongy bone. The white arrows in these two images further illustrate examples of thinning and rarefaction of cortical bone, observed as thin, faded or non-existent lines of radiodensity. Mid-sagittal reconstructed CT images (bottom middle and right) illustrate metrics evaluated for the diagnosis of platybasia and basilar invagination.**


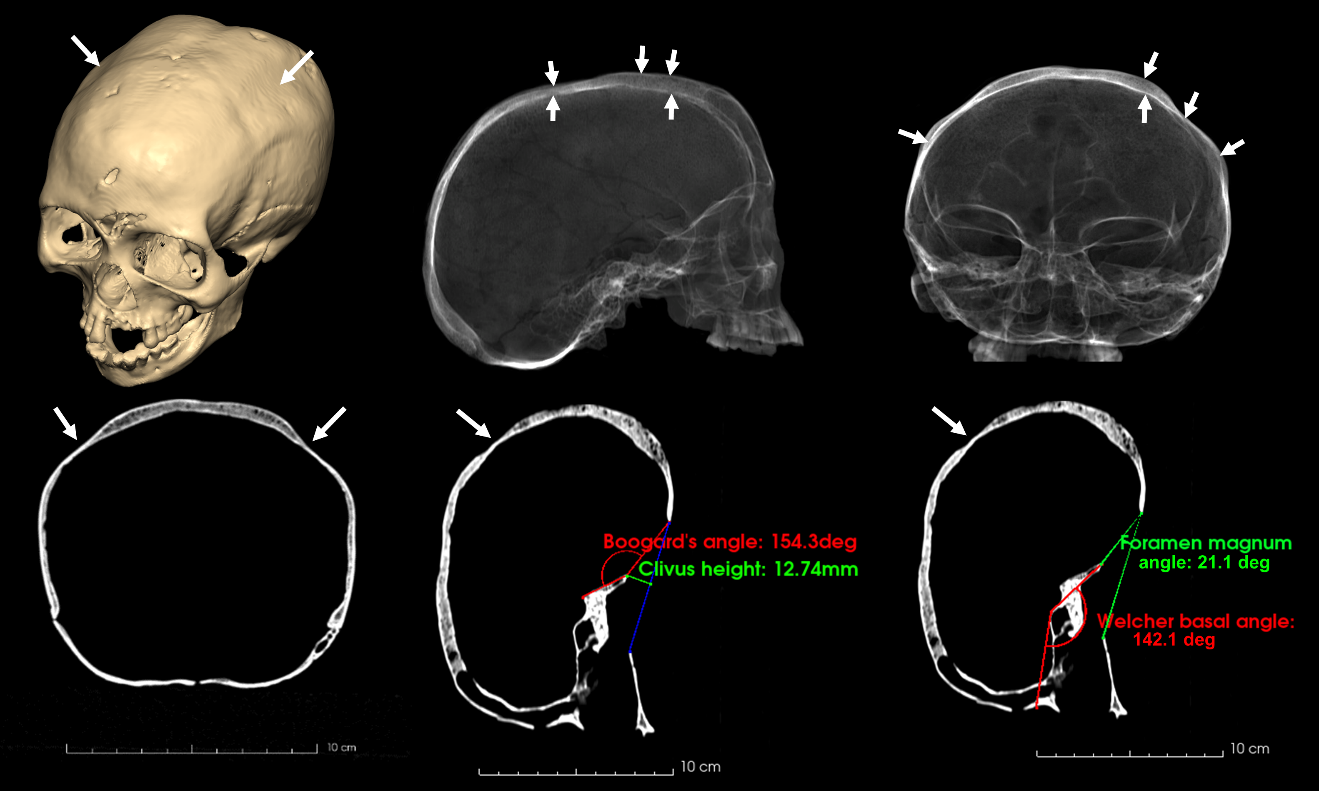


**S7 Fig.** **Process of mandible estimation for Igue We (left, showing in-filled cranial cavity), Voetje (middle) and Klaas (right).**


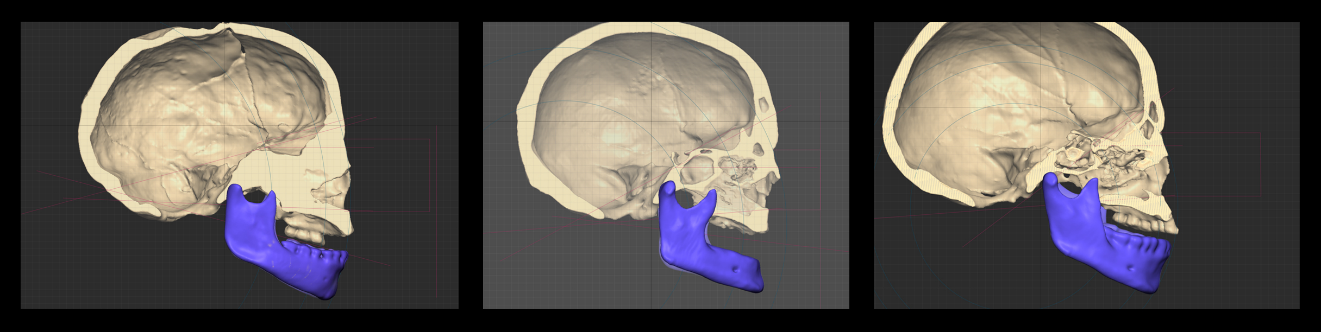


**S8 Fig. 3D cranial model of Igue We from CT data (left) and reconstructed parts (right) including realigned parietal bones and teeth lost post-mortem.**

**
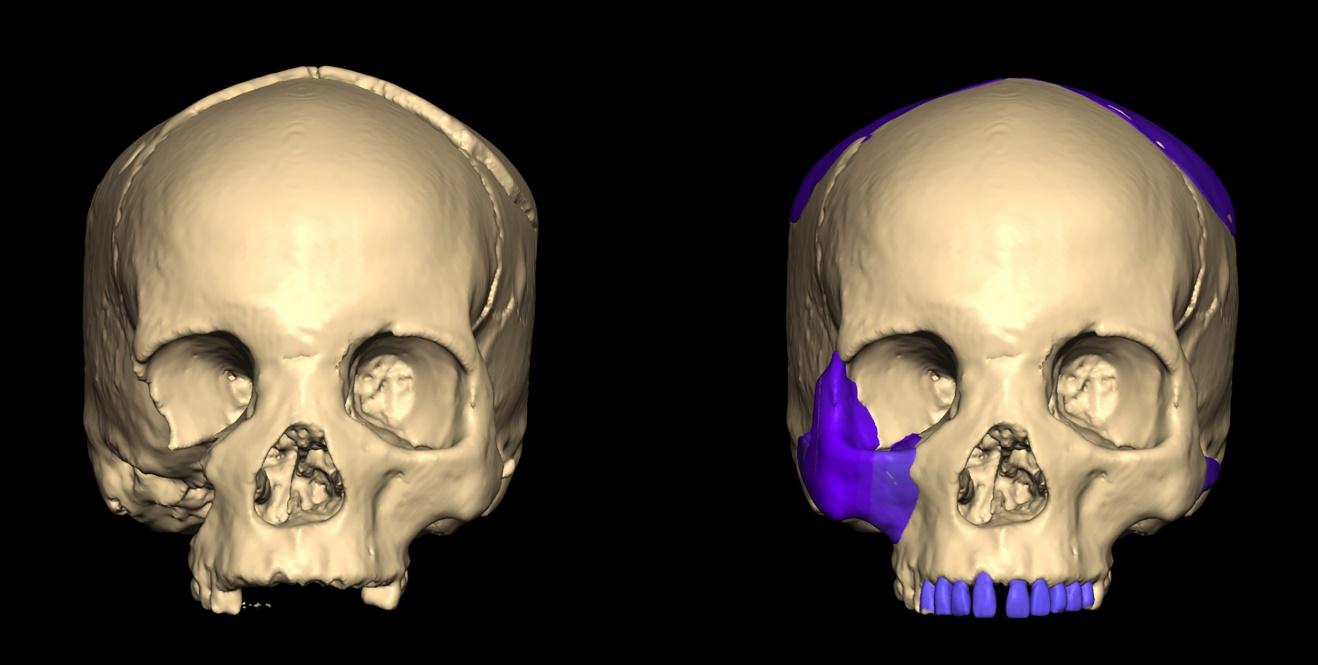
**

**S9 Fig. Process of facial reconstruction (left, middle) and final depiction (right) for Igue We.**


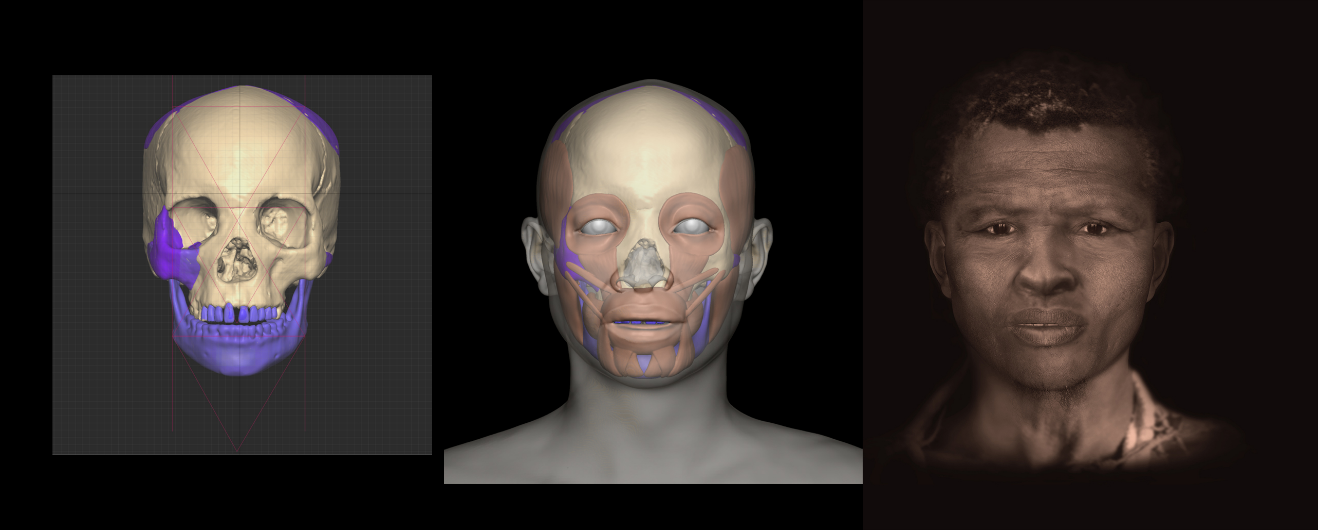


**S10 Fig. Process of facial reconstruction (left, middle) and final depiction (right) for G!ae.**


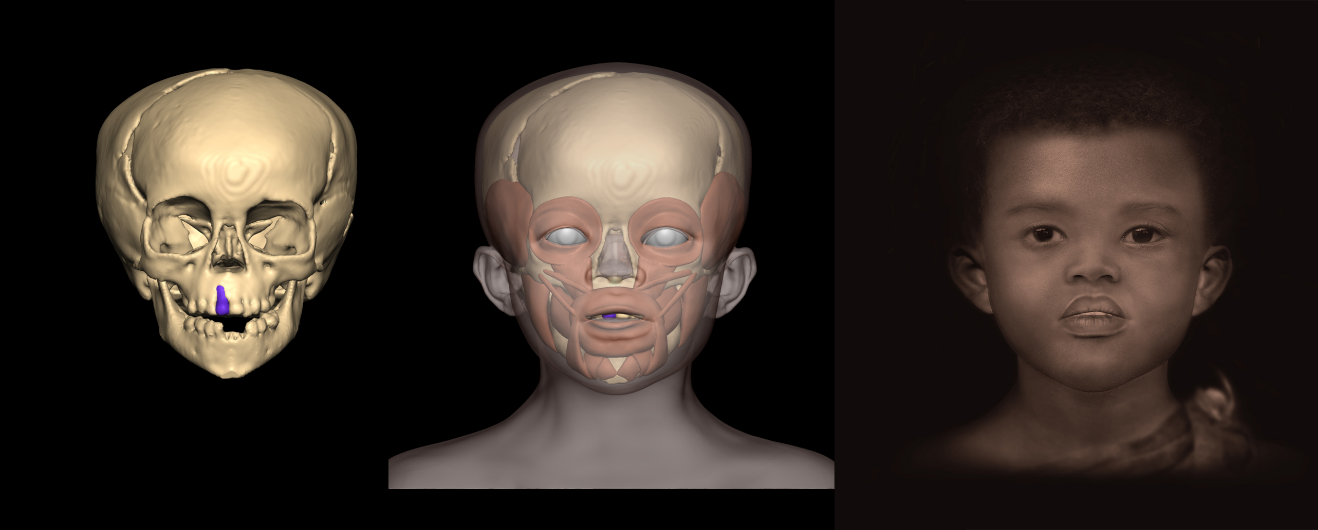


**S11 Fig. Process of facial reconstruction (left, middle) and final depiction (right) for Saa.**


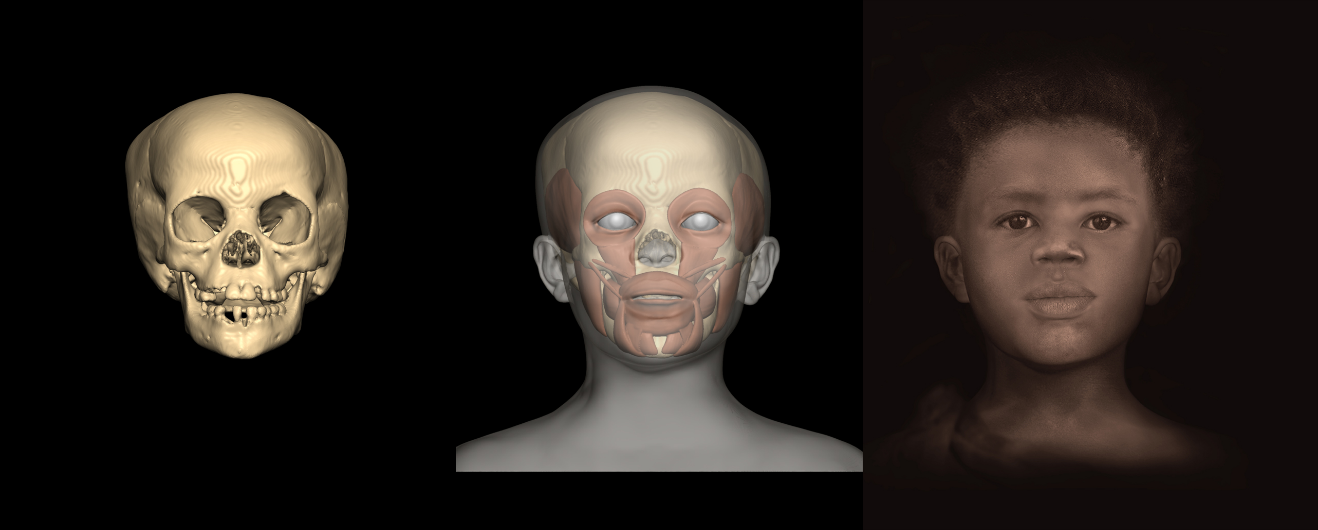


**S12 Fig. Process of facial reconstruction (left, middle) and final depiction (right) for Cornelius.**


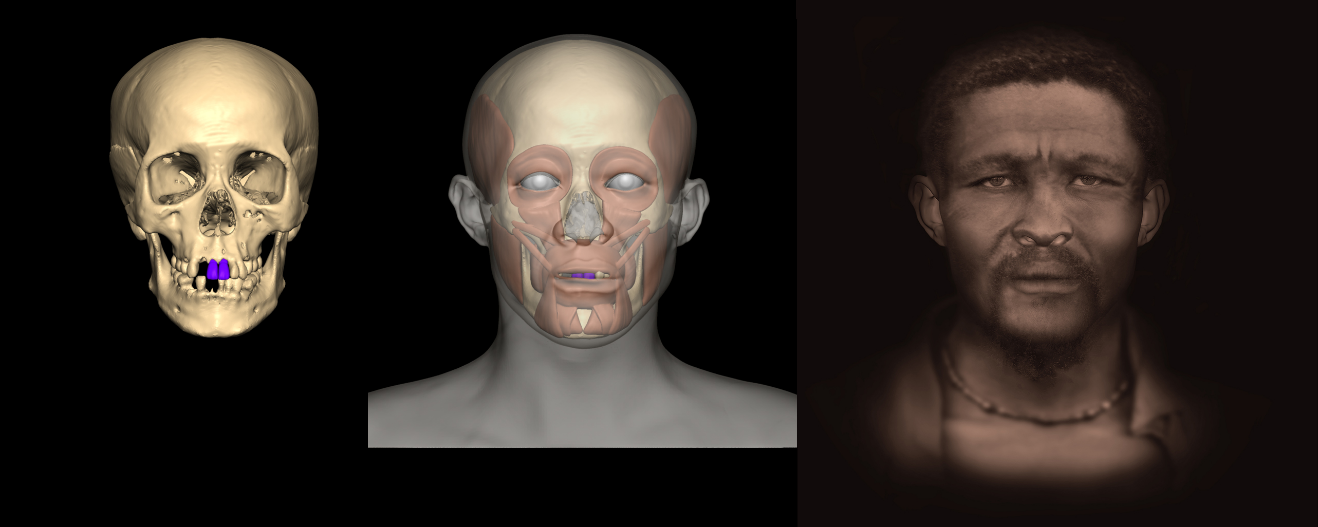


**S13 Fig. Process of facial reconstruction (left, middle) and final depiction (right) for Jannetje.**


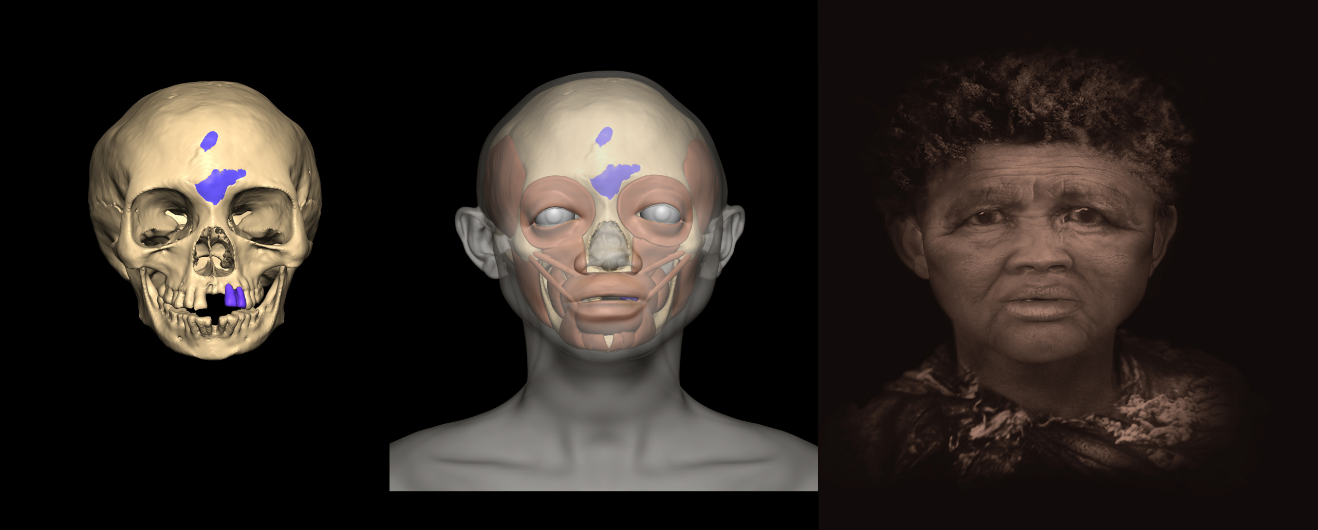


**S14 Fig. Process of facial reconstruction (left, middle) and final depiction (right) for Klaas.**


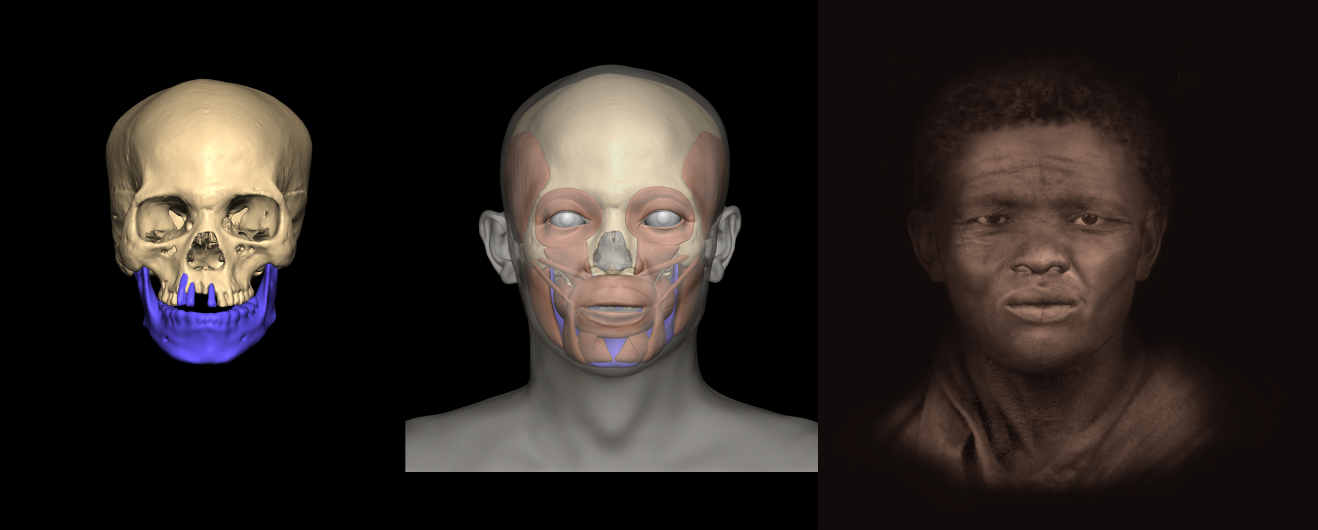


**S15 Fig. Process of facial reconstruction (left, middle) and final depiction (right) for Saartje.**


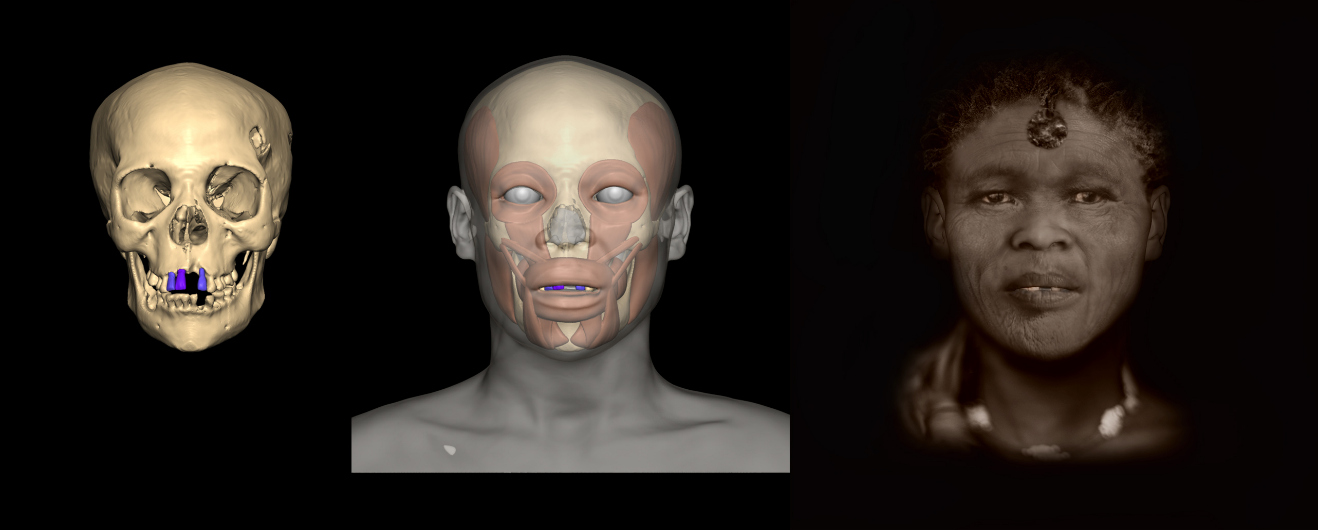


**S16 Fig. Process of facial reconstruction (left, middle) and final depiction (right) for Voetje.**

**
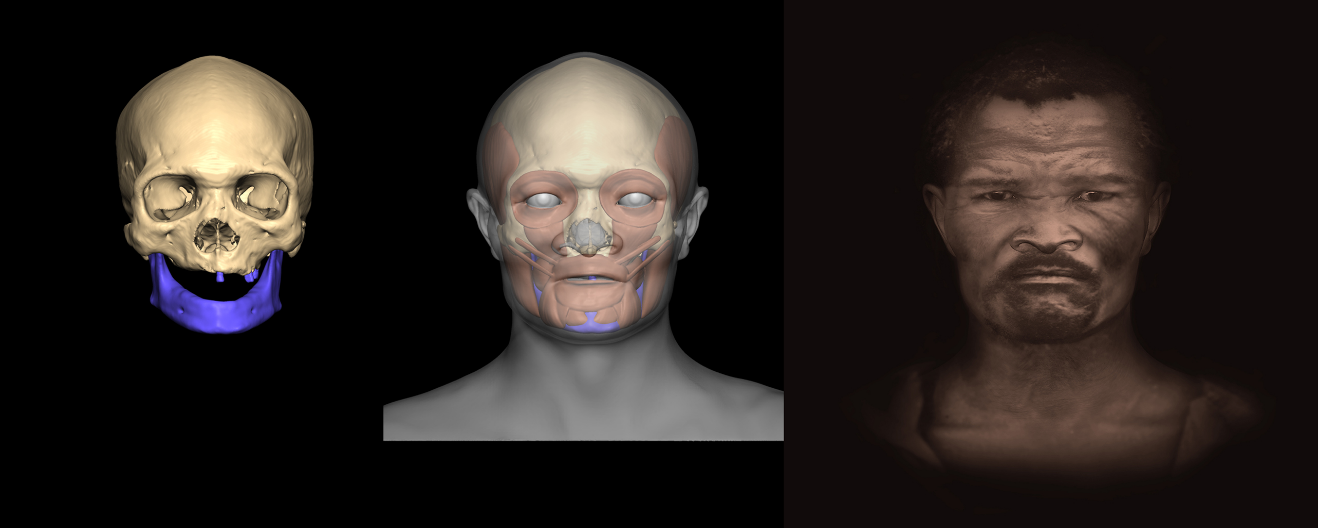
**

**S17 Fig. The facial depictions were digitally printed on wood panels (left) and presented to each family in an archival box (right).**
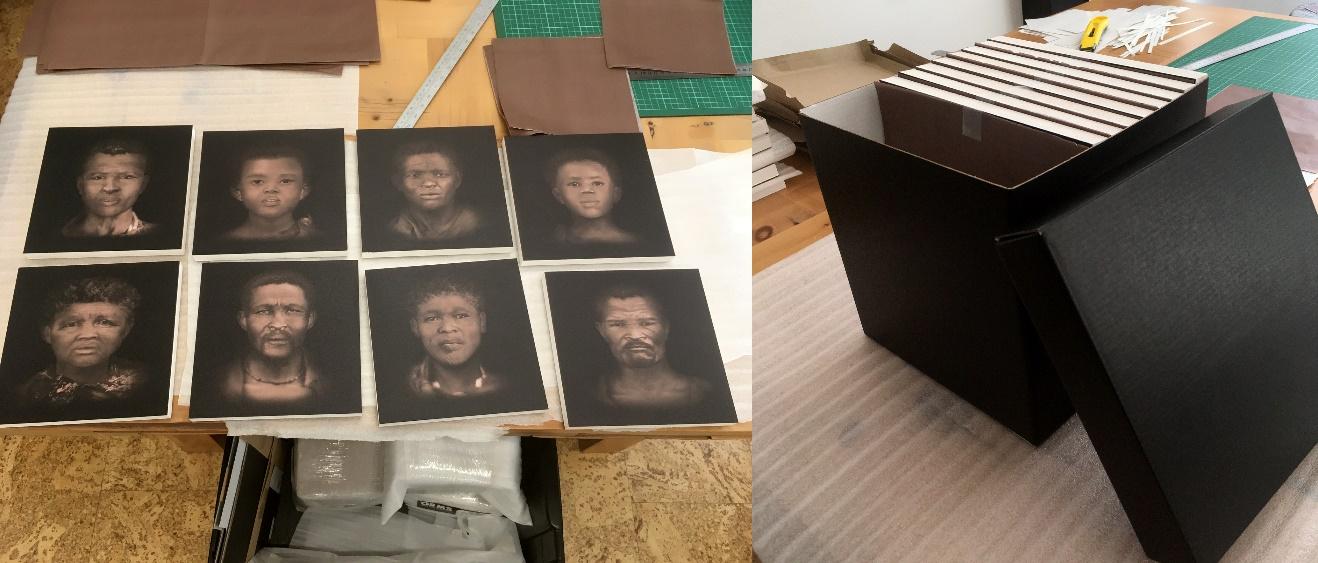


**S18 Fig. A bilingual (Afrikaans and English), illustrated facial reconstruction album visually illustrated the process alongside detailed biographical narratives of each individual. The panels and albums were presented at a knowledge sharing session for the families in Sutherland, October 2019.**


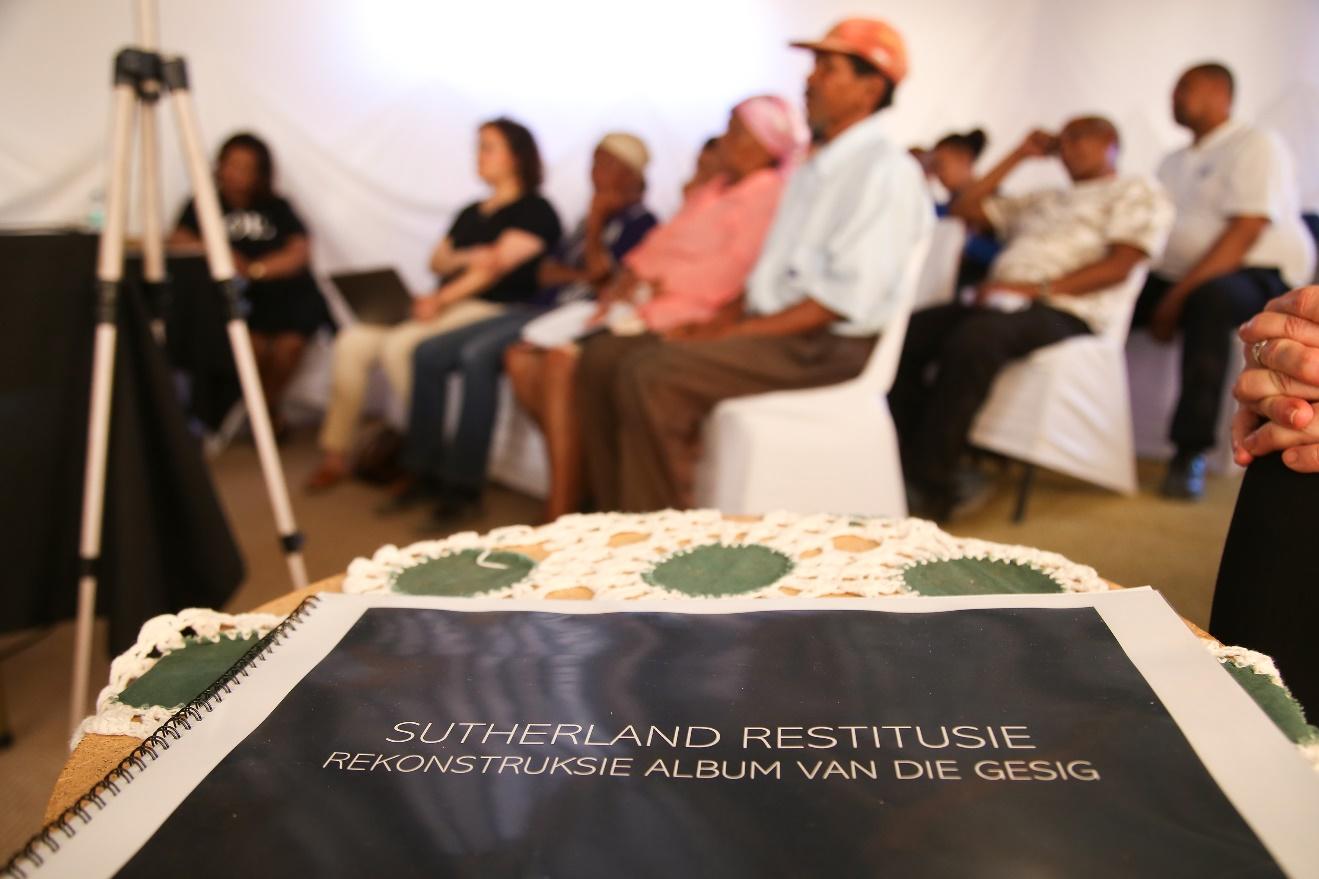


**S19 Fig.** **3D mesh surface model formation from CT images for Klaas’s cranium.**


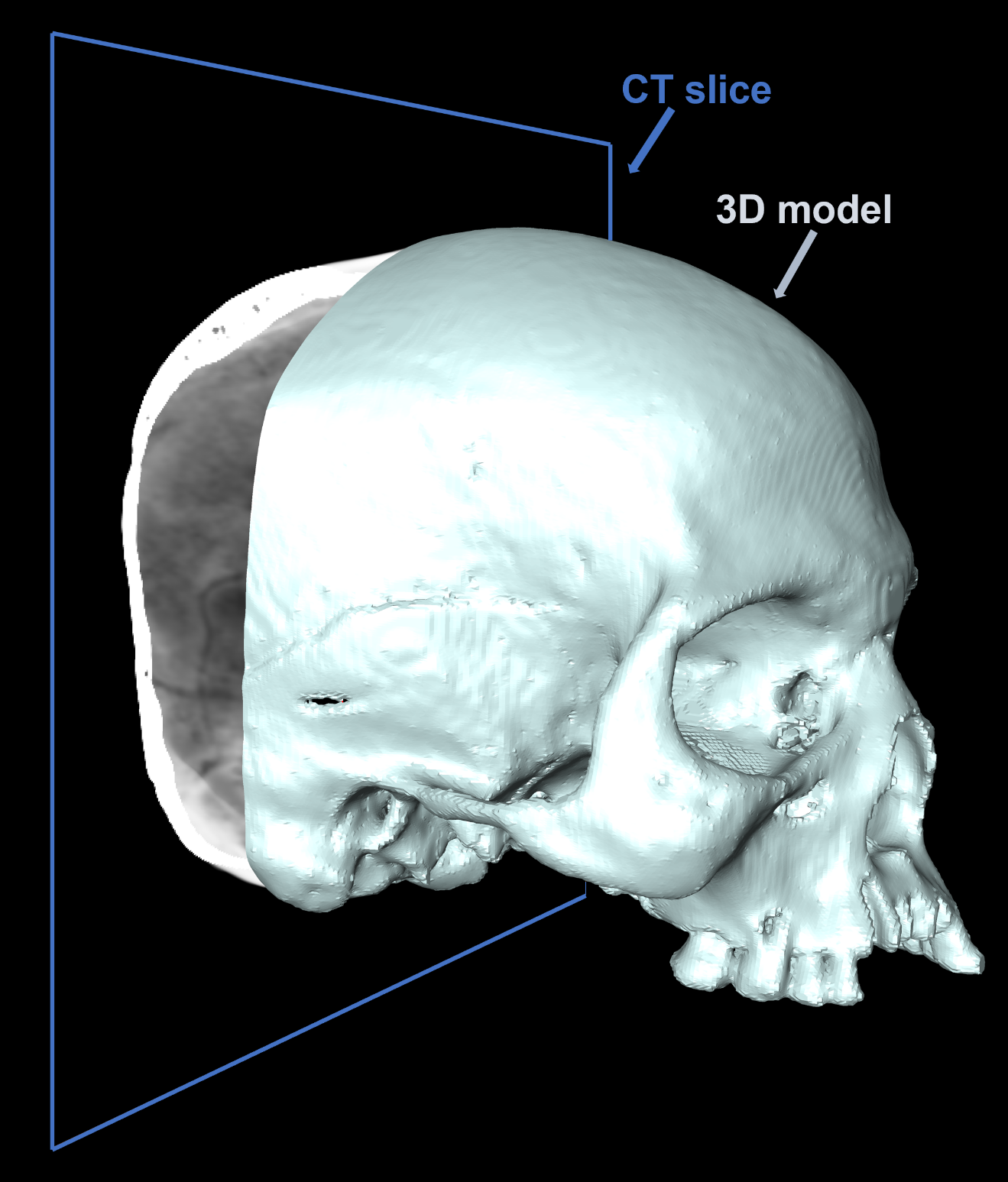


**S20 Fig. Sex determination. X-chromosomal coverage vs. Y-chromosomal coverage, normalized by autosomal coverage. Error bars represent the uncertainty in the calculation of relative coverages.**


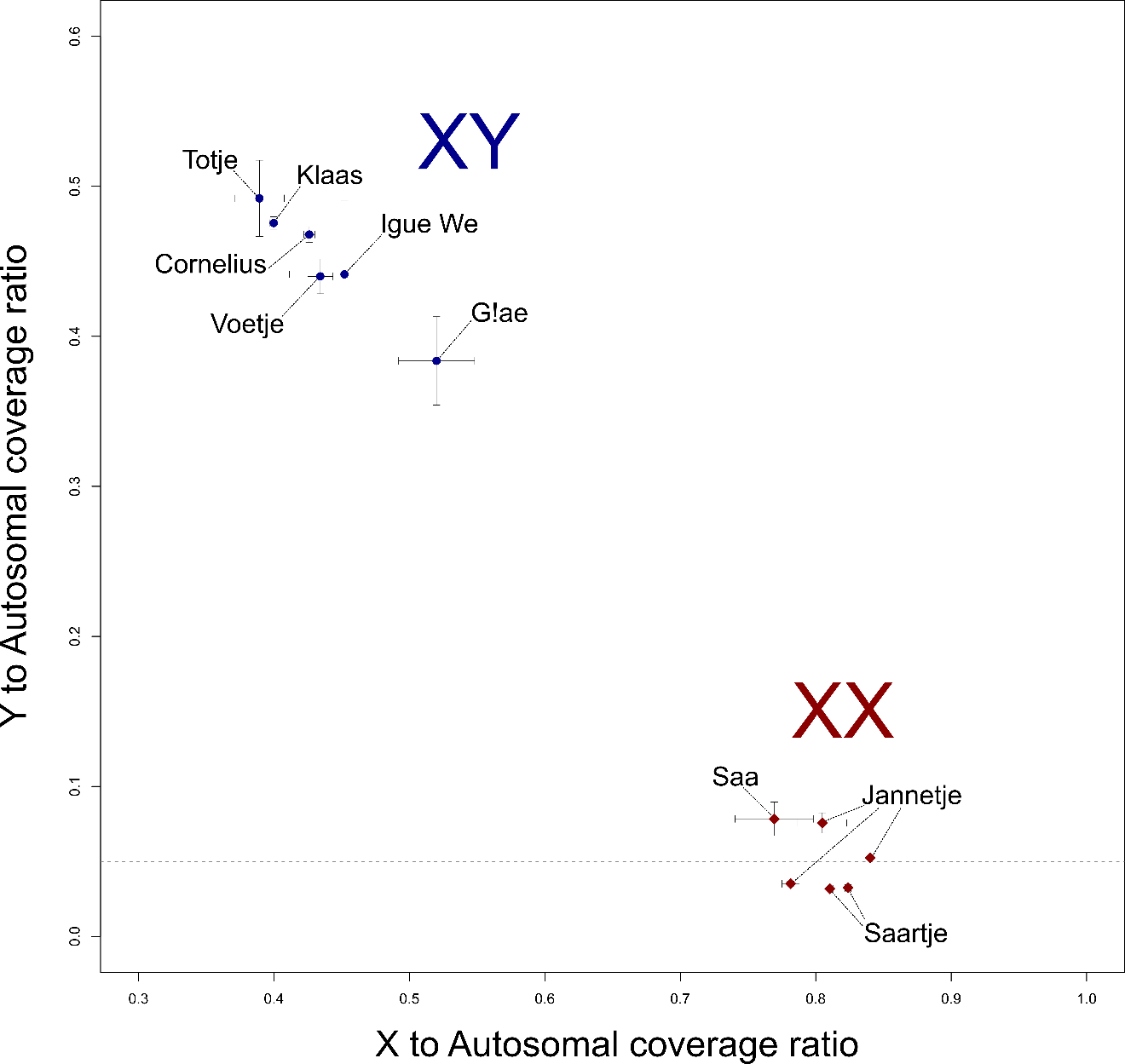


**S21 Fig. Pairwise mismatch rates between all sequenced libraries. Individual pairs of interest are highlighted accordingly.**


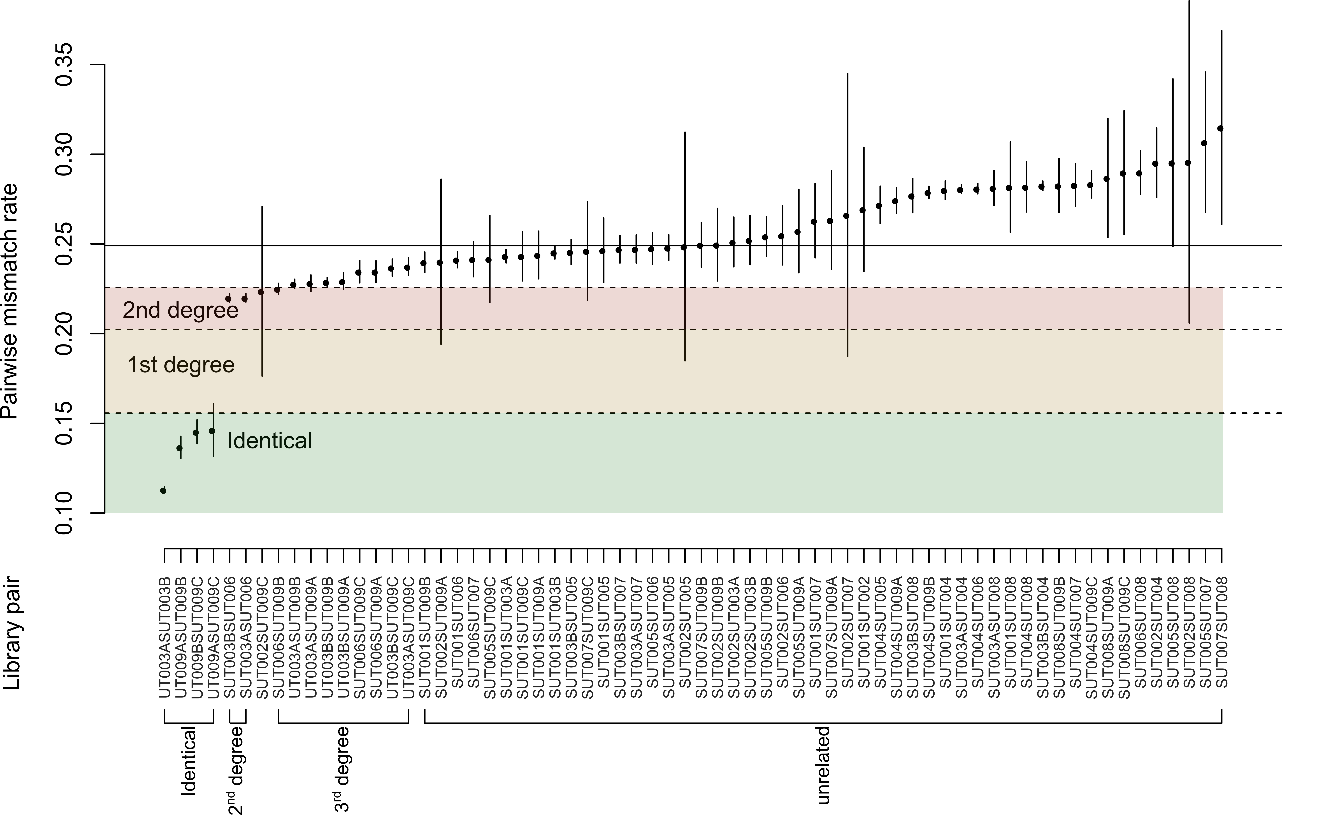


**S22 Fig. PCA and sampling locations of 40 African populations (human origins panel, 600,000 SNPs), the Sutherland and published ancient African samples are projected onto the PCs.**

**
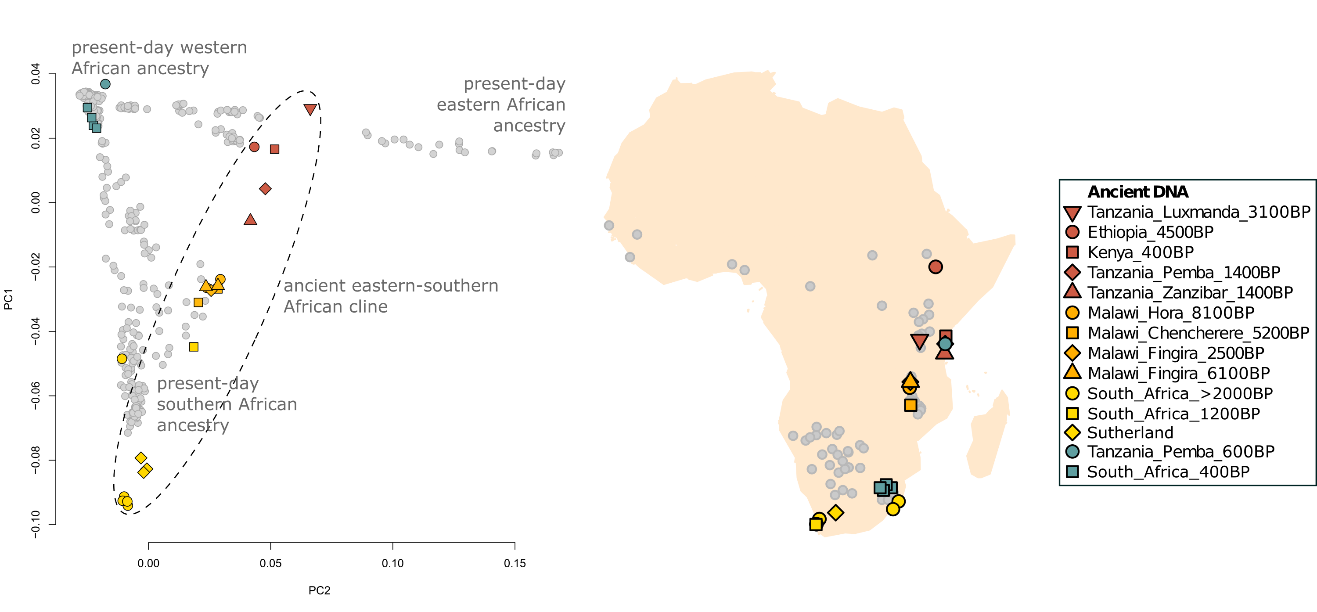
**

**S23 Fig. PCA and sampling locations of seven present-day San and Khoekhoe populations (Schlebusch panel (172), 540,000 SNPs), the Sutherland samples are projected onto the PCs.**


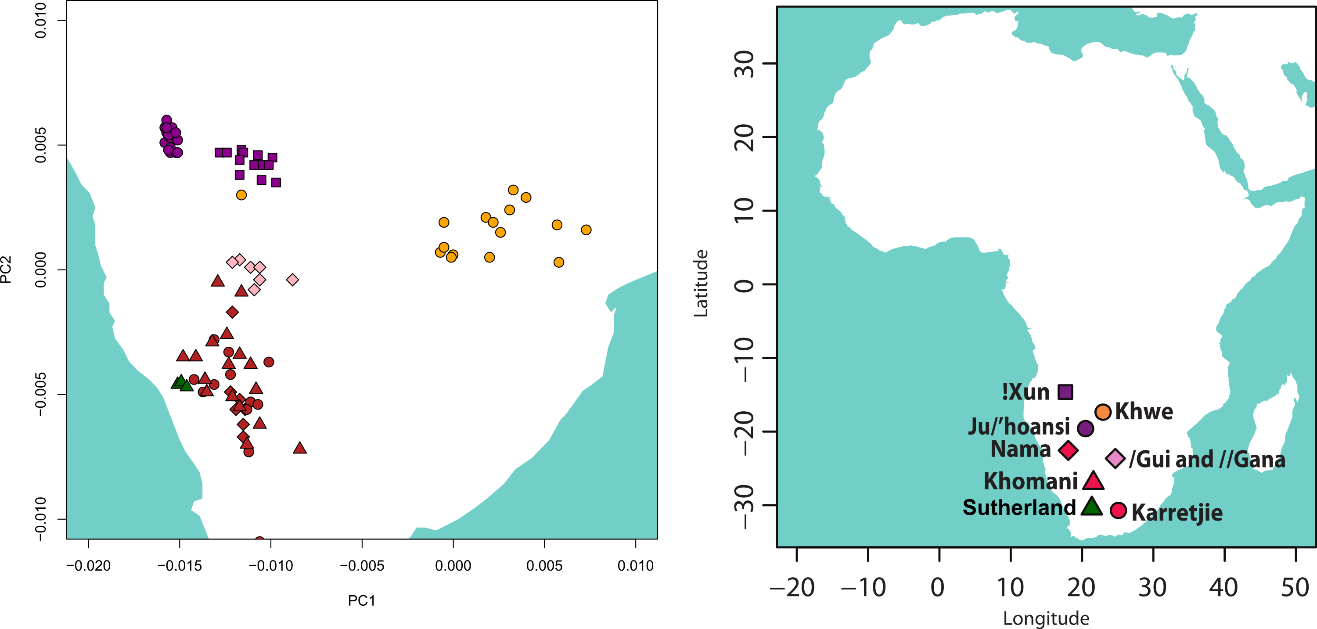


**S24 Fig. Maximum likelihood based on the allele frequencies of 1.2 million SNPs from populations of the <1240k> panel show no evidence of asymmetrical allele sharing with non-Africans indicative of recent gene flow.**


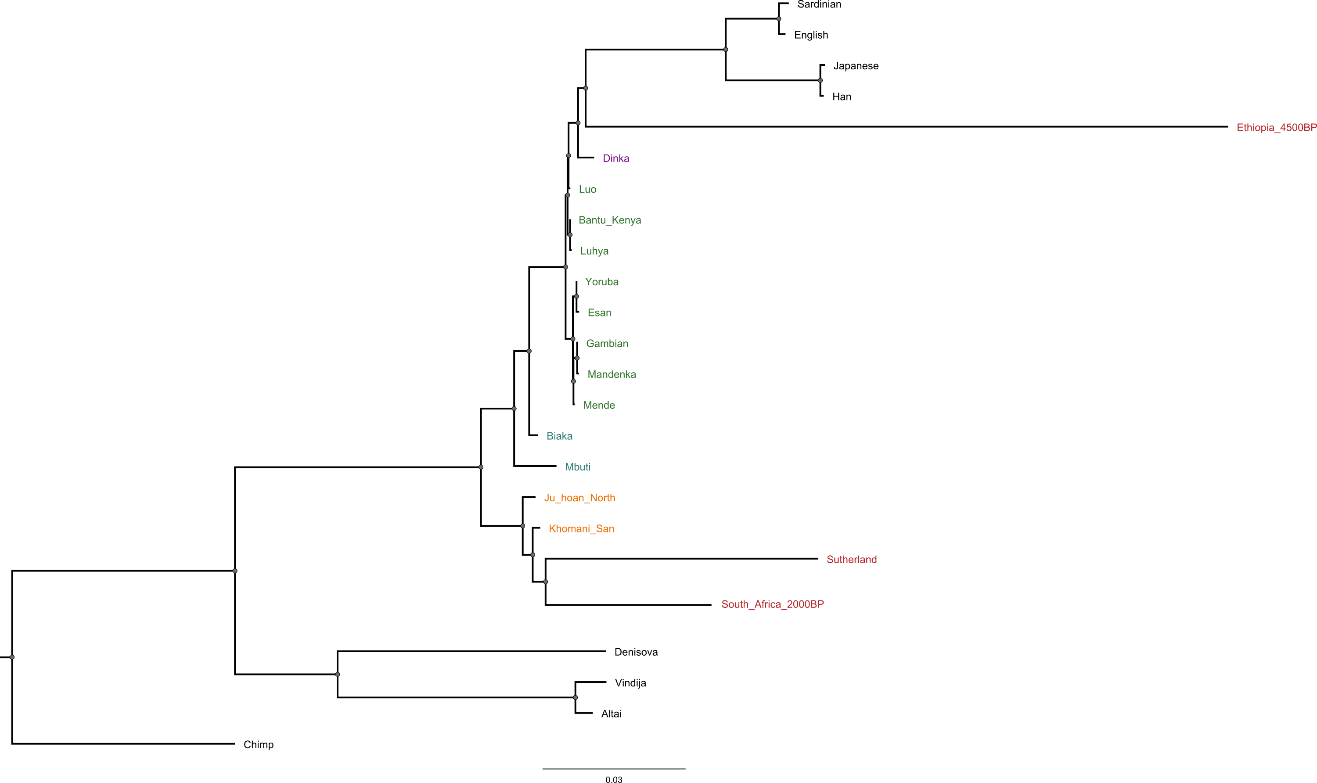


**S25 Fig. ADMIXTURE results for K = (2…10,15,20) based on 21 present-day and ancient populations from the Schlebusch <H0> panel (172).**


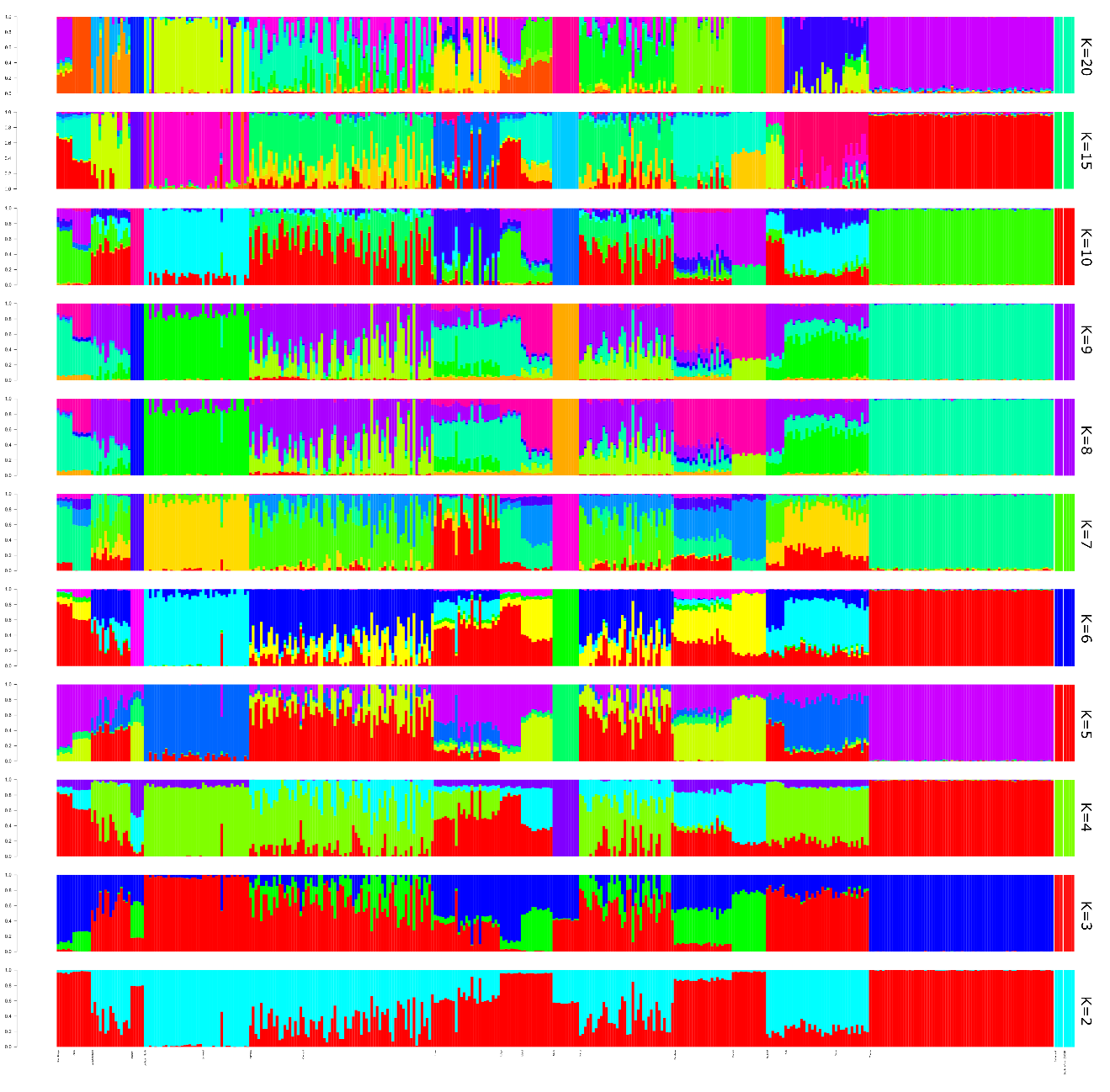


**S26 Fig. a) Outgroup f3 scores of the Sutherland samples calculated on 600,000 SNPs for 42 African populations from the human origins panel. B) Outgroup f3 scores of the Sutherland samples calculated on 540,000 SNPs for 10 Southern African populations from the Schlebusch panel (172).**


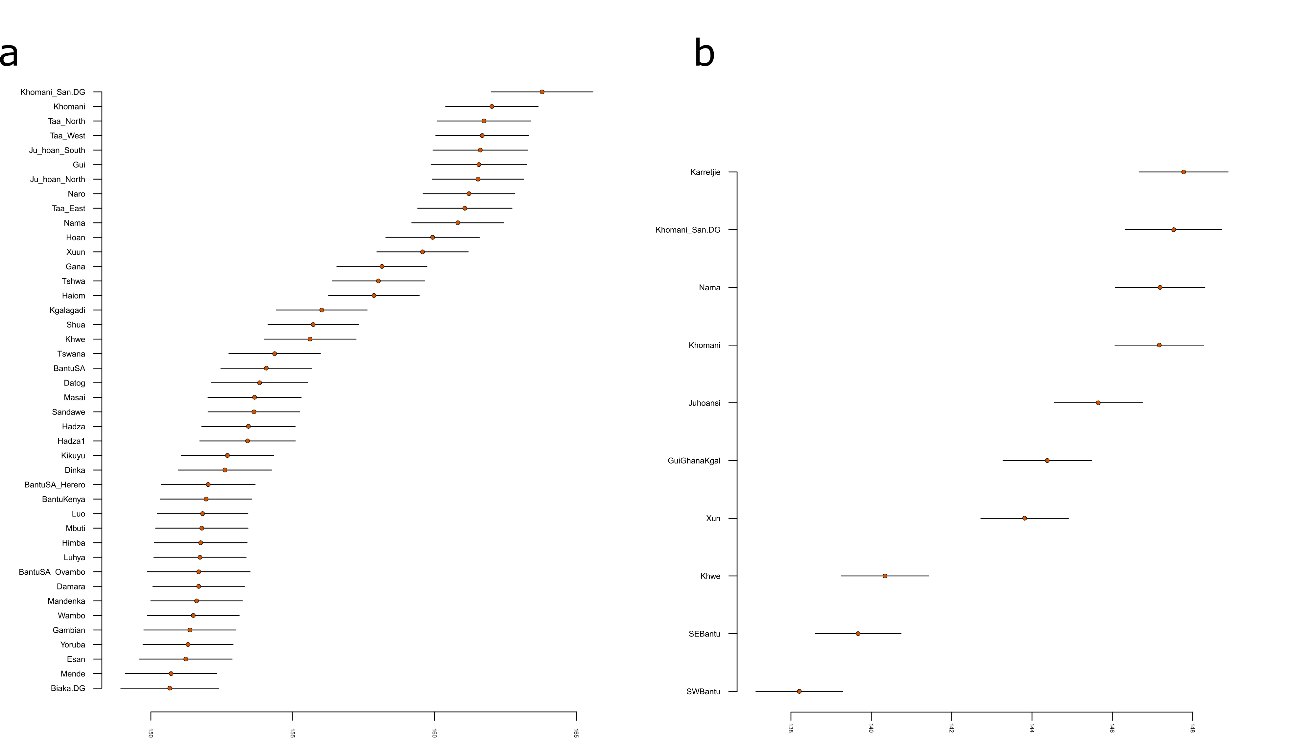


**S27 Fig. a) Heatmap of outgroup f_3_ values calculated pairwise on 1,200,000 SNPs between all analyzed published ancient samples and the 3 Sutherland individuals. b) Corresponding Multidimensional-scaling-plot (MDS) of the outgroup f_3_ values calculated pairwise between all ancient samples and the Sutherland individuals.**


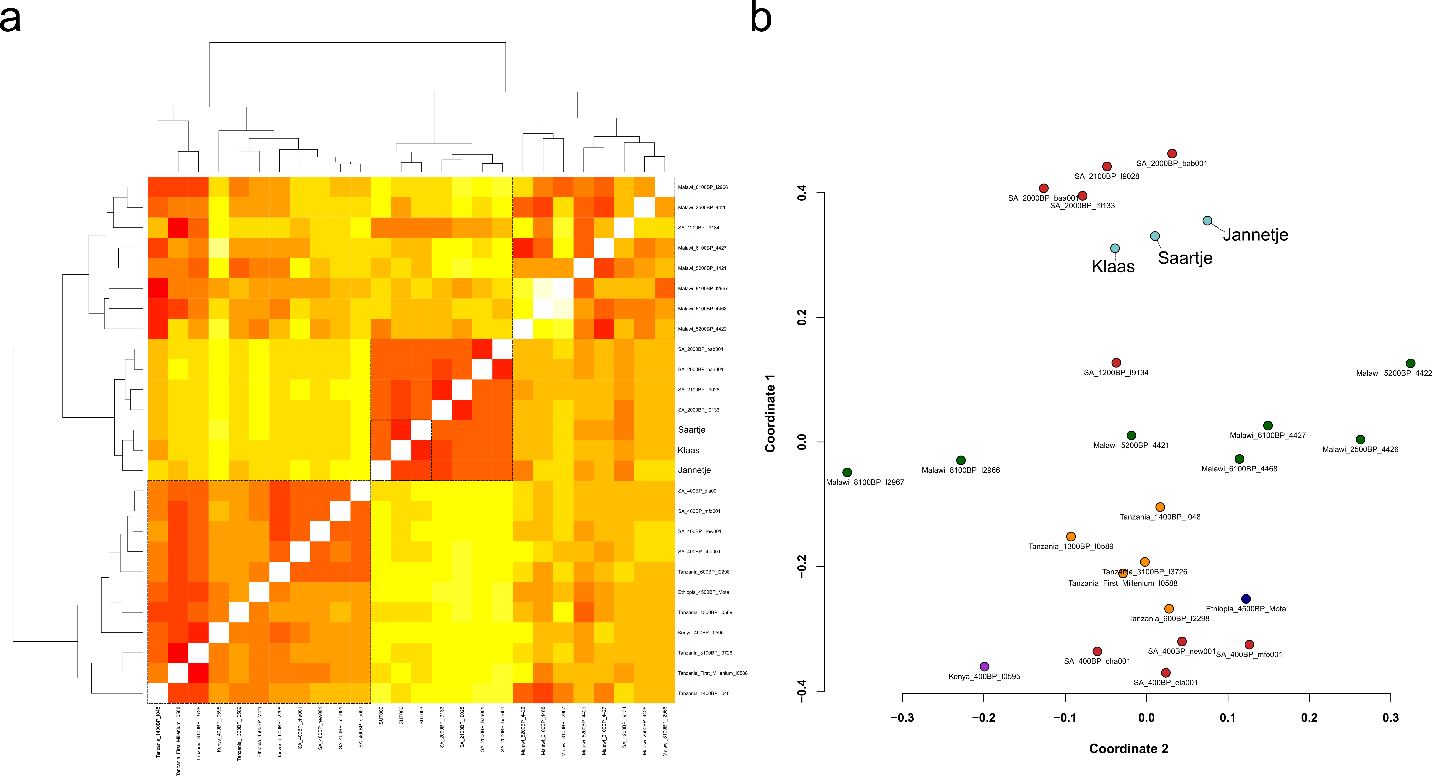


**S28 Fig. a) F_ST_ scores calculated for all 3 Sutherland individuals grouped together on 600,000 SNPs for 42 African populations from the human origins panel. b) F_ST_ scores calculated for all 3 Sutherland individuals grouped together on 540,000 SNPs for 10 southern African populations from the Schlebusch panel (172).**


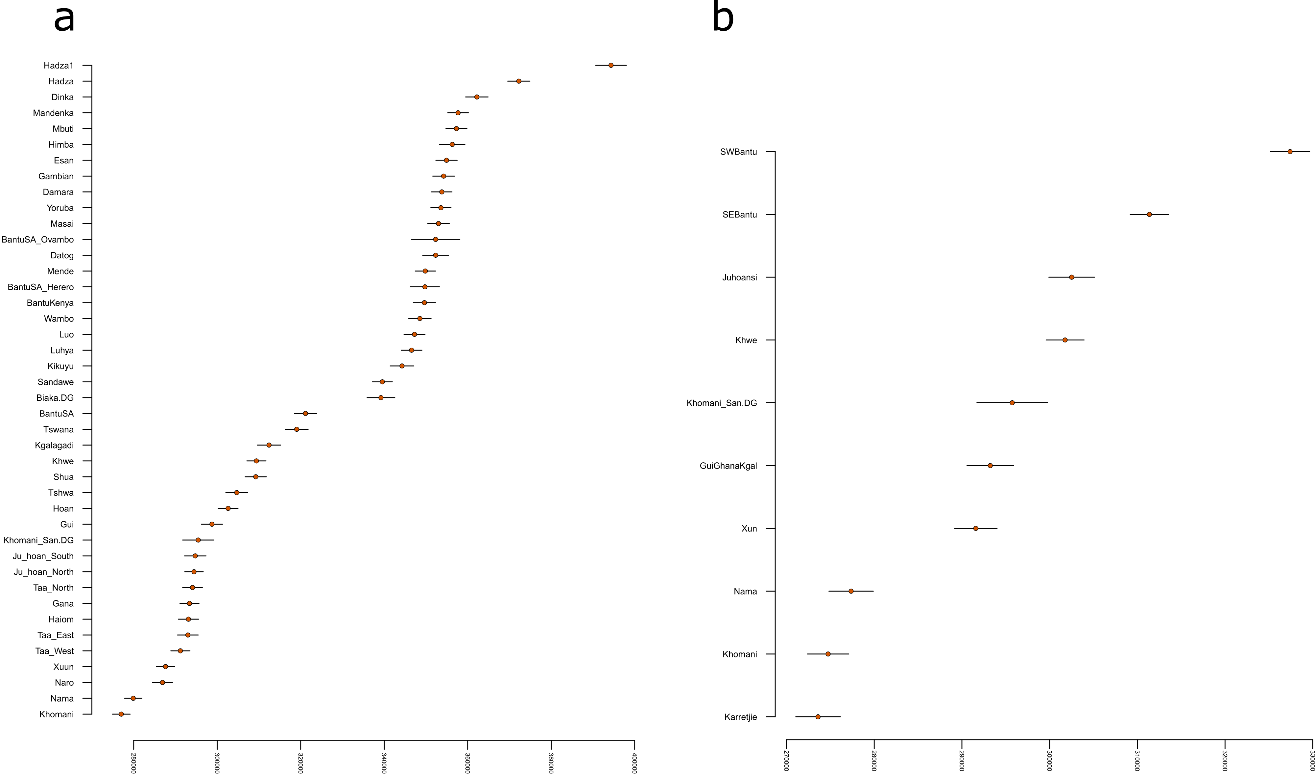


**S29 Fig. a) f_4_ statistic of the form f_4_ (Outgroup, Test; South_Africa_2000BP, Sutherland), calculated on 600,000 SNPs of the human origins panel. The Test population iterates through 45 ancient and present-day Sub-Saharan African populations. b) f_4_ statistic of the form f_4_ (Outgroup, Sutherland; South_Africa_2000BP, Test), calculated on 600,000 SNPs of the human origins panel. The test population iterates through 45 ancient and present-day sub-Saharan African populations.**


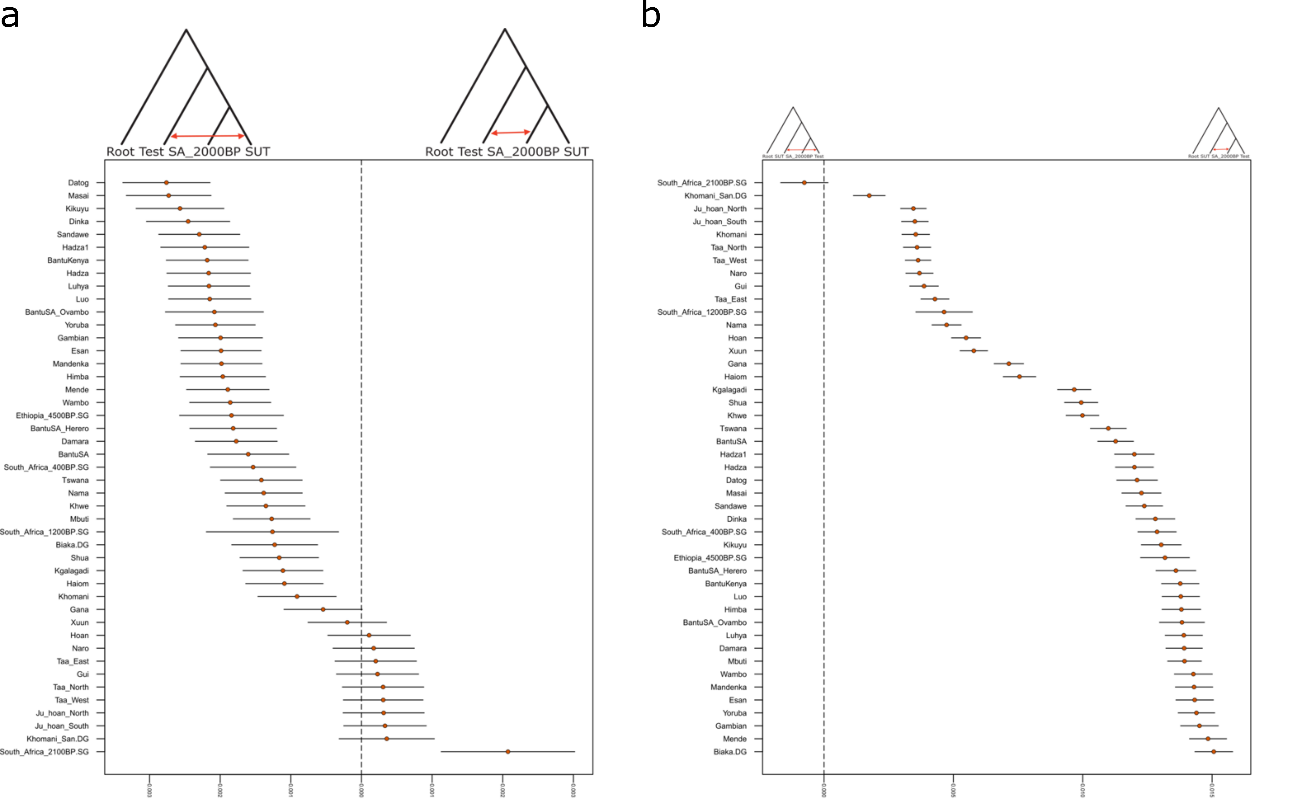


**S30 Fig. a) f_4_ statistic of the form f_4_ (Outgroup, Sutherland; Khomani_San.DG, Test), calculated on 600,000 SNPs of the Human Origins panel. The test population iterates through 44 ancient and present-day sub-Saharan African populations. b) f_4_ statistic of the form f_4_ (Outgroup, Test; Khomani_San.DG, Sutherland), calculated on 600,000 SNPs of the human origins panel. The test population iterates through 43 present-day sub-Saharan African populations.**


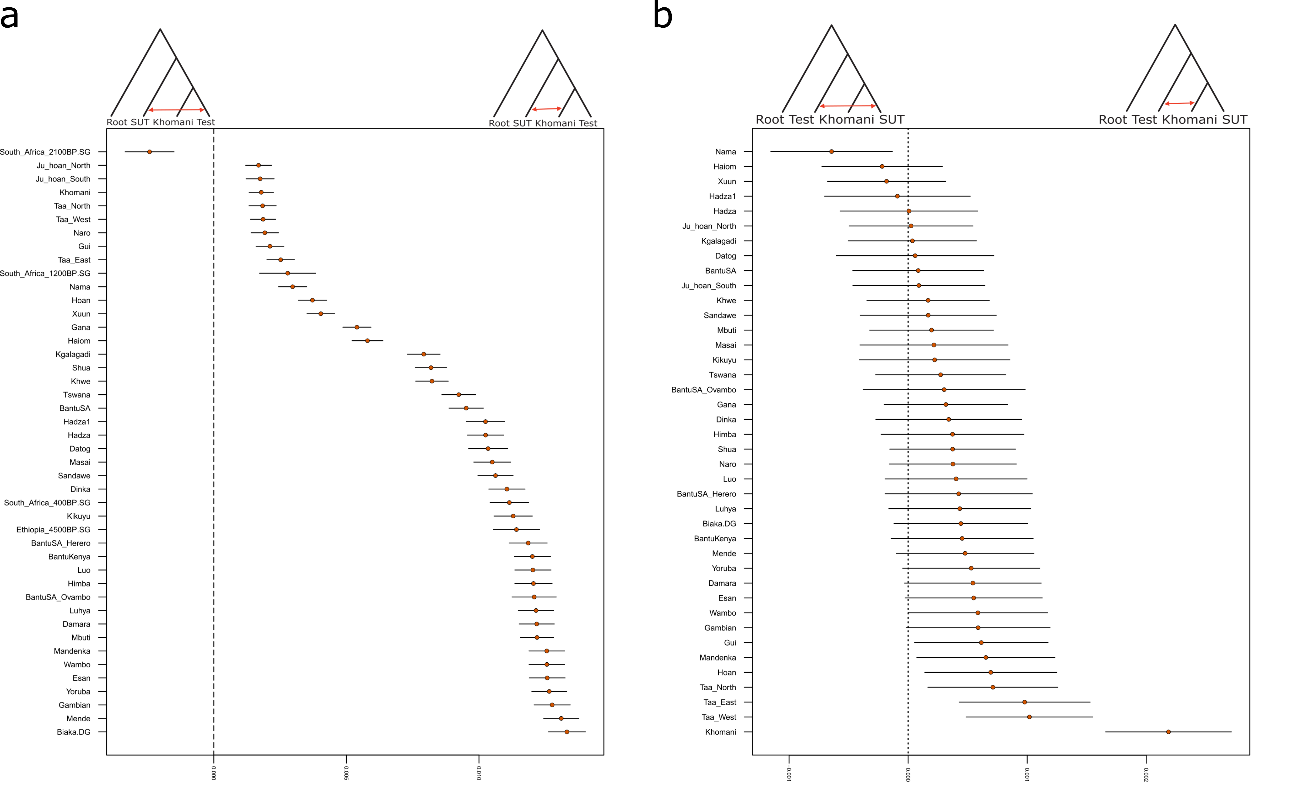


**S31 Fig. f_4_ statistic of the form f_4_ (Outgroup, Test; South_Africa_2000BP_East, South_Africa_2000BP_West), calculated on 600,000 SNPs of the human origins panel. The test population iterates through 46 ancient and present-day sub-Saharan African populations.**


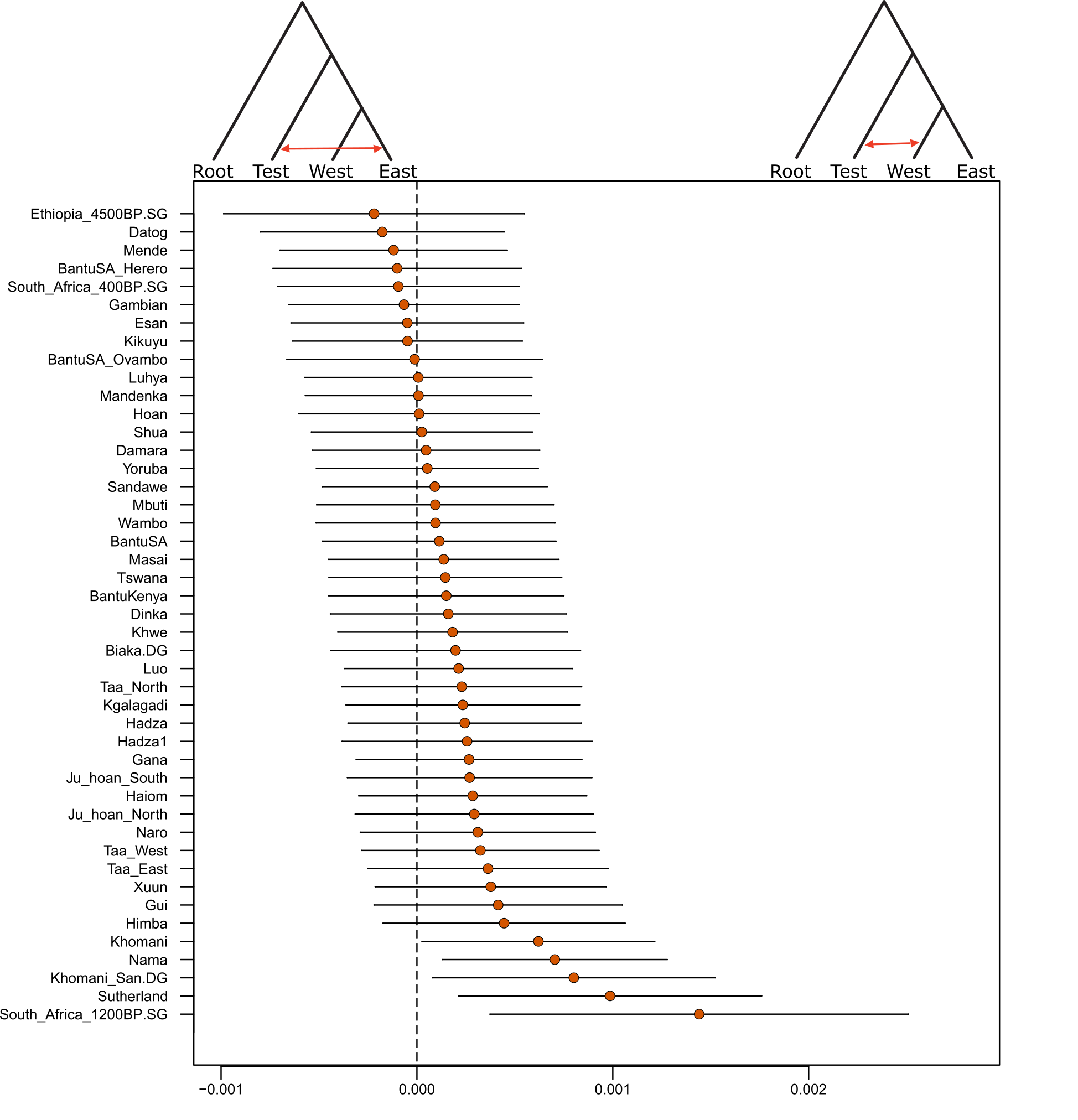


**S32 Fig. qpADM ancestry modelling on ~600,000 SNPs of selected modern and ancient southern African populations from the human origins panel.**


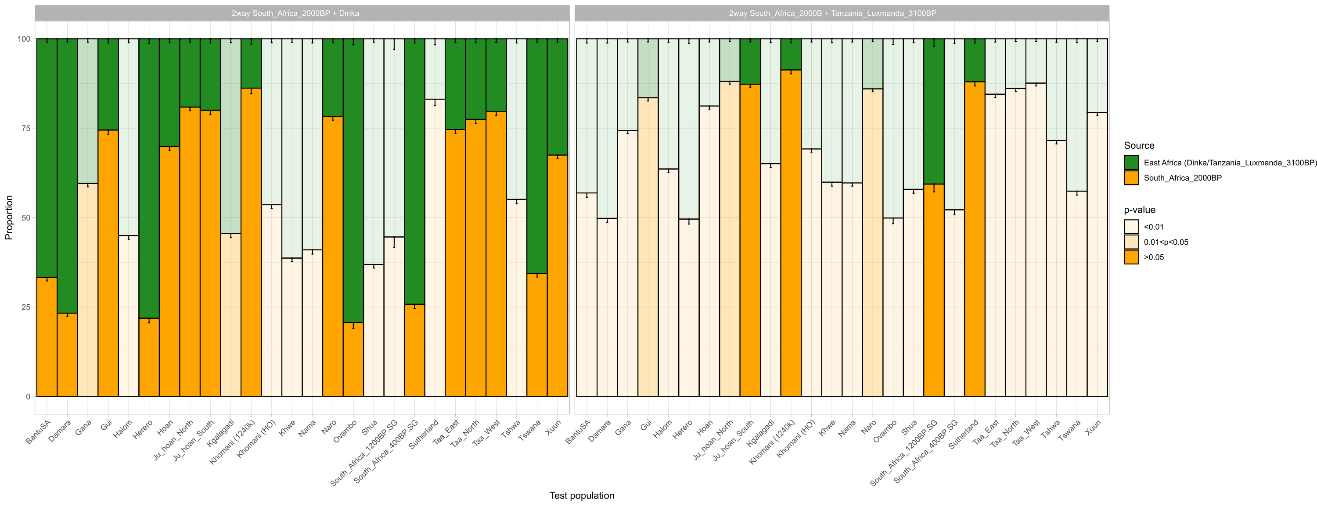


**S33 Fig. a) Admixture graph of six selected present-day and ancient sub-Saharan African populations from the 1240k panel allowing for one admixture event. b) Admixture graph of seven selected present-day and ancient sub-Saharan African populations from the human origins panel, allowing for two admixture events.**


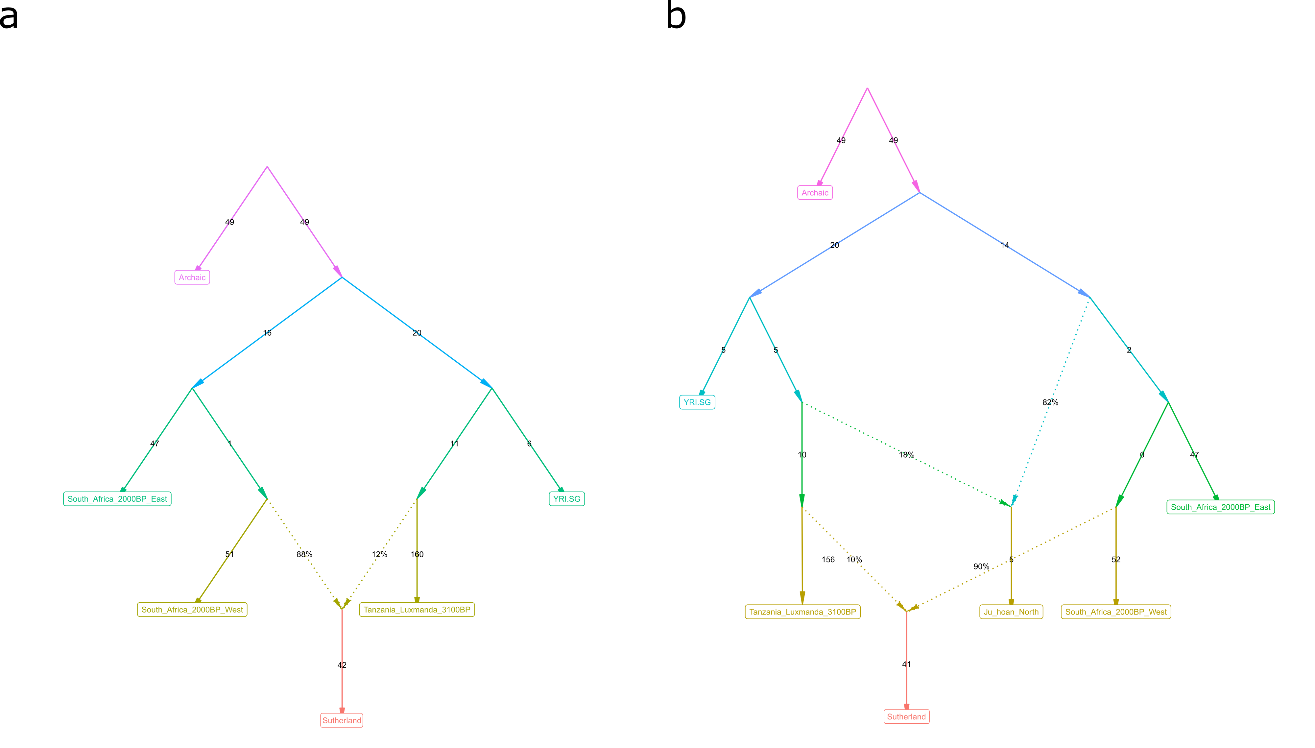


**S34 Fig. δ^15^N and δ^13^C values of serial samples of dentine (reflecting diet in early life) compared with bone from the same individuals.**


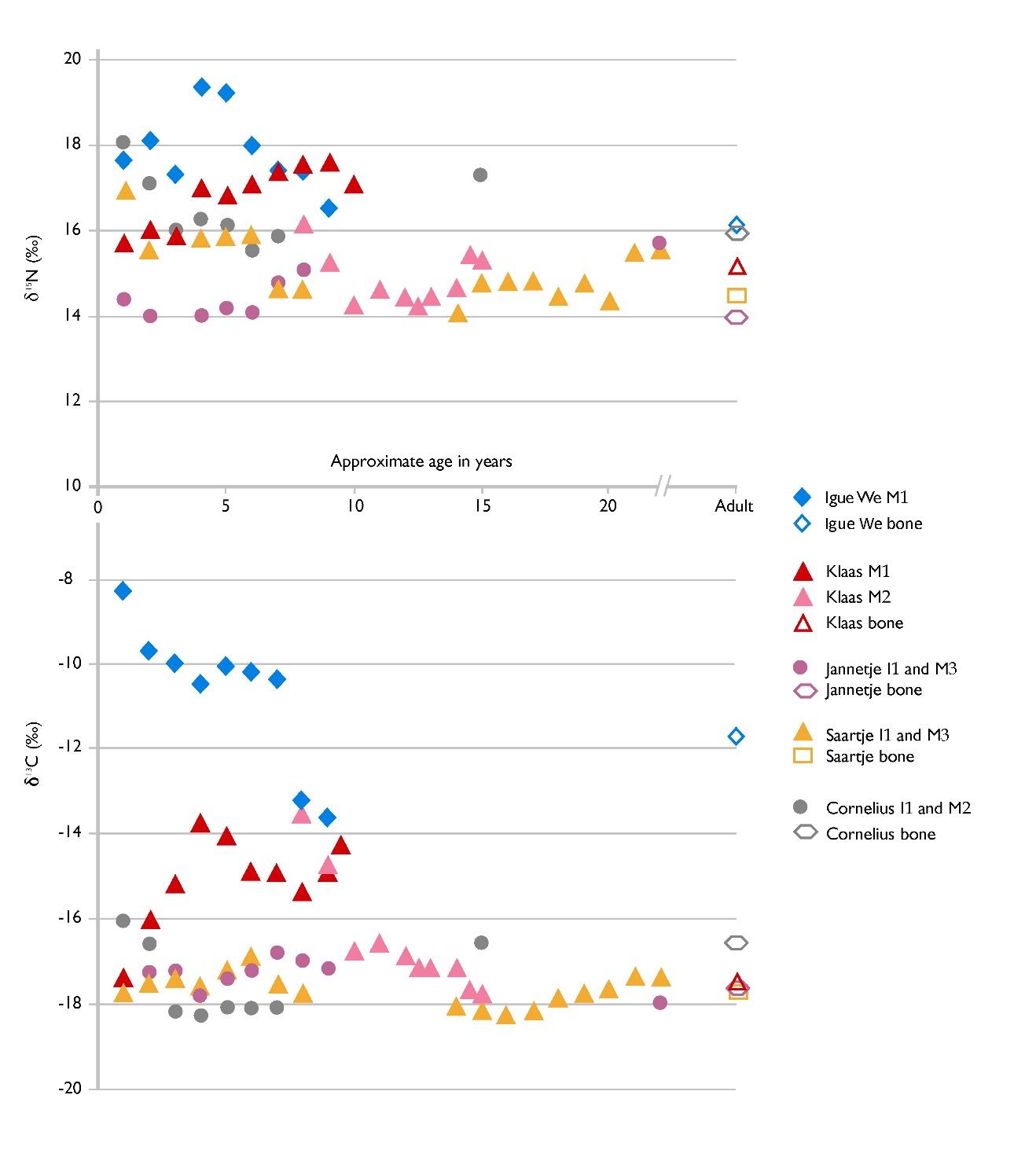


**S1 Table. Consultation guidelines for restitution and repatriation processes covered legally under the National Heritage Resources Act (Act 25 of 1999) (NHRA) and Promotion of Administrative Justice Act (Act 3 of 2000) (PAJA).**

| **SOUTH AFRICAN HERITAGE RESOURCE AGENCY CONSULTATION REQUIREMENTS** |
| --- |
| **PRE-CONSULTATION** |
| *Section 25(1) of the NHRA*: Consult with the relevant Heritage Resources Agency |
| **CONSULTATION** |
| *Sections 27, 29 38 (3) NHRA*: Local authority and ward councilors be informed |
| *Section 25(1) of the NHRA*: Broader consultation may be required that includes a public meeting. |
| *Section 35 (5) NHRA:* Heritage resources contribute significantly to research, education and tourism and they must be developed and presented for these purposes in a way that ensures dignity and respect for cultural values; Advert in local newspaper minimum 30 days for comment; Written correspondence with the landowner. |
| **POST CONSULTATION** |
| *Section 4(3) of PAJA:* Any comments received are considered prior to taking the administrative action. |
| *Section 35 (5) NHRA:* Formal reporting and distribution of the information learned during consultation and research process. |
| *Section 27, 29, 30, 31, 34, 38 (4) NHRA:* Proof of the consultation process; Copies of the newspaper notices; Copies of any and all comments received; Contact details of interested parties. |

**S1 Dataset (separate file).** Genetic analyses sample statistics overview used reference data, mitochondrial DNA and Y chromosome haplogroup determination, and qpAdm modelling.

**S2 Dataset (separate file).**  Stable isotope values and collagen quality indicators.

**SUPPLEMENTARY PAPER REFERENCES**

1. Republic of South Africa Department of Sports, Arts and Culture. Draft: National Policy on the Restitution and Repatriation of Human Remains, Associated Objects and Documentation. Pretoria: Republic of South Africa; 2021.
2. INVOLVE, Briefing notes for researchers: involving the public in NHS, public health and social care research (2004). Available at <https://www.invo.org.uk/resource-centre/resource-for-researchers/>. Accessed May 20, 2021.
3. Haggett C. Public engagement in planning for renewable energy. In: Davoudi S, Crawford J, Mehmood A, editors. Planning for Climate Change: Strategies for Mitigation and Adaptation for Spatial Planners. London:Routledge; 2009. pp. 297–307.
4. Tuler S, Webler T. Voices from the forest: What participants expect of a public participation process. Soc Nat Resour. 1999;12(5): 437-453. <http://dx.doi.org/10.1080/089419299279524>
5. Sealy J. Managing collections of human remains in South African museums and universities: ethical policy-making and scientific value. South African Journal of Science. 2003;99: 238-239. <http://hdl.handle.net/11427/26913>
6. Aitken M, Haggett C, Rudolph D. Practices and rationales of community engagement with wind farms: awareness raising, consultation, empowerment. Planning Theory & Practice. 2016;17: 557-576. <http://doi.org/10.1080/14649357.2016.1218919>.
7. Turnbull P. Vermillion Accord on Human Remains (1989) (indigenous archaeology). In: Smith C, editor. Encyclopaedia of Global Archaeology. New York: Springer; 2014. pp. 7615-7617. <https://doi.org/10.1007/978-1-4419-0465-2_2007>
8. Gibbon VE. African ancient DNA research requires robust ethics and permission protocols. Nat Rev Genet. 2020;21: 645-647.  <https://doi.org/10.1038/s41576-020-00285-w>.
9. Black W, Gibbon VE, Omar R. Navigating shifting sands: Human skeletal restitution lessons and guidelines from South Africa. In: Smith C, Lippert D, Pollard K, Kunango AK, May S, Varela SLL, editors. Oxford Handbook of Global Indigenous Archaeologies. (Oxford, In press), pp. xx-xx.
10. Gibbon VE, Morris AG. UCT Human Skeletal Repository: Its stewardship, history, composition and educational use. Homo. 2021;25(2): 139-147. https://doi.org/[10.1127/homo/2021/1402](https://doi.org/10.1127/homo/2021/1402)
11. Fick SE, Hijmans RJ. WorldClim 2: new 1‐km spatial resolution climate surfaces for global land areas. Int J Climatol. 2017;37(12): 4302-4315. <https://doi.org/10.1002/joc.5086>
12. Penn N. Pastoralists and pastoralism in the Northern Cape frontier zone during the eighteenth century. South African Archaeological Society, Goodwin Series. 1986;5: 62-68. <https://doi.org/10.2307/3858148>
13. Penn N. The Forgotten Frontier: Colonist & Khoisan on the Cape’s Northern Frontier in the 18th Century. Cape Town: Double Storey Books; 2005.
14. Adhikari M. The Anatomy of a South African Genocide: The Extermination of the Cape San Peoples. Cape Town: UCT Press; 2010.
15. Adhikari M. A total extinction confidently hoped for: the destruction of Cape San society under Dutch colonial rule, 1700-1795. J Genocide Res. 2010;12 (1-2): 19-44. <https://doi.org/10.1080/14623528.2010.508274>
16. Smith AB. Khoikhoi susceptibility to virgin soil epidemics in the 18th century. S Afr Med J. 1989;75: 25–26. <https://journals.co.za/doi/pdf/10.10520/AJA20785135_8535>
17. Diamond J. Guns, Germs and Steel: The Fates of Human Societies. New York: W. W. Norton; 1997.
18. Bank A. Bushmen in a Victorian World: The Remarkable Story of the Bleek-Lloyd Collection of Bushman Folklore. Cape Town: Double Storey Books; 2006.
19. De Prada-Samper JM. The forgotten killing fields: ‘San’ genocide and Louis Anthing's mission to Bushmanland, 1862-1863. Historia. 2012;57: 172-187. Available at <http://www.scielo.org.za/scielo.php?script=sci_arttext&pid=S0018-229X2012000100010>
20. Bleek WH, Lloyd LC. Specimens of Bushman Folklore. London: George Allen & Company, Ltd; 1911.
21. Hoff A. The water snake of the Khoekhoen and/Xam. S Afr Archaeol Bull. 1997;52: 21-37.
22. Waldman L. Houses and the ritual construction of gendered homes in South Africa. J Roy Anthrop Inst. 2003;9(4): 657-679.
23. De Prada-Samper JM. The Man who cursed the wind: Karoo storytellers and their narratives. Cape Town: African Sun Press; 2016.
24. Wolter’s Woordenboeken. Nederlands-Engels, Vol. II. Groningen: Wolters-Noordhoff; 1977. p. 779.
25. See V.C. Malherbe, “David Stuurman”, in Dictionary of South African Biography, Vol. IV (Human Science Research Council, Butterworth, 1981), pp. 634-5; “Klaas Stuurman”, in Dictionary of South African Biography, Vol. V (Human Science Research Council, Pretoria, 1987), pp. 753; S. Newton-King and V.C. Malherbe, The Khoikhoi Rebellion in the Eastern Cape (1799-1803) (UCT Centre for African Studies, Cape Town, 1981); V.C. Malherbe, ‘David Stuurman: “Last Chief of the Hottentots” ‘, African Studies, XXXIX, (1980); ‘Hermanus and his Sons: Khoi Bandits and Conspirators in the Post-Rebellion Period , 1803-1818’, African Studies, XLI, (1982).
26. Burchell WJ. Travels in The Interior of Southern Africa, Vol. Two. London: The Batchworth Press; 1953. pp. 117 and 166. <https://doi.org/10.5962/bhl.title.100911>.
27. Legassick MC. The Politics of a South African Frontier: The Griqua, the Sotho-Tswana, and the Missionaries, 1780-1840. Basel: Basler Afrika Bibliographien; 2010. pp. 55.
28. Schoeman K. The Griqua Captaincy of Philippolis, 1826-1861. Pretoria: Protea Book House; 2002. pp. 112.
29. J. Philip. Evidence delivered by Rev. John Philip, 4 July 1836 in Report of the Select Committee on Aborigines, Vol. I, Part I. Imperial Blue Book, 1836 nr VII, 538, pp. 617-618 (1863).
30. M. Heale, “A description and interpretation of Kruisrivier Cemetery”, Archaeology Honours thesis, (University of Cape Town, Cape Town, 2019).
31. I. Schapera, *The Khoisan Peoples of South Africa* (Routledge, London, 1930).
32. P. T. Robertshaw, “Coastal settlement, freshwater fishing and pastoralism in the later prehistory of the Western Cape, South Africa*”,* PhD thesis, (University of Cambridge, Cambridge, 1979).
33. F. B. Silberbauer, “Stable carbon isotopes and prehistoric diets in the Eastern Cape Province, South Africa”, Masters thesis, (University of Cape Town, Cape Town, 1979).
34. Hall S, Binneman J. Later Stone Age burial variability in the Cape: A social interpretation. South African Archaeological Bulletin. 1987;42: 140-152. <https://doi.org/10.2307/3888740>.
35. Potgieter EF. The Disappearing Bushmen of Lake Chrissie: A Preliminary Survey. Pretoria: J. L. Van Schaik, Ltd; 1955.
36. Heinz HJ. A !Xõ Bushmen Burial. In: Vossen R, Keuthmann K, editors. Contemporary Studies on Khoisan. Hamburg: Helmut Buske Verlag; 1986. pp. 23-36.
37. Morris AG. DeTuin, a 19th century mission station in the Northern Cape. In: Swanepoel N, Esterhuysen A, Bonner P, editors. Five Hundred Years Rediscovered. Johannesburg: Witwatersrand University Press; 2008. pp. 103-118.
38. Lewis-Williams JD, Pearce DG. San Spirituality: Roots, Expression and Social Consequences. Walnut Creek: AltaMira Press; 2004.
39. J. Burness, “A first assessment of an 18^th^-century loan farm deposit from Kruisrivier in the Roggeveld”, Unpublished Honours thesis, University of Cape Town (2019).
40. Bronk RamseyC. Bayesian analysis of radiocarbon dates. Radiocarbon. 2009;51(1): 337-360.
41. Hogg AG, Heaton TJ, Hua Q, Palmer JG, Turney CS, Southon J,et al. SHCal20 Southern Hemisphere calibration, 0–55,000 years cal BP. Radiocarbon. 2020;62(4): 759-778.
42. Bennett T, Gibbon VE, Taylor LD, Heathfield LJ. Sex estimation for forensic human identification: a case report of discordance between anthropological assessment and DNA analysis. Am J Phys Anthropol. 2021;174: pp. 9 . <https://doi.org/10.1002/ajpa.24262>
43. Beom J, Woo E, Lee I, Kim M, Kim Y, Oh C, et al. Harris lines observed in human skeletons of Joseon Dynasty, Korea. Anatomy & Cell Biology. 2014;47: 66-72. [10.5115/acb.2014.47.1.66](https://doi.org/10.5115%2Facb.2014.47.1.66)
44. Stinson S. Early childhood health in foragers. In: Ungar PS, Teaford MF, editors. Human Diet: Its Origin and Evolution. New York: Wiley-Liss, 2002. pp. 587-635.
45. Clayton F, Sealy J, Pfeiffer S. Weaning age among foragers at Matjes River rock shelter, South Africa, from stable nitrogen and carbon isotope analyses. Am J Phys Anthropol. 2021;129(2): 311-317. <https://doi.org/10.1002/ajpa.20248>
46. Gibbon VE, Davies B. Holocene Khoesan health: a biocultural analysis of cranial pathology and trauma. Int J Osteoarchaeol*.* 2020;30: 287-296. <https://doi.org/10.1002/oa.2854>
47. Burchell WJ. Travels in the Interior of Southern Africa. Facsimile Reprint of 1824 original. Cape Town: Struik; 1967.
48. Humphreys AJB, O’C Maggs TM. Further graves and cultural material from the banks of the Riet River. South African Archaeological Bulletin. 1970;25: 116-126. <https://doi.org/10.2307/3888135>
49. Resnick DL, Kransdorf MJ. Osteoporosis. In: Ross A, Gaillard JM, editors. Bone and Joint Imaging. Philadelphia: Elsevier Inc.; 2005. pp. 541-562. <https://doi.org/10.1016%2Fb0-7216-0270-3%2F50044-x>
50. Tonina E, Larentis O, Tesi C, Fusco R, Campagnolo M, Licata M. A severe case of biparietal thinning in a medieval skull from a Northern Italy necropolis. J Craniofac Surg. 2022;33(1): 70–75. <https://doi.org/10.1097/scs.0000000000007929>
51. Mann RW, Kobayashi M, Schiller AL. Biparietal thinning: accidental death by a fall from standing height. J Forensic Sci. 2017;62(5): 1406–1409. <https://doi.org/10.1111/1556-4029.13425>
52. Nascimento JJ, Neto EJ, Mello-Junior CF, Valença MM, Araújo-Neto SA, Diniz, PR. Diagnostic accuracy of classical radiological measurements for basilar invagination of type B at MRI. Eur Spine J. 2019;28(2): 345-352. <https://doi.org/10.1007/s00586-018-5841-4>
53. Smoker WR. Craniovertebral junction: normal anatomy, craniometry, and congenital anomalies. Radiographics. 1994;14(2): 255–277. <https://doi.org/10.1148/radiographics.14.2.8190952>
54. Smith JS, Shaffrey CI, Abel MF, Menezes AH. Basilar invagination. Neurosurgery. 2010;66(suppl_3): A39-A47. <https://doi.org/10.1227/01.neu.0000365770.10690.6f>
55. Yochum RT, Rowe JL. Essentials of Skeletal Radiology. Baltimore, Philadelphia: Lippincott Williams & Wilkins; 2005.
56. Baysal B, Eser MB, Sorkun M. Radiological approach to basilar invagination type B: reliability and accuracy. J Neuroradiol. 2022;49(1): 33–40. <https://doi.org/10.1016/j.neurad.2020.08.005>
57. Nascimento JJ, Silva LM, Ribeiro EC, Neto EJ, Araújo-Neto SA, Diniz PR. Foramen magnum angle: a new parameter for basilar invagination of type B. World Neurosurgery. 2021;152: 121–123. <https://doi.org/10.1016/j.wneu.2021.06.028>
58. Xu S, Gong R. Clivus height value: a new diagnostic method for basilar invagination at CT. Clin Radiol. 2016;71(11): 1200.e1-1200.e5. <https://doi.org/10.1016/j.crad.2016.04.007>
59. B. M. Auerbach, 2014, *The William W. Howells Craniometric Data Set*, viewed 12 October 2021, http://volweb.utk.edu/~auerbach/HOWL.htm.
60. Pinter NK, McVige J, Mechtler L. Basilar invagination, basilar impression, and platybasia: clinical and imaging aspects. Curr Pain Headache Rep*.* 2016;20(8): 1–8. <https://doi.org/10.1007/s11916-016-0580-x>
61. Goel A. Short neck, short head, short spine, and short body height–Hallmarks of basilar invagination. Journal of Craniovertebral Junction & Spine. 2017;8(3): 165-167. <https://doi.org/10.4103/jcvjs.jcvjs_101_17>
62. Goel A, Sathe P,Shah A. Atlantoaxial fixation for basilar invagination without obvious atlantoaxial instability (Group B basilar invagination): Outcome analysis of 63 surgically treated cases. World Neurosurg. 2017;99: 164–170. <https://doi.org/10.1016/j.wneu.2016.11.093>
63. Grimoud AM, Gibbon VE, Ribot I. Predictive factors for alveolar fenestration and dehiscence. HOMO. 2017;68: 167-175. <https://doi.org/10.1016/j.jchb.2017.03.00>.
64. Wanneburg A, Johnson P, Bannister A. The Bushmen. Cape Towb: New Holland Books; 1999.
65. Viestad VM. “Nearly Naked”? Indigenous expressions of identity in a colonial world – tradition and change in the San dress of southern Africa. In: Thiaw I, Bocoum H, editors. Conference Proceedings of the 13th Congress of the PanAfrican Archaeological Association for Prehistory and Related Studies PAA and the 20th Meeting of the Society of Africanist Archaeologists Safa - Preserving African Cultural Heritage. Senegal: Université Cheikh Anta Diop; 2015. pp. 405-414.
66. Garza RM, Khosla RK. Nonsyndromic craniosynostosis. Semin. Plast Surg. 2012;26: 53-63. <https://doi.org/10.1055/s-0032-1320063>.
67. Aufderheide AC, Rodríguez-Martín C. The Cambridge Encyclopedia of Human Paleopathology. Cambridge: Cambridge University Press; 1998.
68. Khongsdier R. Bio-cultural approach: The essence of anthropological study in the 21st century. In: Bhasin V, Bhasin MK, editors. Anthropology Today: Trends, Scope and Applications. Gurugram: Kamla-Raj Enterprises; 2007. pp. 39-50.
69. Agarwal SC, Glencross BA. Building a social bioarchaeology. In: Agarwal SC, Glencross BA, editors. Social Bioarchaeology. Hoboken: Wiley-Blackwell; 2011.pp.1-12.
70. Hosek L, Robb J. Osteobiography: A platform for bioarchaeological research. Bioarchaeol Int. 2019;3: 1-22. <https://doi.org/10.5744/bi.2019.1005>.
71. Phenice TW. A newly developed visual method of sexing the os pubis. Am J Phys Anthropol. 1969;30: 297-302. <https://doi.org/10.1002/ajpa.1330300214>.
72. Buikstra JE, Ubelaker DH. Standards for Data Collection from Human Skeletal Remains. Fayetteville: Arkansas Archaeological Survey Research Series, no. 44; 1994.
73. Schour I, Massler M. The development of the human dentition. J Am Dent Assoc. 1941;28: 1153-1160. <https://doi.org/10.1002/oa.2691>.
74. Krogman WM, İşcan MY. The Human Skeleton in Forensic Medicine. Springfield: Charles C Thomas; 1986. <https://doi.org/10.1002/ajpa.1330740117>
75. Schaefer M, Black SM, Scheuer L. Juvenile osteology: A Laboratory and Field Manual. Amsterdam: Academic Press; 2009.
76. Esan TA, Schepartz LA. The Wits atlas: A Black Southern African dental atlas for permanent tooth formation and emergence*.*Am J Phys Anthropol. 2018;166: 208-218. <https://doi.org/10.1002/ajpa.23424>.
77. Suchey JM, Brooks ST, Katz D. Instructional Materials Accompanying Female and Male Pubic Symphyseal Models of the Suchey-Brooks System. Fort Collins: France Casting; 1988.
78. Meindl R, Lovejoy C. Ectocranial suture closure: A revised method for the determination of skeletal age at death based on the lateral‐anterior sutures. Am J Phys Anthropol. 1985;68: 57-66. <https://doi.org/10.1002/ajpa.1330680106>.
79. Lovejoy BO, Meindl RS, Pryzbeck TR, Mensforth RP. Chronological metamorphosis of the auricular surface of the ilium: A new method for the determination of adult skeletal age at death. Am J Phys Anthropol. 1985;68: 15-28. <https://doi.org/10.1002/ajpa.1330680103>.
80. İşcan MY, Loth SR, Wright RK. Casts of Age Phases from the Sternal End of the Rib for White Males and Females. Bellvue, Colorado: France Casting; 1993.

Boldsen JL, Milner GR. “*Transition analysis: A new method for estimating age from skeletons”* In: Hoppa RD, Vaupel JW, editors. Paleodeomography: Age Distributions from Skeletal Samples. Cambridge: Cambridge University Press; 2002. pp. 73-106. <https://doi.org/10.1017/CBO9780511542428.005>

1. Merritt CE. The influence of body size on adult skeletal age estimation methods. Am J Phys Anthropol. 2015;156: 35-57. <https://doi.org/10.1002/ajpa.22626>.
2. Pfeiffer S, Cameron ME, Sealy J, Beresheim AC. Diet and adult age‐at‐death among mobile foragers: A synthesis of bioarcheological methods. Am J Phys Anthropol. 2019;170: 131–147. <https://doi.org/10.1002/ajpa.23883>.
3. Feldsman MR, Fountain RL. “Race” specificity and the femur/stature ratio. Am J Phys Anthropol. 1996;100: 207-224. [https://doi.org/10.1002/(SICI)1096-8644(199606)100:2<207::AID-AJPA4>3.0.CO;2-U](https://doi.org/10.1002/(SICI)1096-8644(199606)100:2%3c207::AID-AJPA4%3e3.0.CO;2-U).
4. İşcan MY, Steyn M, Krogman WM. The Human Skeleton in Forensic Medicine. 3rd ed. Springfield: Charles C Thomas; 2013.
5. Cockburn TA. Infectious diseases in ancient populations. Curr Anthropol. 1971;12: 45- 62. <https://doi.org/10.1086/201168>.
6. Lovell NC. Trauma analysis in paleopathology. Yrbk Phys Anthropol. 1997;40: 139–170. [https://doi.org/10.1002/(SICI)1096-8644(1997)25+<139::AID-AJPA6>3.0.CO;2-%23](https://doi.org/10.1002/(SICI)1096-8644(1997)25+%3c139::AID-AJPA6%3e3.0.CO;2-%23).
7. Byers S. Calculation of age at formation of radiopaque transverse lines. Am J Phys Anthropol. 1991;85(3): 339-343. <https://doi.org/10.1002/ajpa.1330850314>.
8. Kulus M, Dąbrowski P. How to calculate the age at formation of Harris lines? A step-by-step review of current methods and a proposal for modifications to Byers’ formulas. Archaeological and Anthropological Sciences. 2019;11(4): 1169-1185. <https://doi.org/10.1007/s12520-018-00773-5>.
9. Brabant H. Contribution to the knowledge of the pathology of the teeth and jaws among the early population of Belgium and the Nord region of France. The ossuary of Marville (Meuse, France). Bull Group Int Rech Sci Stomatol. 1966;9: 224–241.
10. Gibbon VE, Grimoud AM. Dental pathology, trauma and attrition in a Zambian Iron Age Sample: A macroscopic and radiographic investigation. Int J Osteoarchaeol. 2014;24: 439-458. <https://doi.org/10.1002/oa.2228>
11. Wilkinson CM. A review of forensic art. Research and Reports in Forensic Medical Science. 2015;5: 17–24. <http://dx.doi.org/10.2147/RRFMS.S60767>.
12. Wilkinson CM. Forensic Facial Reconstruction. Cambridge: Cambridge University Press; 2004.
13. Rynn C, Balueva T, Veselovskaya E. Relationships between the skull and face. In: Wilkinson CM, Rynn C, editors. Craniofacial Identification. Cambridge: Cambridge University Press; 2012. pp. 193-202.
14. Schramm K. Casts, bones and DNA: Interrogating the relationship between science and postcolonial indigeneity in contemporary South Africa. Anthropology Southern Africa. 2016;39: 131–144. <https://doi.org/10.1080/23323256.2016.1168267>.
15. Legassick M, Rassool C. Skeletons in the Cupboard: Museums and the Incipient Trade in Human Remains, 1907-1917. Cape Town: South African Museum & McGregor Museum; 2000.
16. Rassool C, Hayes P. Science and the spectacle: /Khanako’s South Africa, 1936-1937. Woodward W, Hayes P, Minkely G, editors. Deep Histories: Gender and Colonialism in Southern Africa. Amsterdam: Rodopi; 2002. pp.117-161.
17. Adhikari M. Contending approaches to coloured identity and the history of the coloured people of South Africa. History Compass. 2005;3: 1-16. <https://doi.org/10.1111/j.1478-0542.2005.00177.x>.
18. Morris AG. Controversies about the study of human remains in post-Apartheid South Africa In: O’Donnabjain B, Lozada MC, editors. Archaeological Human Remains, Springer Briefs in Archaeology*.* New York: Springer; 2014. pp. 189-198. [https://doi.org/](https://doi.org/10.1111/j.1478-0542.2005.00177.x)[10.1007/978-3-319-06370-6_14](https://doi.org/10.1007/978-3-319-06370-6_14)
19. Jethro E. Of ruins and revival: Heritage formation and Khoisan indigenous identity in post-apartheid South Africa. In: Johnson G, Kraft SE, editors. Handbook of Indigenous Religion(s). Leiden: Brill; 2017. pp. 349–365. <https://doi.org/10.1163/9789004346710_022>.
20. Houlton T, Billings B. Blood, sweat and plaster casts: Reviewing the history, composition, and scientific value of the Raymond A. Dart Collection of African Life and Death Masks. HOMO. 2017;68: 362–377. <https://doi.org/10.1016/j.jchb.2017.08.004>
21. Zuckerman MK, Crandall J. Reconsidering sex and gender in relation to health and disease in bioarchaeology. J Anthropol Archaeol. 2019;54: 161–171. <https://doi.org/10.1016/j.jaa.2019.04.001>
22. Agarwal SC, Wesp JK. Exploring Sex and Gender in Bioarchaeology. New Mexico: University of New Mexico Press; 2017.
23. Wilkinson CM, Rynn C, Peters H, Taister M, Kau CH, Richmond S. A blind accuracy assessment of computer-modeled forensic facial reconstruction using computed tomography data from live subjects. Forensic Sci Med Pathol. 2006;2: 179-187. <https://doi.org/10.1007/s12024-006-0007-9>.
24. Mahoney F, Wilkinson CM. Computer-generated facial depiction. In: Wilkinson CM, Rynn C, editors. Craniofacial Identification. Cambridge: Cambridge University Press; 2012. pp. 222-237.
25. Lee WJ, Wilkinson CM, Hwang HS. An accuracy assessment of forensic computerized facial reconstruction employing cone‐beam computed tomography from live subjects. J Forensic Sci. 2012;57: 318-327. <https://doi.org/10.1111/j.1556-4029.2011.01971.x>.
26. Short LJ, Khambay B, Ayoub A, Erolin C, Rynn C, Wilkinson C. Validation of a computer modelled forensic facial reconstruction technique using CT data from live subjects: A pilot study. Forensic Sci Int. 2014;237: 147.e1-147.e8. <https://doi.org/10.1016/j.forsciint.2013.12.042>
27. Miranda GE, Wilkinson C, Roughley M, Beaini TL, Melani RFH. Assessment of accuracy and recognition of three-dimensional computerized forensic craniofacial reconstruction. PLoS One. 2018;13: e0196770. <https://doi.org/10.1371/journal.pone.0196770>
28. Wilkinson C. Forensic Facial Reconstruction. Cambridge: Cambridge University Press; 2004. pp. 151-6.
29. Stephan CN, Simpson EK. Facial soft tissue depths in craniofacial identification (part I): an analytical review of the published adult data. J Forensic Sci. 2008;53: 1257–1272. <https://doi.org/10.1111/j.1556-4029.2008.00852.x>
30. Stephan CN, Simpson EK. Facial soft tissue depths in craniofacial identification (part II): an analytical review of the published sub‐adult data. J Forensic Sci. 2008;53: 1273–1279. <https://doi.org/10.1111/j.1556-4029.2008.00853.x>
31. Stephan CN. 2018 tallied facial soft tissue thicknesses for adults and sub-adults. For Sci Int. 2017;280: 113–123. <https://doi.org/10.1016/j.forsciint.2017.09.016>
32. Phillips VM, Smuts NA. Facial reconstruction: Utilization of computerized tomography to measure facial tissue thickness in a mixed racial population. Forensic Sci Int. 1996;83: 51–59. <https://doi.org/10.1016/0379-0738(96)02010-5>.
33. Aulsebrook W, Becker P, İşcan MY. Facial soft-tissue thicknesses in the adult male Zulu. Forensic Sci Int 1996;79(2): 83–102.
34. Cavanagh D, Steyn M. Facial reconstruction: Soft tissue thickness values for South African black females. Forensic Sci Int. 2011;206: 215e1-e7. <https://doi.org/10.1016/j.forsciint.2011.01.009>.
35. Briers N, Briers TM, Becker PJ, Steyn M. Soft tissue thickness values for black and coloured South African children aged 6–13 years. Forensic Sci Int. 2015;252: 188.e1-e10. <https://doi.org/10.1016/j.forsciint.2015.04.015>.
36. Tawha T, Dinkele E, Mole C, Gibbon VE. Assessing zygomatic shape and size for estimating sex and ancestry in a South African sample. Sci Justice. 2020;60: 284-292. <https://doi.org/10.1016/j.scijus.2020.01.003>
37. Peckmann TR, Manhein MH, Listi GA, Fournier M. *In vivo* facial tissue depth for Canadian aboriginal children: A case study from Nova Scotia, Canada. J Forensic Sci. 2013;58: 1429–38. <https://doi.org/10.1111/1556-4029.12211>.
38. Manhein MH, Listi GA, Barsley RE, Musselman R, Barrow NE, Ubelaker DH. *In vivo* facial tissue depth measurements for children and adults. J Forensic Sci. 2000;45: 48–60. <https://doi.org/10.1520/JFS14640J>.
39. Sassouni V. Palatoprint, physioprint, and roentgenographic cephalometry, as new methods in human identification. J. Forensic Sci. 1957a;2: 428-442. Available at <https://archive.org/details/sim_journal-of-forensic-sciences_1957-01_2_1>
40. Sassouni V. A roentgenographic cephalometric analysis of cephalo-facio-dental relationships. Am J Orthod. 1955;41: 735-764. <https://doi.org/10.1016/0002-9416(55)90171-8>.
41. Sassouni V. Physical individuality and the problem of identification. Temple Law Quarterly. 1957b;31: 341.
42. Beistle RT. Sassouni plus*.* A comprehensive cephalometric system for diagnosis and treatment planning in functional therapy. Funct Orthod. 1984;1: 39–40.
43. Mahoney G, Milani C, Billinger M, Lywood V, Gruppioni G. Using a haptic device and virtual sculpting software for predicting a missing mandible: the case of Angelo Poliziano. Boll Soc Ital Biol Sper. 2012;85(1): 368–370. <https://doi.org/10.4081/jbr.2012.4170>
44. Ide K, Rynn C. Estimation of the mandibular dimensions from linear cranial measurements for use in craniofacial reconstruction: A preliminary study. Leg Med Tok. 2020;47: 101770. <https://doi.org/10.1016/j.legalmed.2020.101770>
45. Omran KA, Wertheim D, Smith K, Liu CYJ, Naini FB. Mandibular shape prediction using cephalometric analysis: applications in craniofacial analysis, forensic anthropology and archaeological reconstruction. Maxillofac Plast Reconstr Surg. 2020;42: 37. <https://jkamprs.springeropen.com/articles/10.1186/s40902-020-00282-3>
46. Morris AG. Missing and Murdered: A Personal Adventure in Forensic Anthropology. Cape Town: Zebra Press; 2011.
47. Schramm K. Stuck in the Tearoom: Facial Reconstruction and Postapartheid Headache. American Anthropologist (special section). 2020;122: 1–14. <https://doi.org/10.1111/aman.13384>
48. Johnson H. Craniofacial reconstruction and its socio-ethical implications. Museums & Social Issues. 2016;11: 97–113. <https://doi.org/10.1080/15596893.2016.1204598>
49. Hayes S. Faces in the museum: revising the methods of facial reconstructions. Museum Management and Curatorship. 2016;31: 218–245. <https://doi.org/10.1080/09647775.2015.1054417>
50. Harries I, Fibiger L, Smith J, Adler T, Szöke A. Exposure: the ethics of making, sharing and displaying photographs of human remains. Human Remains and Violence: An Interdisciplinary Journal. 2018;4: 3–24. [https://doi.org/ 10.7227/HRV.4.1.2](https://doi.org/%2010.7227/HRV.4.1.2)
51. Balachandran S. Among the dead and their possessions: A conservator’s role in the death, life, and afterlife of human remains and their associated objects. Journal of the American Institute for Conservation. 2009;48: 199–222. <https://doi.org/10.1179/019713612804514224>
52. J. Smith, *et al.*, One of Us? Navigating ‘rehumanization’ in the depiction and display of two ancient Egyptians from the Johns Hopkins Archaeological Museum. Proceedings of *Art, Materiality and Representation* (Royal Anthropological Institute, London, 2018), pp. 165–166.
53. Zhang Y. A survey on evaluation methods for image segmentation. Pattern Recognition. 1996;29(8): 1335-1346. <https://doi.org/10.1016/0031-3203(95)00169-7>.
54. Schlebusch CM, de Jongh M, Soodyall H. Different contributions of ancient mitochondrial and Y-chromosomal lineages in “Karretjie people” of the Great Karoo in South Africa. J Hum Genet. 2011;56: 623–630. <https://doi.org/10.1038/jhg.2011.71>.
55. Skoglund P, Thompson JC, Prendergast ME, Mittnik A, Sirak K, Hajdinjak M, et al. Reconstructing prehistoric African population structure. Cell. 2017;171: 59-71.e21. <https://doi.org/10.1016/j.cell.2017.08.049>.
56. Schlebusch CM, Malmström H, Günther T, Sjödin P, Coutinho A, Edlund H, et al. Southern African ancient genomes estimate modern human divergence to 350,000 to 260,000 years ago. Science. 2017;358: 652–655. <https://doi.org/10.1126/science.aao6266>.
57. Gallego Llorente M, Jones E, Eriksson A, Siska V, Arthur KW, Arthur JW, et al. Ancient Ethiopian genome reveals extensive Eurasian admixture throughout the African continent. Science. 2015;350: 820–822. <https://doi.org/10.1126/science.aad2879>.
58. Barbieri C, Vicente M, Rocha J, Mpoloka SW, Stoneking M, Pakendorf B. Ancient substructure in early mtDNA lineages of Southern Africa. Am J Hum Genet. 2013;92: 285–292. <https://doi.org/10.1016/j.ajhg.2012.12.010>.
59. Barbieri C, Vicente M, Oliveira S, Bostoen K, Rocha J, Stoneking M, et al*.* Migration and interaction in a contact zone: mtDNA variation among Bantu-speakers in Southern Africa. PLoS One. 2014;9: e99117. <https://doi.org/10.1371/journal.pone.009911>.
60. Chan EKF, Hardie RA, Petersen DC, Beeson K, Bornman RM, Smith AB, et al. Revised timeline and distribution of the earliest diverged human maternal lineages in Southern Africa. PLoS One. 2015;10: e0121223. <https://doi.org/10.1371/journal.pone.0121223>.
61. Uren C, Kim M, Martin AR, Bobo D, Gignoux CR, van Helden PD, et al. Fine-scale human population structure in Southern Africa reflects ecogeographic boundaries. Genetics. 2016;204: 303–314. <https://doi.org/10.1534/genetics.116.187369>.
62. Schlebusch CM, Lombard M, Soodyall H. MtDNA control region variation affirms diversity and deep sub-structure in populations from Southern Africa. BMC Evol Biol. 2013;13:56. <https://doi.org/10.1186/1471-2148-13-56>.
63. Pickrell JK, Patterson N, Barbieri C, Berthold F, Gerlach L, Güldemann T, et al. The genetic prehistory of southern Africa. Nat Commun. 2012;3: 1143. <https://doi.org/10.1038/ncomms2140>.
64. Vicente M, Jakobsson M, Ebbesen P, Schlebusch CM. Genetic affinities among Southern Africa hunter-gatherers and the impact of admixing farmer and herder populations. Mol Biol Evol. 2019;36: 1849–1861. <https://doi.org/10.1093/molbev/msz089>.
65. Schlebusch CM, Skoglund P, Sjödin P, Gattepaille LM, Hernandez D, Jay F, et al. Genomic variation in seven Khoe-San groups reveals adaptation and complex African history. Science. 2012;338: 374–379. <https://doi.org/10.1126/science.1227721>.Patterson N, Price AL, Reich D. Population structure and eigenanalysis. PLoS Genet. 2006;2: e190. <https://doi.org/10.1371/journal.pgen.0020190>.
66. Alpaslan-Roodenberg S, Anthony D, Babiker H, Bánffy E, Booth T, Capone P, et al. Ethics of DNA research on human remains: five globally applicable guidelines. Nature. 2021;599: 41 - 46. <https://doi.org/10.1038/s41586-021-04008-x>
67. Neumann GU, Valtuena AA, Fellows Yates JA, Stahl R, Brandt G. Tooth Sampling from the inner pulp chamber for ancient DNA Extraction. *protocols.io* (2020). <https://dx.doi.org/10.17504/protocols.io.bqebmtan>.
68. Dabney J, Knapp M, Glocke I, Gansauge MT, Weihmann A, Nickel B, et al. Complete mitochondrial genome sequence of a Middle Pleistocene cave bear reconstructed from ultrashort DNA fragments. Proc Natl Acad Sci U S A. 2013;110: 15758–15763. <https://doi.org/10.1073/pnas.1314445110>.
69. Velsko I, Skourtanioti E, Brandt G. Ancient DNA extraction from skeletal material. *protocols.io* (2020). <https://doi.org/10.17504/protocols.io.baksicwe>.
70. Meyer M, Kircher M. Illumina sequencing library preparation for highly multiplexed target capture and sequencing. Cold Spring Harb Protoc. 2010;6: pdb.prot5448. <https://doi.org/10.1101/pdb.prot5448>.
71. Kircher M, Sawyer S, Meyer M. Double indexing overcomes inaccuracies in multiplex sequencing on the Illumina platform. Nucleic Acids Res. 2012;40: e3. <https://doi.org/10.1093/nar/gkr771>.
72. Aron F, Neumann GU, Brandt G. Half-UDG treated double-stranded ancient DNA library preparation for Illumina sequencing. *protocols.io* (2020). <http://dx.doi.org/10.17504/protocols.io.bmh6k39e>.
73. Stahl R, Warinner C, Velsko I, Orfanou E, Aron F, Brandt G. Illumina double-stranded DNA dual indexing for ancient DNA. *protocols.io* (2020). <https://doi.org/10.17504/protocols.io.bakticwn>
74. Rohland N, Harney E, Mallick S, Nordenfelt S, Reich D. Partial uracil-DNA-glycosylase treatment for screening of ancient DNA. Philos Trans R Soc Lond B Biol Sci. 2015;370: 20130624. <https://doi.org/10.1098/rstb.2013.0624>.
75. Peltzer A, Jäger G, Herbig A, Seitz A, Kniep C, Krause J, et al. EAGER: efficient ancient genome reconstruction. Genome Biol. 2016;17: 60. <https://doi.org/10.1186/s13059-016-0918-z>
76. Li H, Durbin R. Fast and accurate short read alignment with Burrows-Wheeler transform. Bioinformatics. 2009;25: 1754–1760. <https://doi.org/10.1093/bioinformatics/btp324>.
77. Jónsson H, Ginolhac A, Schubert M, Johnson PLF, Orlando L. mapDamage2.0: fast approximate Bayesian estimates of ancient DNA damage parameters. Bioinformatics 2013;29: 1682–1684. <https://doi.org/10.1093/bioinformatics/btt193>
78. Lamnidis TC, Majander K, Jeong C, Salmela E, Wessman A, Moiseyev V, et al. Ancient Fennoscandian genomes reveal origin and spread of Siberian ancestry in Europe. Nat Commun. 2018;9: 5018. <https://doi.org/10.1038/s41467-018-07483-5>.
79. Korneliussen TS, Albrechtsen A, Nielsen R. ANGSD: Analysis of next generation sequencing data. BMC Bioinformatics. 2014;15: 356. <https://doi.org/10.1186/s12859-014-0356-4>.
80. Renaud G, Slon V, Duggan AT, Kelso J. Schmutzi: estimation of contamination and endogenous mitochondrial consensus calling for ancient DNA. Genome Biol. 2015;16: 224. <https://doi.org/10.1186/s13059-015-0776-0>.
81. Li H, Handsaker B, Wysoker A, Fennell T, Ruan J, Homer N, et al*.* The Sequence Alignment/Map format and SAMtools. Bioinformatics. 2009;25: 2078–2079. <https://doi.org/10.1093/bioinformatics/btp352>.
82. Kearse M, Moir R, Wilson A, Stones-Havas S, Cheung M, Sturrock S, et al. Geneious Basic: An integrated and extendable desktop software platform for the organization and analysis of sequence data. Bioinformatics. 2012;28: 1647–1649. <https://doi.org/10.1093/bioinformatics/bts199>.
83. Weissensteiner H, Pacher D, Kloss-Brandstätter A, Forer L, Specht G, Bandelt HJ, et al. HaploGrep 2: Mitochondrial haplogroup classification in the era of high-throughput sequencing. Nucleic Acids Res. 2016;44: W58-63. <https://doi.org/10.1093/nar/gkw233>
84. Kuhn JMM, Jakobsson M, Günther T. Estimating genetic kin relationships in prehistoric populations. PLoS One. 2018;13: e0195491. <https://doi.org/10.1371/journal.pone.0195491>.
85. Lazaridis I, Nadel D, Rollefson G, Merrett DC, Rohland N, Mallick S, et al. Genomic insights into the origin of farming in the ancient Near East. Nature. 2016;536: 419–424. <https://doi.org/10.1038/nature19310>.
86. Meyer M, Kircher M, Gansauge MT, Li H, Racimo F, Mallick S, et al. A high-coverage genome sequence from an archaic Denisovan individual. Science. 2012;338: 222–226. <https://doi.org/10.1126/science.1224344>.
87. Prüfer K, Racimo F, Patterson N, Jay F, Sankararaman S, Sawyer S, et al. The complete genome sequence of a Neanderthal from the Altai Mountains. Nature. 2014;505: 43–49. <https://doi.org/10.1038/nature12886>.
88. Fu Q, Li H, Moorjani P, Jay F, Slepchenko SM, Bondarev AA, et al. Genome sequence of a 45,000-year-old modern human from western Siberia. Nature. 2014;514: 445–449. <https://doi.org/10.1038/nature13810>.
89. Mathieson I, Lazaridis I, Rohland N, Mallick S, Patterson N, Roodenberg SA, et al. Genome-wide patterns of selection in 230 ancient Eurasians. Nature. 2015;528: 499–503. <https://doi.org/10.1038/nature16152>.
90. Lazaridis I, Patterson N, Mittnik A, Renaud G, Mallick S, Kirsanow K, et al. Ancient human genomes suggest three ancestral populations for present-day Europeans. Nature. 2014;513: 409–413. <https://doi.org/10.1038/nature13673>.
91. Haak W, Lazaridis I, Patterson N, Rohland N, Mallick S, Llamas B, et al. Massive migration from the steppe was a source for Indo-European languages in Europe. Nature. 2015;522: 207–211. <https://doi.org/10.1038/nature14317>.
92. Mallick S, Li H, Lipson M, Mathieson I, Gymrek M, Racimo F, et al. The Simons Genome Diversity Project: 300 genomes from 142 diverse populations. Nature. 2016;538: 201–206. <https://doi.org/10.1038/nature18964>.
93. Schlebusch CM, Prins F, Lombard M, Jakobsson M, Soodyall H. The disappearing San of southeastern Africa and their genetic affinities. Hum Genet. 2016;135: 1365–1373. <https://doi.org/10.1007/s00439-016-1729-8>.
94. Pickrell JK, Pritchard JK. Inference of population splits and mixtures from genome-wide allele frequency data. PLoS Genet. 2012;8: e1002967. <https://doi.org/10.1371/journal.pgen.1002967>.
95. Alexander DH, Novembre J, Lange K. Fast model-based estimation of ancestry in unrelated individuals. Genome Res. 2009;19: 1655–1664. <https://doi.org/10.1101/gr.094052.109>.
96. Mittnik A, Wang CC, Pfrengle S, Daubaras M, Zariņa G, Hallgren F, et al. The genetic prehistory of the Baltic Sea region. Nat Commun*.* 2018;9: 442. <https://doi.org/10.1038/s41467-018-02825-9>.
97. Patterson N, Moorjani P, Luo Y, Mallick S, Rohland N, Zhan Y, et al. Ancient admixture in human history. Genetics. 2012;192: 1065–1093. <https://doi.org/10.1534/genetics.112.145037>.
98. Vogel JG, Fuls A, Ellis RP. The geographical distribution of Kranz grasses in South Africa. South African Journal of Science. 1978;74: 209-215.
99. Knapp AK, Chen A, Griffin-Nolan RJ, Baur LE, Carroll CJ, Gray JE, et al. Resolving the Dust Bowl paradox of grassland responses to extreme drought. Proceedings of the National Academy of Sciences. 2020;117: 22249-22255. <https://doi.org/10.1073/pnas.1922030117>.
100. Diefendorf AF, Mueller KE, Wing SL, Koch PL, Freeman KH. Global patterns in leaf 13C discrimination and implications for studies of past and future climate. Proc Natl Acad Sci USA. 2010;107: 5738-5743. <https://doi.org/10.1073/pnas.0910513107>.
101. Kohn MJ. Carbon isotope compositions of terrestrial C3 plants as indicators of (paleo) ecology and (paleo) climate. Proc Natl Acad Sci USA*.* 2010;107: 19691-19695. <https://doi.org/10.1073/pnas.1004933107>.
102. Cornwell WK, , A global dataset of leaf delta 13C values. Zenodo. <https://doi.org/10.5281/zenodo.569501>. Deposited 27 April 2017.
103. Keeling RF, Graven HD, Welp LR, Resplandy L, Bi J, Piper SC, et al. Atmospheric evidence for a global secular increase in carbon isotopic discrimination of land photosynthesis. Proc Natl Acad Sci USA. 2017;114: 10361-10366. <https://doi.org/10.1073/pnas.1619240114>.
104. Beaumont J, Montgomery J. The Great Irish Famine: Identifying starvation in the tissues of victims using stable isotope analysis of bone and incremental dentine collagen. PLoS One. 2016;11: e0160065. <https://doi.org/10.1371/journal.pone.0160065>.
105. Doi H, Akamatsu F, González AL.Starvation effects on nitrogen and carbon stable isotopes of animals: An insight from meta-analysis of fasting experiments*.* R Soc Open Sci. 2017;4: 170633. <https://doi.org/10.1098/rsos.170633>.
106. Howland MR, Corr LT, Young SM, Jones V, Jim S, Van Der Merwe NJ, et al. Expression of the dietary isotope signal in the compound-specific δ^13^C values of pig bone lipids and amino acids. Int J Osteoarchaeol. 2003;13: 54-65. <https://doi.org/10.1002/oa.658>.
107. Jim S, Jones V, Ambrose SH, Evershed RP. Quantifying dietary macronutrient sources of carbon for bone collagen biosynthesis using natural abundance stable carbon isotope analysis. Br J Nutr. 2006;95: 1055-1062. <https://doi.org/10.1079/bjn20051685>.
108. DeNiro MJ, Schoeninger MJ. Stable carbon and nitrogen isotope ratios of bone collagen: Variations within individuals, between sexes, and within populations raised on monotonous diets. J Archaeol Sci. 1983;10: 199-203. <https://doi.org/10.1016/0305-4403(83)90002-X>.
109. Plomp E, von Holstein IC, Kootker LM, Verdegaal‐Warmerdam SJ, Forouzanfar T, Davies GR. Strontium, oxygen, and carbon isotope variation in modern human dental enamel. Am J Phys Anthropol. 2020;172: 586-604. <https://doi.org/10.1002/ajpa.24059>.
110. Sealy J, Pfeiffer S, Yates R, Willmore K, Manhire A, Maggs T, et al. Hunter-gatherer child burials from the Pakhuis mountains, Western Cape: Growth, diet and burial practices in the Late Holocene. South African Archaeological Bulletin. 2000;55: 32-43. <https://doi.org/10.2307/3888890>.
111. Beaumont J, Montgomery J. Oral histories: A simple method of assigning chronological age to isotopic values from human dentine collagen. Ann Hum Biol. 2015;42: 407-414. <https://doi.org/10.3109/03014460.2015.1045027>.
112. Makarewicz AA, Sealy J. Dietary reconstruction, mobility, and the analysis of ancient skeletal tissues: Expanding the prospects of stable isotope research in archaeology. J Archeol Sci. 2015;56: 146-158. <http://dx.doi.org/10.1016/j.jas.2015.02.0350305-4403/>.
113. Kelly RL. The Lifeways of Hunter-Gatherers: The Foraging Spectrum. 2nd ed. Cambridge: Cambridge University Press; 2013.
114. Lee-Thorp JA, Manning L, Sponheimer M. Problems and prospects for carbon isotope analysis of very small samples of fossil tooth enamel. Bulletin de la Societé Geologique de France. 1997;168: 767-773.
