# Supplementary material for: Confronting historical legacies of biological anthropology in South Africa—Restitution, redress and community-centered science: The Sutherland Nine
Source: PLoS One. 2023 May 24;18(5):e0284785. doi: 10.1371/journal.pone.0284785 (PMC10208512; doi:10.1371/journal.pone.0284785)
Supplement: S1 Raw images — (PDF) [file pone.0284785.s036.pdf]

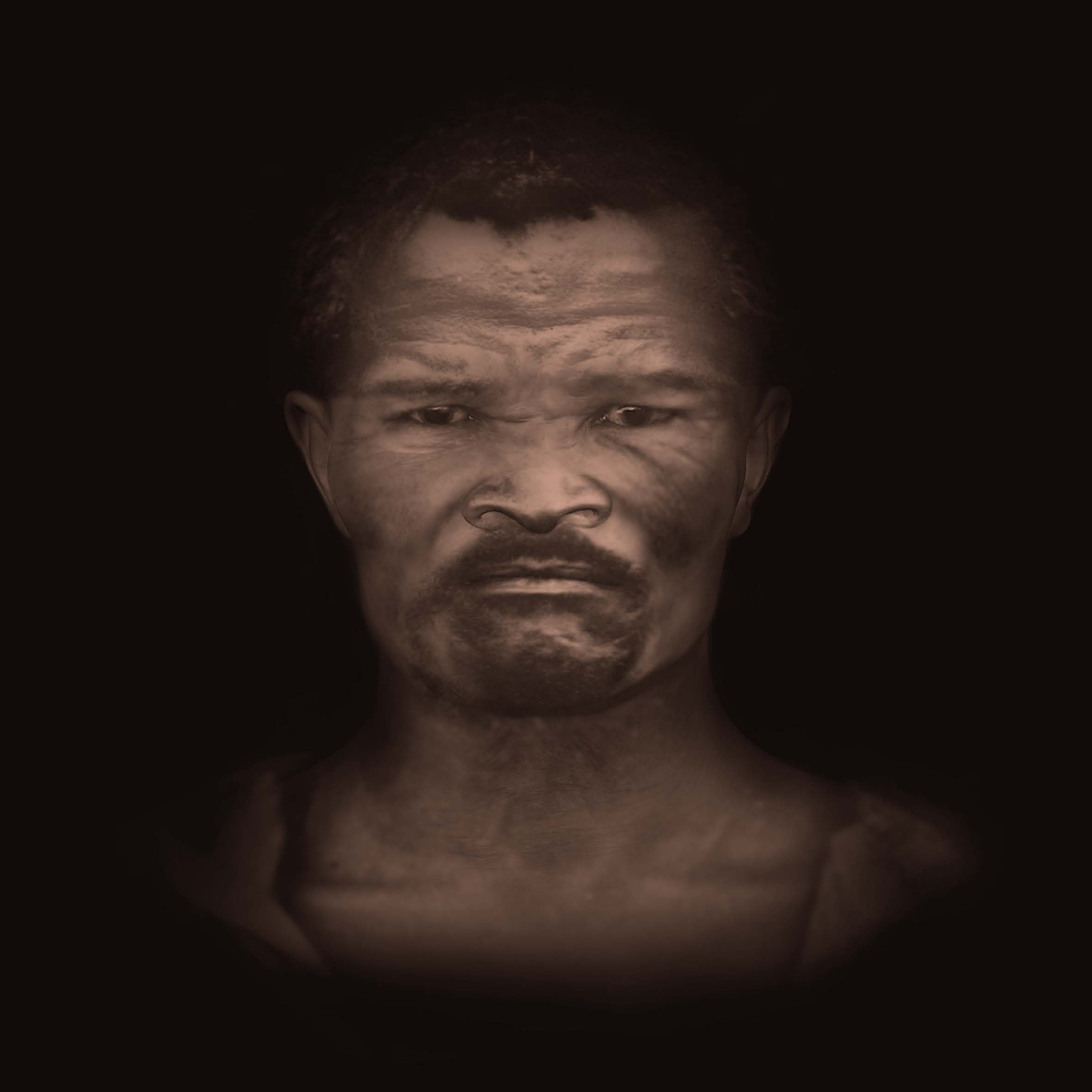

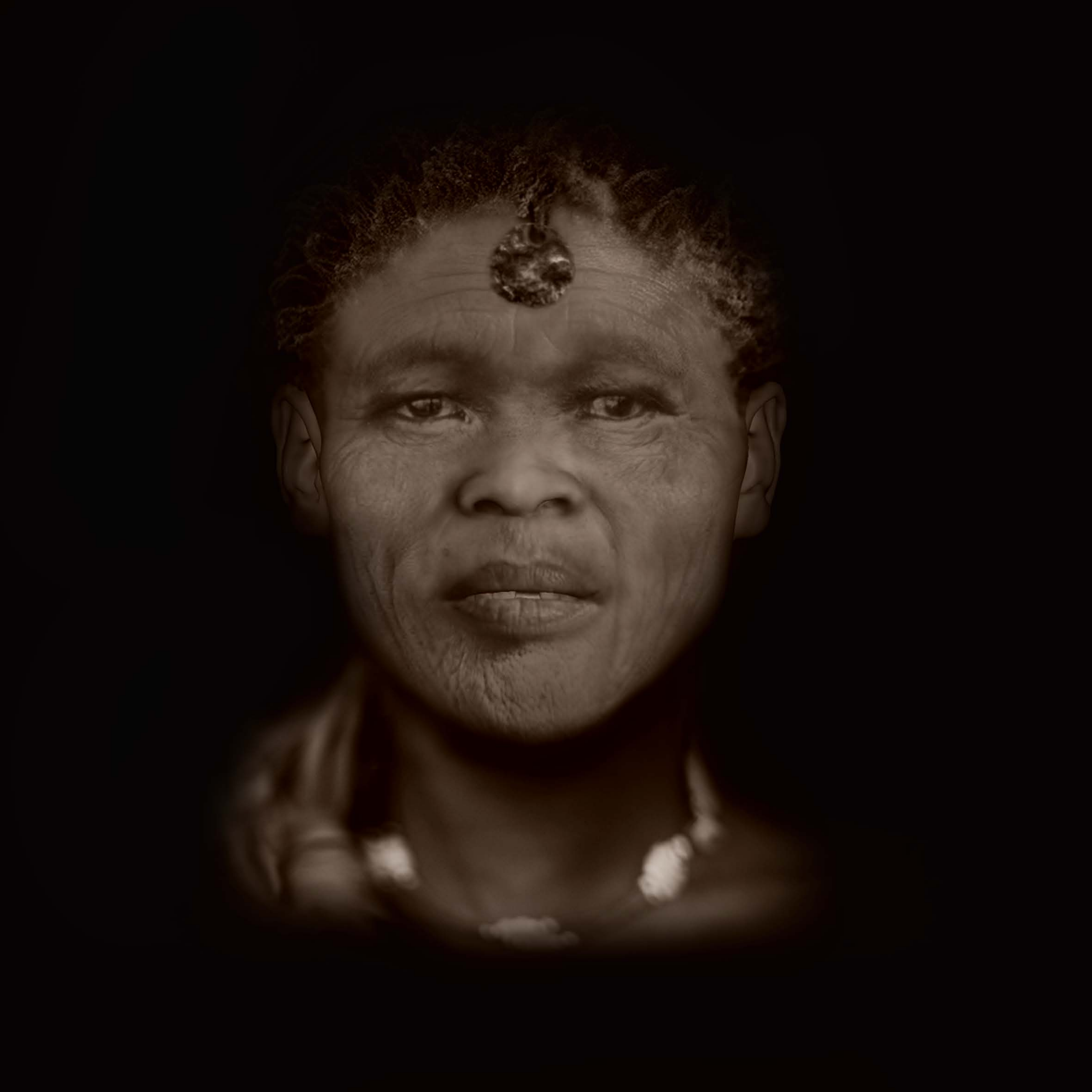

Cover option 1: Nearly a hundred years later, the human remains of the Sutherland Nine are being returned to their community, accompanied by a range of community-driven studies to document as far as possible their lives and deaths. These individuals were exhumed as specimens but will be reburied as people. This image is of a facial reconstruction of Voetje a 2.5D still frontal screenshot from his 3D model with photographic texture applied using digital montage techniques. The facial reconstruction and depiction were supported through a National Geographic Society Explorer grant (EC-60001R-19) Liverpool John Moores University QR fund and UCT.

Cover option 2: Nearly a hundred years later, the human remains of the Sutherland Nine are being returned to their community, accompanied by a range of community-driven studies to document as far as possible their lives and deaths. These individuals were exhumed as specimens but will be reburied as people. This image is of a facial reconstruction of Saartje a 2.5D still frontal screenshot from his 3D model with photographic texture applied using digital montage techniques. The facial reconstruction and depiction were supported through a National Geographic Society Explorer grant (EC-60001R-19) Liverpool John Moores University QR fund and UCT.
